# Mechanistic Insight into the Thermal “Blueing” of Cyanine Dyes

**DOI:** 10.1021/jacs.4c02171

**Published:** 2024-07-11

**Authors:** Aria Vahdani, Mehdi Moemeni, Daniel Holmes, Richard R. Lunt, James E. Jackson, Babak Borhan

**Affiliations:** ^‡^Department of Chemistry^§^Department of Chemical Engineering, Michigan State University, East Lansing, Michigan 48824, United States

## Abstract

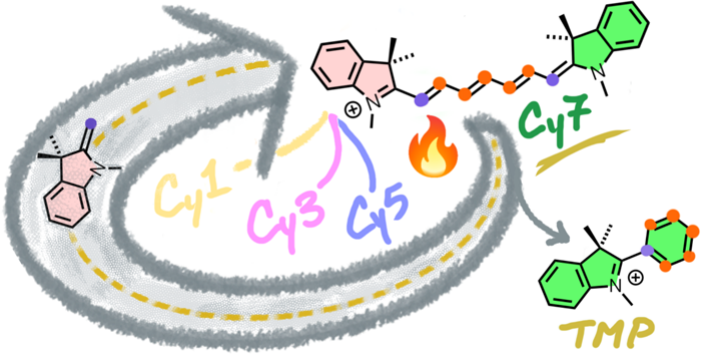

In recent work to develop cyanine dyes with especially large Stokes shifts, we encountered a “blueing” reaction, in which the heptamethine cyanine dye **Cy7** (IUPAC: 1,3,3-trimethyl-2-((1*E*,3*E*,5*E*)-7-((*E*)-1,3,3-trimethylindolin-2-ylidene)hepta-1,3,5-trien-1-yl)-3*H*-indol-1-ium) undergoes shortening in two-carbon steps to form the pentamethine (**Cy5**) and trimethine (**Cy3**) analogs. Each step blue-shifts the resulting absorbance wavelength by ca. 100 nm. Though photochemical and oxidative chain-shortening reactions had been noted previously, it is simple heating alone or with amine bases that effects this unexpected net C_2_H_2_ excision. Explicit acetylene loss would be too endothermic to merit consideration. Our mechanistic studies using ^2^H labeling, mass spectrometric and NMR spectroscopic analyses, and quantum chemical modeling point instead to electrocyclic closure and aromatization of the heptamethine chain in **Cy7** forming Fischer’s base **FB** (1,3,3-trimethyl-2-methyleneindoline), a reactive carbon nucleophile that initiates chain shortening of the cyanine dyes by attack on their polymethine backbones. The byproduct is the cationic indolium species **TMP** (IUPAC: 1,3,3 trimethyl-2-phenyl indolium).

## Introduction

Organic fluorescent dyes have been instrumental in areas ranging from cellular biology^1−12^ and drug design^13,14^ to advanced materials engineering^7,15−27^ including energy capture and conversion.^19,28−30^ Key to continued progress in this field is the ability to tune the chromophores’ structural and photophysical properties to suit their target applications. Among the most widely utilized dye families, cyanines boast high brightness and easy synthetic tunability.^31^ Cyanine dyes are typically categorized based on the (odd) number of methine units in their electronic push–pull structure between the terminating alkyl-indolenine/indolium groups. Monomethine (**Cy1**) and trimethine (**Cy3**) absorb and emit light in the UV–vis range,^3,32^ while the longer pentamethine (**Cy5**) and heptamethine (**Cy7**) cyanines span from visible to near-infrared (NIR) wavelengths,^9,29,31,33−36^ where the light’s deep tissue penetration and the dyes’ minimal autofluorescence enable their use for *in vivo* imaging and photodynamic therapy.^4,5,10,11,37−41^ Recently, because their absorption range covers a substantial fraction of the solar spectrum, **Cy5** and **Cy7** dyes have also been developed for energy harvesting.^23,28^

**Figure 1:**
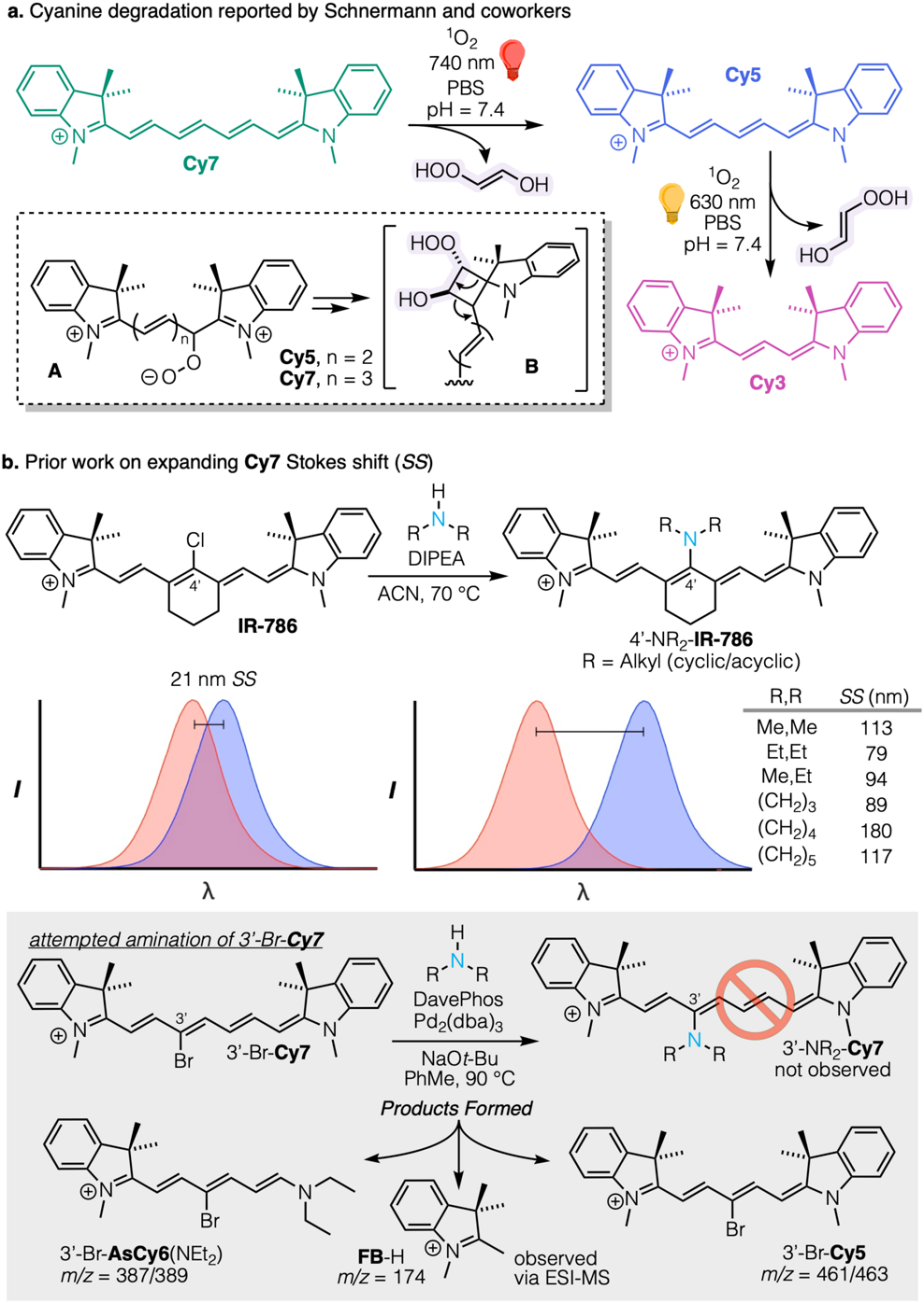
(a) Prior work by Schnermann et al. on oxygen-mediated cyanine phototruncation; (b) nucleophilic chloride displacement from **IR-786**, a centrally constrained **Cy7**, by 2° amines to yield 4’-aminated **IR-786** dyes with large Stokes shifts; (c) attempted Buchwald amination of 3’-Br-**Cy7** and the isolated products 3’-Br-**Cy5**, 3’-Br-**AsCy6**(NEt_2_) along with detection of **FB** (as **FB**-H^+^ via ESI-MS).

As polyene iminium chromophores, the cyanine dyes show substantial chemical reactivity.^5,13,20,42−45^ Their degradation involving singlet oxygen has been investigated in recent decades, leading to novel insights and methods. For instance, Schnermann et al. found that *in situ* photooxidation of **Cy7** systems yields truncated cyanines via net C_2_H_2_ excision from the parent polymethine chromophore.^5^ The resulting hypsochromic shift, dubbed “photoblueing”, has potential applications in super-resolution imaging and single-particle tracking.^40,43,46^ As first discovered, this reaction gave <2% yield of the phototruncated product **Cy5**, but by raising the pH to 9.5 in 1 M CAPSO buffer, the Schnermann group was able to increase the observed truncation yield to 17.2% (Figure 1a).^4^

Our own work^47−49^ has recently extended the previously reported strategy^50,51^ of attaching donor substituents midchromophore to tune the heptamethine cyanines’ Stokes shift, as shown in Figure 1b. Specifically, the synthesis entailed displacement of a halide by a secondary amine at C4’ of a **Cy7** framework. Noting the large spectral changes brought on by this substitution, we sought to compare the effects of installing similar donors in the C3’ position, which lacks the conjugation pathways of the C4’ location. The required 3’-Br-**Cy7** was readily accessed via Klan’s practical route to backbone functionalized heptamethine cyanines via pyridinium salt (Zincke) ring opening.^31,34^ However, upon attempting Buchwald amination with diethylamine (Figure 1c), instead of the target 3’-aminated **Cy7**, we isolated the symmetric polymethine 3’-Br-**Cy5**, an apparent result of C_2_H_2_ loss from the parent cyanine. The other significant product observed was Fischer’s Base (1,3,3-trimethyl-2-methyleneindoline, designated herein as **FB**). Further investigation found that these transformations occurred with the amine components alone, omitting the Buchwald catalyst and excluding both molecular oxygen and light. They thus follow a path different from that reported by Schnermann and others.^4,5^

Herein, we report a mechanistic analysis of this base-promoted, anaerobic, thermal shortening of cyanine dyes. We use the “**Cy**” names to denote the unfunctionalized *N*-methyl indoline/indolium polymethine structures as shown in Figure 1a; these share the core chromophoric framework found in the commercial “**Cy7**” dye (sulfonated on the indole moieties and bearing *N*-alkyl sulfonate side chains). Furthermore, the counteranion to all of the cationic structures in this manuscript are iodide (omitted in the structures), unless specified otherwise. The conclusion is that **Cy7** is unique; even in the absence of other reagents, it can undergo facile electrocyclization and aromatization. This process forms a new species, **TMP** (IUPAC: 1,3,3 trimethyl-2-phenyl indolium) and liberates free **FB**, which can then nucleophilically attack **Cy7** and related polymethine cyanines, shortening their polyene backbones. Like **FB**, secondary amines can react with cyanines, generating asymmetric analogs of varying length. A byproduct of this mechanistic analysis is a practical method for the “blueing” of cyanine dyes and their respective symmetric and asymmetric derivatives. We note the beautiful work of Klan and co-workers that describes findings related to those described in this report. Professor Klan and his team were gracious enough to wait to submit findings at the same time as ours; the reader is referred to the accompanying manuscript in this journal.^52^

## Results and Discussion

### Cyanine Reactions with Amines

As noted above, we initially observed the **Cy7** to **Cy5** two-carbon truncation in the context of attempted Buchwald amination of 3’-Br-**Cy7** (Figure 1c). With or without the Buchwald catalyst, treatment with diethylamine (10 equiv) and Hunig’s base, (diisopropylethylamine, DIPEA, 2 equiv) at 70 °C in acetonitrile took a surprising direction. In addition to free **FB**, ESI-MS revealed two new polyene products with molecular ions *m*/*z* = 387/389 and *m*/*z* = 461/463, corresponding to 3’-Br-**AsCy6**(NEt_2_) and 3’-Br-**Cy5**, respectively (Figure 1c). Note a point of usage here: “**AsCy6**(NEt_2_)” designates the asymmetric product with the secondary amine as one end group of a hexamethine chain. These new derivatives were isolated, purified, and structurally analyzed by NMR, HRMS, and ESI-MS. Interestingly, small amounts of dehalogenated products **Cy7**, **Cy5**, and **Cy3** were also noted (see Figure S2 for full details of the products).

**Figure 2:**
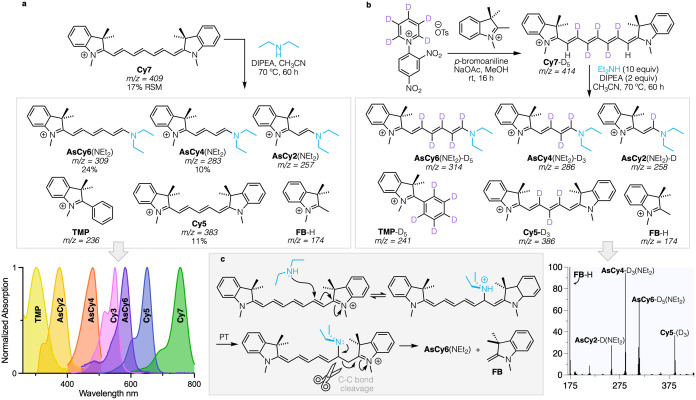
(a) Reaction of **Cy7** with DIPEA and diethylamine (70 °C, 60 h) to yield chain-shortened dyes, both symmetric and asymmetric. Yields are given for isolated products, along with the amount of **Cy7** recovered after 60 h. (b) Synthesis of **Cy7**-(D_5_) and its truncation products corresponding to the penta, tri, and mono labeled **AsCy** products seen with the unlabeled **Cy7**. (c) Proposed mechanism for amine attack on **Cy7** liberating **FB**, and **AsCy6**(NEt_2_).

Omitting the halogen to simplify the system, we then examined **Cy7** itself (*m*/*z* = 409). Treatment with diethylamine and DIPEA (Figure 2a) yielded the analogous **AsCy6**(NEt_2_) polymethine in addition to chain shortened species **AsCy4**(NEt_2_) and **AsCy2**(NEt_2_). As with the previously isolated 3’-Br-**Cy5**, pentamethine **Cy5** was also found in this reaction. We also noted the formation of two monomeric indolines, cationic 1,1,3-trimethyl-2-phenyl indolium (**TMP**, *m*/*z* = 236) and Fischer base itself, **FB** (observed by ESI-MS as **FB**-H). To track the polymethine components of the parent cyanine, pentadeutero **Cy7**-D_5_ was synthesized as depicted in Figure 2b. Its treatment with diethylamine and DIPEA led to isolation of centrally trideuterated **Cy5**-D_3_, along with the corresponding **AsCy**[2,4, and 6](NEt_2_) species, mono-, tri-, and pentadeuterated, respectively (see Supporting Information for characterization). Notably, here, the **TMP** formed retained all five deuterium atoms, while the **FB** showed none.

The above treatment of **Cy7** with a mixture of DIPEA and diethylamine gave the isolated products **AsCy6**(NEt_2_) (*m*/*z* = 309) and **Cy5** (*m*/*z* = 383) in a ca. 2:1 ratio (Table 1, entry 1) and 35% overall yield. Further simplifying the reaction conditions to probe the role of each component, **Cy7** was treated with diethylamine alone (10 equiv; Table 1, entry 2) giving a higher selectivity for **AsCy6**(NEt_2_) and higher yield (45% after 60 h at 70 °C). The analogous room temperature reaction was slower, but after 160 h the yield was higher (73%), with similar selectivity (Table 1, entry 3). At even longer times, the **AsCy6**(NEt_2_) was converted to **AsCy4**(NEt_2_) and on to **AsCy2**(NEt_2_). With 30 equiv of diethylamine, the preference for **AsCy6**(NEt_2_) over **Cy5** increased, as did the chain-shortening processes (Figure S3). At 70 °C, reaction of **Cy7** with 10 equiv of another secondary amine, morpholine, was faster, giving a 51% yield in 20 h with an **AsCy6**(Morph):**Cy5** selectivity of 3.6:1 (Table 1, entry 4). All the above reaction mixtures also showed substantial amounts of **FB**, the end group displaced by the secondary amine. Importantly, the polyene chain shortening such as **AsCy6**(NEt_2_) → **AsCy4**(NEt_2_) seen in these reaction mixtures also suggested the thermochemically reasonable (*vide infra*) release of the C_2_H_2_ fragments in the form of vinyldialkylamines.

**Table 1:**
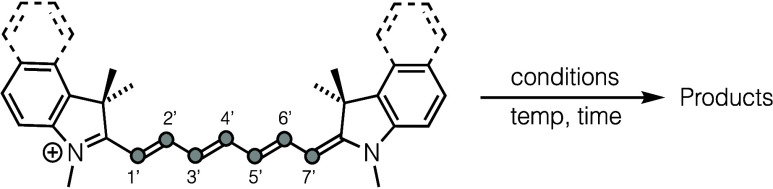
Summary of Reactions with Isolated yields^a^

entry	cyanine	nucleophile	3° amine	solvent	temp (°C)	time (h)	isolated products (yield)^b^
1^c^	**Cy7**	HNEt_2_	DIPEA	ACN	70	60	**Cy5** (11%), **AsCy6**(NEt_2_) (24%)
2	**Cy7**	HNEt_2_		ACN	70	60	**Cy5** (12%), **AsCy6**(NEt_2_) (33%)
3	**Cy7**	HNEt_2_		ACN	RT	160	**Cy5** (17%), **AsCy6**(NEt_2_) 56%)
4	**Cy7**	morpholine		ACN	RT	20	**Cy5** (11%), **AsCy6**(Morph) (30%)
5	**Cy7**		DIPEA	ACN	70	60	**Cy5** (18%), **Cy3** (2%)
6	**Cy7**		DIPEA	ACN	100	48	**Cy5** (51%), **Cy3** (3%)
7	**Cy7**		DIPEA	ACN	180	3	**Cy5** (38%), **Cy3** (8%)
8	**Cy7**		DIPEA	EtOH	70	60	**Cy5** (2%)
9	**Cy7**		DIPEA	CH_2_Cl_2_	70	60	**Cy5** (26%), **Cy3** (2%)
10	**Cy7**		quinuclidine	ACN	70	60	**Cy5** (5%), **Cy3** (15%)
11	**Cy7**		quinuclidine	ACN	100	36	**Cy5** (10%), **Cy3** (24%)
12	**Cy7.5**		DIPEA	ACN	100	40	**Cy5.5** (25%), **Cy3.5** (17%)
13	**IR-786**	**FB**		ACN	100	48	**Cy3** (30%)
14^d^	**Cy7**			ACN	100	60	**Cy5**, **Cy3**, **TMP**, **FB**

a Reactions were run in sealed vessels on a 0.04 mmol scale in degassed solvents [0.03 M cyanine dye] with 10 equiv of additive (amine or **FB**) used.

b Note that **FB** and **Cy7** were observed for all reactions with the exception of **Cy7** for entries 10 and 11 (with quinuclidine). Recovered **Cy7** yields for entries 1–4 are 17%, 15%, 10%, and 32%, respectively.

c Diethyl amine (10 equiv) and DIPEA (2 equiv). See the SI for other products identified by ESI-MS.

d Products were identified by MS and crude NMR.

**Figure 3:**
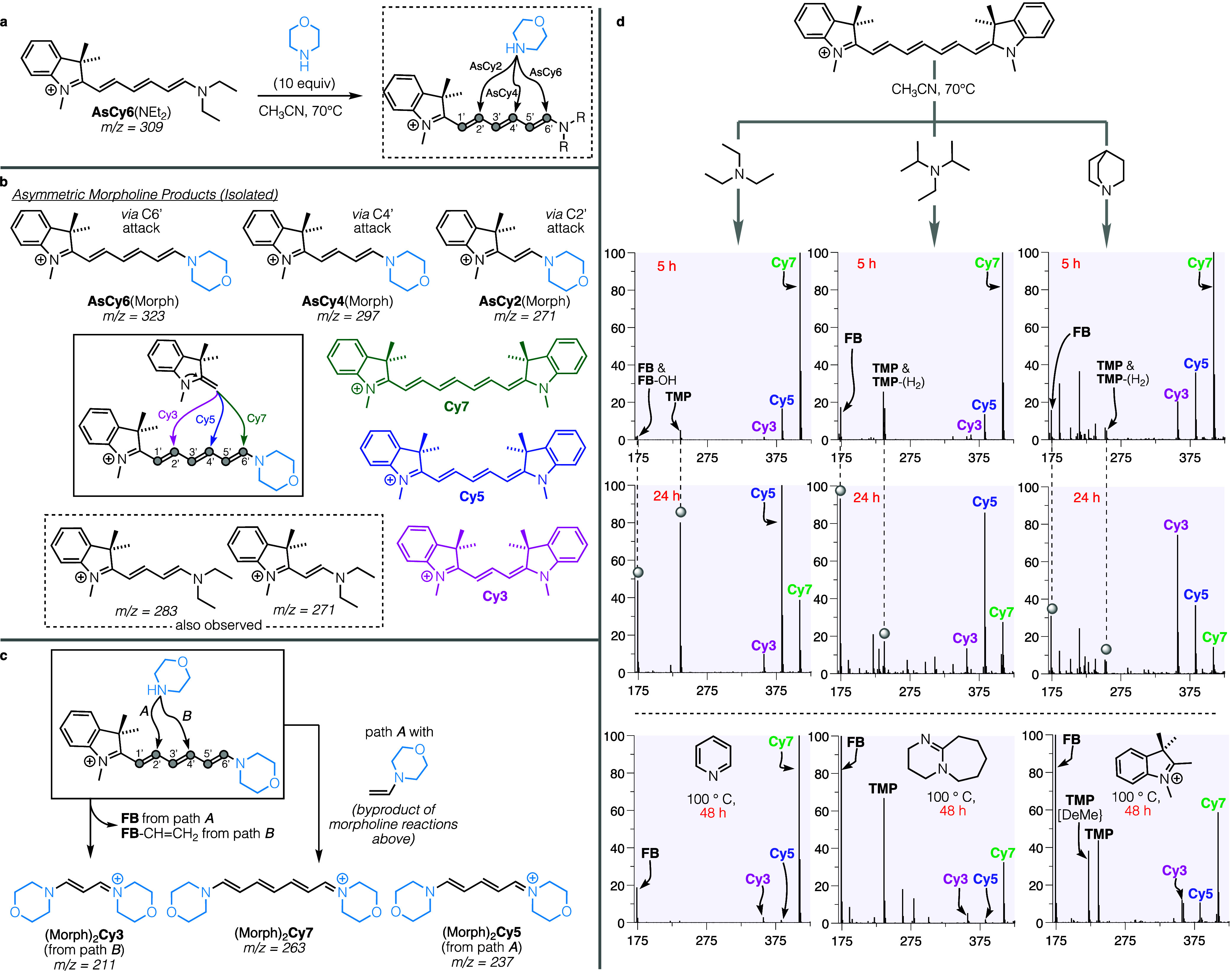
(a) Attack pathways and (b) products from morpholine attack on **AsCy6**(NEt_2_) at 70 °C, which opens up pathways of **FB** attacking **AsCy6**(Morph) leading to formation of **Cy3**, **Cy5**, and **Cy7.** (c) Dimorpholine (Morph)_2_-polyene fragments identified by ESI-MS. Of particular note here is the presence of (Morph)_2_**Cy7**, implying addition of vinylmorpholine. (d) Reaction of **Cy7** in the presence of different 3° amines and **FB**-H iodide salt.

To further probe these end-group exchange events, we reacted pure **AsCy6**(NEt_2_) with morpholine (10 equiv), the secondary amine depicted in Figure 3a. Almost instantaneously, exchange of **AsCy6**(NEt_2_) (*m*/*z* = 309) to **AsCy6**(Morph) (*m*/*z* = 323) was observed, along with small amounts of **Cy3**, **Cy5**, and **Cy7** (Figure 3b). We envision formation of these cyanines via liberation of **FB** from **AsCy6**(NR_2_) by free morpholine forming conjugated diamines (denoted Morph_2_**CyX**, X = 3, 5, and 7; see Figure 3c). The liberated **FB** in turn forms **Cy7** by displacing HNR_2_ from **AsCy6**(NR_2_) at C6’ (see numbering in Figure 3b). Attack at C4’ leads to **Cy5** and vinylamine (R_2_NCH=CH_2_) and analogously at C2’, giving **Cy3** and the corresponding dienylamine. Particularly noteworthy is the observation of Morph_2_**Cy7**, which implies displacement of an end group by N-vinylmorpholine (Figure 3c). These ready exchanges of secondary amines, **FB**, and vinylamines produce a diverse mixture of polymethines with increasing proportions of the chain-shortened products over time.

Having noted a greater preference for the truncated product **Cy5** over **AsCy6**(NEt_2_) when DIPEA was present, we next studied this tertiary amine acting alone on cyanines. Treated with only DIPEA (10 equiv) at 70 °C for 60 h, **Cy7** reacted slowly to form 18% **Cy5**, along with 2% of the further truncated trimethine **Cy3** (Table 1, entry 5). Higher temperatures accelerated polyene shortening (Table 1, entries 5–7). As in the previous reactions, we noted the early formation of indolines **TMP** and **FB** via ESI-MS. Replacement of the initially studied acetonitrile with the protic solvent ethanol dramatically decreased the yield of **Cy5**; only about 2% **Cy5** was isolated after 60 h at 70 °C with 10 equiv of DIPEA. On the other hand, the **Cy5** yield improved to 26% when the same reaction was run in dichloromethane (Table 1, entries 8 and 9). Lacking further evidence, we have added the speculation that both the DIPEA and any **FB** formed are inhibited by hydrogen bonding interactions with hydroxylic solvents, whereas in DCM, their full reactivity is available for attack on the less solvent-stabilized **Cy7**.

Beyond solvent and temperature, the next parameter explored was the choice of tertiary amine. Reaction of **Cy7** with triethylamine (Figure 3d) gave results like those from DIPEA treatment, albeit slightly faster. Other tertiary amines gave similar, but not identical, behavior. For instance, after 48 h at 100 °C with pyridine or DBU, (1,8-diazabicyclo[5.4.0]undec-7-ene) **Cy7** was largely unconsumed; only trace chain shortened polymethines **Cy5** and **Cy3** were detected, along with the now familiar **TMP** and **FB**. Conversion of **Cy7** using quinuclidine gave more rapid chain truncation; after 60 h at 70 °C, yields of **Cy5** and **Cy3** were 5% and 15%, respectively, with 13% recovery of **Cy7**. At 100 °C, **Cy7** was nearly consumed in 36 h, yielding 10% and 24% of **Cy5** and **Cy3**, together with **TMP** and **FB**. Here, the majority product was **Cy3**, not **Cy5** (compare Table 1, entries 10, 11 vs entries 5–7). In this case, the observation of a large *m*/*z* peak at 435 (see Figure S4), corresponding to nonamethine **Cy9**, was of particular importance. As **FB** is the nucleophile that effects end group exchange, the byproduct implied by chain shortening is the vinylated Fischer base, **FB**-CH=CH_2_. Like **FB** itself, this dienamine species may attack and displace **FB**, leading to polyene chain lengthening. This finding of chain extension is analogous to the observation of (Morph)_2_**Cy7** above (Figure 3c), implying the transfer of vinylated end groups.

The above **Cy7** reactions offer selectivity to a range of symmetric/asymmetric polymethines with varying degrees of conjugation, and thus absorption/emission wavelengths ranging from the NIR to UV (Figure S1, Figure 2a). This conversion has potential for useful applications as highlighted by Schnermann et al. in the context of their related photoactivated truncations.^4,5^ However, mechanisms by which cyanines could undergo the above anaerobic, thermal C_2_H_2_ excisions are not obvious. Thus, the activation and fate of the C_2_H_2_ fragment must be considered as part of any proposed chain-shortening mechanism.

**Figure 4:**
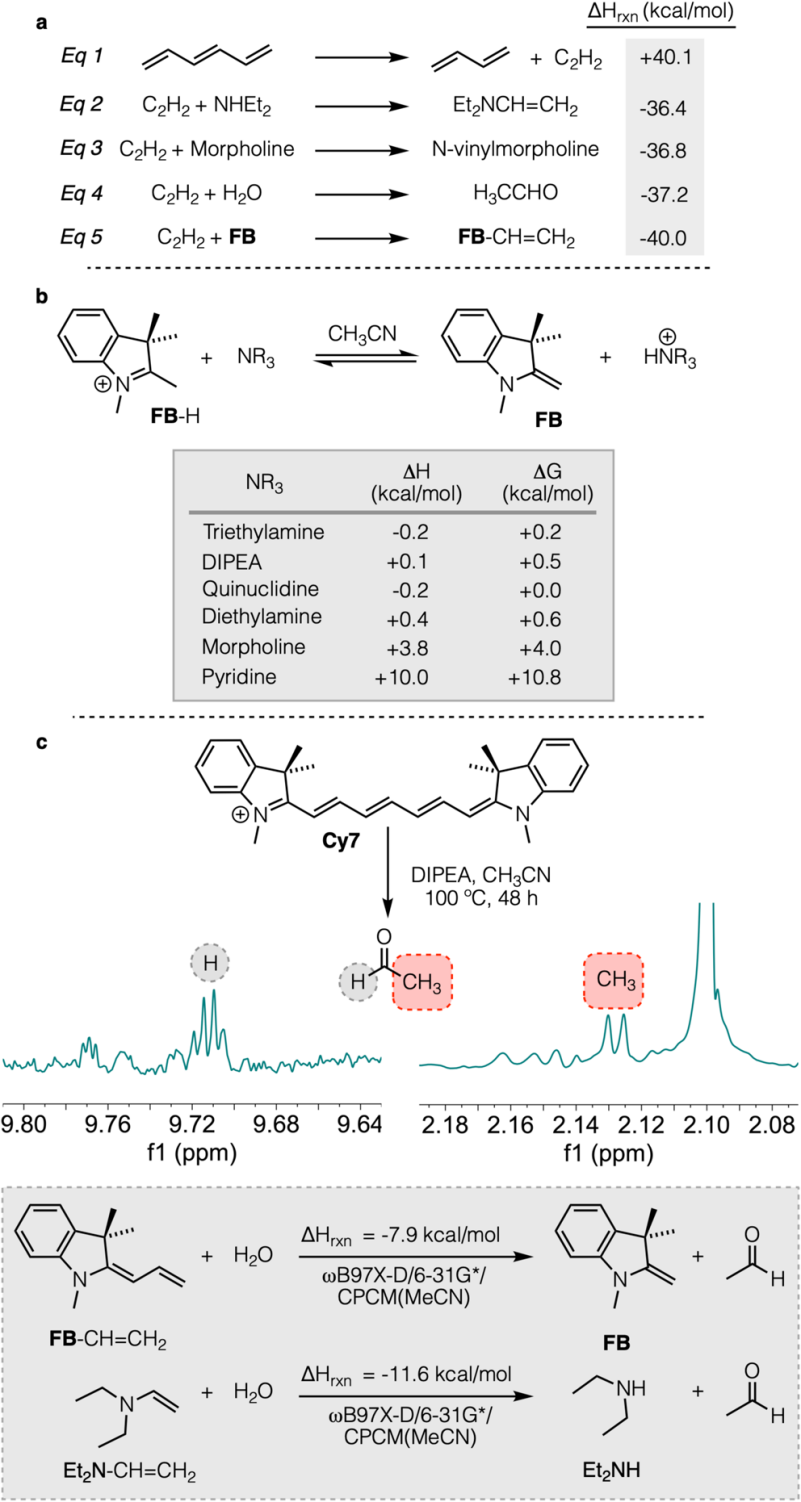
(a) Except as otherwise specified, Δ*H*_rxn_ values shown above are computed from Δ*H*_f_ data in the NIST webbook database.^53^ For diethylamine, the Δ*H*_f_ was computed using the G3(MP2) method;^54^ (b) comparison of calculated relative acidities of **FB** and amines in acetonitrile; (c) NMR spectrum of acetaldehyde in the reaction mixture, and the calculated hydrolysis energy.

Experimentally no C_2_H_2_ was detected in solution or headspace. On simple thermochemical grounds, it is difficult to imagine direct release of C_2_H_2_; such a process should be at least as endothermic (roughly uphill by 40 kcal/mol) as the simple polyene shortening in eq (1). What, then, becomes of this two-carbon fragment? To “pay” the energetic cost of net C_2_H_2_ excision, four candidate energy-compensating reactions may be envisioned (eq 2–5, Figure 4a) (a) net N–H addition across the C_2_H_2_ fragment by the secondary amine to form an N,N-dialkylvinylamine (e.g., eqs 2 and 3); (b) net addition of adventitious water across the acetylene to form acetaldehyde (eq 4); (c) net C–H addition of **FB** across C_2_H_2_ to form **FB**-CH=CH_2_ (eq 5); or (d) net formal trimerization of acetylene to form benzene (exothermic by 143 kcal/mol or ca. 48 kcal/mol per C_2_H_2_ unit) or a phenyl moiety.

The simple mechanism depicted in Figure 2c rationalizes the conversion of **Cy7** to the corresponding aminated (asymmetric) polymethines by secondary amines. Here, the secondary amine attacks the C2’ position of the methine backbone, neutralizing the indolium nitrogen. Proton transfer from the now cationic amine nitrogen to the C1’ carbon enables cleavage of the C1’-C2’ bond, releasing **FB** and the **AsCy6** product. Consistent with this mechanism, as in Figure 2b, diethylamine treatment of C2’-C6’ deuterium-labeled **Cy7** showed full label retention in the corresponding asymmetric product, **AsCy6**(NEt_2_)-D_5_, along with unlabeled **FB**. Evidently, secondary amine attack like that shown in Figure 3c removed the elements of C_2_D_2_, rather than degrading from the other end of the chain. Based on the order of product appearance, growth, and decay as detected by ESI-MS over the course of the reaction, we concluded that **AsCy4**(NEt_2_) and **AsCy2**(NEt_2_) form downstream from **AsCy6**(NEt_2_) via sequential chain-shortening steps, rather than directly forming from **Cy7**. This was confirmed via the explicit reaction of **AsCy6**(NEt_2_) with morpholine, as depicted in Figure 3. Notably, these processes lead to enamine byproducts, both the methylene indoline **FB** and (presumed) *N,N*-diethylvinylamine. This scenario bypasses high energy species such as free acetylene, removing the lost C_2_H_2_ fragment instead as the vinyl group in *N,N*-diethylvinylamine. Although the **FB** is detected, the latter vinylamine is a reactive, low molecular weight species that we were not able to directly detect via ESI-MS. In the presence of even traces of water, this species would be expected to readily hydrate and hydrolyze to liberate acetaldehyde and diethylamine. Indeed, acetaldehyde was detected by ^1^H NMR in these reaction mixtures (Figure 4c).

Though the formation of asymmetric polymethines (**AsCy**s) from end group replacement/chain shortening of **Cy7** by secondary amines appears mechanistically straightforward, as discussed above and shown in Figure 3, the conundrum of truncation promoted by tertiary amines must be examined more closely. To begin the tertiary amine-promoted conversion of **Cy7** to symmetric **Cy5**, amine attack at C2’ or C4’ of the methine chain was considered. However, with no reasonably acidic protons available, further reaction, analogous to that shown in Figure 3c, seemed unlikely. Quantum chemical modeling, including the effects of the CH_3_CN as a solvent, found attack at the C2’ or C4’ positions by trimethylamine (as a model tertiary amine) to be more than 10 kcal/mol endergonic. In all the above tertiary amine-promoted reactions, **TMP** and free **FB** were detected, and as further discussed below, these appear to be the essential players in the tertiary amine-promoted chain shortening reactions. We argue that the tertiary amines mainly serve as bases that work by promoting the proton transfers that isomerize adducts of **FB** with **Cy7** and the shorter cyanines leading to net C_2_H_2_ excision. A key finding (*vide infra*) or this ultimate mechanistic interpretation was the fact that purified **Cy5** showed no reaction (specifically, no chain shortening, release of **FB**, or presence of **TMP**) on treatment (100 °C for 48 h) with the tertiary amines quinuclidine or DIPEA (see Figure 7b).

### Mechanistic Experiments

**Figure 5:**
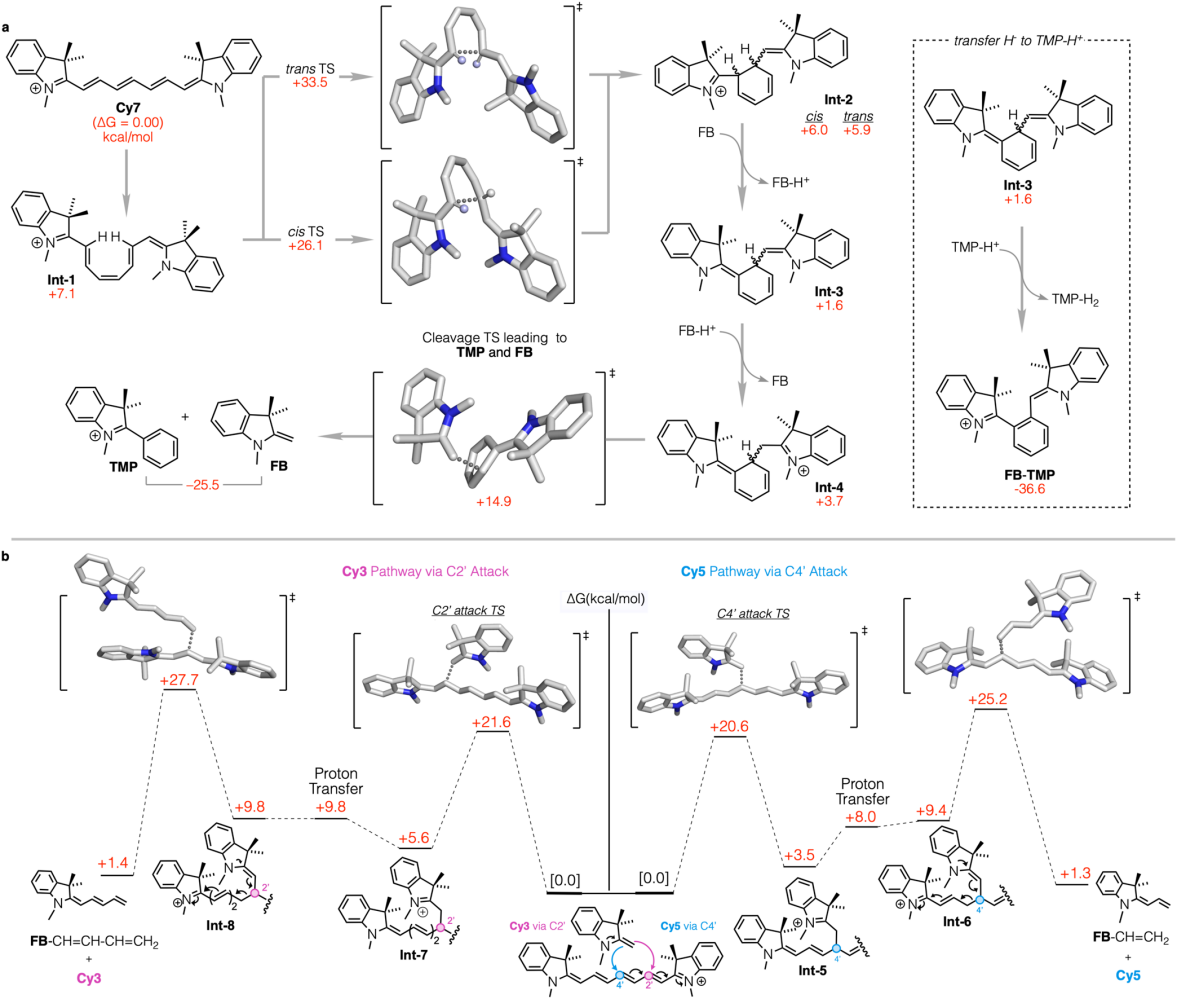
Proposed intramolecular electrocyclization pathway of **Cy7**, resulting in formation of **TMP** and **FB**. Computed TSs are shown for *cis* and *trans* cyclizations from coiled **Int 1** and for the cleavage of **Int 4** to release **FB** and **TMP**. At right is shown the strongly exothermic oxidative aromatization path of **Int 2**, illustrating the strong hydride accepting capacity of **TMP**. The prototropic reaction energetics are computed using **FB** as the proton trafficking base, but as seen in Figure 4b, the relative basicities of **FB** and aliphatic amines are nearly equal. Notably, like tautomerization, proton equilibration in the absence of added base is slow, so that heating of **Cy7** without base or with a weak base (e.g., pyridine) leads to slow emergence of **TMP** and **FB**, which then autocatalytically accelerates the reaction. (b) Free energy diagram of **FB** attack pathways on **Cy7**, leading to **Cy5** (via C4’ attack, right) and **Cy3** (via C2’ attack, left), along with their corresponding **FB**-polyene species. Computed TSs are shown for attack and cleavage steps.

**Figure 6:**
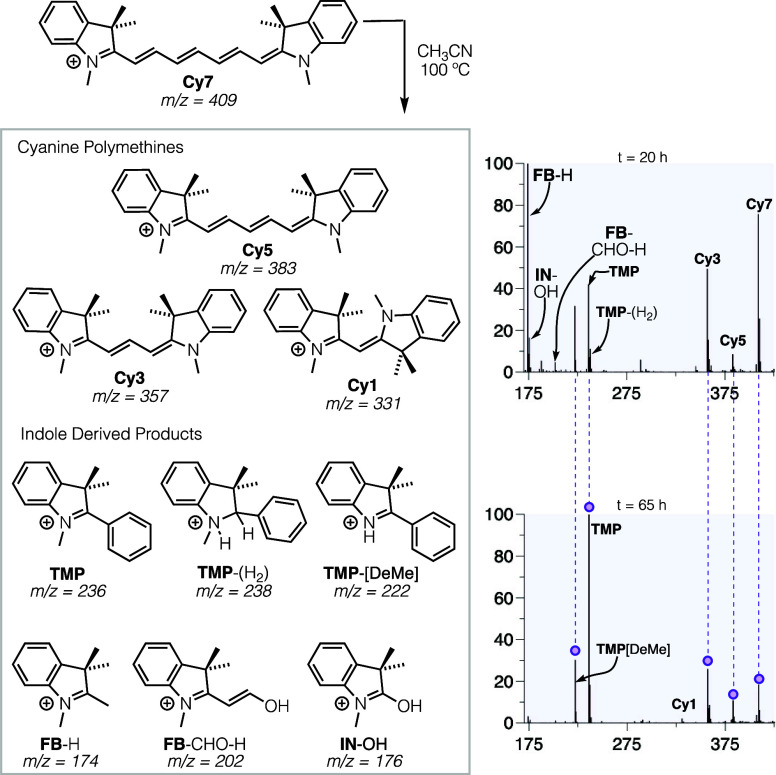
Heating of **Cy7** alone in acetonitrile at 100 °C, showing formation of species **Cy5**, **Cy3**, and **Cy1**, in addition to **TMP**, **FB**, and other indole products previously mentioned at 20 h (top) and 65 h (bottom).

**Figure 7:**
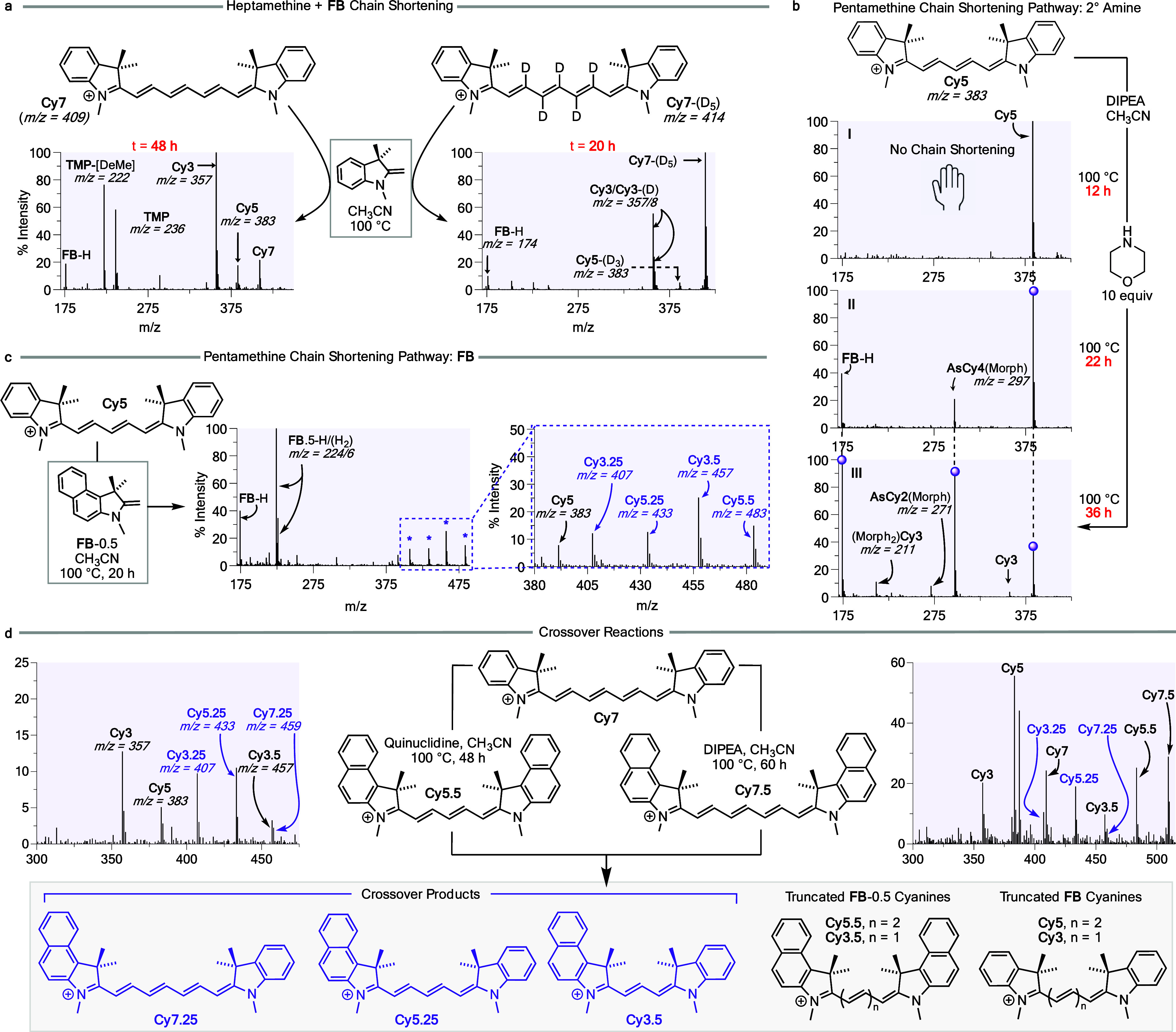
(a) Reaction and comparison of ESI-MS sequences of **Cy7** and **Cy7**-(D_5_) (48 and 20 h) with **FB** in acetonitrile at 100 °C. (b) I ESI-MS sequence after 12 h of **Cy5** and DIPEA before addition of morpholine. II ESI-MS sequence after 22 h after adding morpholine to the mixture. III ESI-MS sequence after 36 h reaction time. (c) Reaction of **Cy5** + **FB**-0.5 (right) and expansion of the scrambled **FB** cyanines (left) observed by ESI-MS. (d) Independent reactions of **Cy7** + **Cy7.5** (with DIPEA, right) and **Cy7** + **Cy5.5** (with quinuclidine, left) lead to the same crossover cyanine products, and truncated **FB**-0.5 and **FB** cyanines.

If the tertiary amines are not directly acting on the **Cy7**, how do the chain-shortening reactions occur? Tertiary amines differ from the reactive secondary amines in lacking an exchangeable proton. A new set of hypotheses was sparked by our observation of free **FB** formed from **Cy7** in the presence of tertiary amines. As the source of the **FB**, an electrocyclization path was suggested by the concomitant appearance of 1,3,3-trimethyl-2-phenylindolinium ion (**TMP**) (Figure 5a) and analogous **TMP**-D_5_ noted above. The exchange of secondary amines and **FB** seen above as cyanine end groups also suggests a direct role for **FB** in chain shortening. Insights supporting this path came from two minimal reactions: (a) **Cy7** heated alone at 100 °C in acetonitrile (Figure 6); (b) **Cy5** treated similarly; these were supplemented by four simple binary reactions: (c) DIPEA + **Cy7** (Figure 3d); (d) DIPEA + **Cy5** (Figure 7b); (e) **FB** + **Cy7** (Figure 7a); and (f) **FB** + **Cy5** (Figure 7c, see Figure S8 for graphical summary).

In reaction (a), heating of **Cy7** alone (Figure 6) forms substantial amounts of **TMP**, **FB**, and chain-shortening products **Cy5** and **Cy3**. In contrast, in reaction (b), heating of the shorter **Cy5** resulted in no change. Even in cases where **Cy5** was subjected to reaction temperatures exceeding 175 °C, no chain shortening was observed. This comparison most starkly illustrates the unique additional reactivity of **Cy7,** whose heptamethine chain enables cyclization, **TMP** formation, and **FB** release, initiating chain shortening processes.

In reaction (c) as recounted previously, DIPEA + **Cy7** forms **TMP** and **FB** (Figure 3d). As in the reagent-free reaction of **Cy7**, the chain shortening to form **Cy5** and **Cy3** is then proposed to occur via attack by the released **FB** on **Cy7** at the C4’ and C2’ positions as shown in Figure 5b. Proton transfers, assisted by the DIPEA (when present) and/or by the **FB** formed when **Cy7** is heated alone, then enable the release of **Cy5** along with **FB**-CH=CH_2_, the vinylated analog of **FB**. Like the vinylation of a secondary amine, this represents a thermochemically reasonable fate for the C_2_H_2_ fragment removed in the net chain shortening.

In case (d), treatment of **Cy5** with DIPEA (10 equiv) at 100 °C (see Figure 7b, top panel) showed essentially no reaction. However, subsequent addition of the secondary amine morpholine (10 equiv) initiated an immediate reaction as expected, forming **AsCy4**(Morph) and (Morph)_2_**Cy3**, and liberating **FB**. Small amounts of **Cy3** were also observed.

Reaction (e), **FB** + **Cy7** combined alone without an amine base (see Figure 7a) led mainly to **Cy3.** The implied byproduct, **FB**-CH=CH–CH=CH_2_, is a polyene even more susceptible to intermolecular reactions than **FB**-CH=CH_2_.^55^ As a control experiment, treatment of **Cy7** with the iodide salt of **FB**-H in the absence of base was explored (Figure 3d); this reagent, lacking the reactive methylene group, led simply to ESI traces analogous to those of the **Cy7** reaction alone, a process that begins by forming the cyclization product **TMP** and releasing **FB.**

In reaction (f), **FB** + **Cy5** slowly produced **Cy3** but interestingly, also generated small amounts of **Cy7**. This latter finding implies release and recapture of **FB**-CH=CH_2_ by **Cy5**, confirming the reversibility of these attacks and exchanges. This is analogous to the N-vinylmorpholine addition shown in Figure 3c. In an extension of this reaction, treatment of **Cy5** with **FB**(0.5) was explored (Figure 7c). Here, the “0.5” designation signifies the presence of benzo[*e*]indolium, IUPAC: 1,1,3-trimethyl-2-methylidenebenzo[*e*]indole. This experiment produced a mixture of crossover products **Cy**(0.5) and **Cy**(0.25) (“0.25” refers to a polymethine with **FB** and **FB**(0.5) end groups) pentamethine and trimethine cyanines (Figure 7c). This points to nucleophilic attack at C2’ of **Cy5** by **FB**, efficiently exchanging end groups, while more slowly effecting chain shortening. These processes are directly analogous to the above-described reactions with secondary amines.

Though the formation of **Cy7** from **Cy5** in experiment (f) implies its intermediacy and reactivity, the vinylated **FB** byproduct was not directly observed. Previous attempts to synthesize and isolate it in monomeric form led to dimer- or oligomerization, as described by Hubschwerlen and co-workers.^55^ Like vinylamines, this diene may be easily protonated and hydrolyzed by trace water, forming acetaldehyde and regenerating **FB**. Quantum chemical modeling finds this hydrolysis to be −7.9 kcal/mol exothermic in acetonitrile (Figure 4c, shaded box). Meanwhile, no byproducts that could be attributed to breakdown of DIPEA (or any of the tertiary amines studied) itself were observed.

Isotopic labeling studies provided further support for the scenario outlined in Figure 5a. As noted earlier, DIPEA + **Cy7**-D_5_ yielded the centrally deuterated pentamethine **Cy5**-D_3_, reflecting removal of the C2’-C3’ (CD)_2_ fragment from the polymethine chain, while the **TMP** product retained all five deuteriums. In addition, the protons at the C1’ and C7’ backbone positions were revealed to be slowly exchangeable by stirring an acetonitrile solution of **Cy7** that included 10 equiv of D_2_O at ambient temperature for 48 h. The resulting C1’/C7’-deuterated-**Cy7** was isolated and characterized by NMR and ESI-MS (Figure S6). This finding presumably reflects the proton exchange expected at the C1’/C7’ sites that occurs during reversible **FB** attack and end group exchange. Importantly, little or no exchange is seen at sites C2’-C6’ under these conditions.

To further explore the reversibility of attack and release of **FB** end groups, we heated a 1:1 mixture of **Cy7** and **Cy5.5** in acetonitrile with quinuclidine (Figure 7d). The resulting cyanine products, **Cy5.25** and **Cy3.25** identified by their *m*/*z* peaks, had exchanged end groups. Additionally, **Cy7** and **Cy7.5** were reacted with DIPEA as described above, and the crossover cyanine products including **Cy7.25** were formed in the reaction. In both experiments, the corresponding **TMP**, **TMP**(0.5), and **FB**(0.5) fragments were also seen.

This observation of end group exchange confirmed that the methylene indoline units such as **FB** readily undergo release and reattachment, consistent with their participation in chain shortening. The chain shortening mechanism presented in Figure 5 above also implies a reasonable pathway for direct truncation of **Cy7** to symmetric trimethine, **Cy3**. The initial part of the mechanism is the same as described for **Cy7** undergoing end group exchange in which **FB** attacks the **Cy7** backbone at the C2’ position. Proton transfer then may occur to the former C1’ position or the former C3’ position; the energetically degenerate protonation at C1’ activates end group exchange, replacing the original **FB** moiety with that of the attacking **FB**. The higher energy protonation at C3’ enables cleavage of the original C2’-C3’ bond to release **Cy3** and (by implication) **FB**-CH=CH–CH=CH_2_ (Figure 5b).

The fate of the excised C_2_H_2_ fragment remains somewhat elusive. ESI-MS peaks corresponding to the proposed vinyl and dienyl **FB** species **FB**-CH=CH_2_ and **FB**-CH=CH–CH=CH_2_ were not directly observed. However, as noted above, the appearance of small amounts of chain *lengthening* product **Cy7**, formed during **FB** treatment of **Cy5**, implies the presence and participation as a nucleophile of **FB**-CH=CH_2_. Analogous C_2_H_2_ transfers were evidenced in the reactions of morpholine and **AsCy6**(NEt_2_) (see Figure 3c). Such species are highly reactive, and may undergo further chemistry, either in the form of self-condensation or by reaction with adventitious water.^55^ Despite our best efforts to ensure dryness, hydrolysis is clearly possible in light of the observation of other oxygen-containing fragments, most notably 1,3,3-trimethyl-2-indolone (depicted below as **IN**–OH, the protonated M+H adduct detected by ESI-MS), the lactam corresponding to **FB**.

### Quantum Chemical Modeling

To develop a more complete picture of the reaction paths involved in the thermal “blueing” of the **Cy7** cyanine dye, quantum chemical simulations were run using the ωB97X-D density functional,^56^ the 6-31G* basis set,^57−59^ and the conductor-like polarizable continuum model (CPCM) to account for the acetonitrile solvent;^60^ we designate this overall model ωB97X-D/6-31G*/CPCM(MeCN). These calculations were performed with the Spartan program.^61^ Candidate structure lists for each species were initially generated using Spartan’s molecular mechanics conformation generator. Reoptimization and energy ordering of the lists (typically tens to hundreds of structures) were performed with the semiempirical PM6 method. After removal of duplicate structures, single-point ωB97X-D/6-31G*/CPCM(MeCN) energies were computed for structures within the lowest 5 kcal/mol energy window, and the lowest ten of these were then fully optimized at this level, including vibrational analysis. For transition structures (TSs), intrinsic reaction coordinate (IRC) calculations were used to verify their connections to the respective starting materials and products. The resulting free energy changes for the computed chain shortening events are summarized in Figure 5a,b. Additional relevant computed reaction energetics are shown in Figure 4.

Figure 5a depicts the ring closure of **Cy7**, whose ground state is the extended linear structure shown. Regarding the *trans*-*cis* isomerizations required to coil the polyene to enable the ring closure leading ultimately to **TMP**, multiple TSs and energy barriers were computed, with none exceeding 14 kcal/mol. Given the well-established experimental conformational mobility of **Cy7**, synthesized itself from a cyclic building block via the Zincke reaction,^34^ we omit here a detailed exploration of the many minima en route from the linear to the coiled conformations of **Cy7** that set the stage for the electrocyclization. Importantly, however, the requisite coiled forms are calculated to be close in energy to the extended global minimum indicating that such geometries are thermally accessible at 100 °C among the equilibrating conformations of **Cy7**.

Figure 5b shows the sequence of steps by which free **FB** is able to reversibly attack **Cy7** and achieve chain shortening. Transition state calculations for these attacks at C2’/C6’ and at C4’ of **Cy7** find free energy barriers of 21.6 and 20.6 kcal/mol, respectively, suggesting little selectivity between these two paths. Similarly, the respective adducts are close in energy. Nonetheless, the former path has twice the site degeneracy of the latter, suggesting that the C2’ attack should predominate. In fact, relatively rapid end group exchange without chain shortening is experimentally observed for both **Cy7** and **Cy5**, as depicted in Figure 7. The uphill proton transfer to form the intermediate that liberates **FB**-CH=CHCH=CH_2_ translates into easier exchange (degenerate) of **FB** end groups than release of the chain shortened **Cy3**. Similar logic applies to the attack at C4’ in that reversibility of the **FB** attack is easier than proton transfer and cleavage to release **FB**-CH=CH_2_. Likewise, in **Cy5**, **FB** attack shows a barrier of 22.4 kcal/mol, but a relatively higher barrier for chain shortening than for simple end group release. Interestingly, the barrier to **FB** addition to **Cy3** is even higher, at 26.2 kcal/mol.

That cyclization of **Cy7** occurs to form **TMP** and **FB** is experimentally clear, as is the chain-shortening action of free **FB** on **Cy7** and **Cy5**. Though the proposed vinylated and dienylated **FB** byproducts are not directly observed, their presence is implied by the appearance of chain-extended products, as noted above. Acetaldehyde was also detected by NMR in a **Cy7** + DIPEA reaction mixture (Figures 4c and S7). Trace water is hard to completely eliminate, especially in the handling of the ionic cyanine dyes, the highly polar acetonitrile solvent, and the hygroscopic amines used. We therefore propose that the initially formed alkenylated **FB** derivatives undergo hydrolysis, either in situ or in postreaction handling, to release acetaldehyde (or potentially acrolein) which would not be easily seen in ESI-MS. Calculations indicate that, like vinylamine, this reaction is substantially exothermic, supporting the proposed hydrolytic modes of breakdown shown in Figure 4c.

### Reactivity and Structure of TMP

To explore the possible chemical behaviors of the phenyl indolium species **TMP**, it was independently synthesized and its reactions with amines and **FB** were examined (Figure S5). The computational results indicate that **TMP** is a potent oxidant, capable of hydride abstraction from alkyl amines such as triethylamine (−7.8 kcal/mol). This reactivity is easily rationalized in terms of the phenyl ring’s steeply twisted orientation to the indoline, enforced by three flanking methyl groups (for the X-ray crystal structure of **TMP** see Supporting Information). This twist (46°) limits effective resonance stabilization of its attached cationic C=N^+^ moiety. The reduction product **TMP**-H (detected by ESI-MS as **TMP**-H_2_^+^, *m*/*z* = 238), seen in reactions that generate **TMP**, implies formation of an oxidized byproduct. The calculated reaction free energy for hydride transfer from the deprotonated species (**Int-3** in Figure 5a) formed after **Cy7** cyclization is −38.2 kcal/mol, as expected for an aromatizing reaction. Similarly, hydride transfer from the **FB-Cy3** adduct is calculated to release 20.4 kcal/mol. In reactions involving **Cy7**, **FB**, and **TMP**, the expected ions resulting from these processes, *m*/*z* = 407 and *m*/*z* = 528 respectively, do appear in conjunction with the **TMP**-H formation. Another reaction of **TMP** is demethylation of the N–CH_3_ group by amines and by **FB**, as verified via study of the N-CD_3_ and N–^13^CH_3_ isotopomers (Figure S5). We denote the resulting 2-phenylindolenine (*m*/*z* = 222) **TMP**-[DeMe] (*DeMethylated*). The analogous **TMP**-0.5-[DeMe] was likewise noted in reactions of the parent heptamethine **Cy7.5** via HRMS. Substantial further rearrangement and reaction chemistry follows from **TMP** formation but untangling the details of the resulting convoluted reaction manifold is deferred to a later publication.

## Conclusions & Outlook

**Figure 8:**
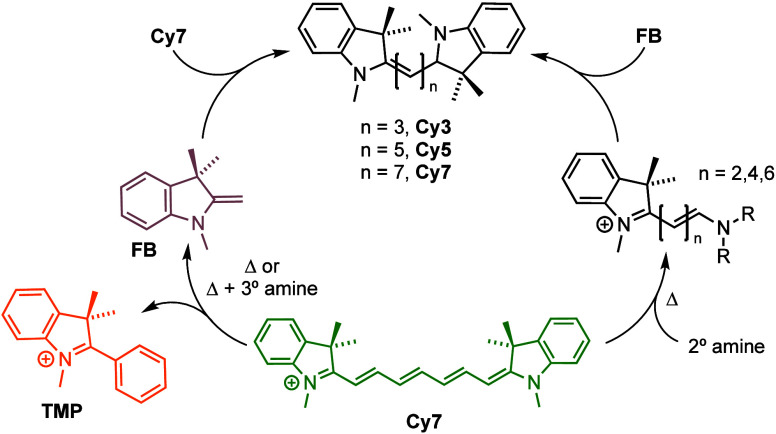
Heating of **Cy7** alone in acetonitrile at 100 °C forms **TMP** and its byproduct **FB**, which enables shortening of the **Cy7** polymethine chain to form species **Cy5**, **Cy3**, and **Cy1**. Analogous reactions with secondary amines form asymmetric polymethines as well as the shortened **Cy** species.

This work has presented several key findings regarding the chemistry of cyanine dyes when heated in acetonitrile (see Figure 8 for a pictorial summary):

- A new mechanism is described for initiation of thermal cyanine “blueing” in the absence of reagents, oxygen, or light. This process is unique to **Cy7**, in which the polyene chromophore undergoes 6-membered ring cyclization and aromatization, releasing Fischer’s base (**FB**) and the 1,3,3-trimethyl-2-phenylindolium (**TMP**) ion.
- The carbon nucleophile **FB** attacks **Cy7**, **Cy5**, and **Cy3** at C2’ or C4’ positions. Degenerate proton shuffling in the resulting intermediates enables rapid replacement of their indoline end groups with the attacking **FB** group.
- Proton exchange in the above **FB** adducts may also form higher energy species, enabling chain-shortening to form **Cy5**, **Cy3**, and **Cy1.** These modes of reaction are verified by explicit treatment of **Cy7** with **FB**, from which the same suite of products quickly arise. Release of vinylated and dienylated **FB** end groups account for the 2- or 4-carbon fragments excised from the polymethine chains.
- In contrast to **Cy7**, **Cy5** is unreactive in isolation, but does react with added **FB** or secondary amines to undergo end group exchange and chain shortening analogous to that seen with **Cy7**.
- Secondary amines mimic the **FB** reactivity described above, nucleophilically attacking **Cy7** at C2’ or C4’ sites, exchanging protons, and reversibly displacing the **FB** or amine end groups to form asymmetric cyanines of type **AsCy6**(NR_2_) and further, to form (R_2_N)_2_**Cy** species. As with **FB**, proton shuffling within the amine adducts enables facile exchange of the amine end groups. Analogous chain shortening also occurs, implying release of R_2_NCH=CH_2_ enamines or dienamines R_2_NCH=CHCH=CH_2_. Like the parent cyanine dyes, the isolable **AsCy** products’ spectral properties span across the visible range, allowing for applications in multicolor imaging.
- Cyanines undergo ready end group exchange, equilibrating **FB** and secondary amines. Displacement of an aniline by **FB** is in fact intrinsic to the Zincke and classic related syntheses of the cyanines. Thus, in **FB**, itself an enamine, the *exo*-methylene site reacts much like an amine. This similarity is supported by theoretical calculations that find very similar acidities for the protonated forms of **FB** and aliphatic amine bases in acetonitrile.
- The alkenylated end groups such as **FB**-CH=CH_2_ and R_2_N–CH=CH_2_, invoked as the carriers of the C_2_H_2_ fragments lost in chain shortening, have evaded direct observation. However, their presence is confirmed by the observation of small amounts of chain *extended* cyanines, for instance in reaction of **FB** with **Cy5**, and of morpholine with **Cy7**. Literature efforts to isolate and directly observe **FB**-CH=CH_2_ noted failures due to dimerization and polymerization. In the presence of water, these enamines are subject to exothermic hydrolysis, which readily generates acetaldehyde, and indeed, small amounts of acetaldehyde can be detected in reaction mixtures.
- Secondary amines such as morpholine react with **Cy7** and **Cy5** to form **AsCy** chromophores even at room temperature. This reactivity offers potential for *in situ* adjustment of the chromophores’ spectral coverage, potentially via both chain length and amine π donor ability variations.
- The role of tertiary amines in the present studies appears to be limited to assisting in the proton shuffling involved in the cyclization of **Cy7** and the end group exchange and chain shortening by **FB** or secondary amines. Their steric preferences in acid–base reactions may affect the selectivity in conversion of **Cy7** to **Cy5** and **Cy3**, as seen in comparing the reactivity of DIPEA (favoring **Cy5**) and quinuclidine (favoring **Cy3**). However, no products or intermediates incorporating tertiary amine or their fragments were noted, though as supported by the calculations, it is clear that they can reduce **TMP** to **TMP**-H_2_.
- Lastly, we believe that the new understanding of the cyclization behavior of longer cyanine dyes such as **Cy7**, and the broader reactivity of this family of dyes with amine nucleophiles will help in interpreting and exploiting the rich chemistry involved in the in situ reactions of cyanine chromophores.

## Supporting Information Available

Additional experimental details, supplimentary figures, X-ray data, and coordinates and energies from quantum chemical studies (PDF)
