# Supplementary material for: Mechanistic Insight into the Thermal “Blueing” of Cyanine Dyes
Source: J Am Chem Soc. 2024 Jul 11;146(29):19756–67. doi: 10.1021/jacs.4c02171 (PMC11273608; doi:10.1021/jacs.4c02171)
Supplement: Supplementary file 1 — ja4c02171_si_001.pdf [file ja4c02171_si_001.pdf]

Supporting Information

Mechanistic Insight into the Thermal "Blueing" of Cyanine Dyes

Aria Vahdani, Mehdi Moemeni, Daniel Holmes, Richard R. Lunt, James E. Jackson,* Babak Borhan*

Michigan State University, Department of Chemistry, East Lansing, MI 48824.

Michigan State University, Department of Chemical Engineering, East Lansing, MI 48824.

Jackson@chemistry.msu.edu, Babak@chemistry.msu.edu

I. GENERAL REMARKS 3
II. GENERAL EXPERIMENTAL REMARKS 4
III. SYNTHESIS AND EXPERIMENTAL PROCEDURES 5
Synthesis of Zincke Pyridinium Salts *5*
IV. SYNTHESIS OF HEPTAMETHINE CYANINE DYES 8
Synthesis of Cyanine Dyes from Zincke Salts[1] *8*
Synthesis of Cy7.5 via Glutacondianil Condensation *10*
Synthesis of TMP and Derivatives *11*
V. REACTIONS OF HEPTAMETHINES AND 3° AMINES 13
VI. REACTIONS OF NUCLEOPHILES WITH HEPTAMETHINE CYANINES 19
VII. REACTION OF IR-786 AND FB 25
Isolation of 3'-Br-AsCy6(NEt_2) from 3'-Br-Cy7 + $NHEt_2$ *26*
VIII. SUPPLEMENTARY FIGURES 27
IX. NMR SPECTRA 35
X. QUANTUM CHEMICAL CALCULATION RESULTS 93
Section 1: Building blocks *96*
Section 2: Cy7 cyclization to form TMP and FB *110*
Section 3: FB+Cy7 reaction at C2' *128*
Section 4: FB+Cy7 reaction at C4' *142*
Section 5: FB+Cy5 reaction at C2' *156*
Section 6: FB+Cy3 reaction at C2' *166*
XI. X-RAY CRYSTAL STRUCTURE OF TMP 174
XII. REFERENCES 181

I. General Remarks

Solvents and reagents were of the highest available purity, used as purchased, or they were purified/dried using standard methods. Synthetic procedures were performed under an atmosphere of nitrogen unless stated otherwise. NMR spectra were obtained using either a 500 MHz or 600 MHz, Varian Inova NMR spectrometer and referenced using the residual ^{1}H peak from the deuterated solvent. Column chromatography was performed using Silicycle 60Å 35-75 μm irregular silica gel. Pre-coated 0.25 mm thick silica gel Analtech 60 F254 plates were used for analytical TLC and visualized using UV light (short wave-254 nm). UV-Vis spectra were obtained on an Agilent Cary 100 series instrument with matched 1.0 cm quartz cuvettes. Fluorescence was measured on an Fluorolog by ISA automated luminescence spectrometer in 1.0 cm quartz cuvettes at 26 ± 1°C. Reactions were monitored via ESI-MS using a Waters Micromass ZQ instrument, with a mobile phase of acetonitrile containing 0.01% trifluoroacetic acid. High Resolution Mass Spectrometry using electrospray in positive ion mode was performed on a Q-TOF Ultima system in the Department of Chemistry at Michigan State University Mass Facility. ESI-MS and spectral data analysis were performed using GraphPad Prism 10. Fluorescence quantum yield measurements were performed on a Quantarus (C11347-11) QY Absolute PL quantum yield spectrometer.

II. General Experimental Remarks

For reaction monitoring via ESI(+), aliquots from crude reaction mixtures at given time points were obtained via by first cooling the reaction tube to room temperature, replacing its threaded cap with a septum maintaining a dynamic flow of argon, and then removing ~20 μL via an oven dried gas tight Hamilton© 100 μL syringe. Exposure to atmospheric moisture was avoided by rapidly replacing the septum with the threaded cap reseal the tube prior to re-heating.

For reactions yielding multiple co-eluting species with negligible differences in polarity, the chromatography column fractions that contained one or more desired products were collected and further separated using a longer, smaller diameter column with a specified gradient for optimal separation and yield. The resulting isolated samples were used to calculate yields (see individual products for specific characterization remarks).

*Generally, purifications entailed dissolving of the crude material in a minimal quantity of DCM, and its transfer to a silica gel column, which was packed under N_2 pressure from a suspension in DCM.

III. Synthesis and Experimental Procedures

Synthesis of Zincke Pyridinium Salts

Synthesis of 2,4-Dinitrophenyl p-Toluenesulfonate (S1)[1]

To a solution of 2,4-dinitrophenol (1 equiv) in distilled dichloromethane (0.06 M 2,4-dinitrophenol) was added p-toluenesulfonyl chloride (1.1 equiv), followed by distilled triethylamine (2.5 equiv). The reaction was stirred at room temperature for 16 h, after which water was added (15 mL/g dinitrophenol) and the mixture transferred to a separatory funnel, where the aqueous layer was extracted with CH_2Cl_2 (2 x 5 mL/g dinitrophenol). The combined organic phase was subsequently washed with a saturated solution of $NaHCO_3$ (10 mL/g dinitrophenol), followed by a saturated solution of NaCl (10 mL/g dinitrophenol), before drying over anhydrous Na_2SO_4. The solvent (dichloromethane) was then evaporated under reduced pressure, yielding a crude yellow residue. Purification was performed via serial trituration (addition and decanting of hot methanol in three portions of 10 mL/g of dinitrophenol) removing most of the dark yellow coloration. The resultant pale yellow-white solid was further dried, giving **S1**, which was used in the next step without further purification. **^{1}H-NMR** (500 MHz, dmso-*d*$_6$) δ (ppm) 8.83 (d, J = 2.8 Hz, 1H), 8.58 (dd, J = 9.1, 2.8 Hz, 1H), 7.81 – 7.75 (m, 2H), 7.58 (d, J = 9.0 Hz, 1H), 7.53 (d, J = 8.1 Hz, 2H), 2.45 (s, 3H). **^{13}C-NMR** (126 MHz, dmso-*d*$_6$) δ (ppm) 147.31, 145.66, 144.31, 142.58, 130.74, 129.98, 129.68, 129.52, 128.52, 128.06, 126.10, 125.51, 121.95, 119.59, 21.32, 20.81.

Synthesis of Zincke Salts (S2-S4) from S1[1]

General Procedure

To a solution of **S1** (1.1 equiv) in distilled toluene (7 mL/mmol) was added the corresponding pyridine derivative (1 equiv). After the mixture was refluxed for 16 h and cooled to room temperature, solids which precipitated were separated by vacuum filtration and subsequently washed with toluene (2 × 5 mL/mmol **S1**) and Et_2O (2× 5 mL/mmol **S1**) before drying further under reduced pressure. The resultant Zincke salt products were used in the next step without further purification.

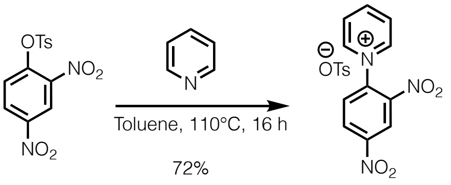

S2: *1-(2,4-dinitrophenyl)pyridin-1-ium 4-methylbenzenesulfonate*

The title compound was synthesized according to a procedure previously reported[1] and isolated as a white powder (2.7 g, 1.11 mmol, 72%). **^{1}H-NMR** (500 MHz, dmso-*d6*): δ (ppm) 9.42-9.35 (m, 2H), 9.12 (d, J = 2.5 Hz, 1H), 8.99-8.92 (m, 2H), 8.48-8.37 (m, 3H), 7.46 (d, J = 8.1 Hz, 2H), 7.10 (d, J = 7.8 Hz, 2H), 2.28 (s, 3H). **^{13}C-NMR** (126 MHz, dmso-*d6*)): δ (ppm) 149.1, 148.8, 146.1, 145.8, 143.0, 138.7, 137.5, 131.9, 130.2, 128.0, 125.5, 121.4, 20.8. **HRMS (ESI+):** calcd. for $C_{11}H_8N_3O_4^+$ [M-TsO$^-$]: 246.0509; found 246.0557.

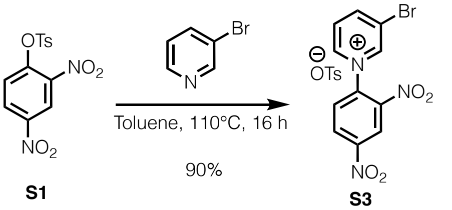

S3: *3-bromo-1-(2,4-dinitrophenyl)pyridin-1-ium 4-methylbenzenesulfonate*

The title compound was synthesized according to a previously reported procedure[1] and isolated as a beige-white powder (1.58 g, 3.18 mmol, 90%). **^{1}H-NMR** (500 MHz, dmso-*d6*): δ (ppm) 9.85 (d, J = 1.7 Hz, 1H), 9.44 (dt, J = 6.1, 1.2 Hz, 1H), 9.30-9.19 (m, 1H), 9.10 (d, J = 2.5 Hz, 1H), 8.97 (dd, J = 8.7, 2.5 Hz, 1H), 8.40 (dd, J = 8.6, 2.3 Hz, 2H), 7.43 (d, J = 8.1 Hz, 2H), 7.10 (d, J = 7.8 Hz, 2H), 2.28 (3H). **^{13}C-NMR** (126 MHz, dmso-*d6*): δ (ppm) 151.10, 149.26, 147.27, 145.70, 142.80, 137.89, 137.60, 130.24, 128.64, 128.05, 125.46, 121.83, 121.36, 20.80. **HRMS (ESI+):** calcd. for $C_{11}H_7BrN_3O_4^+$ [M-TsO$^-$]: 323.9614; found 323.9677.

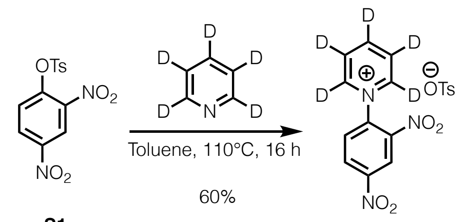

S1 **S4**

S4: *1-(2,4-dinitrophenyl)pyridin-1-ium-2,3,4,5,6-d_5 4-methylbenzenesulfonate*

The title compound was prepared according to a previous report[1] using pyridine-d_5; **S4** was isolated as a white solid (2.1 g, 4.9 mmol, 60%). ^{1}**H-NMR** (500 MHz, dmso-d_6): δ (ppm) 9.12 (d, J = 2.4 Hz, 1H), 8.97 (dd, J = 8.7, 2.4 Hz, 2H) 8.42 (d, J = 8.7 Hz, 1H), 7.45 (d, J = 8.1 Hz, 2H), 7.10 (d, J = 7.5 Hz, 2H), 2.28 (s, 3H). ^{13}**C-NMR** (126 MHz, dmso-d_6): δ (ppm) 149.6, 146.3, 143.5, 139.1, 137.9, 132.3, 130.7, 128.5, 126, 121.9, 21.2. **HRMS (ESI+):** calcd. for $C_{11}H_3D_5N_3O_4$ [M-TsO$^-$]: 251.0823; found 251.0860.

IV. Synthesis of Heptamethine Cyanine Dyes

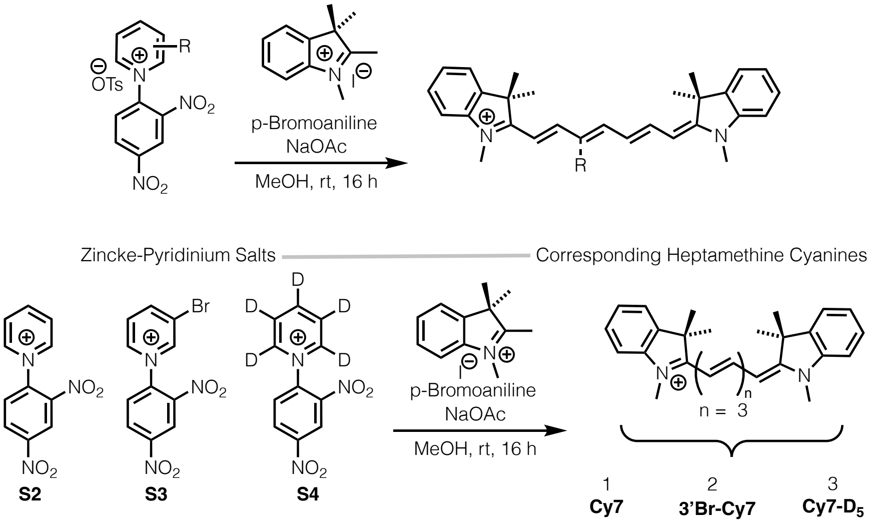

Synthesis of Cyanine Dyes from Zincke Salts[1]

General Procedure

To a round bottom flask containing a solution of Zincke salt (1 equiv) in methanol (7 mL/mmol Zincke salt), was added 4-bromoaniline (1.2 equiv), and the mixture was stirred at room temperature for 30 min. 1,2,3,3-tetramethyl-3H-indolium iodide (3 equiv) and NaOAc (6 equiv) were added at once. The resultant mixture was stirred for an additional 16 h at room temperature, after which diethyl ether (21 mL/mmol Zincke salt) was added, and the mixture was cooled in a freezer (-15 ℃) for 3 h. The resulting precipitate was collected by vacuum filtration, rinsed with water (2 x 10 mL/mmol Zincke salt) followed by diethyl ether (10 mL/mmol Zincke salt), and dried under reduced pressure for several hours. The crude residue was then purified by flash column chromatography [CH_2Cl_2; then 95:5 CH_2Cl_2/MeOH] to furnish the corresponding cyanines.

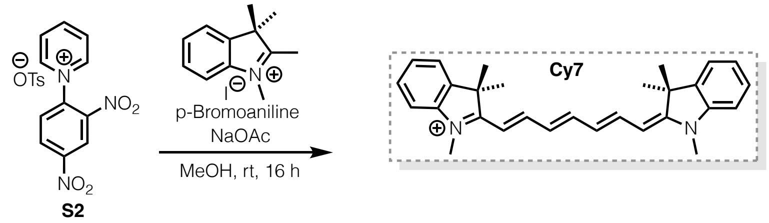

Cy7: *1,3,3,-trimethyl-2-((1E,3E,5E)-7-((E)-1,3,3-trimethylindolin-2-ylidene)hepta-1,3,5-trien-1-yl)-3H-indol-1-ium iodide*

The title compound **Cy7** was prepared as reported in a previous procedure,[1] and isolated as a metallic-green solid (550 mg, 0.29 mmol, 85%).^{1}**H-NMR** (500 MHz, CD_3OD-d_4): δ (ppm) 7.94 (t, *J* = 13.1 Hz, 2H), 7.62 (t, *J* = 12.8 Hz, 1H), 7.47 (dd, *J* = 7.3, 1.2 Hz, 2H), 7.40 (td, *J* = 7.7, 1.2 Hz, 2H), 7.30-7.20 (m, 2H), 6.56 (t, *J* = 12.6 Hz, 2H), 6.27 (d, *J* = 13.6 Hz, 2H), 3.60 (s, 6H), 1.69 (s, 12H). ^{13}**C-NMR** (126 MHz, CD_3OD-d_4): δ (ppm) 173.77, 157.71, 152.93, 144.41, 142.34, 129.72, 126.95, 125.98, 123.26, 111.61, 104.79, 50.24, 31.45, 27.89. **HRMS (ESI+):** calcd. For $C_{29}H_{33}N_2^+$ [M-I$^-$]: 409.2638; found 409.2660.

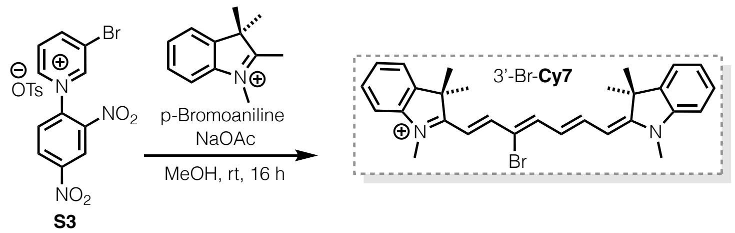

3'Br-**Cy7**: *2-((1 E,3Z,5E)-3-bromo-7-((E)-1,3,3-trimethylindolin-2-ylidene)hepta-1,3,5-trien-1-yl)-1,3,3-trimethyl-3H-indol-1-ium*

The title compound was prepared according to a previously reported procedure,[1] and isolated as a metallic-red solid (443 mg, 0.72 mmol, 74%) ^{1}**H-NMR** (500 MHz, CD_3OD-d_4): δ (ppm) 8.16 (dd, *J* = 14.2, 12.1 Hz, 1H), 7.95 (d, *J* = 13.10 Hz, 1H), 7.85 (d, *J* = 12.6 Hz, 1H), 7.56 (dt, *J* = 7.4, 1.0 Hz, 1H), 7.50-7.43 (m, 3H), 7.41-7.34 (m, 2H), 7.27-7.20 (m, 2H), 6.85 (t, *J* = 12.3 Hz, 1H), 6.60 (d, *J* = 14.2 Hz, 1H), 6.23 (d, *J* = 13.0 Hz, 1H), 3.75 (s, 3H), 3.59

(s, 3H), 1.74 (s, 6H), 1.72 (s, 6H). ^{13}**C-NMR** (126 MHz, CD_3OD-d_4): δ (ppm) 177.33, 173.28, 155.16, 153.74, 146.93, 144.46, 143.94, 143.13, 142.03, 129.96, 129.68, 127.59, 125.73, 123.47, 123.26, 116.34, 112.98, 111.34, 108.08, 101.56, 51.31, 50.06, 32.30, 31.30, 27.88, 27.54. **HRMS (ESI+):** calcd. for $C_{29}H_{32}BrN_2^+$ [M-I$^-$]: 487.1743; found 489.1747.

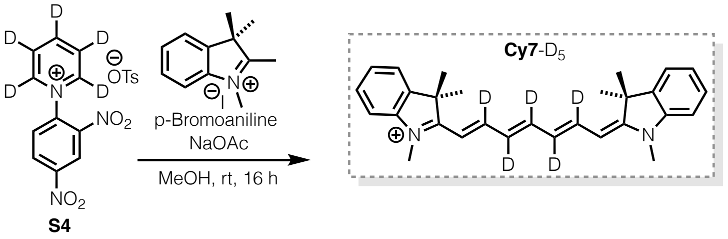

Cy7-D_5: 1*,3,3,-trimethyl-2-((1E,3E,5E)-7-((E)-1,3,3-trimethylindolin-2-ylidene)hepta-1,3,5-trien-1-yl-2,3,4,5,6-*d_5*)-3H-indol-1-ium iodide*

The title compound was prepared according to a previously reported procedure,[1] and isolated as a metallic-green solid (1.02 g, 0.6 mmol, 80%). ^{1}**H-NMR** (500 MHz, CD_3OD-d_4): δ (ppm) 7.47 (dd, *J* = 7.5, 1.2 Hz, 2H), 7.42-7.38 (m, 2H), 7.28-7.22 (m, 4H), 6.26 (bs, 2H), 3.59 (s, 6H), 1.69 (s, 12H). ^{13}**C-NMR** (126 MHz, CD_3OD-d_4): δ (ppm) 144.41, 142.32, 140.90, 129.71, 125.96, 123.25, 111.59, 48.80, 31.41, 27.88. **HRMS (ESI+):** calcd. for $C_{29}H_{28}D_5N_2^+$ [M-I$^-$]: 414.2952; found 414.2967.

Synthesis of Cy7.5 via Glutacondianil Condensation

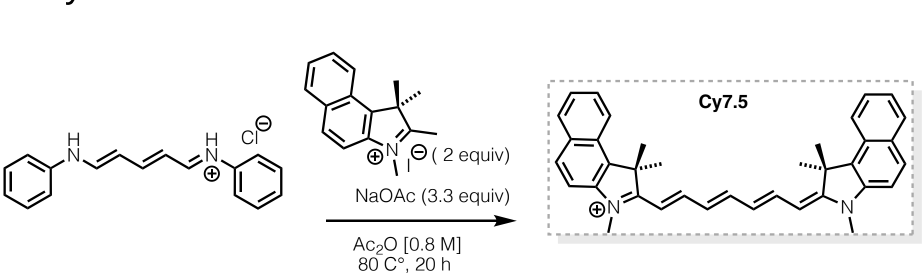

Cy7.5: 1,1,3-trimethyl-2-((1E,3E,5E,7E)-7-(1,1,3-trimethyl-1,3-dihydro-2H-benzo[e]indol-2-ylidene)hepta-1,3,5-trien-1-yl)-1H-benzo[e]indol-3-ium iodide

The title compound was prepared according to an adaptation of a previously published procedure;[2] glutacondianil hydrochloride (1 equiv, 0.35 mmol), and 1,1,2,3-tetramethyl-1H-benzo[e]indol-3-ium iodide (2 equiv, 0.7 mmol). The product was isolated via normal phase flash column chromatography [$CHCl_3$ to 90:10 $CHCl_3$/MeOH] yielding a dark-green solid (96 mg, 0.15 mmol, 43%). **^{1}H-NMR** (500 MHz, CD_3OD-d_4): δ (ppm) 8.23 (d, *J* = 8.6 Hz, 2H), 8.00 (m, 6H), 7.65 – 7.57 (m, 5H), 7.47 (t, *J* = 7.5 Hz, 2H), 6.59 (t, *J* = 12.7 Hz, 2H), 6.33 (d, *J* = 13.5 Hz, 2H), 3.72 (s, 6H), 1.99 (s, 12H). **^{13}C-NMR** (126 MHz, CD_3OD-d_4): δ (ppm) 141.83, 133.37, 131.66, 131.12, 129.46, 128.70, 125.93, 123.32, 111.86, 52.11, 31.79, 27.49. **HRMS (ESI+):** calcd. for $C_{37}H_{37}N_2^+$ [M-I$^-$]: 509.2951; found 509.2959.

Synthesis of TMP and Derivatives

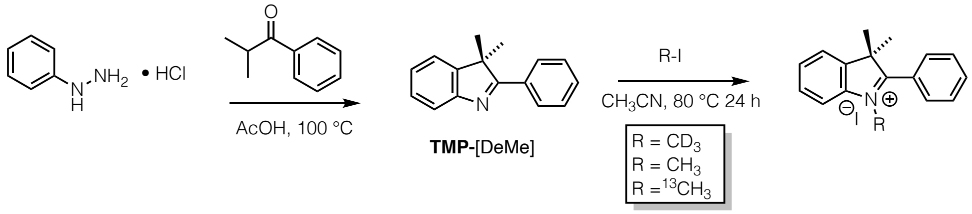

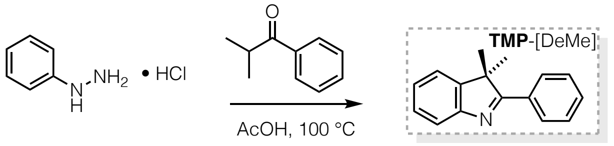

TMP-[DeMe]: *3,3-dimethyl-2-phenyl-3H-indole*

The above compound was prepared according to a previously published procedure[3] as a clear oil (1.78 g, 8.02 mmol, 80%). **^{1}H-NMR** (500 MHz, Chloroform-*d*) δ (ppm) 8.19-8.11 (m, 2H), 7.70 (dt, *J* = 7.6, 0.8 Hz, 1H), 7.54-7.45 (m, 3H), 7.40-7.32 (m, 2H), 7.31-7.24 (m, 1H), 1.60 (s, 6H). **^{13}C-NMR** (126 MHz, Chloroform-*d*) δ (ppm) 183.40, 153.22, 147.72, 133.45, 130.66, 128.73, 128.43, 127.88, 125.98, 121.06, 121.03, 121.02, 77.41, 77.36, 77.16, 76.91, 53.73, 53.71, 24.90, 24.88. **HRMS (ESI+):** calcd. for $C_{16}H_{16}N$ [M$^+$]: 222.1277 found 222.1292.

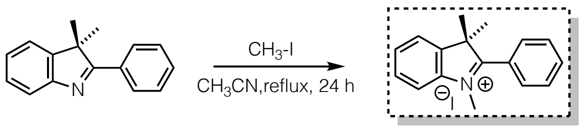

TMP: *1,3,3-trimethyl-2-phenyl-3H-indol-1-ium iodide*

The above compound was prepared according to a previously published procedure.[3] The product was isolated as a yellow solid, (yield 1.53 g, 4.22 mmol, 93%). **^{1}H-NMR** (500 MHz, Chloroform-*d*) *δ* (ppm) 8.19-8.13 (m, 2H), 7.70 (dt, *J* = 7.5, 0.9 Hz, 1H), 7.52 – 7.46 (m, 3H), 7.40 – 7.33 (m, 2H), 7.29 (dd, *J* = 7.3, 1.0 Hz, 1H), 1.60 (s, 6H). **^{13}C-NMR** (126 MHz, Chloroform-*d*) *δ* (ppm) 191.13, 141.90, 141.67, 133.12, 130.89, 129.73, 128.63, 125.15, 123.07, 116.82, 77.41, 77.37, 77.16, 76.91, 55.73, 38.58, 22.78. **HRMS (ESI+):** calcd. for $C_{17}H_{18}N^{+}$ [M-I^{-}]: 236.1434; found 236.1443.

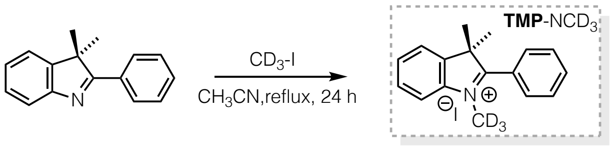

TMP-NCD$_3$: *3,3-dimethyl-1-(methyl-d$_3$)-2-phenyl-3H-indol-1-ium iodide*

The above compound was prepared from an adaptation of a previously published procedure;[3] CD$_3$-I (5 mmol, 5 equiv). The product was isolated as a dark red solid, (yield 1.34 g, 0.936 mmol, 94%).**^{1}H-NMR** (500 MHz, Chloroform-*d*) *δ* (ppm) 7.95 (d, *J* = 6.7 Hz, 2H), 7.82-7.79 (m, 1H), 7.75-7.59 (m, 6H), 1.75 (s, 6H). **^{13}C-NMR** (126 MHz, Chloroform-*d*) *δ* (ppm) 141.75, 133.36, 131.11, 129.98, 129.94, 128.64, 125.24, 123.10, 116.86, 69.9 55.90, 22.90. **HRMS (ESI+):** calcd. for $C_{17}H_{15}D_{3}N^{+}$ [M-I^{-}]: 239.1622; found 239.1602

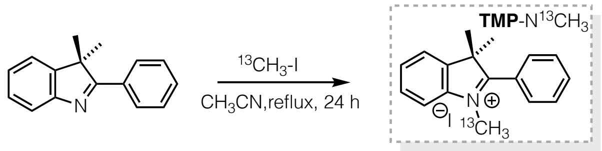

TMP-N^{13}CH$_3$: *3,3-dimethyl-2-phenyl-3H-1λ^4-indol-1-yl)methylium-^{13}C iodide*

The title compound was prepared based on a previous report;[3] using ^{13}CH$_3$-I (7 mmol, 5 equiv). The product was isolated as a yellow solid, (1.50 g, 0.501 mmol, 98%). ^{1}**H-NMR** (500 MHz, Chloroform-*d*) *δ* (ppm) 7.95 (d, *J* = 6.6 Hz, 1H), 7.83 – 7.79 (m, 2H), 7.78 – 7.62 (m, 6H), 4.37 (s, 1.5 H), 4.08 (s, 1.5 H), 1.75 (s, 6H).^{13}**C-NMR** (126 MHz, Chloroform-*d*) *δ* (ppm) 141.73, 133.29, 131.06, 129.93, 129.89, 128.66, 125.27, 123.10, 116.89, 55.86, 38.56, 29.45, 22.88. **HRMS (ESI+):** calcd. for $C_{16}{}^{13}CH_{18}N^+$ [M-I$^-$]: 237.1462; found 237.1445.

V. Reactions of Heptamethines and 3° Amines

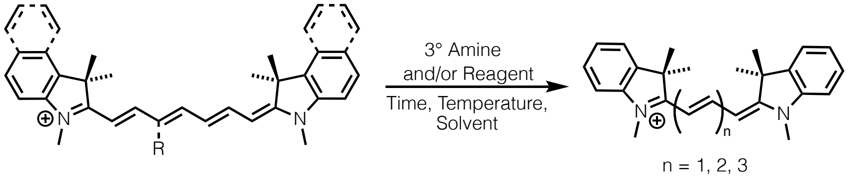

General Procedure for Reactions of Heptamethine Cyanines with 3° Amines

An oven dried (180 °C, 16 h) 15 mL threaded pressure tube was transferred, with a septum fitted over the top, to a desiccator to cool to room temperature. To maintain an inert atmosphere in further handling, an argon balloon with an 18 Ga needle was inserted, along with an outlet needle (18 Ga). Then via syringe, anhydrous acetonitrile (an appropriate volume to achieve ~0.03 mM cyanine), was added, and the solvent was degassed by bubbling with argon for a period of 15 minutes. The cyanine derivative (1 equiv) was then added by removing the septum to quickly transfer the solids, and immediately screwing on the pressure tube cap (also stored in a desiccator and removed before adding solids). The sealed reaction tube was then placed in a preheated oil bath (thermostatically controlled) at the desired temperature and stirred for the specified amount of time. The reaction was then cooled to room temperature and the solvent was removed under reduced pressure (rotavap), or alternatively for small

samples, under a flow of N_2. The resultant crude residue was re-dissolved in a minimal quantity of CH_2Cl_2 and loaded onto a flash silica column and the degradation products isolated as indicated. (see individual characterization for specific purification remarks).

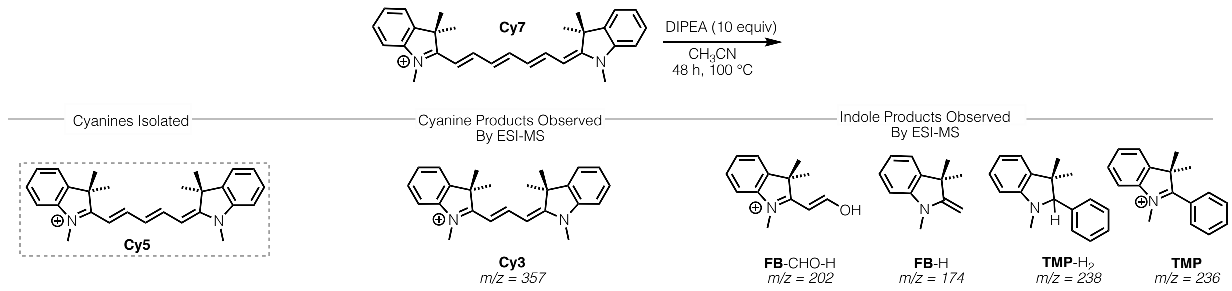

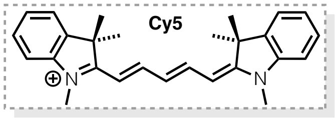

Cy5: *1,3,3-trimethyl-2-((1E,3E)-5-((E)-1,3,3-trimethylindolin-2-ylidene)penta-1,3-dien-1-yl)-3H-indol-1-ium iodide*.

Cy7 (20 mg, 0.037 mmol) was subjected to the above reaction conditions from which the major product, **Cy5**, was recovered and purified twice, via normal phase flash chromatography: [CH_2Cl_2 then gradual increase to 95:5 CH_2Cl_2/MeOH] then [CH_2Cl_2/MeOH: 99:1, then gradual increase to 98:2] yielding a metallic-blue residue (9.70 mg, 0.019 mmol, 51%). **^{1}H-NMR** (500 MHz, CD_3OD-d_4): δ (ppm) 8.23 (t, J = 13.1 Hz, 2H), 7.46 (d, J = 7.4 Hz, 2H), 7.37 (t, J = 7.7 Hz, 2H), 7.29 (d, J = 7.9 Hz, 2H), 7.22 (t, J = 7.5 Hz, 2H), 6.67 (t, J = 12.4 Hz, 1H), 6.28 (d, J = 13.7 Hz, 2H), 3.64 (s, 6H), 1.68 (s, 12H). **^{13}C-NMR** (126 MHz, CD_3OD-d_4): δ (ppm) 175.19, 155.44, 144.23, 142.52, 129.66, 126.15, 123.27, 111.79, 104.41, 50.45, 31.71, 27.86. **HRMS (ESI+):** calcd. for $C_{27}H_{31}N_2^+$ [M-I$^-$]: 383.2482; found 383.2491.

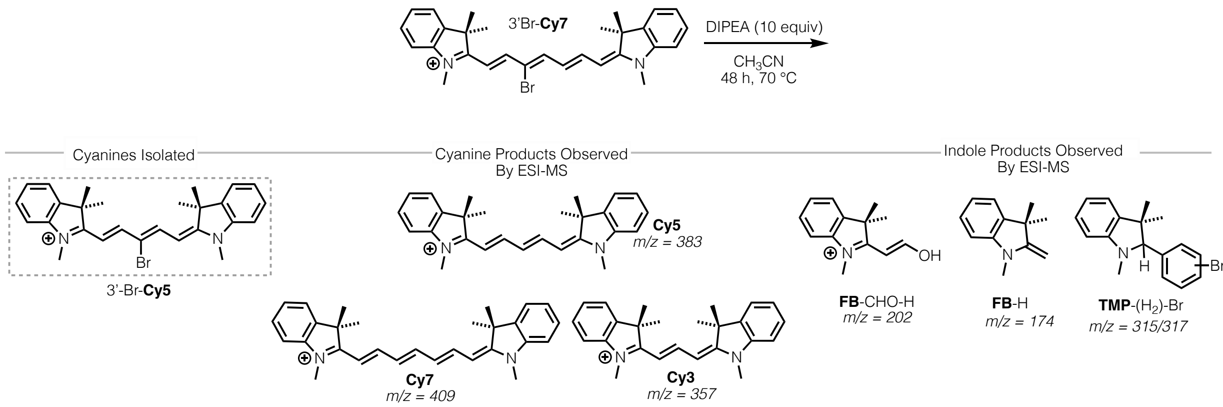

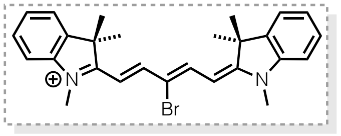

3’-Br-**Cy5**: *2-((1E,3Z)-3-bromo-5-((E)-1,3,3-trimethylindolin-2-ylidene)penta-1,3-dien-1-yl)-1,3,3-trimethyl-3H-indol-1-ium iodide*

Cy7 (20 mg, 0.037 mmol) was subjected to the above reaction conditions from which the major product 3’Br-**Cy5**, was recovered and purified twice via normal phase chromatography: [CH_2Cl_2 then gradual increase to 95:5 CH_2Cl_2/MeOH] then, [CH_2Cl_2/MeOH: 99:1, then gradual increase to 98:2] yielding a metallic-blue residue (3.1 mg, 1.80 mmol, 16%). **^{1}H-NMR** (500 MHz, CD_3OD-d_4): δ (ppm) 8.57-8.50 (m, 2H), 7.53 (dd, J = 7.8, 1.0 Hz, 2H), 7.44-7.36 (m, 2H) 7.31-7.24 (m, 4H), 5.88 (d, J = 14.1 Hz, 2H), 3.47 (s, 6H), 1.83 (s, 12H). **^{13}C-NMR** (126 MHz, CD_3OD-d_4): δ (ppm): 176.15, 153.99, 144.07, 142.53, 129.79, 126.71, 123.37, 112.16, 102.19, 50.88, 31.69, 27.65. **HRMS (ESI+):** calcd. for $C_{27}H_{30}BrN_2^+$ [M-I$^-$]: 461.1587; found 461.1592.

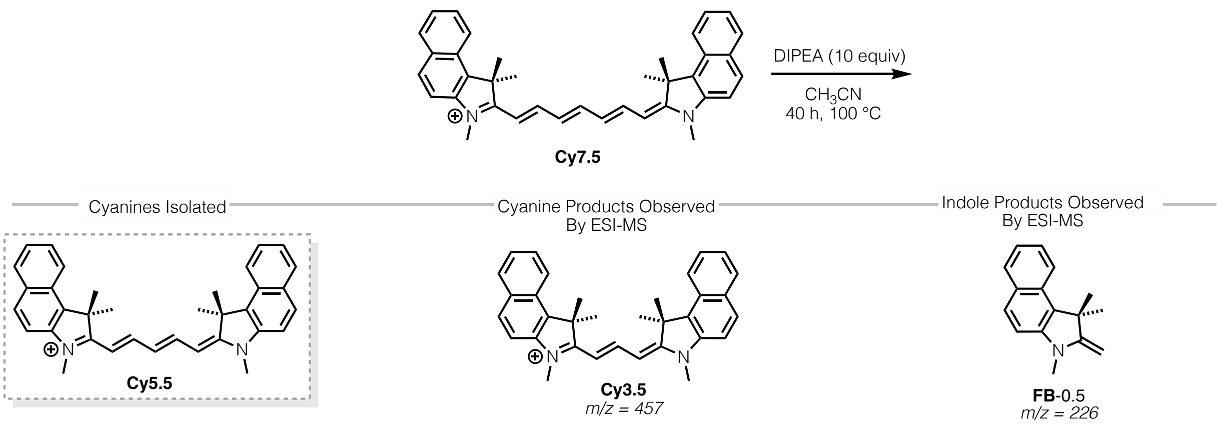

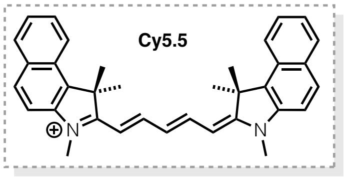

Cy5.5: *1,1,3-trimethyl-2-((1E,3E,5E)-5-(1,1,3-trimethyl-1,3-dihydro-2H-benzo[e]indol-2-ylidene)penta-1,3-dien-1-yl)-1H-benzo[e]indol-3-ium iodide*

Cy7.5 (10 mg, 0.016 mmol) was subjected to the above reaction conditions from which the major product, **Cy5.5**, was recovered and purified twice, via normal phase flash chromatography: [$CHCl_3$ then gradual increase to 92:8 $CHCl_3$/MeOH] then [CH_2Cl_2 then gradual increase to 98:2 CH_2Cl_2/MeOH] yielding a dark blue-green residue (2.14 mg, 0.004 mmol, 24%). ^{1}**H-NMR** (500 MHz, CD_3OD-d_4): δ (ppm) 8.37 (t, J = 12.4 Hz, 2H), 8.25 (d, J = 8.6 Hz, 2H), 8.04-7.98 (m, 4H), 7.67-7.60 (m, 4H), 7.50-7.46 (m 2H), 6.68 (t, J = 12.4 Hz, 1H), 6.33 (d, J = 13.8 Hz, 2H), 3.76 (s, 6H), 2.02 (s, 12H). ^{13}**C-NMR** (126 MHz, CD_3OD-d_4): δ (ppm) 176.44, 154.42, 141.68, 134.86, 133.40, 131.66, 131.12, 129.42, 128.72, 126.04, 123.34, 111.92, 103.99, 52.35, 31.83, 27.49. **HRMS (ESI+):** calcd. for $C_{35}H_{35}N_2^+$ [M-I$^-$]: 483.2795; found 483.2798.

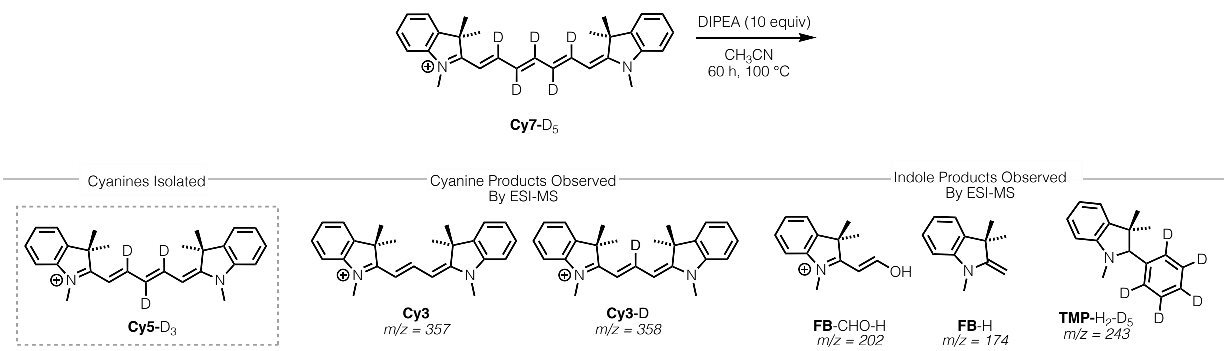

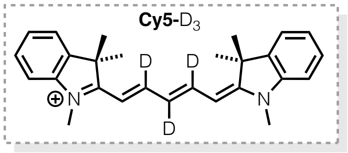

Cy5-D_3: *1,3,3-trimethyl-2-((1E,3E)-5-((E)-1,3,3-trimethylindolin-2-ylidene)penta-1,3-dien-1-yl-2,3,4-d₃)-3H-indol-1-ium iodide*

Cy7-D_5 (20 mg, 0.037 mmol) was subjected to the above reaction conditions from which the major product **Cy5**-D_3, was recovered and purified via normal phase chromatography: [CH_2Cl_2 then gradual increase to 95:5 CH_2Cl_2/MeOH] yielding a metallic blue residue (4.2 mg, 0.01 mmol, 22 %). **^{1}H-NMR** (500 MHz, CD_3OD-d_4): δ (ppm) 7.49 (dd, J = 7.5, 1.2 Hz, 2H), 7.41 (dd, J = 7.7, 1.2 Hz, 2H), 7.32-7.24 (m, 6H), 6.26 (s, 2H), 3.62, (s, 6H), 1.73 (s, 12H). **^{13}C-NMR** (126 MHz, CD_3OD-d_4): δ (ppm) 173.95, 144.3, 142.5, 129.7, 126.2, 123.3, 111.8, 104.1, 50.5, 31.5, 27.8. **HRMS (ESI+):** calcd. for $C_{27}H_{28}D_3N_2^+$ [M-I$^-$]: 386.2670; found 386.2695.

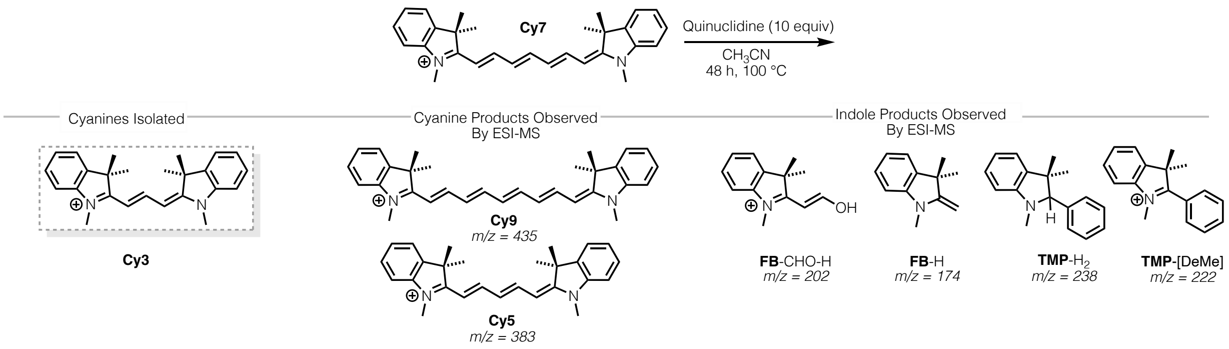

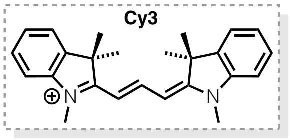

Cy3: *1,3,3-trimethyl-2-((E)-3-((E)-1,3,3-trimethylindolin-2-ylidene)prop-1-en-1-yl)-3H-indol-1-ium iodide*

Cy7 (20 mg, 0.037 mmol) was subjected to the above reaction conditions from which the major product, **Cy3**, was recovered and purified [CH_2Cl_2 then gradual increase to 95:5 CH_2Cl_2/MeOH] yielding a metallic pink-red residue (4.3 mg, 0.01 mmol, 24%). **^{1}H-NMR** (500 MHz, CD_3OD-d_4): δ (ppm) 7.49 (dd, J = 7.5, 1.2 Hz, 2H), 7.41 (dd, J = 7.7, 1.2 Hz, 2H), 7.32-7.24 (m, 6H), 6.26 (s, 2H), 3.62, (s, 6H), 1.73 (s, 12H). **^{13}C-NMR** (126 MHz, CD_3OD-d_4): δ (ppm) 176.68, 152.16, 144.09, 142.06, 129.95, 126.75, 123.38, 111.2, 103.64, 50.61, 31.71, 28.15. **HRMS (ESI+):** calcd. for $C_{25}H_{29}N_2^+$ [M-I$^-$]: 357.2325; found 357.2346.

VI. Reactions of Nucleophiles with Heptamethine Cyanines

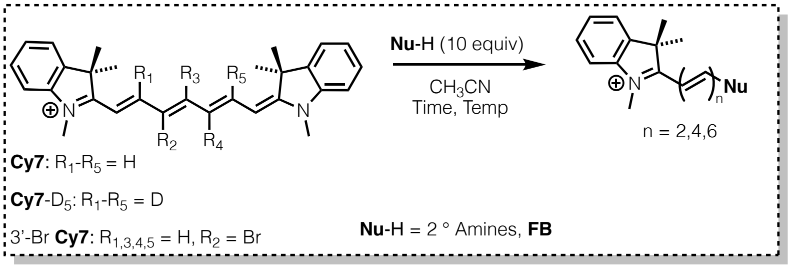

General Procedure for Nucleophiles with Heptamethine Cyanines

An oven dried (180 °C, 16 h) 15 mL threaded pressure tube was transferred, with a septum fitted over the top, to a desiccator to cool to room temperature. To maintain an inert atmosphere in further handling, an argon balloon with an 18 Ga needle was inserted, along with an outlet needle (18 Ga). Then via syringe, anhydrous acetonitrile (an appropriate volume to achieve ~0.03 mM cyanine), was added, and the solvent was degassed by bubbling with argon for a period of 15 minutes. Any liquid reagents were then added by syringe. The cyanine derivative (1 equiv) and other solids were then added by removing the septum to quickly complete the transfer, and immediately screwing on the pressure tube cap (also stored in a desiccator and removed before adding solids). The sealed reaction tube was then placed in a preheated oil bath (thermostatically controlled) at the desired temperature and stirred for the specified amount of time. The reaction was then cooled to room temperature and the solvent was removed under reduced pressure (rotavap), or alternatively for small samples, under a flow of N_2. The resultant crude residue was re-dissolved in a minimal quantity of CH_2Cl_2 and loaded onto a flash silica column and the degradation products isolated as indicated. (see individual characterization for specific purification remarks).

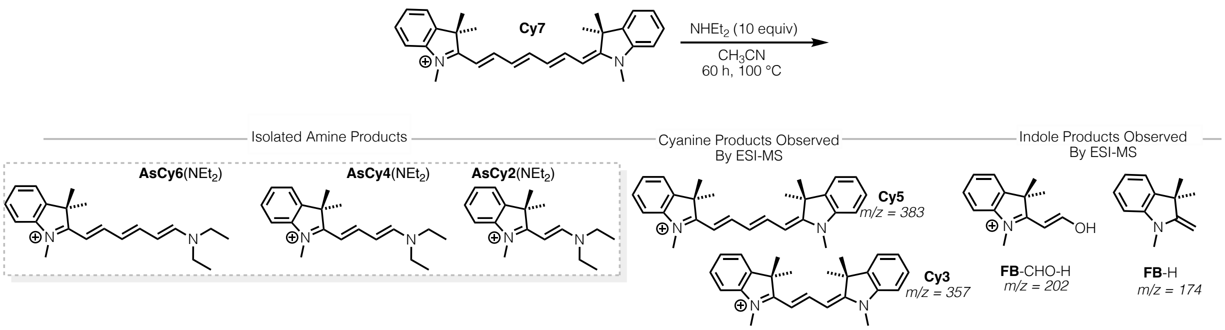

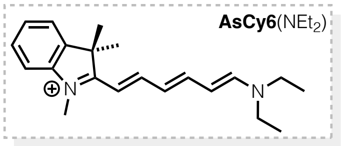

AsCy6(NEt₂): *2-((1E,3E,5E)-6 (diethylamino)hexa-1,3,5-trien 1-yl)-1,3,3-trimethyl-3H-indol-1-ium iodide*

Cy7 (20 mg, 0.037 mmol) was subjected to the above reaction conditions from which the major product **AsCy6**(NEt₂) was recovered and purified twice, via normal phase chromatography: [CH_2Cl_2 to 97:3 CH_2Cl_2/MeOH] then [CH_2Cl_2/MeOH 99:1] yielding a metallic-purple red solid (4.3 mg, 0.001 mmol, 27%). ^{1}**H-NMR** (500 MHz, CD_3OD-d₄): δ (ppm) 7.91 (t, *J* = 13.1 Hz, 1H), 7.83 (d, *J* = 11.7 Hz, 1H), 7.64 (t, *J* = 12.7 Hz, 1H), 7.43-7.39 (m, 1H), 7.37-7.32 (m, 1H), 7.16-7.14 (m, 2H), 6.48 (t, *J* = 12.6 Hz, 1H), 6.19 (t, *J* = 13.1 Hz, 1H), 6.02 (d, *J* = 13.1 Hz, 1H), 3.65 (q, *J* = 7.3 Hz, 4H), 3.48 (s, 3H), 1.65 (s, 6H), 1.36 (t, *J* = 7.2 Hz, 3H), 1.31 (t, *J* = 7.3 Hz, 3H). ^{13}**C-NMR** (126 MHz, CD_3OD-d₄): δ (ppm) 172.64, 163.00, 162.88, 153.96, 144.62, 129.54, 124.98, 123.38, 123.09, 110.70, 109.39, 101.22, 53.40, 45.62, 30.84, 28.19, 14.52, 12.86. **HRMS (ESI+):** calcd. for $C_{21}H_{29}N_2^+$ [M-I$^-$]: 309.2325; found 309.2350.

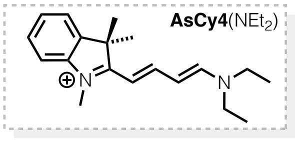

AsCy4(NEt₂): *2-((1E,3E)-4-(diethylamino)buta-1,3-dien-1-yl)-1,3,3-trimethyl-3H-indol-1-ium iodide*

The title compound was isolated and purified as described for **AsCy6**(NEt_2) above. The product was isolated as a metallic-yellow red solid **^{1}H-NMR** (500 MHz, CD_3OD-d_4): δ (ppm) 8.19 (dd, J = 13.6, 12.5 Hz, 1H), 8.02 (d, J = 11.7 Hz, 1H), 7.47-7.44 (m, 1H), 7.41-7.37 (m, 1H), 7.25-7.21 (m, 2H), 6.23-6.17 (m, 1H), 6.15 (d, J = 13.6 Hz 1H), 3.64 (qd, J = 7.3, 2.4 Hz, 4H), 3.54 (s, 3H), 1.68 (s, 6H), 1.37 (t, J = 7.2 Hz, 3H), 1.32 (t, J = 7.2 Hz, 3H). **^{13}C-NMR** (126 MHz, CD_3OD-d_4): δ (ppm) δ 175.72, 162.96, 159.10, 155.40, 130.87 129.64, 125.74, 123.19, 111.33, 108.24, 50.13, 45.41, 30.87, 28.07, 14.55, 12.79. **HRMS (ESI+):** calcd. for $C_{19}H_{27}N_2^+$ [M-I$^-$]: 283.2169; found 283.2195.

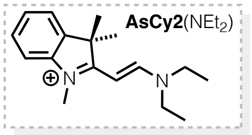

AsCy2(NEt_2): *(E)-2-(2-(diethylamino)vinyl)-1,3,3-trimethyl-3H-indol-1-ium iodide*

The title compound was isolated and purified as described for **AsCy6**(NEt_2) above. The product was isolated as a metallic-orange yellow solid. **^{1}H-NMR** (500 MHz, CD_3OD-d_4): δ (ppm) 8.27 (d, J = 12.8 Hz, 1H), 7.50 (dd, J = 7.4, 1.2 Hz, 1H), 7.43 (td, J = 7.7, 1.2 Hz, 1H), 7.34-7.27 (m, 2H), 3.80-3.70 (m, 4H), 3.62 (s, 3H), 1.68 (s, 6H), 1.41-1.35 (m, 6H). **HRMS (ESI+):** calcd. for $C_{17}H_{25}N_2^+$ [M-I$^-$]: 257.2012; found 257.2047.

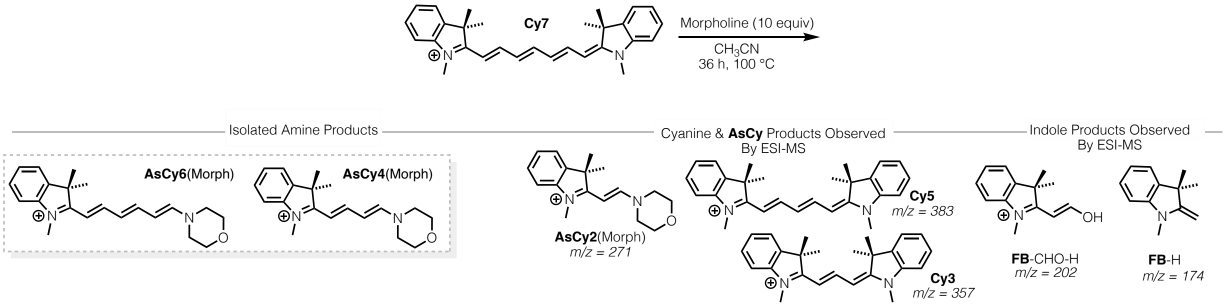

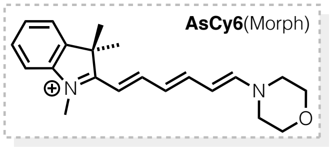

AsCy6(Morph): *4-((2E,4E)-6-((E)-1,3,3-trimethylindolin-2-ylidene)hexa-2,4-dien-1-ylidene)morpholin-4-ium iodide*

The title compound was isolated from the conditions shown above and purified via normal phase chromatography: [CH_2Cl_2 to 95:5 CH_2Cl_2/MeOH] yielding a metallic purple-red solid. **^{1}H-NMR** (500 MHz, CD_3OD-d_4): δ (ppm) 7.96 (t, J = 13.1 Hz, 1H), 7.77 (t, J = 11.7 Hz, 1H), 7.65 (t, J = 12.7 Hz, 1H), 7.45-7.42 (m, 1H), 7.37 (t, J = 7.6 Hz, 1H), 7.22-7.18 (m, 2H), 6.48 (t, J = 12.7 Hz, 1H), 6.21 (t, J = 11.7 Hz, 1H), 6.08 (d, J = 13.5 Hz, 1H), 3.85-3.80 (m, 4H), 3.75-3.72 (m, 4H), 3.52 (s, 3H), 1.66 (s, 6H). **^{13}C-NMR** (126 MHz, CD_3OD-d_4): δ (ppm) 163.33, 162.78, 161.68, 154.82, 141.81, 130.78 129.70, 125.47, 123.14, 111.10, 108.01, 49.63, 33.08, 30.46, 31.03, 28.08, 23.74, 14.43. **HRMS (ESI+):** calcd. for $C_{21}H_{27}N_2O^+$ [M-I$^-$]: 323.2118; found 323.2140.

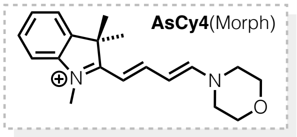

AsCy4(Morph): *4-((E)-4-((E)-1,3,3-trimethylindolin-2-ylidene)but-2-en-1-ylidene)morpholin-4-ium iodide*

The title compound was isolated and purified as described for **AsCy6**(Morph) above, as a metallic-yellow red solid. **^{1}H-NMR** (500 MHz, CD_3OD-d_4): δ (ppm) 8.21 (t, J = 12.9 Hz, 1H), 7.98 (d, J = 11.7 Hz, 1H), 7.47 (d, J = 7.4 Hz, 1H), 7.40 (td, J = 7.7, 1.2 Hz, 1H), 7.28-7.23 (m, 2H), 6.24 (t, J = 12.7 Hz, 1H), 6.18 (t, J = 13.7 Hz, 1H), 3.86-3.81 (m, 4H), 3.76-3.71 (m, 4H), 3.57, (s, 3H), 1.68 (s, 6H).**^{13}C-NMR** (126 MHz, CD_3OD-d_4): δ (ppm) 176.27, 162.48, 159.47, 144.15, 142.03, 129.68, 126.02, 123.23, 111.61, 107.26, 101.04, 68.08, 67.10, 56.17, 30.78, 28.04. **HRMS (ESI+):** calcd. for $C_{19}H_{25}N_2O^+$ [M-I$^-$]: 297.1961; found 297.1995.

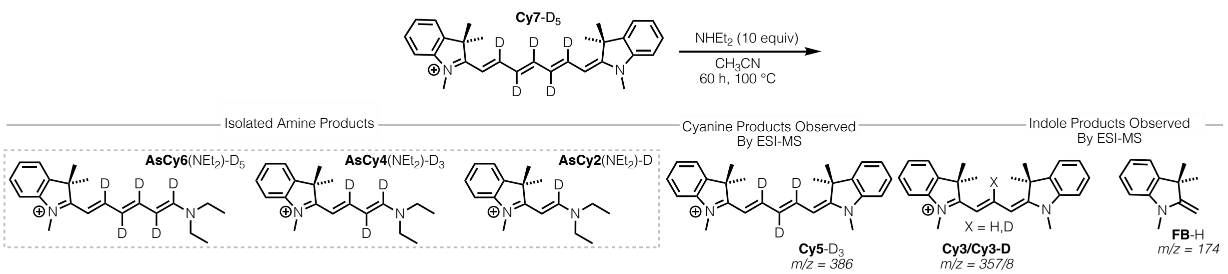

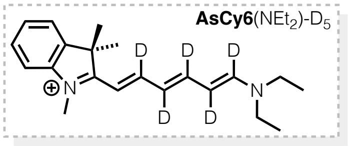

AsCy6(NEt$_2$)-D$_5$: *2-((1E,3E,5E)-6-(diethylamino)hexa-1,3,5-trien-1-yl-2,3,4,5,6-d5)-1,3,3-trimethyl-3H-indol-1-ium*

The title compound was isolated from the conditions shown above and purified via normal phase chromatography: [CH_2Cl_2 to 97:3 CH_2Cl_2/MeOH] yielding a metallic purple-red solid. ^{1}**H-NMR** (500 MHz, CD_3OD-d$_4$): δ (ppm) 7.42 – 7.39 (m, 1H), 7.34 (td, J = 7.8, 1.1 Hz, 1H), 7.16 (t, J = 7.2 Hz, 2H), 6.01 (s, 1H), 3.65 (q, J = 7.2 Hz, 4H), 3.48 (s, 3H), 1.65 (s, 6H), 1.36 (t, J = 7.2 Hz, 6H). ^{13}**C-NMR** (126 MHz, CD_3OD-d$_4$): δ (ppm) 172.65, 163.07, 144.63, 141.66, 129.54, 124.97, 123.09, 110.69, 101.04, 45.59, 30.77, 28.19, 14.50, 12.85. **HRMS (ESI+)**: calcd. For $C_{21}H_{24}D_5N_2^+$[M-I$^-$]: 314.2639; found 314.2662

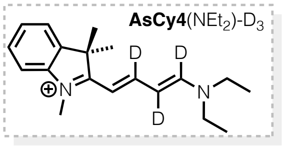

AsCy4(NEt$_2$)-D$_3$: *2-((1E,3E)-4-(diethylamino)buta-1,3-dien-1-yl-2,3,4-d$_3$)-1,3,3-trimethyl-3H-indol-1-ium iodide*

The title compound was obtained according to general procedure above, with **Cy7**-D_5 and diethylamine. The product was isolated a metallic-yellow red residue. **^{1}H-NMR** (500 MHz, CD_3OD-d_4): δ (ppm) 7.45 (d, J = 7.4 Hz, 1H), 7.39 (d, J = 7.9 Hz, 1H), 7.25-7.21 (m, 2H), 6.15 (s, 1H), 3.64 (qd, J = 7.3, 1.5 Hz, 4H), 3.54 (s, 3H), 1.68 (s, 6H), 1.37 (t, J = 7.3 Hz, 3H), 1.34 (t, J = 7.3 Hz, 3H). **^{13}C-NMR** (126 MHz, CD_3OD-d_4): δ (ppm) 174.0, 161.8, 161.6, 161.4, 142.8, 140.4, 128.2, 124.3, 109.9, 98.9, 28.0, 14.5, 12.7 **HRMS (ESI+):** calcd. for $C_{19}H_{24}D_3N_2^+$[M-I$^-$]:286.2357; found 286.2387.

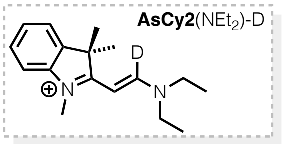

AsCy2(NEt$_2$)-D: *(E)-2-(2-(diethylamino)vinyl-2-d)-1,3,3-trimethyl-3H-indol-1-ium iodide*

The title compound was isolated and purified, as described for **AsCy6**(NEt$_2$)-D_5. The product was isolated as a metallic-yellow gold residue. **^{1}H-NMR** (500 MHz, CD_3OD-d_4): δ (ppm) 7.50 (dd, J = 7.4, 1.2 Hz, 1H), 7.44 (td, J = 7.8, 1.2 Hz, 1H), 7.34-7.28 (m, 2H), 5.67 (s, 1H), 3.75 (dq, J = 21.6, 7.3 Hz, 4H), 3.62 (s, 3H), 1.68 (s, 6H), 1.41-1.37 (m, 6H). **^{13}C-NMR** (126 MHz, CD_3OD-d_4): δ (ppm) 179.59, 163.07, 143.80, 129.78, 126.51, 123.26, 111.88, 87.45, 53.52, 49.56, 28.37, 14.64, 12.08.

VII. Reaction of **IR-786** and **FB**

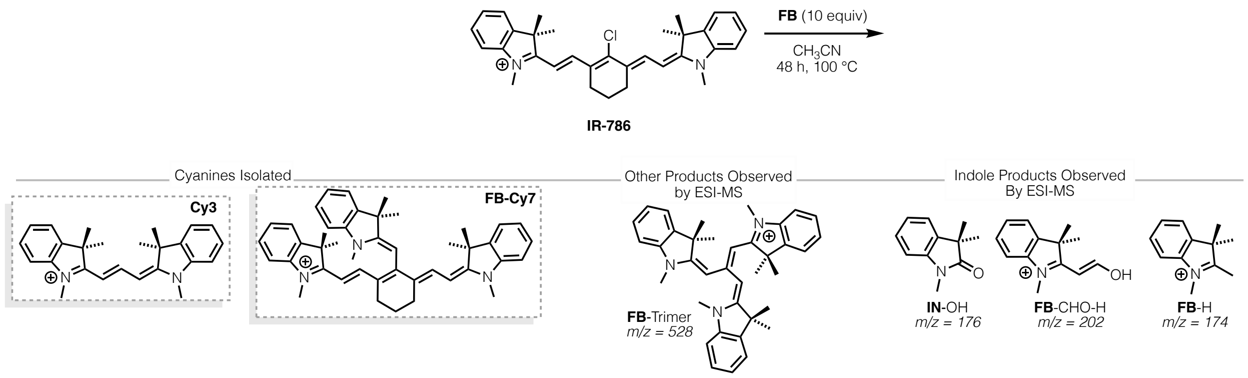

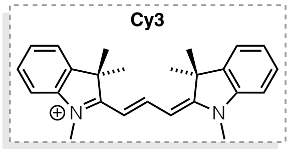

Cy3: *1,3,3-trimethyl-2-((E)-3-((E)-1,3,3-trimethylindolin-2-ylidene)prop-1-en-1-yl)-3H-indol-1-ium iodide*

IR-786 (25 mg, 0.041 mmol) was subjected to the above reaction conditions from which the major product, Cy3, was recovered and purified and characterized as previously described for Cy3 (page S18) (5.95 mg, 0.012 mmol, 30%).

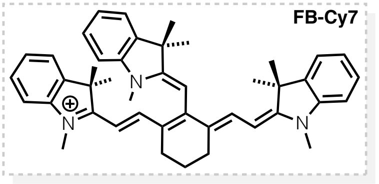

FB-Cy7: *1,3,3-trimethyl-2-((E)-2-((E)-3-(2-((E)-1,3,3-trimethylindolin-2-ylidene)ethylidene)-2-(((Z)-1,3,3-trimethylindolin-2-ylidene)methyl)cyclohex-1-en-1-yl)vinyl)-3H-indol-1-ium iodide*

^{1}H-NMR (500 MHz, Chloroform-*d*) δ (ppm) 8.00 (d, *J* = 13.9 Hz, 2H), 7.33 (d, *J* = 7.8 Hz, 2H) 7.20 (m, 3H), 7.13 (d, *J* = 7.2 Hz, 3H), 7.07 (d, *J* = 8.2 Hz, 2H), 6.93 (d, *J* = 7.4 Hz, 1H), 6.60 (d, *J* = 7.9 Hz, 1H), 6.14 (d, *J* = 13.9 Hz,

2H), 5.18 (s, 1H), 3.66 (s, 6H), 3.04 (s, 3H), 2.85-2.73 (m, 2H), 2.69-2.58 (m, 2H) 1.68 (s, 6H), 1.60 (s, 6H), 1.32 (s,6H). **HRMS (ESI+):** calcd. for $C_{44}H_{50}N_3^+$ [M-I$^-$]: 620.3999; found 620.4014.

Isolation of 3'-Br-**AsCy6**(NEt$_2$) from 3'-Br-**Cy7** + NHEt$_2$

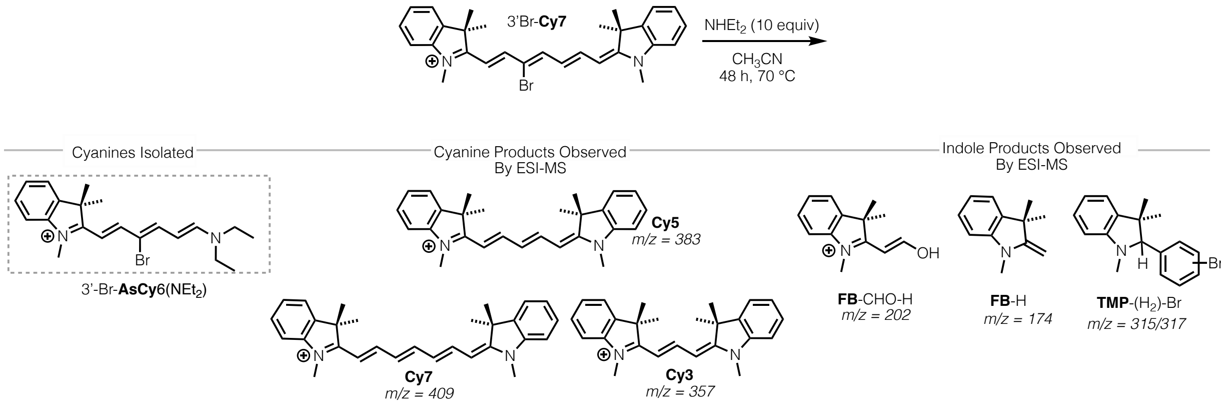

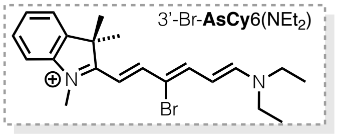

Identical conditions as described previously for **Cy7** and diethylamine were used in the preparation of and isolation of the above compound [CH_2Cl_2 to 97:3 CH_2Cl_2/MeOH], isolated as a dark purple residue ^{1}**H-NMR** (500 MHz, CD_3OD) δ (ppm) 8.04 (d, J = 13.1 Hz, 1H), 7.97 (d, J = 11.6 Hz, 1H), 7.82 (d, J = 12.6 Hz, 1H), 7.49 -7.47 (m, 1H), 7.40 (dd, J = 7.9, 1.2 Hz, 1H), 7.30-7.24 (m, 2H), 6.28 (dd, J = 25.1, 12.1 Hz, 1H), 6.20 (d, J = 13.0 Hz, 1H), 3.74-3.70 (m, 4H), 3.60 (s, 3H), 1.68 (s, 6H), 1.39 (t, J = 7.4 Hz, 6H). ^{13}**C-NMR** δ (ppm) 163.19, 159.21, 150.20, 129.89, 126.22, 123.45, 111.77, 108.65, 100.74, 46.14, 31.49, 28.00, 14.62, 12.88. **HRMS (ESI+):** calcd. For $C_{21}H_{28}BrN_2^+$[M-I$^-$]: 387.1430; found 387.1449.

VIII. Supplementary Figures

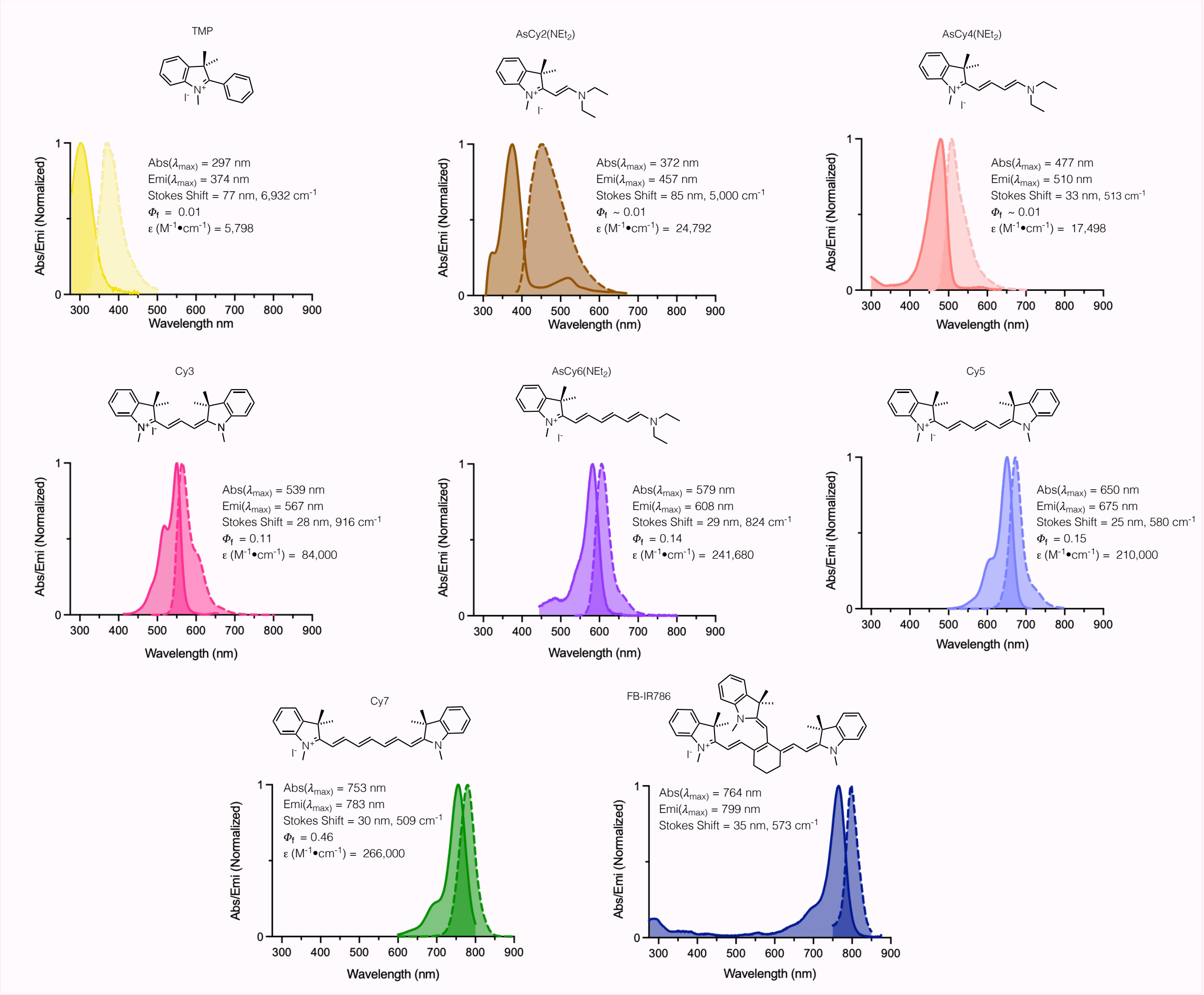

Figure S1. Normalized absorption/emission, Stokes shift, extinction coefficient, and absolute fluorescence quantum yield (Φ_f) for select species, in dichloromethane. Emission wavelength (dashed) measured from the maximum excitation wavelength (solid). ε values calculated using an internal ^{1}H-NMR standard (MTBE).

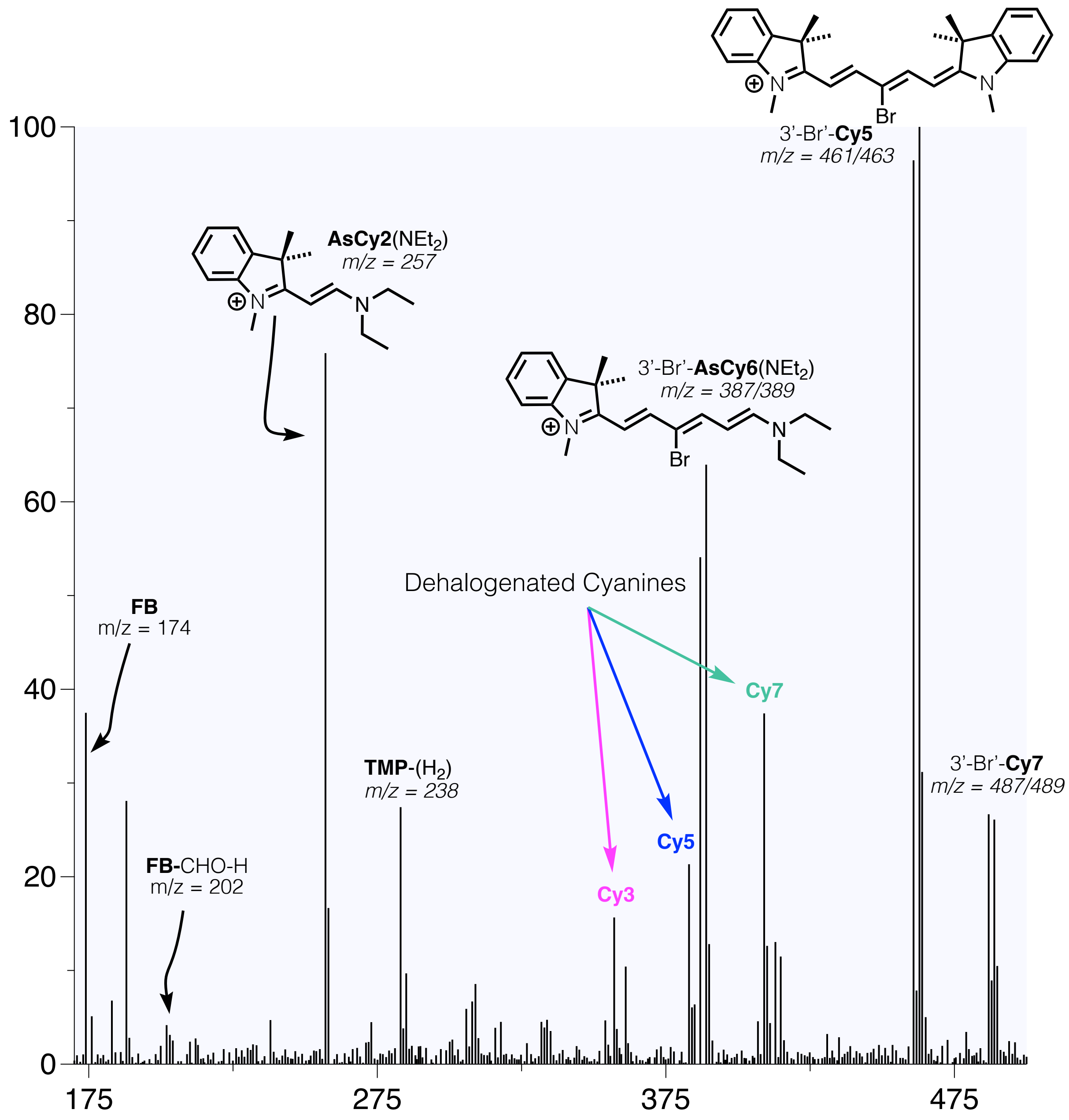

Figure S2. Crude ESI-MS from reaction of 3'-Br-**Cy7** and DIPEA (2 equiv) and diethylamine (10 equiv) at 70 °C, 60 h. Dehalogenated cyanines are shown above in color.

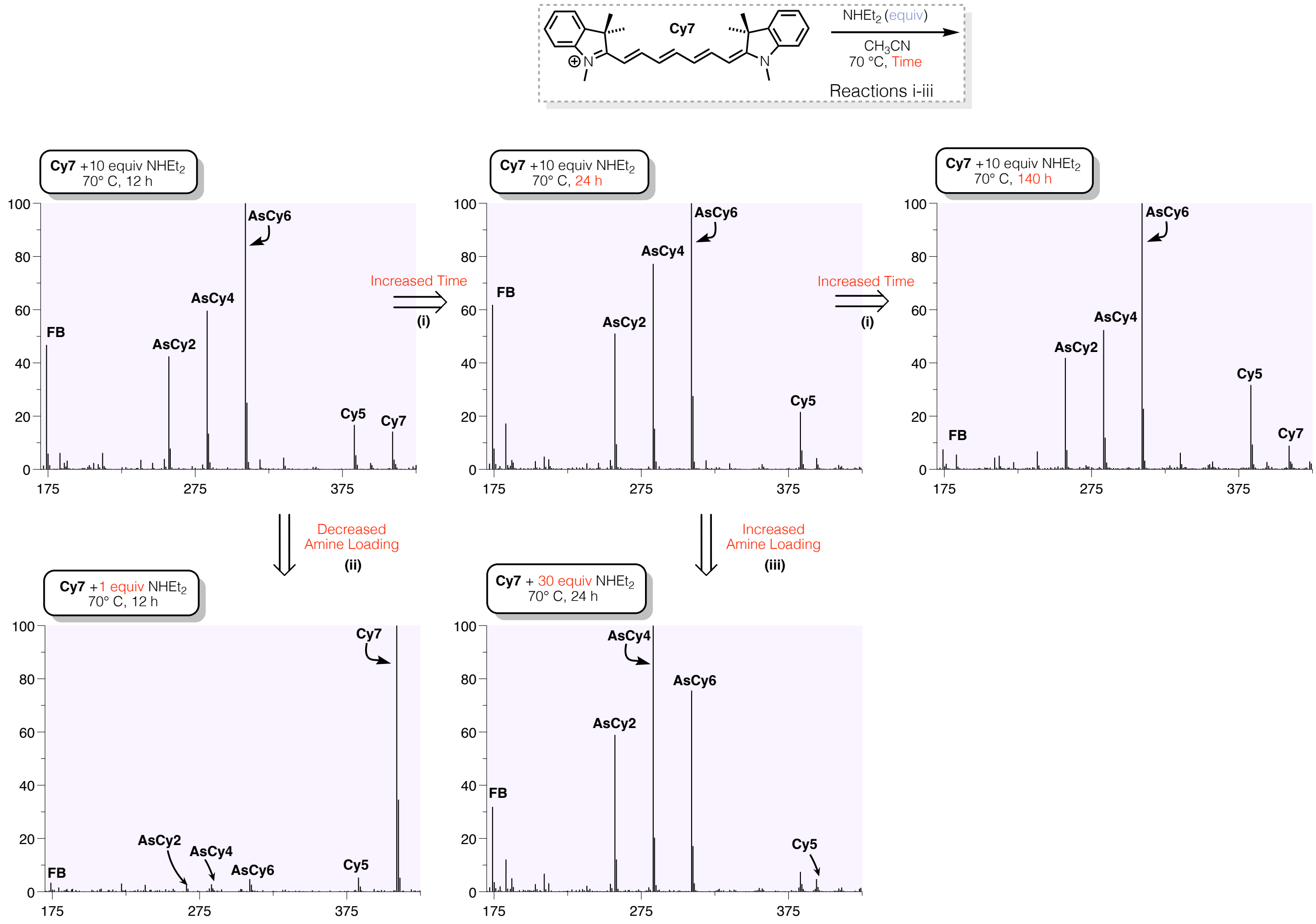

Figure S3. Scope of parameters explored with **Cy7** and diethylamine; (**i**) increased reaction time, **(ii)** decreased amine loading **(iii)** increased amine loading. Data above reflects crude ESI-MS traces from the depicted time point.

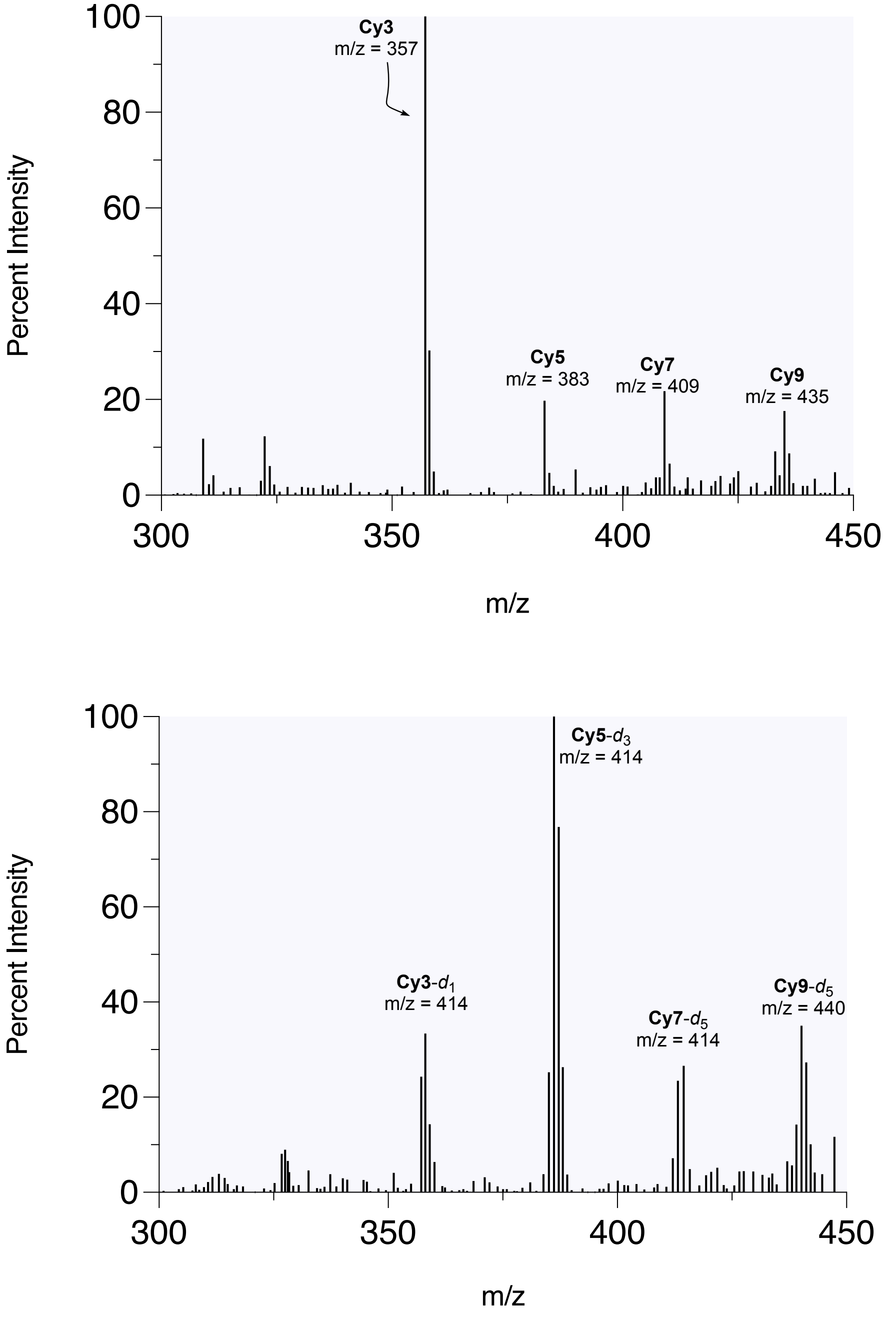

Figure S4. Crude ESI-MS trace from reaction of **Cy7** (top) and **Cy7**-d_5 (bottom) with quinuclidine (10 equiv) at 100 °C showing formation of nonamethine **Cy9** (m/z = 435), and corresponding penta-deutero analogue **Cy9**-d_5 (m/z = 440).

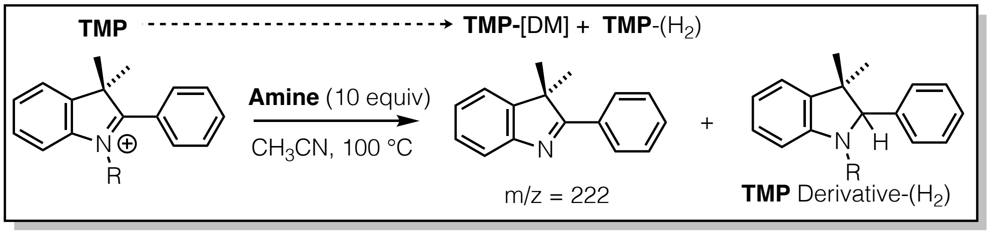

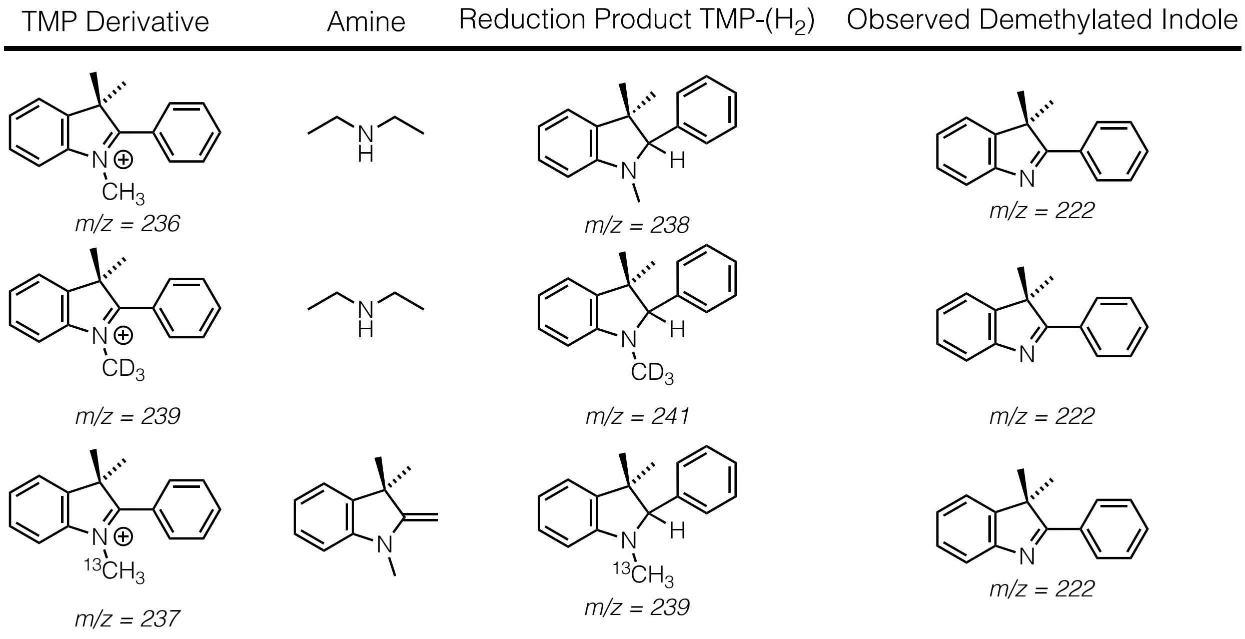

Figure S5. Reactions of **TMP** and ^{2}H and ^{13}C labeled derivatives with amines and **FB** producing species **TMP**-[DeMe]. The m/z of all neutral species shown above are observed as protonated (M+H) in ESI-MS.

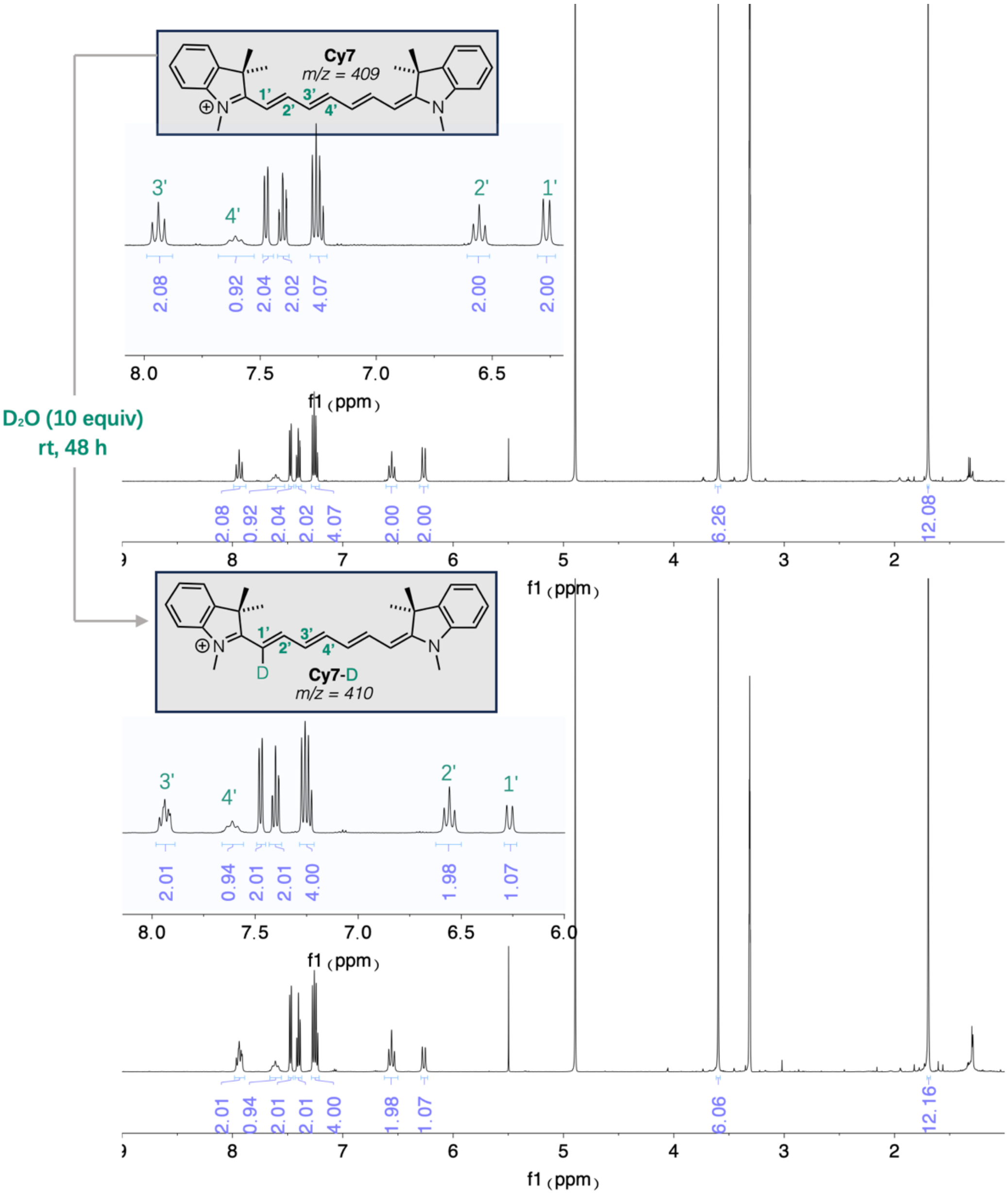

Figure S6. Comparison of ^{1}H-NMR of **Cy7** before treatment with D_2O (10 equiv) at 100 °C (top) and monodeuterated **Cy7-D** after isolation (bottom). Methine protons assignments from both species denoted in green.

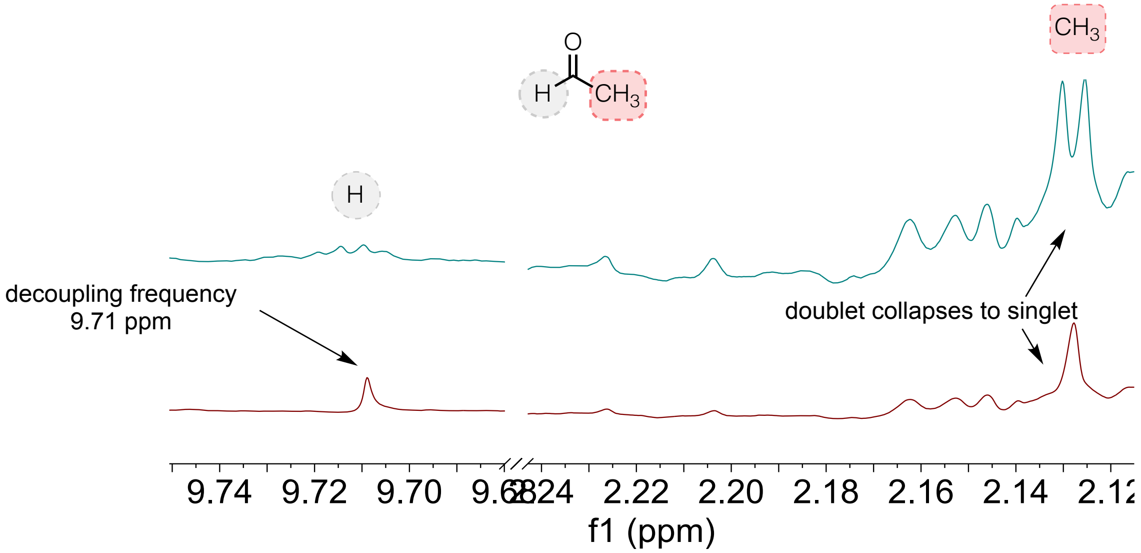

Figure S7. Selective homonuclear decoupling NMR experiment irradiating at 9.71 ppm, and collapse of the doublet corresponding to the acetaldehyde CH_3.

Core Mechanistic Reactions (a-f)

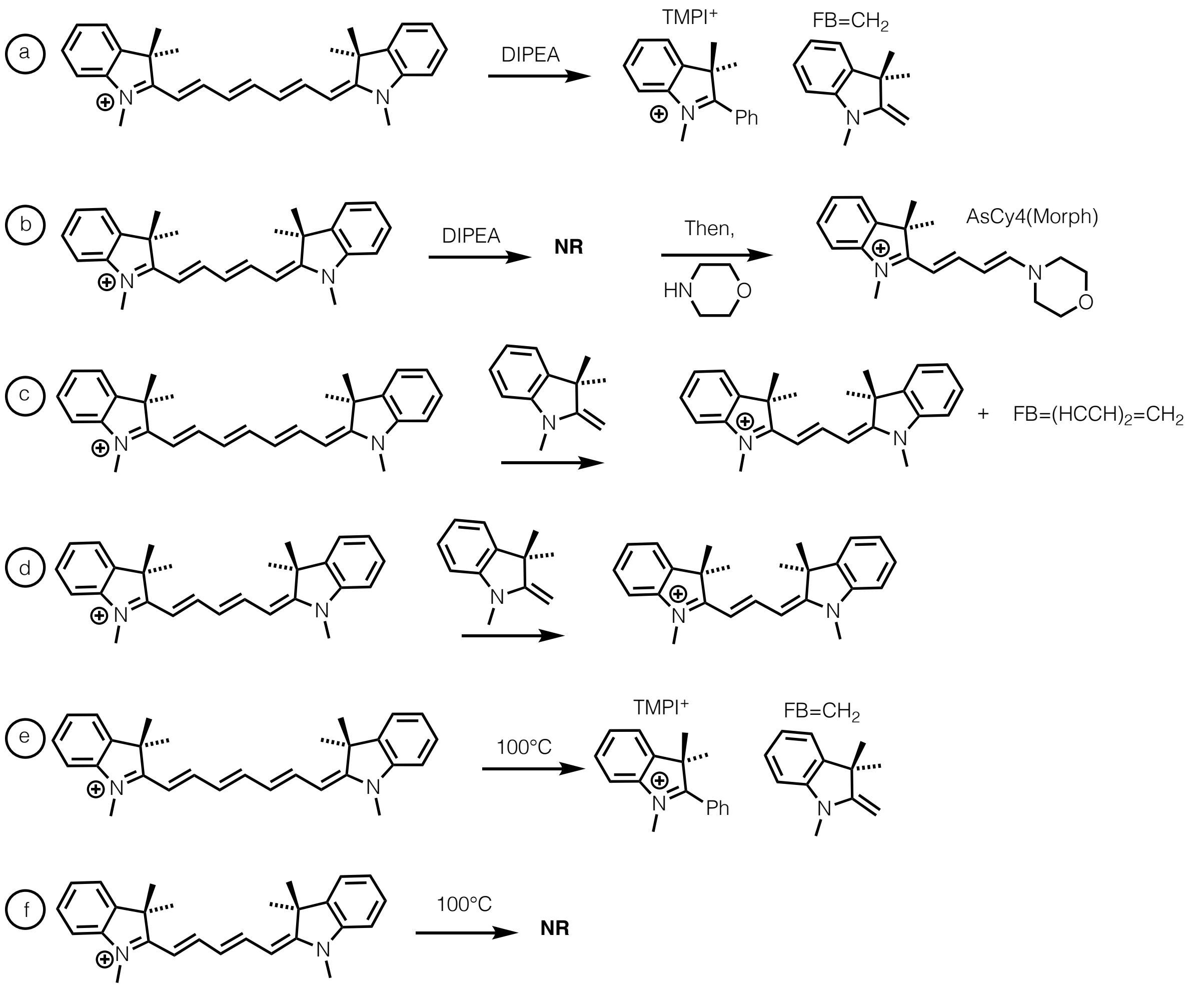

Figure S8. Summary of reactions used for mechanistic studies.

IX. NMR Spectra

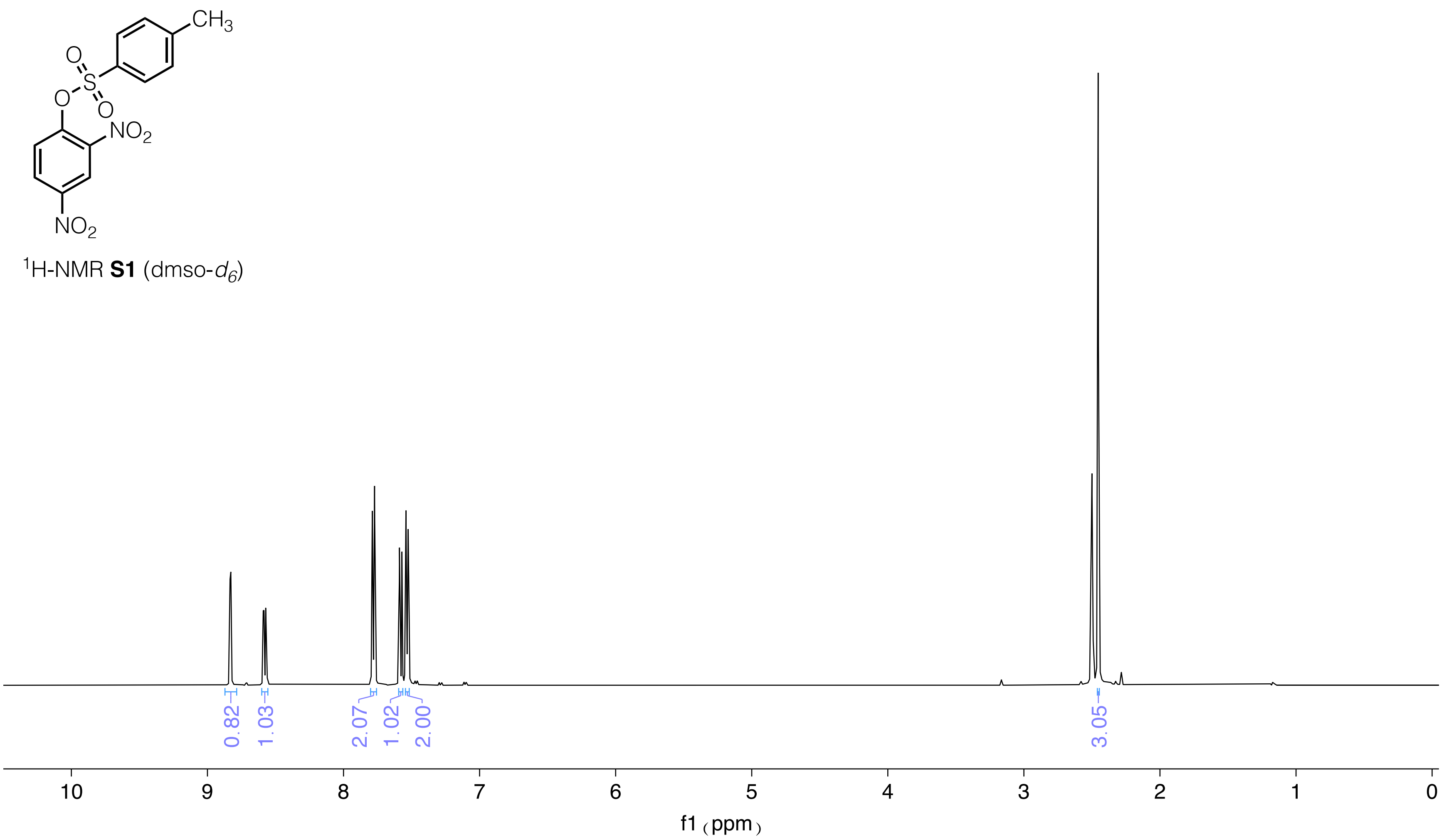

^{1}H-NMR **S1** (dmso-d_6)

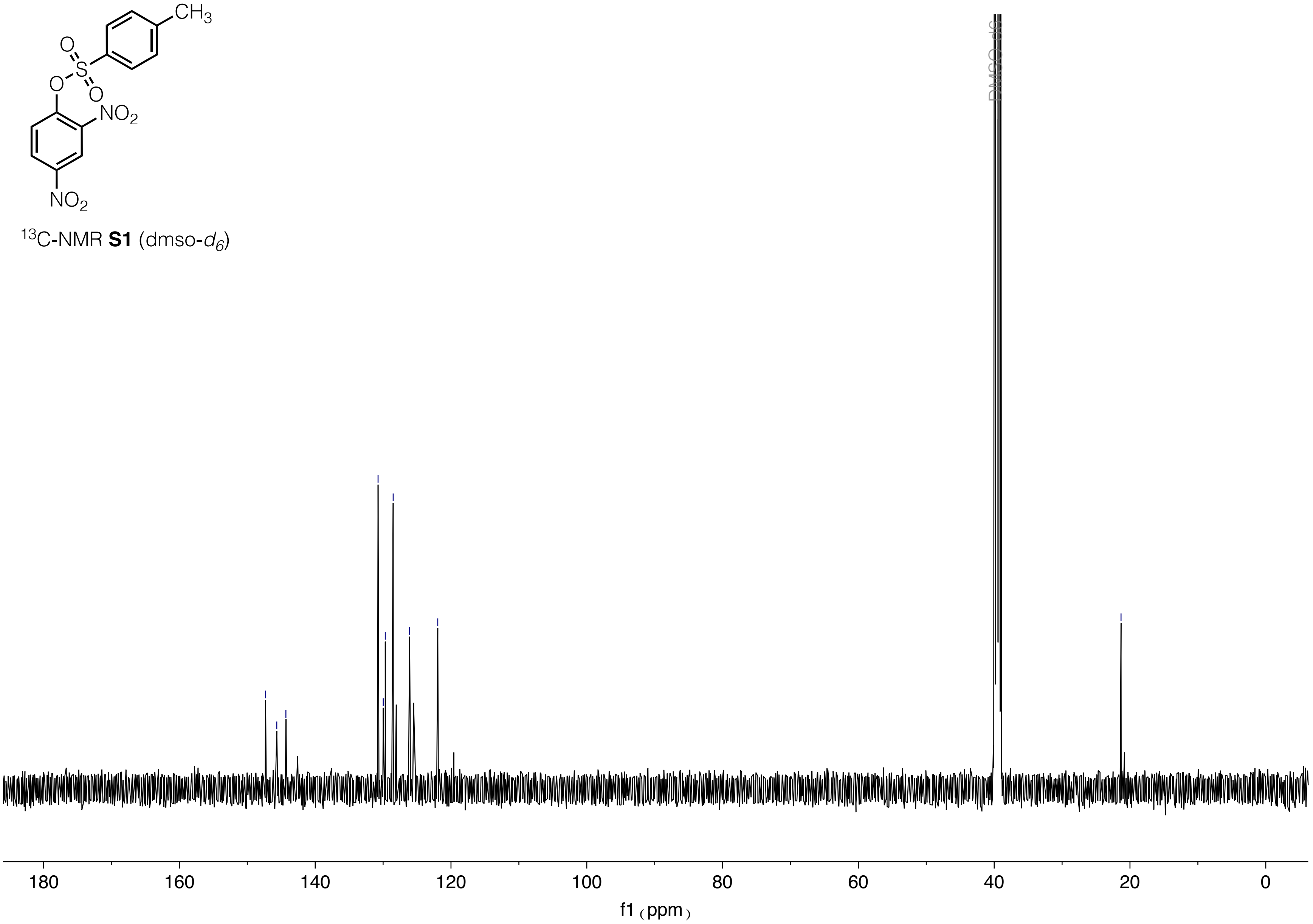

^{13}C-NMR **S1** (dmso-d_6)

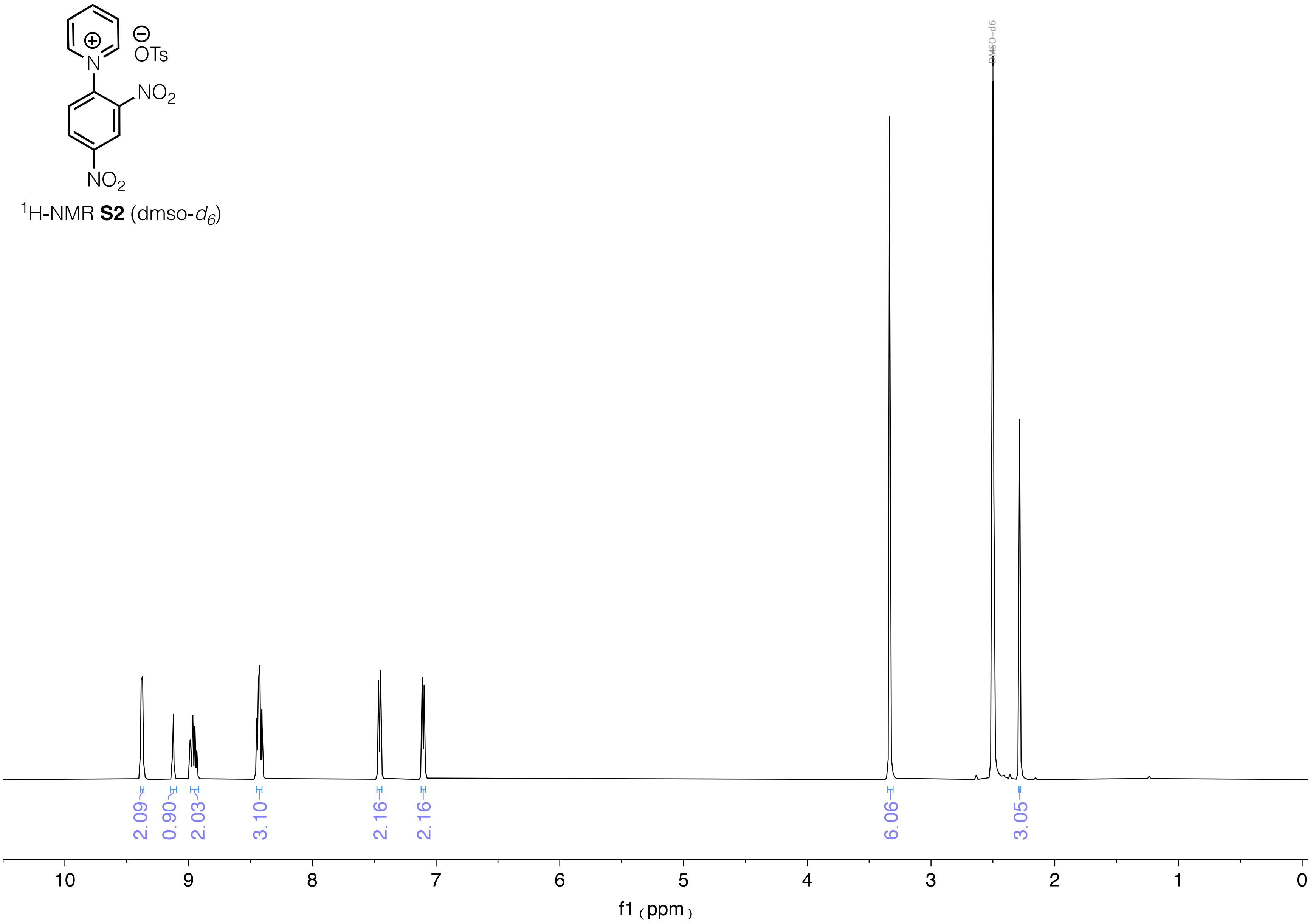

^{1}H-NMR **S2** (dmso-d_6)

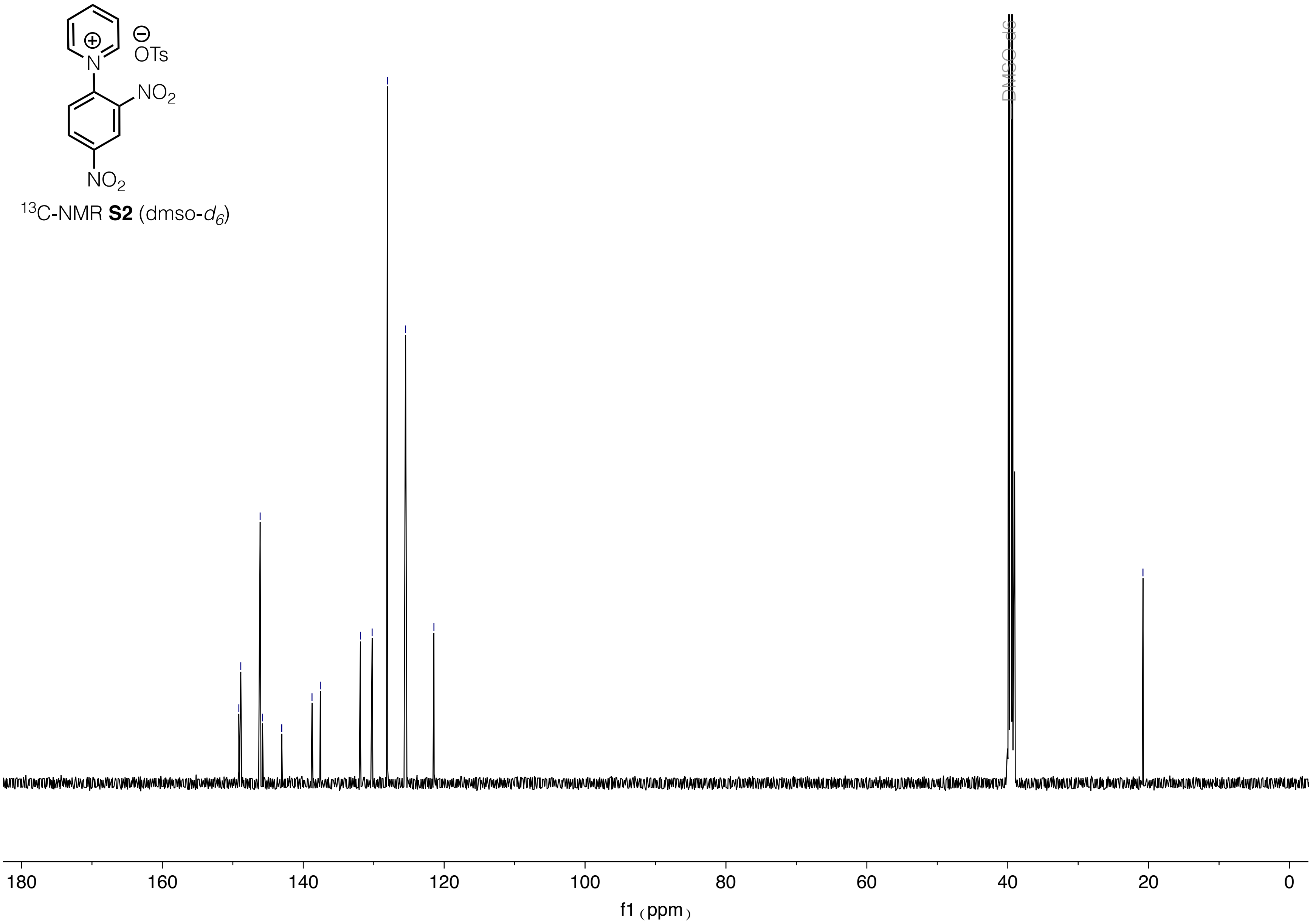
OTs
NO2
NO2
13C-NMR S2 (dmso-d6)
DMSO-d6
180
160
140
120
100
80
60
40
20
0
f1 (ppm)

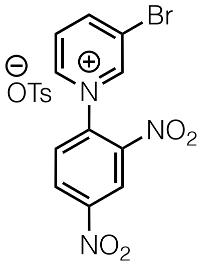

^{1}H-NMR **S3** (dmso-d_6)

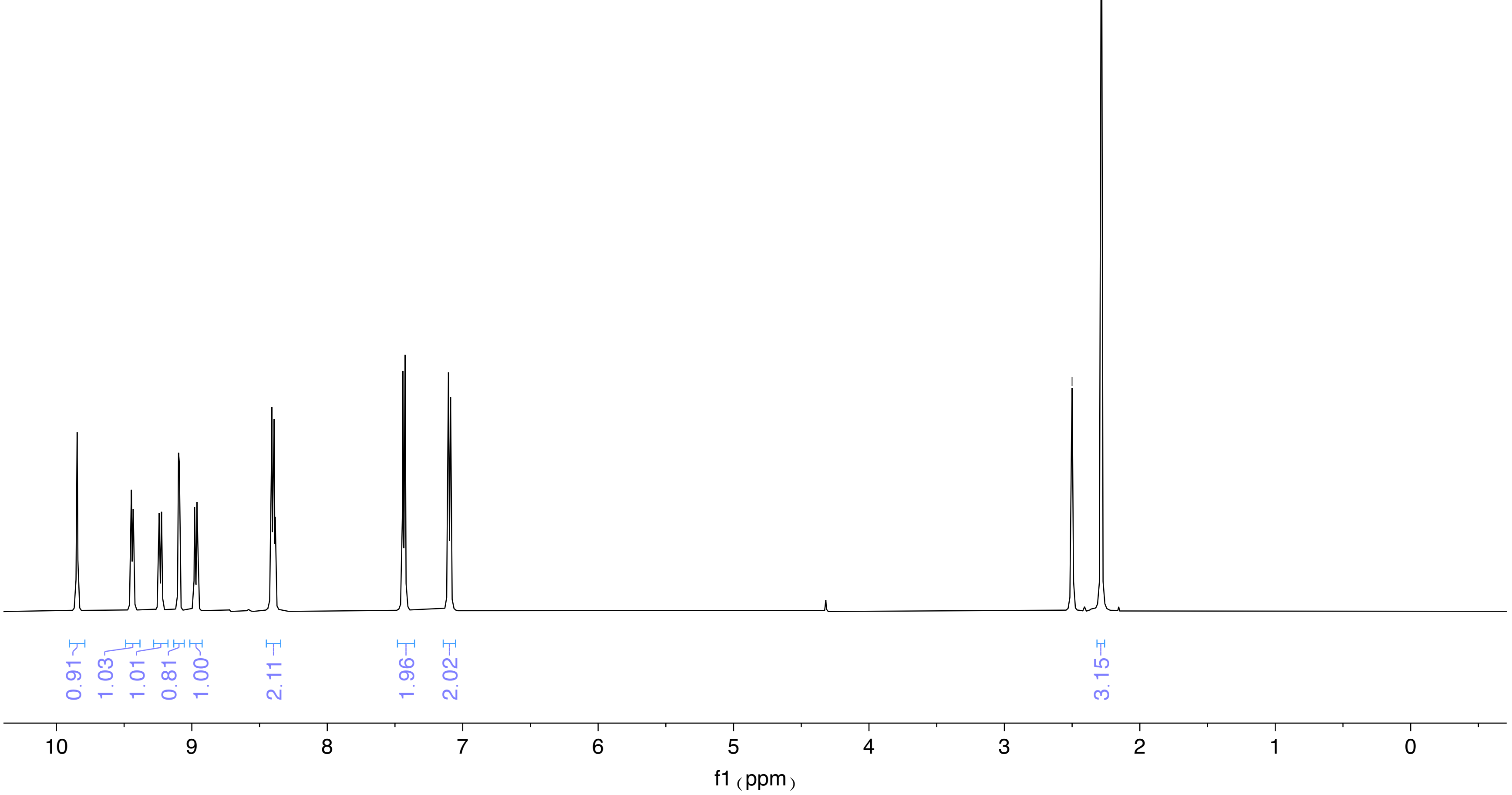

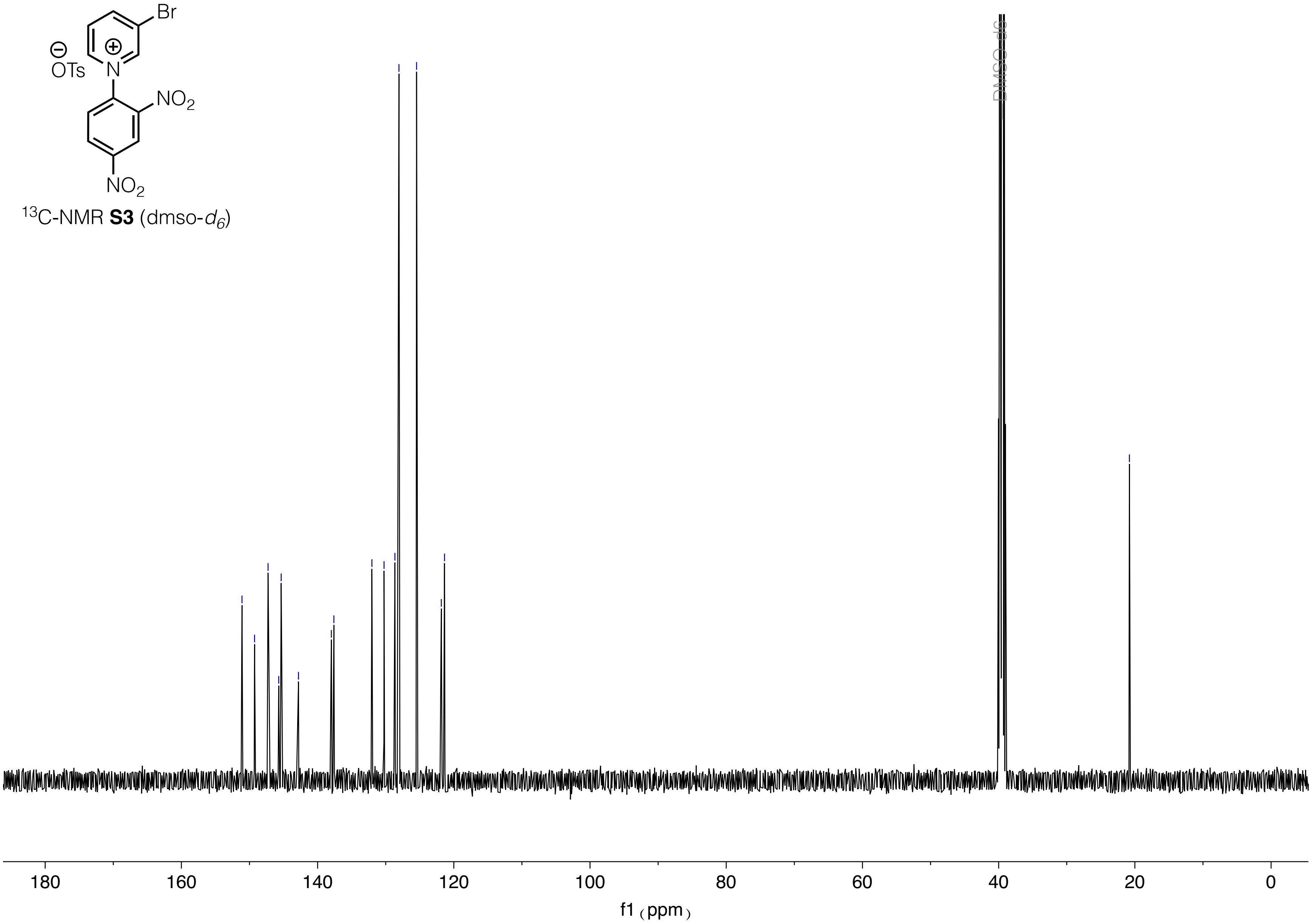

^{13}C-NMR **S3** (dmso-d_6)

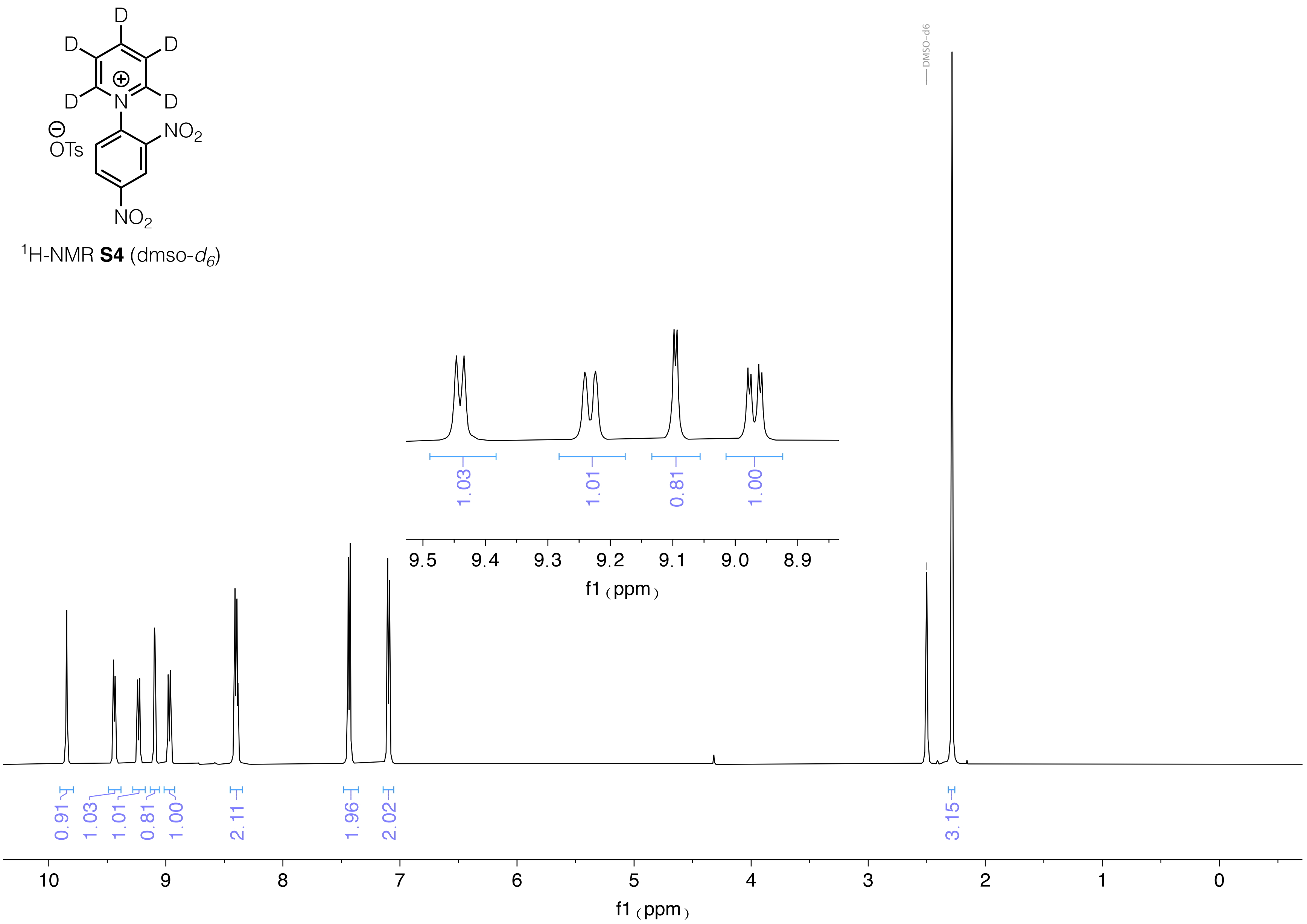

^{1}H-NMR **S4** (dmso-d_6)

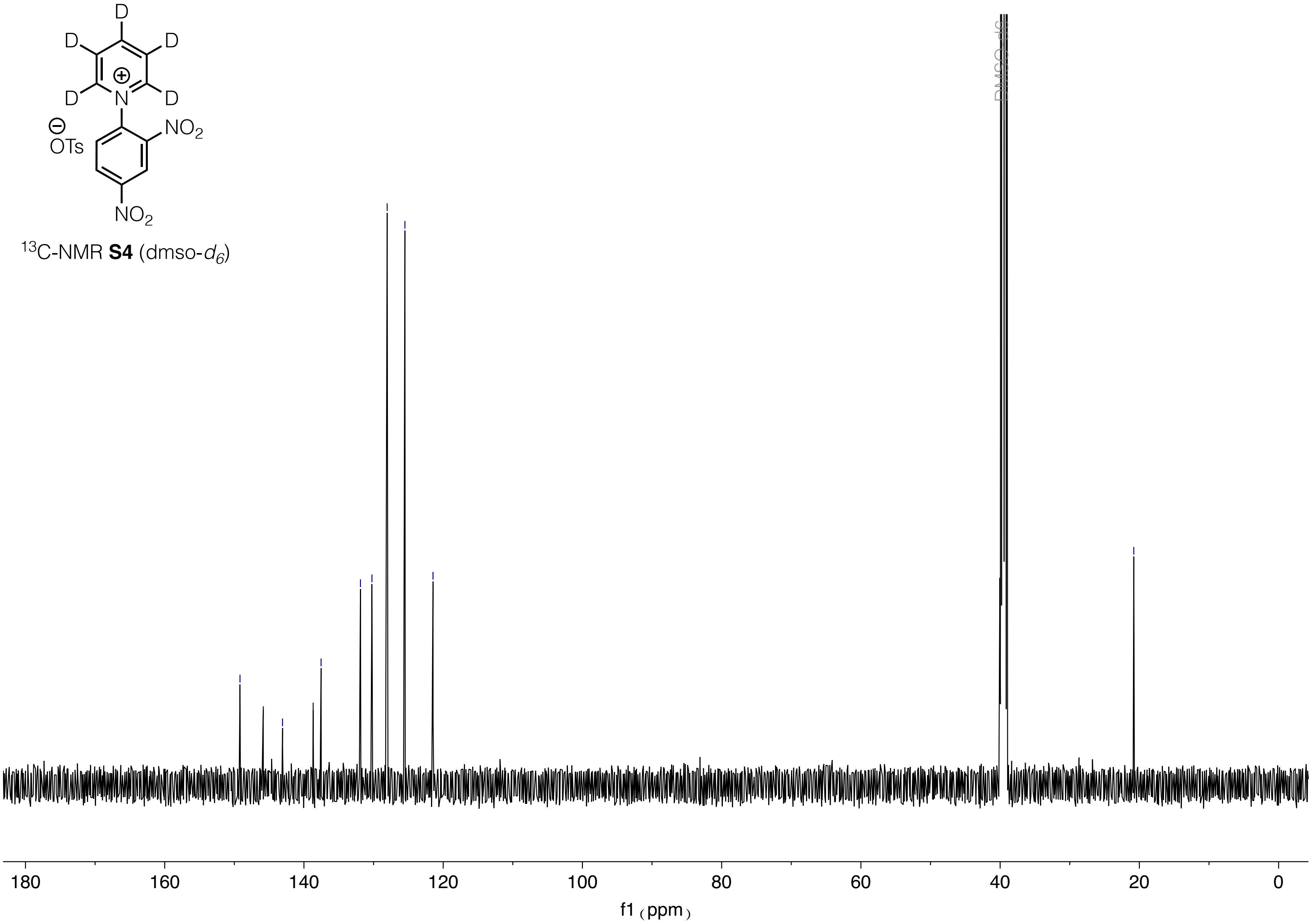

^{13}C-NMR **S4** (dmso-d_6)

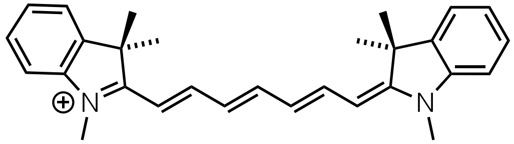

^{1}H-NMR **Cy7** (CD_3OD-d_4)

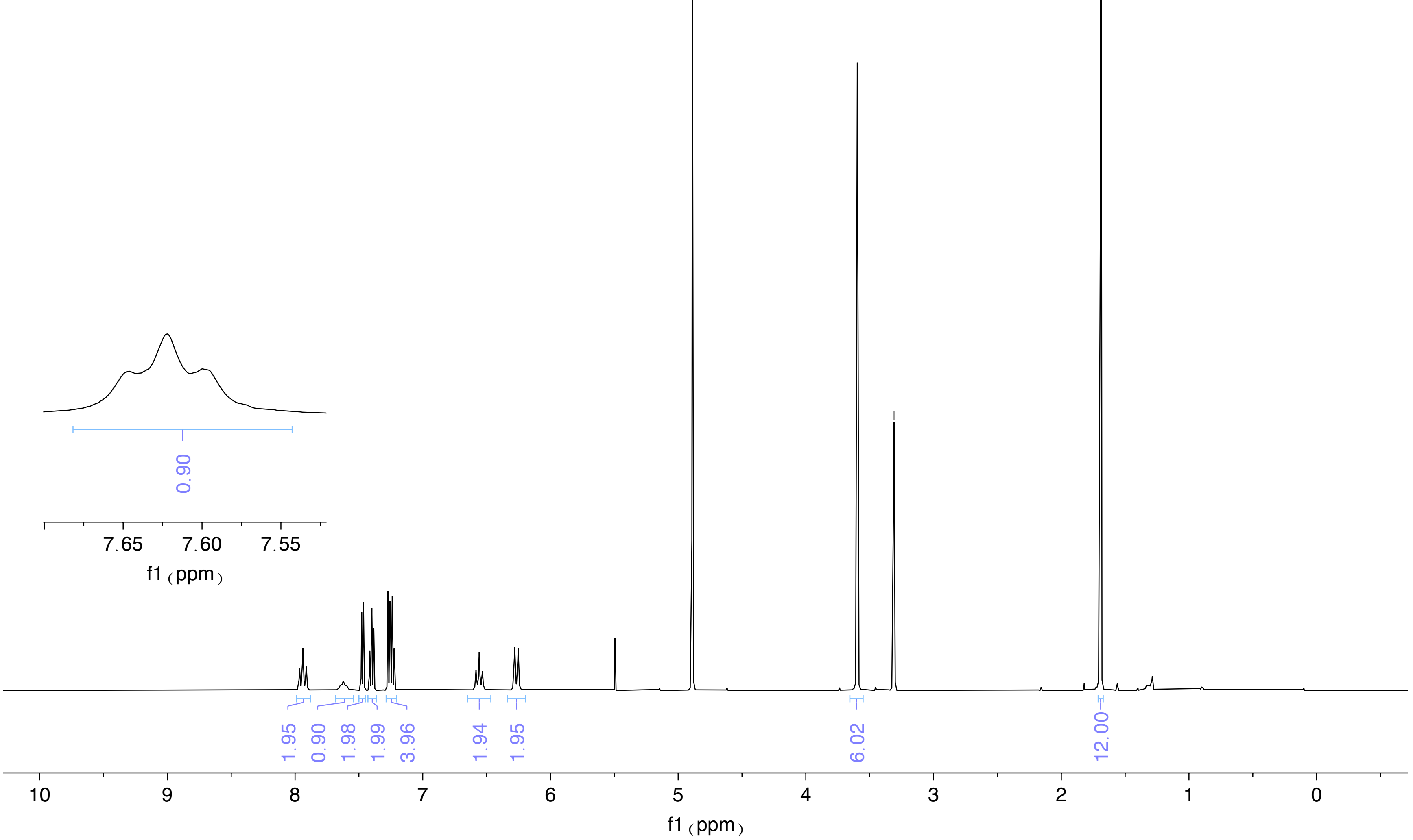

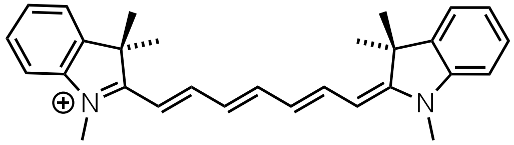

^{13}C-NMR **Cy7** (CD_3OD-d_4)

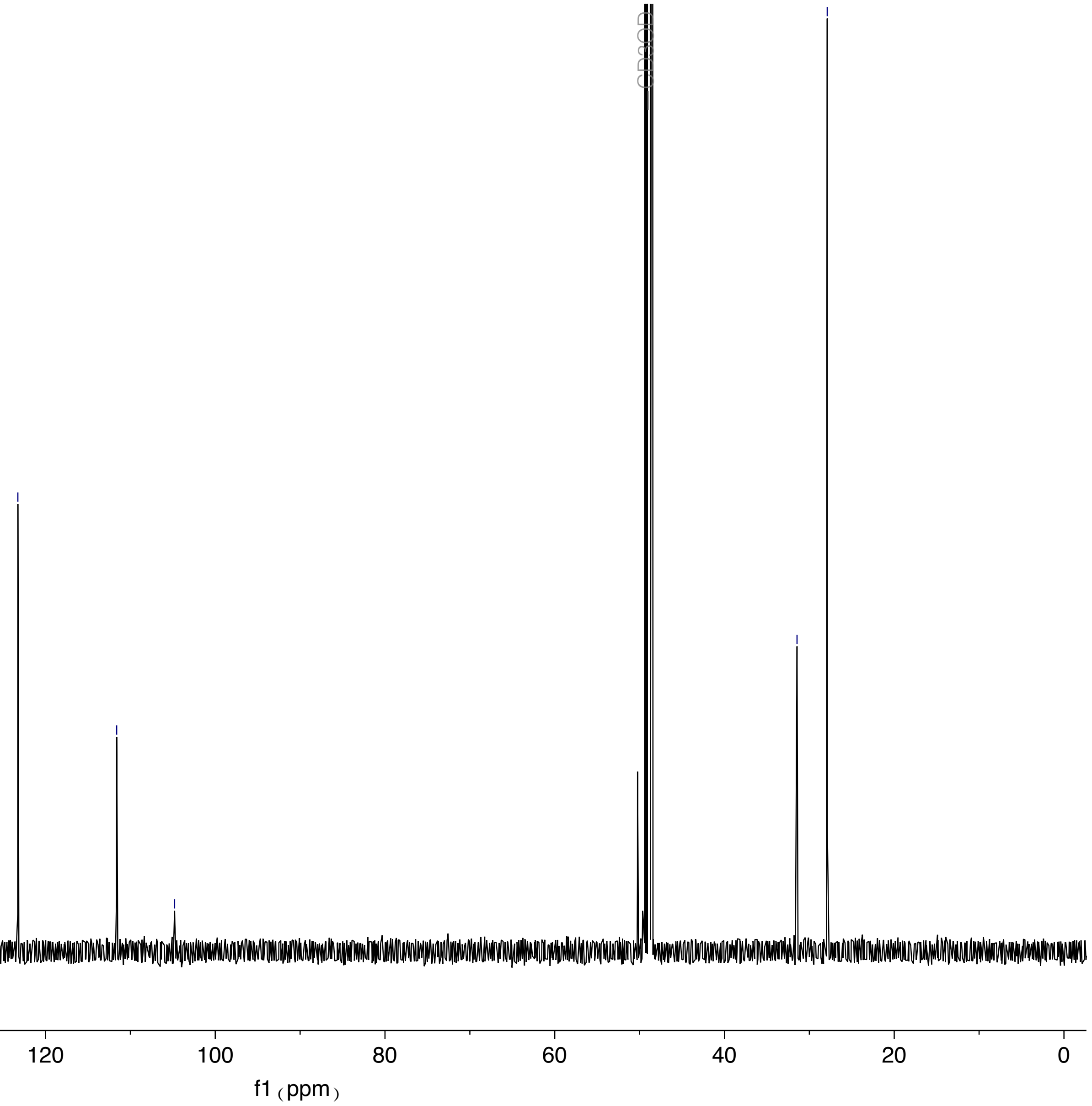

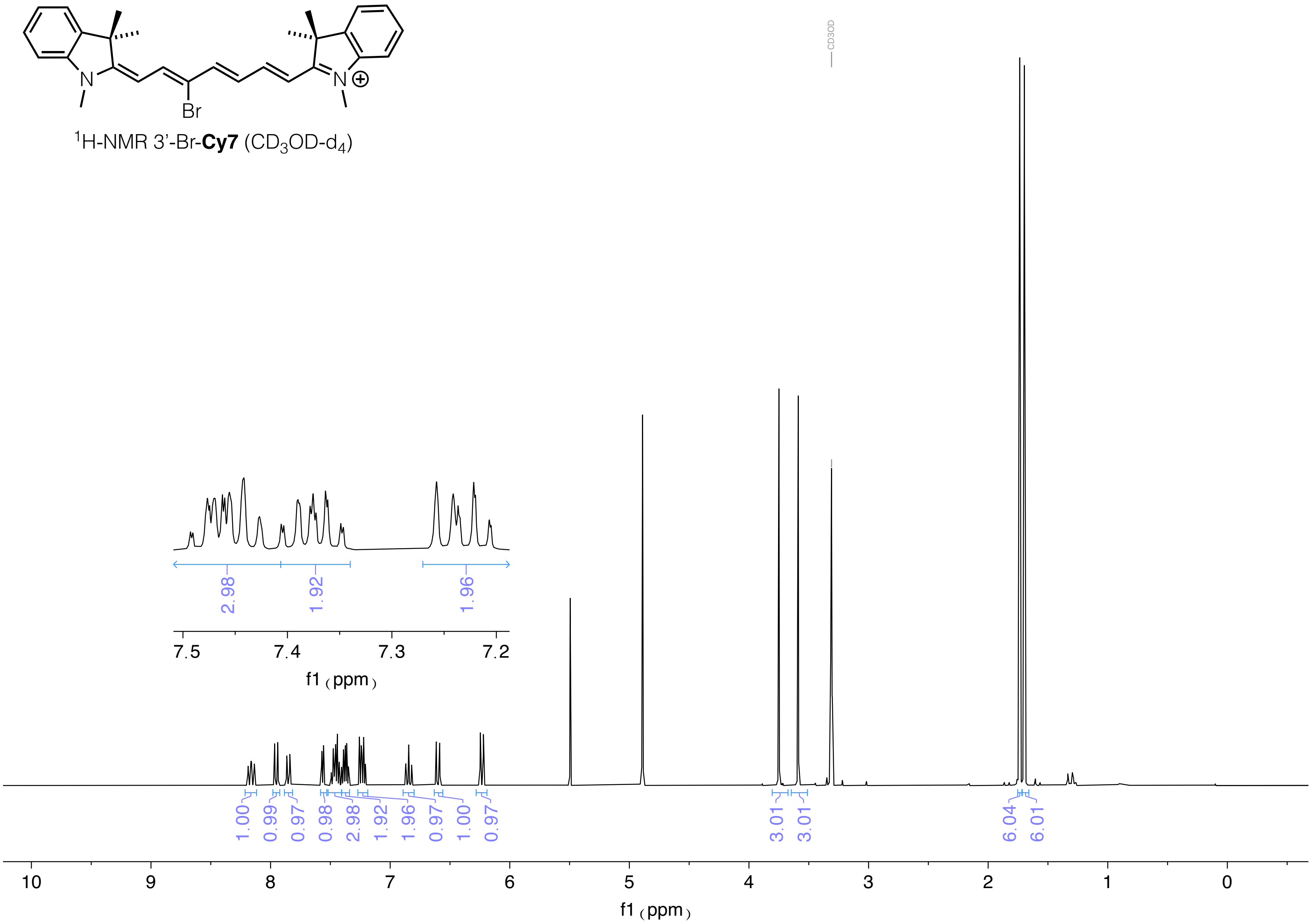

N
N
Br
CD3OD
^{1}H-NMR 3'-Br-**Cy7** (CD3OD-d4)
2.98
1.92
1.96
7.5
7.4
7.3
7.2
f1 (ppm)
1.00
0.99
0.97
0.98
2.98
1.92
1.96
0.97
1.00
0.97
3.01
3.01
6.04
6.01
10
9
8
7
6
5
4
3
2
1
0
f1 (ppm)

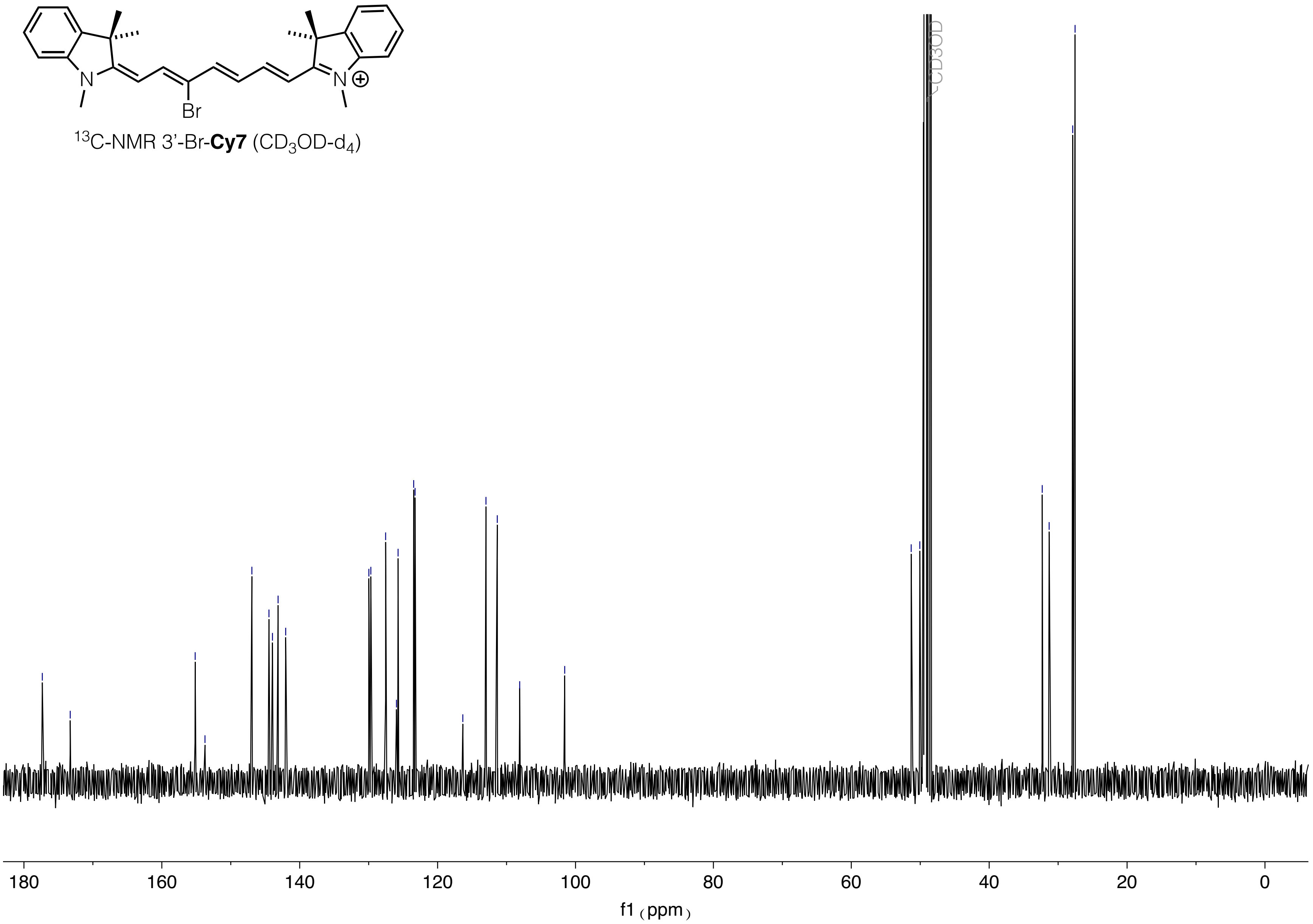

N
N⊕
Br
^{13}C-NMR 3'-Br-**Cy7** (CD_3OD-d_4)
CD3OD
180
160
140
120
100
80
60
40
20
0
f1 (ppm)

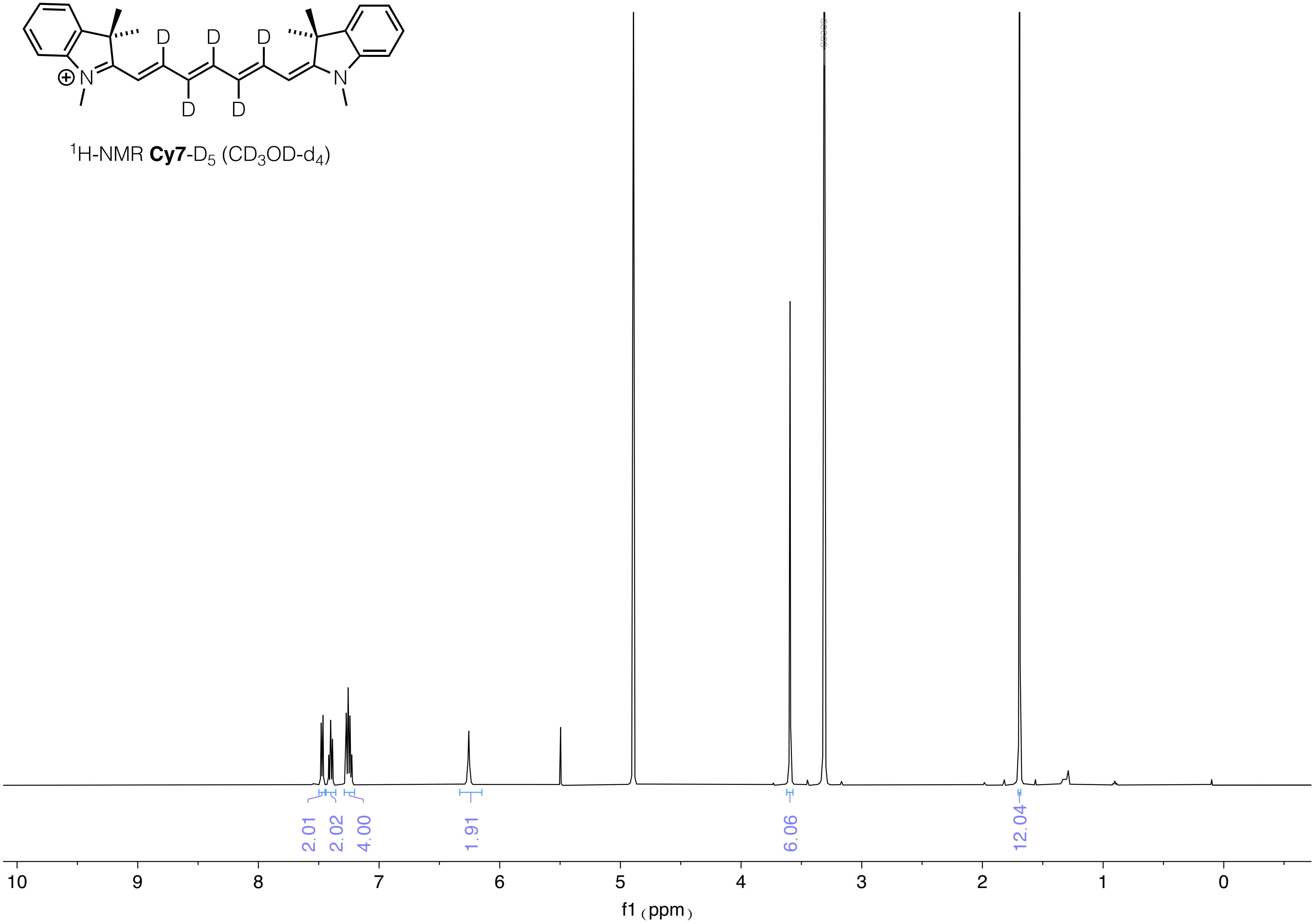

D
D
D
D
D
N
N
^{1}H-NMR **Cy7**-D_5 (CD_3OD-d_4)
2.01
2.02
4.00
1.91
6.06
12.04
10
9
8
7
6
5
4
3
2
1
0
f1 (ppm)

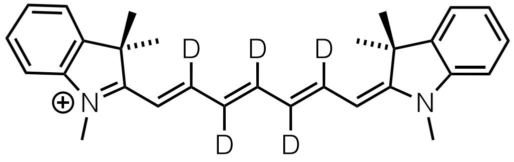

^{13}C-NMR **Cy7**-D$_5$ (CD$_3$OD-d$_4$)

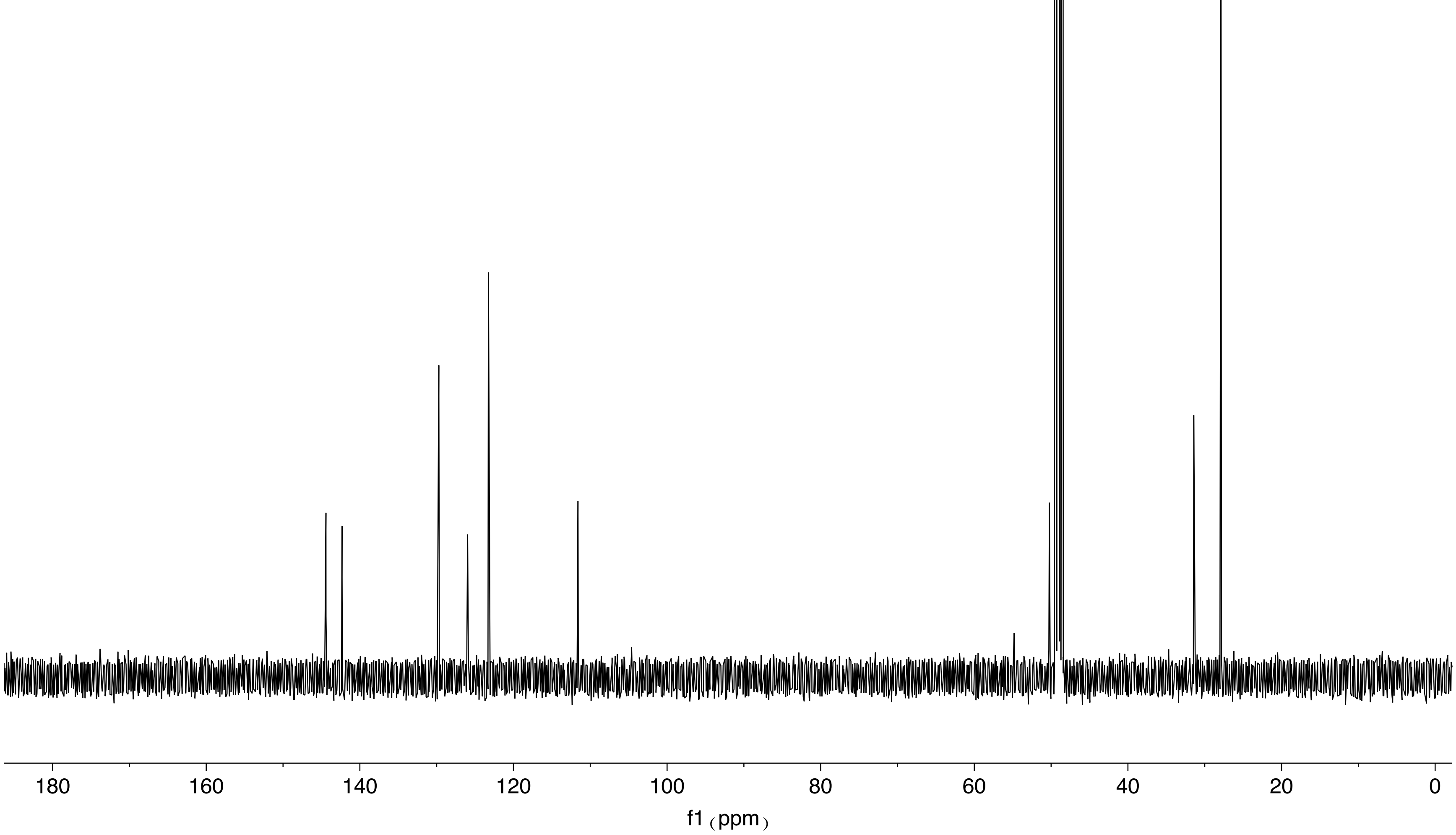

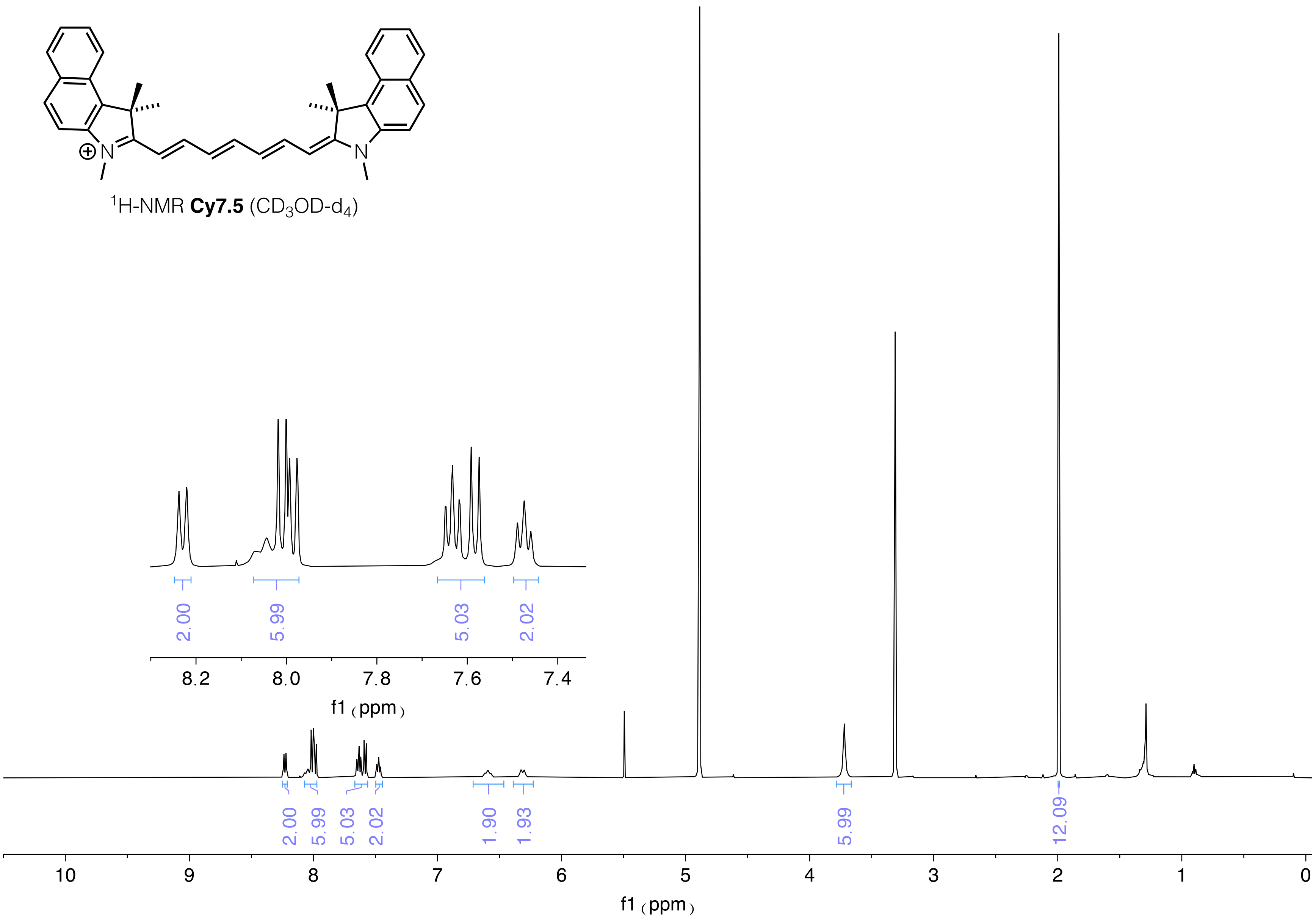

N
N
^{1}H-NMR **Cy7.5** (CD_3OD-d_4)
2.00
5.99
5.03
2.02
8.2
8.0
7.8
7.6
7.4
f1 (ppm)
2.00
5.99
5.03
2.02
1.90
1.93
5.99
12.09
10
9
8
7
6
5
4
3
2
1
0
f1 (ppm)

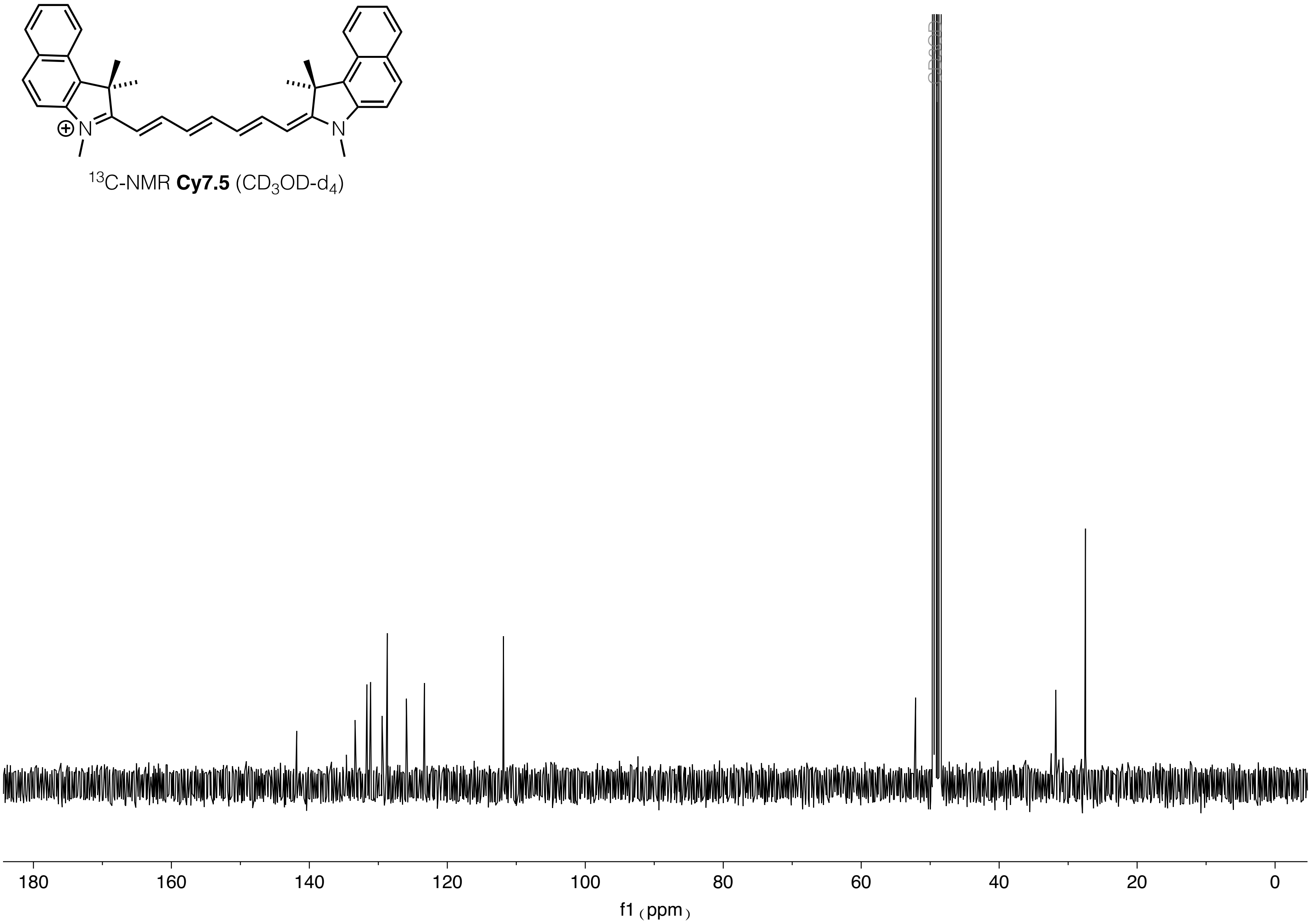

^{13}C-NMR **Cy7.5** (CD_3OD-d_4)

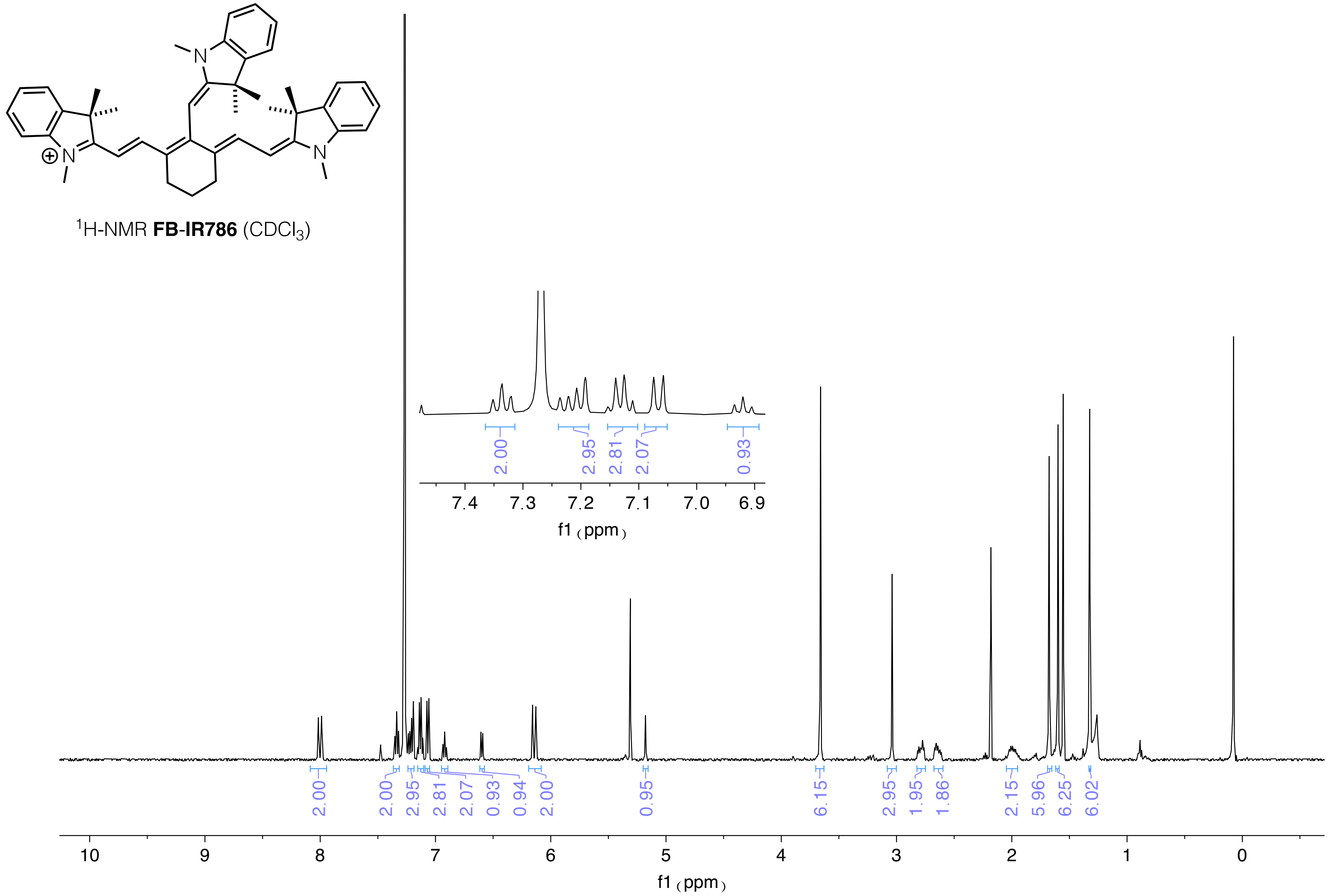

^{1}H-NMR **FB-IR786** ($CDCl_3$)

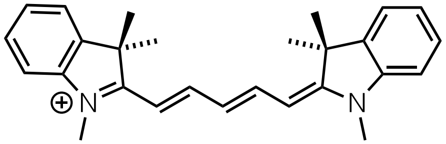

^{1}H-NMR **Cy5** (CD_3OD-d_4)

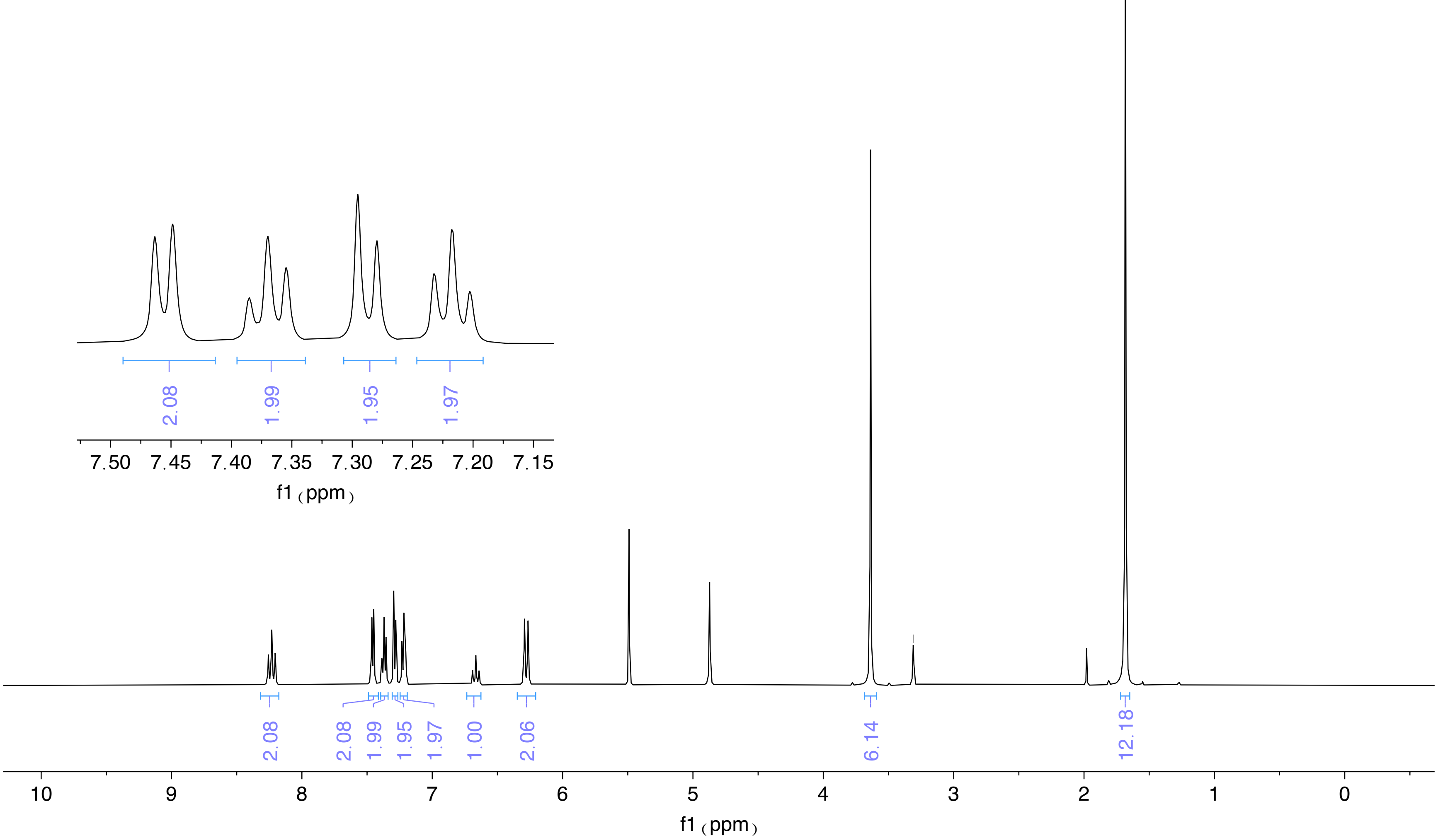

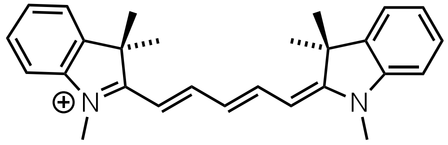

^{13}C-NMR **Cy5** (CD_3OD-d_4)

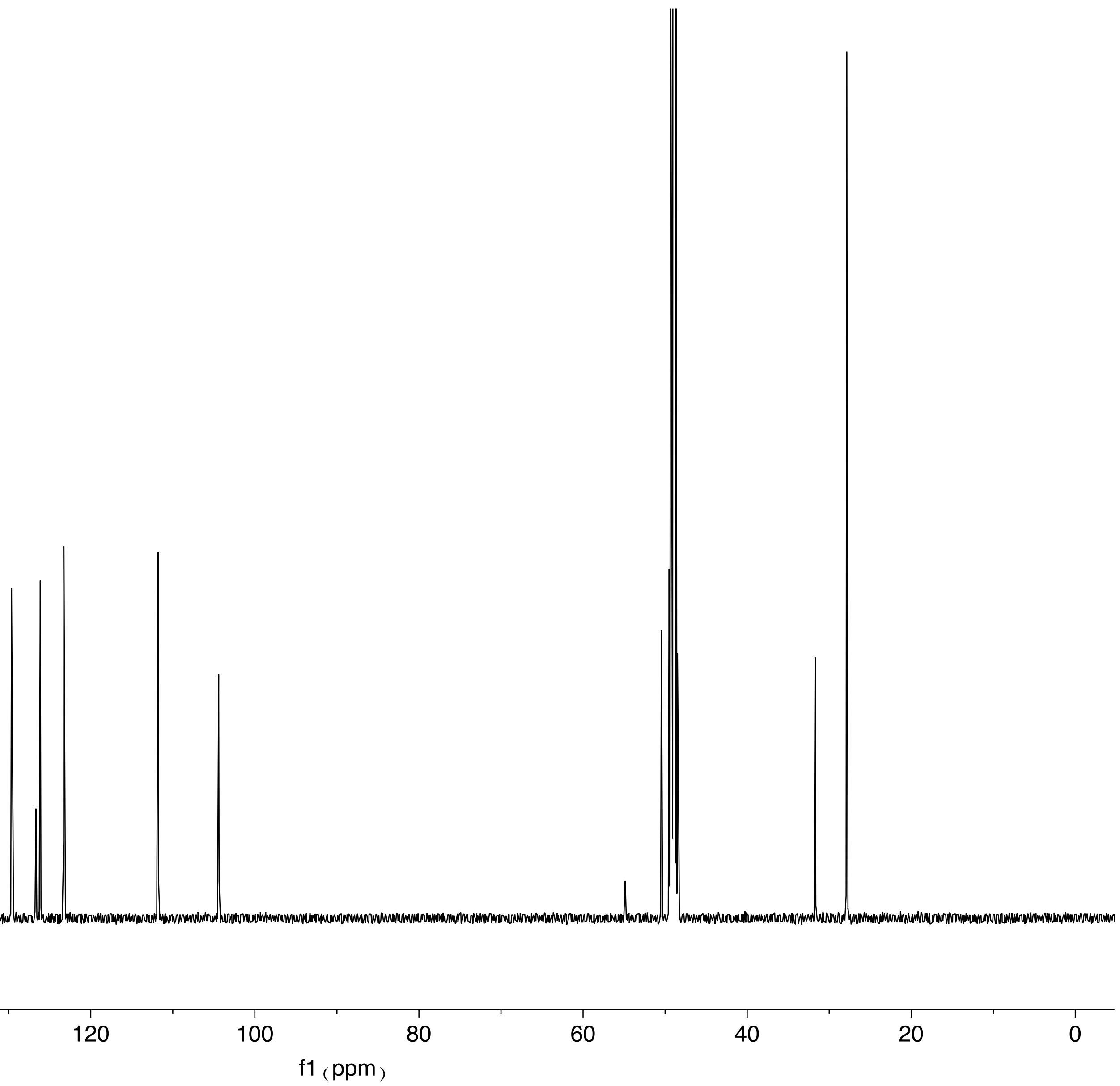

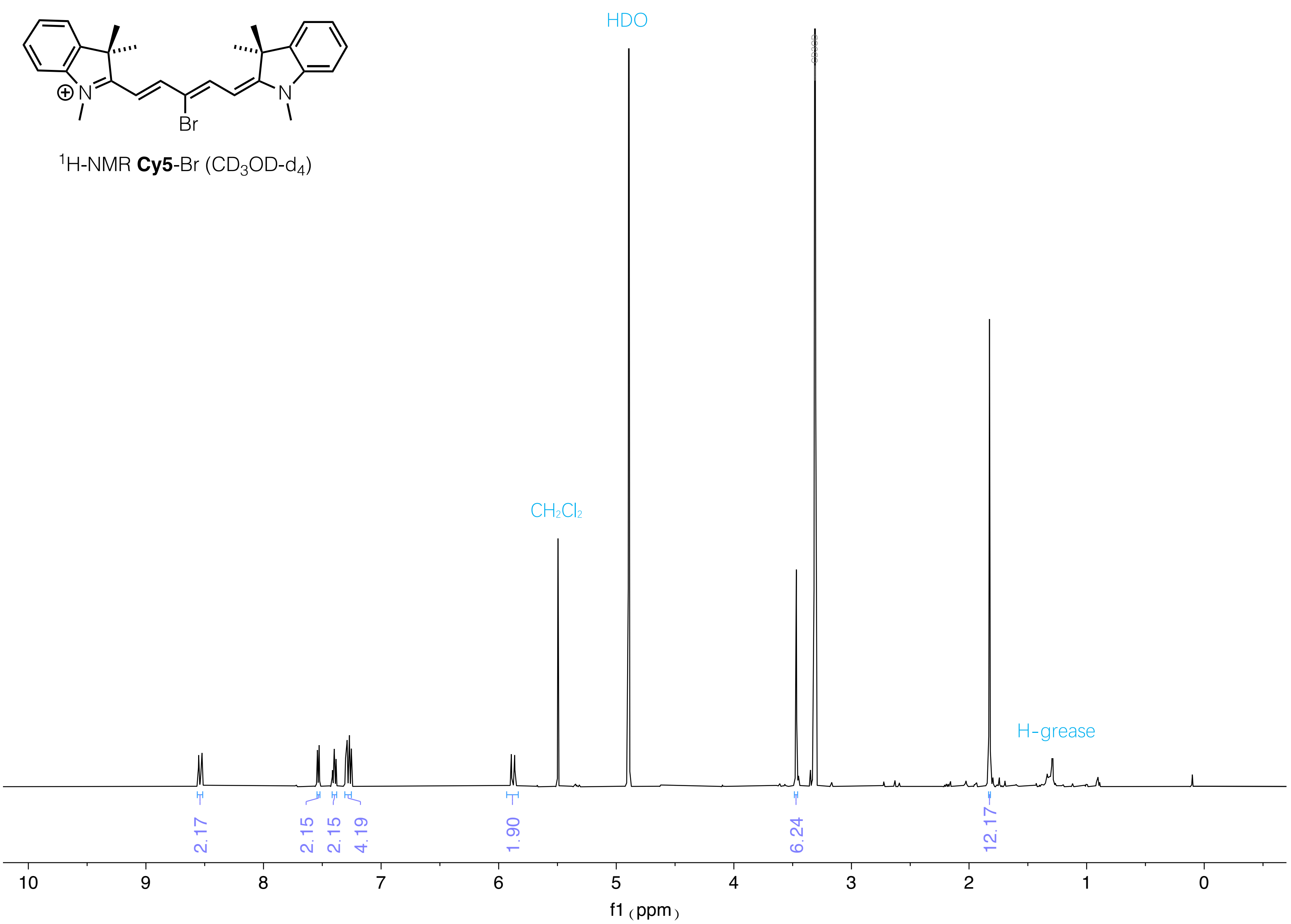

^{1}H-NMR **Cy5**-Br (CD_3OD-d_4)

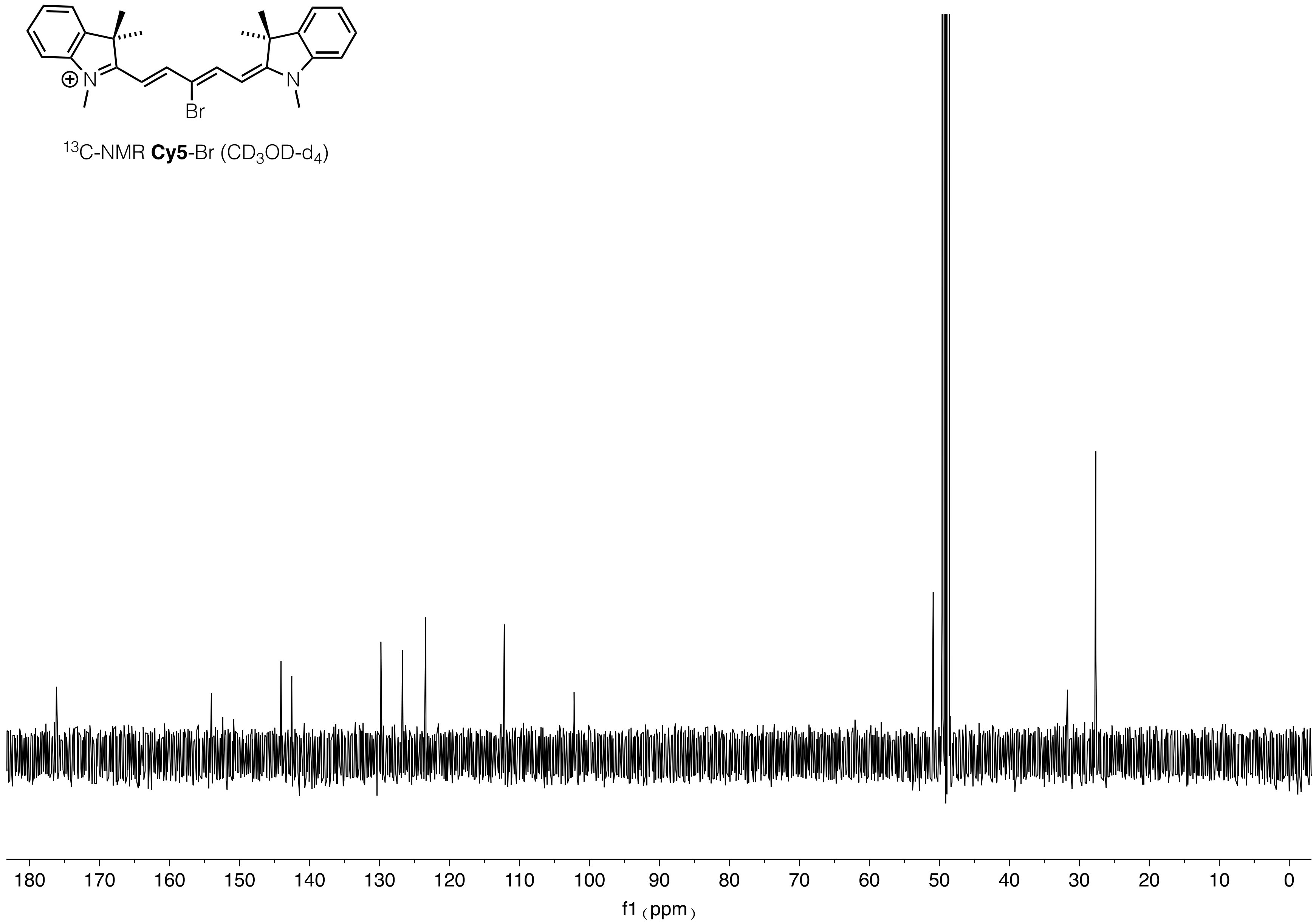
Br
N
N
^{13}C-NMR **Cy5**-Br (CD_3OD-d_4)
180 170 160 150 140 130 120 110 100 90 80 70 60 50 40 30 20 10 0
f1 (ppm)

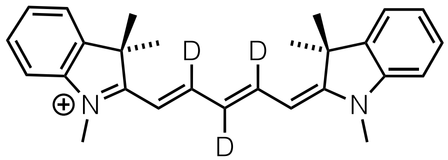

^{1}H-NMR **Cy5**-D_3 (CD_3OD-d_4)

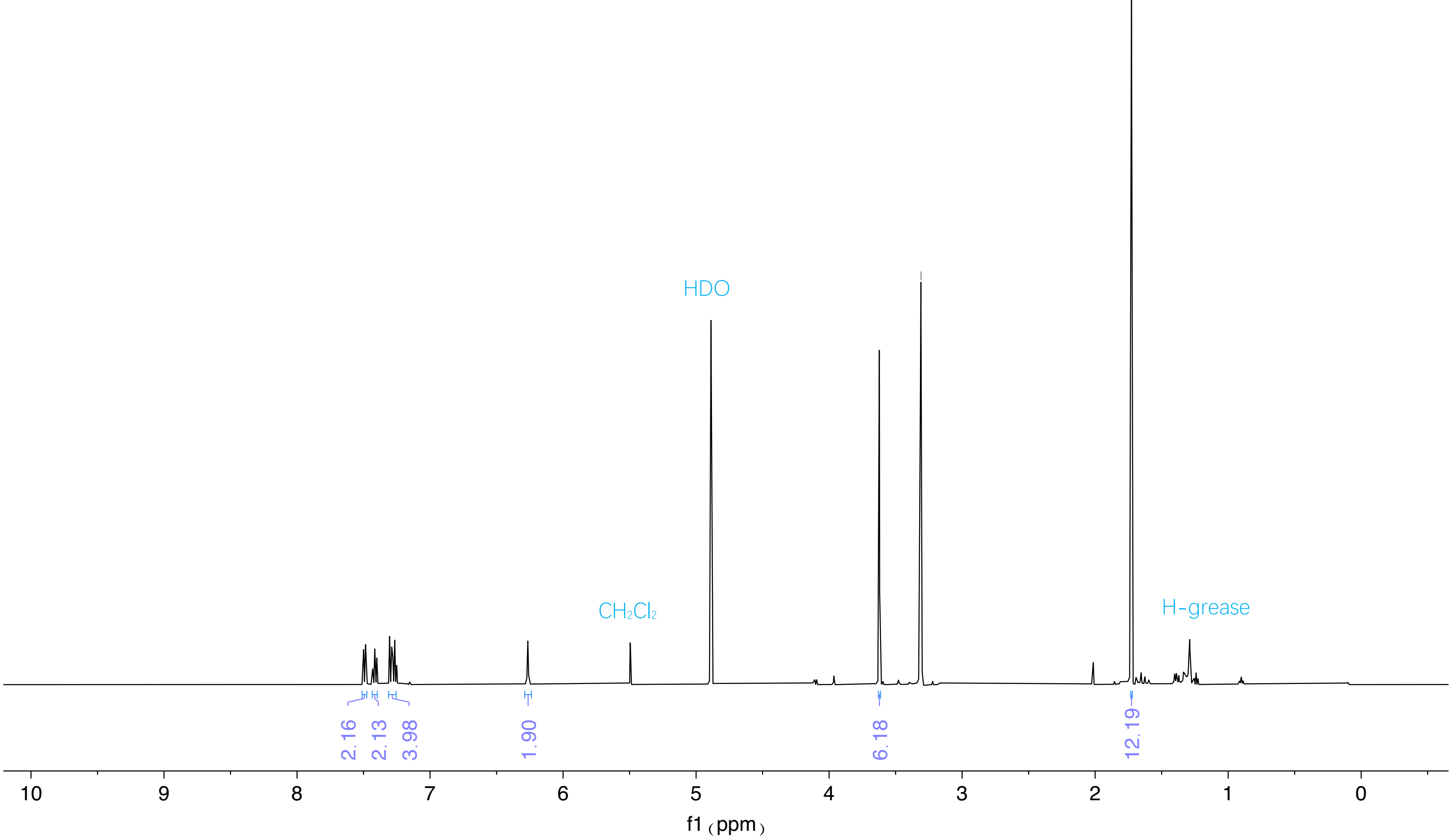

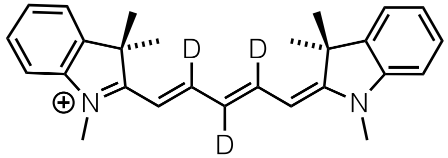

^{13}C-NMR **Cy5**-D_3 (CD_3OD-d_4)

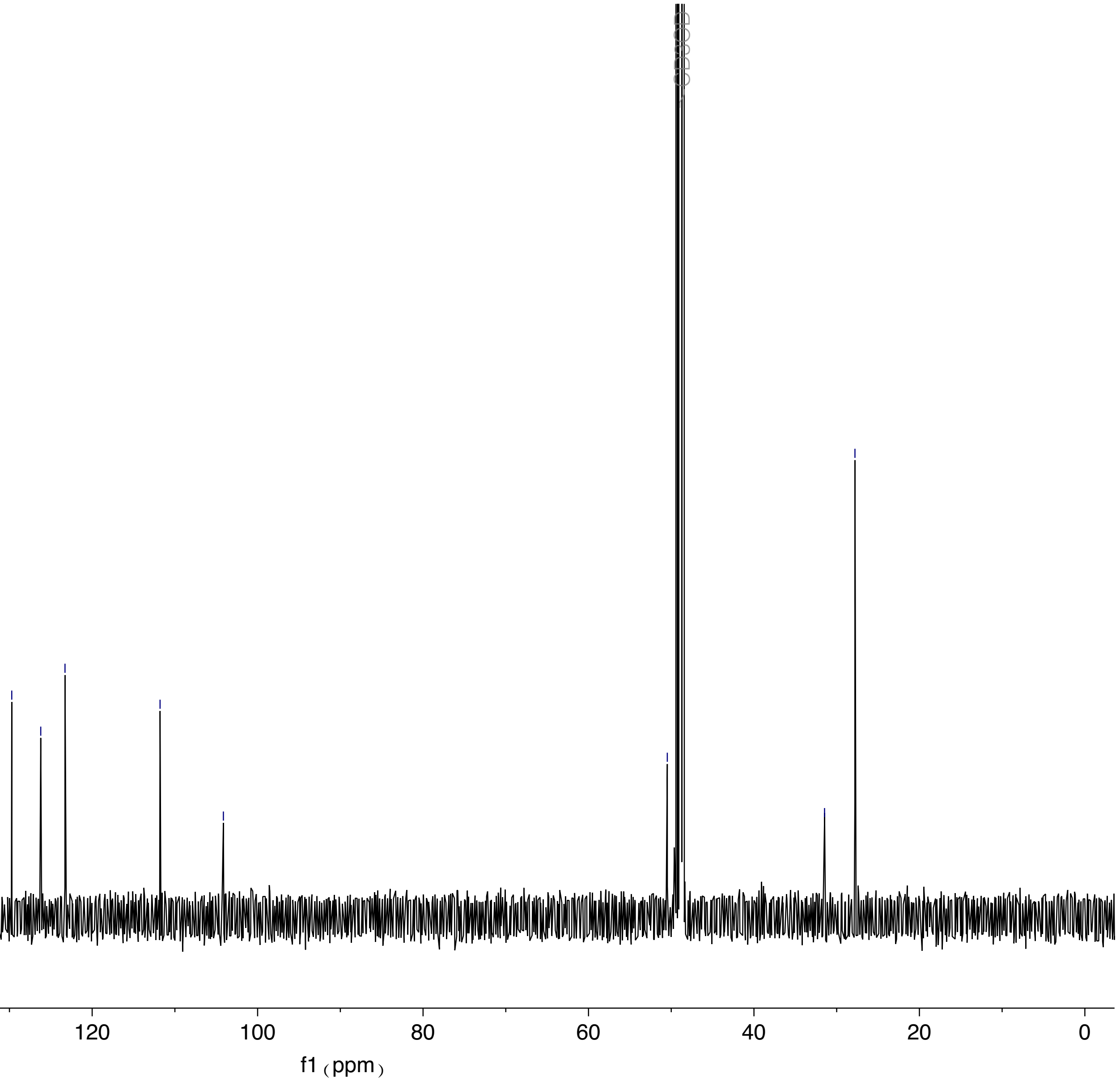

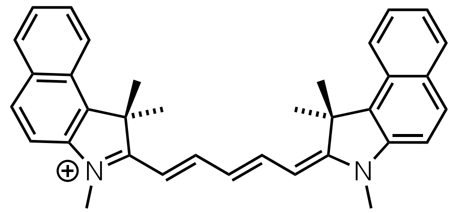

^{1}H-NMR **Cy5.5** (CD_3OD-d_4)

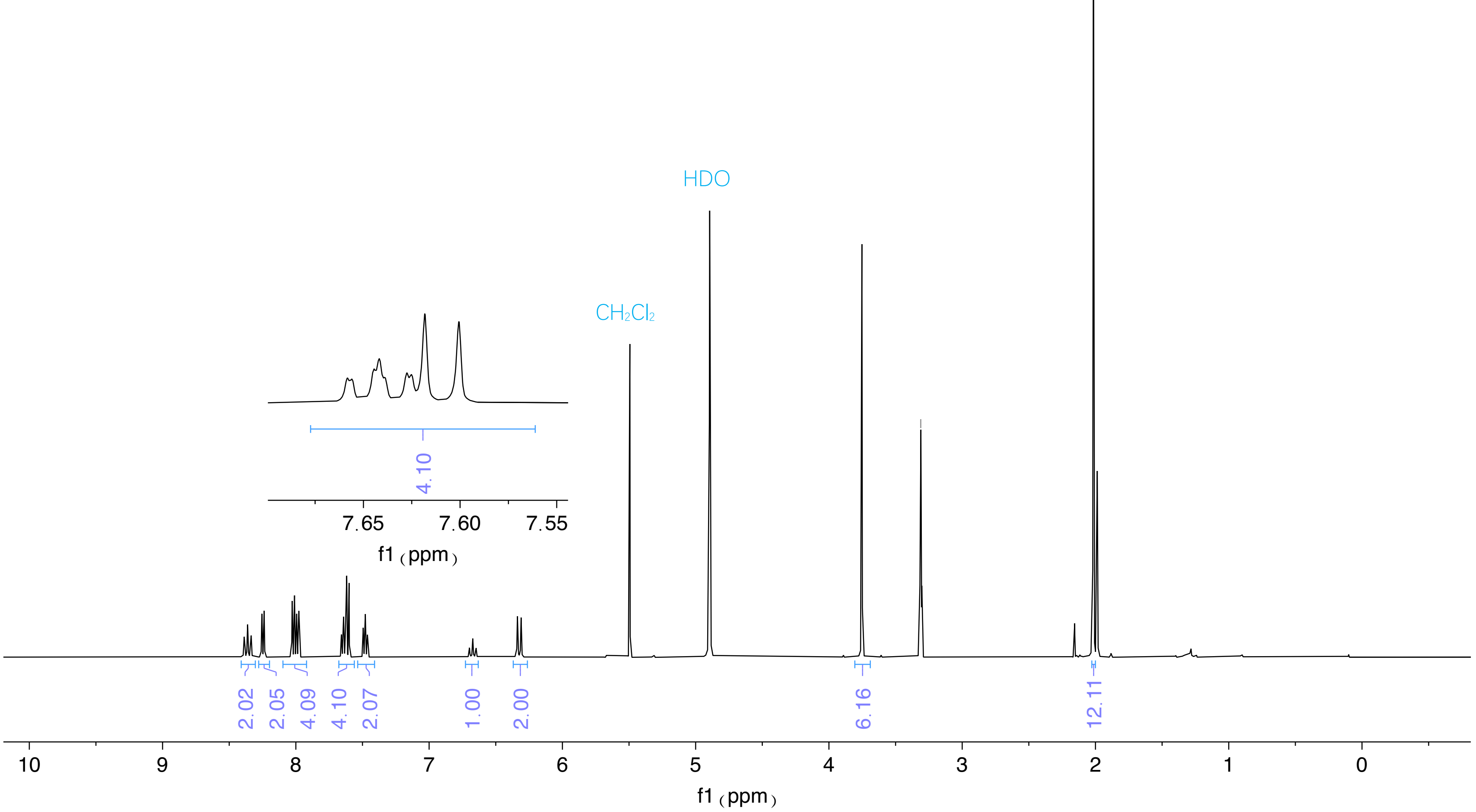

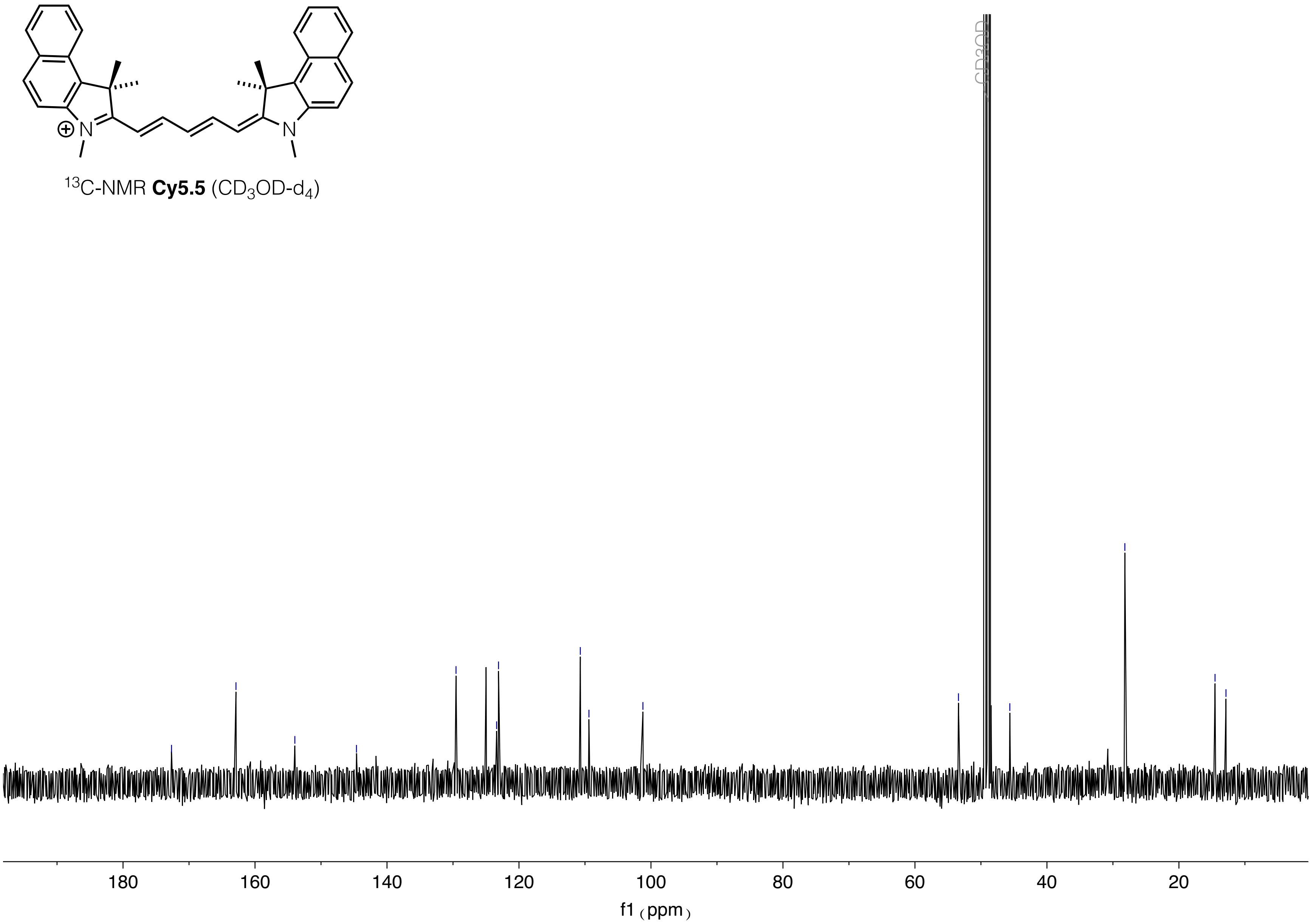
^{13}C-NMR **Cy5.5** (CD_3OD-d_4)
CD3OD
180
160
140
120
100
80
60
40
20
f1 (ppm)

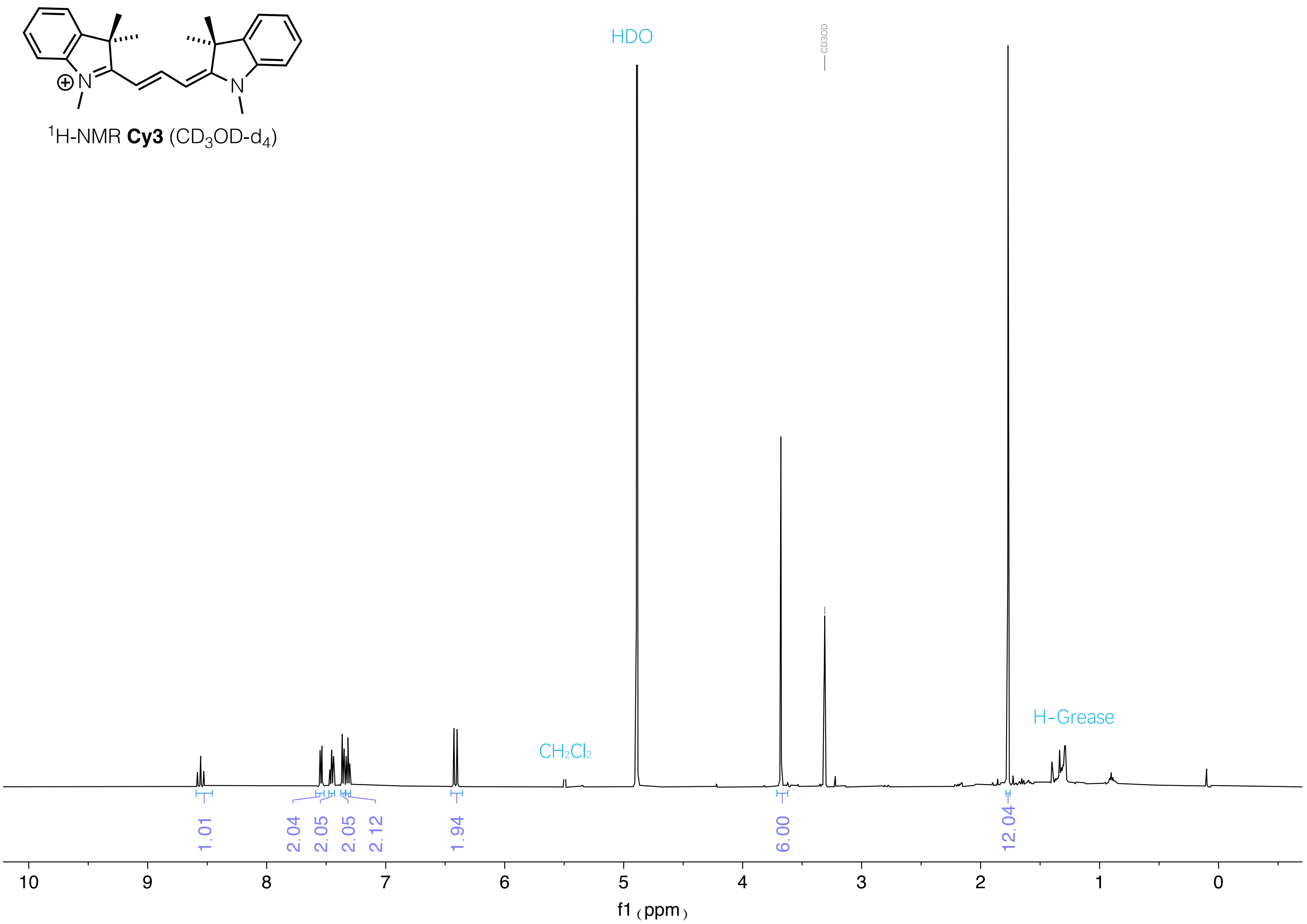

¹H-NMR Cy3 (CD3OD-d4)
HDO
CD3OD
CH2Cl2
H-Grease
1.01
2.04
2.05
2.05
2.12
1.94
6.00
12.04
10
9
8
7
6
5
4
3
2
1
0
f1 (ppm)

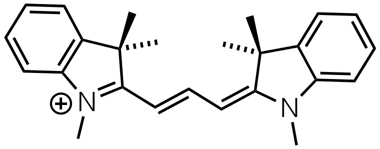

^{13}C-NMR **Cy3** (CD_3OD-d_4)

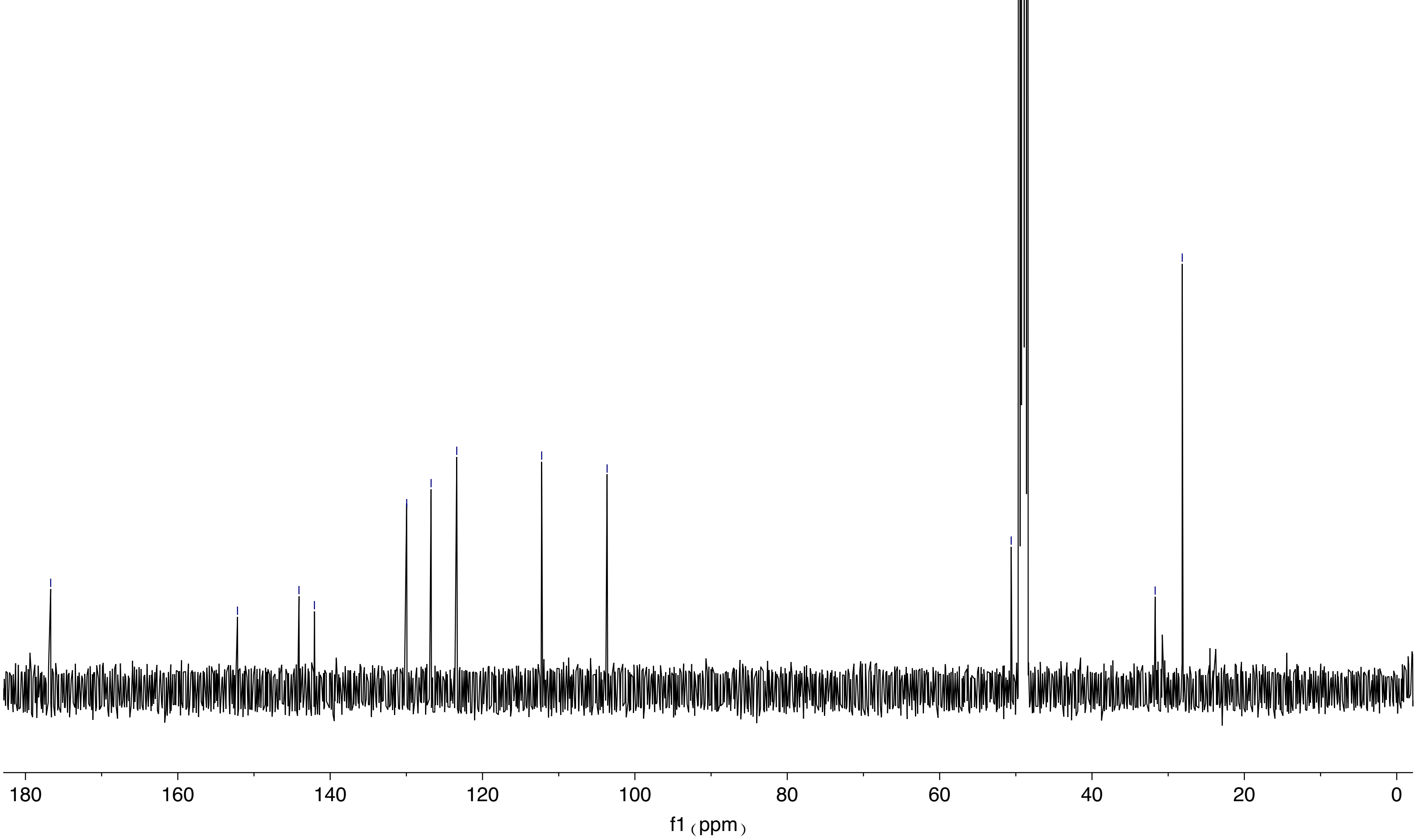

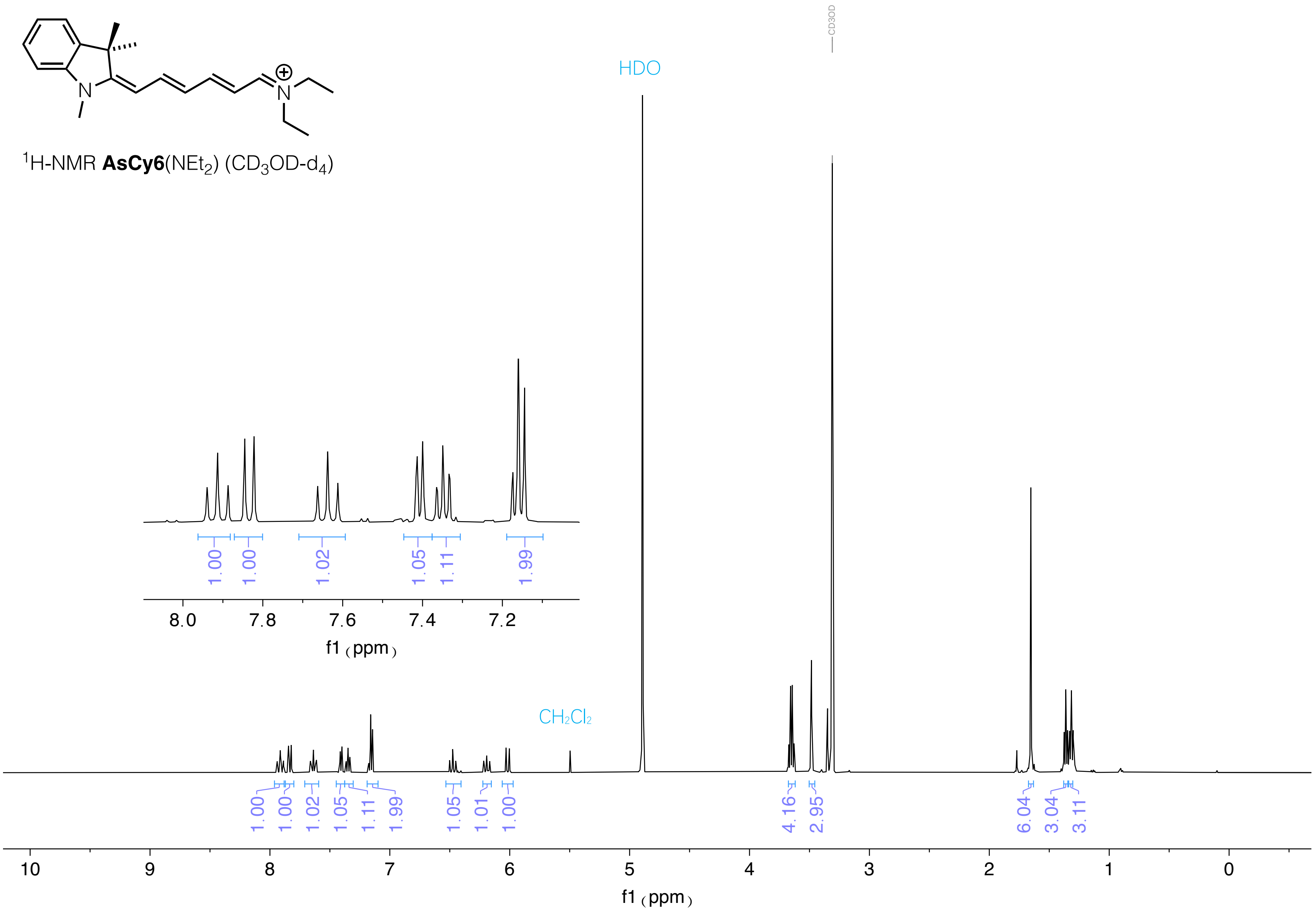

N
N
¹H-NMR AsCy6(NEt₂) (CD₃OD-d₄)
HDO
CD3OD
CH₂Cl₂
1.00
1.00
1.02
1.05
1.11
1.99
8.0
7.8
7.6
7.4
7.2
f1 (ppm)
1.00
1.00
1.02
1.05
1.11
1.99
1.05
1.01
1.00
4.16
2.95
6.04
3.04
3.11
10
9
8
7
6
5
4
3
2
1
0
f1 (ppm)

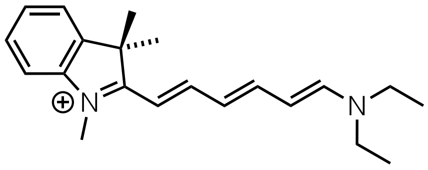

^{13}C-NMR **AsCy6**-(NEt$_2$) (CD$_3$OD-d$_4$)

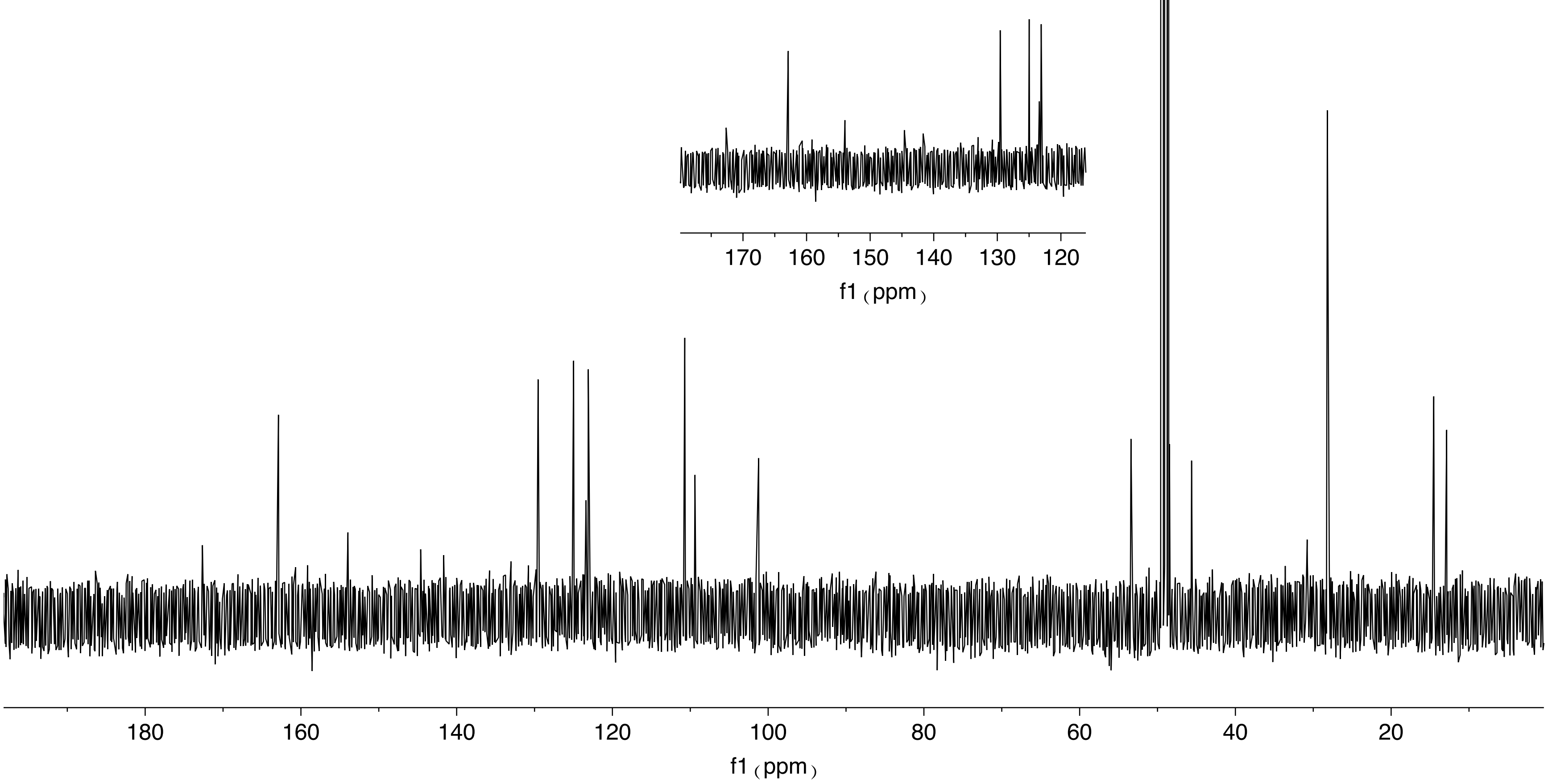

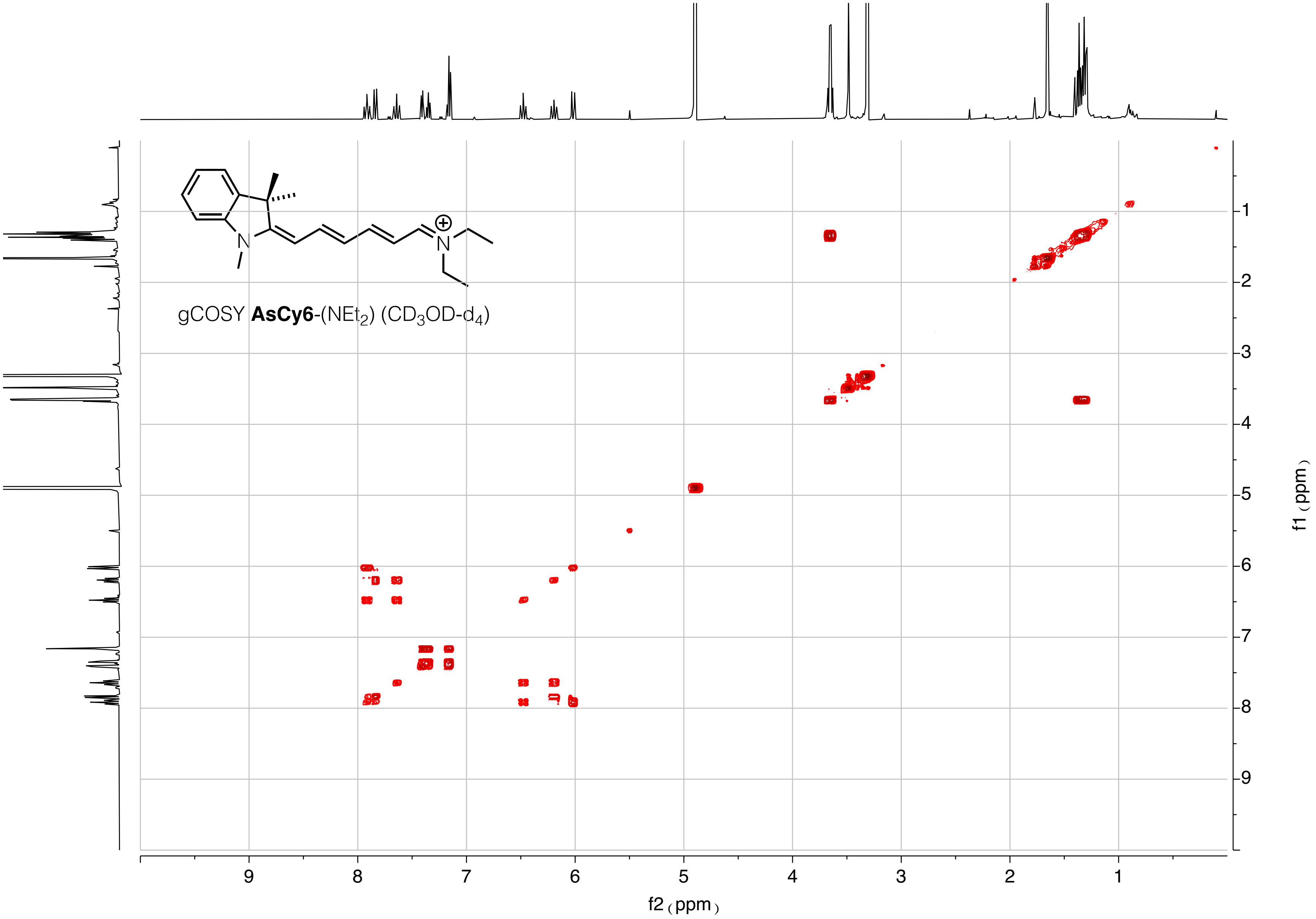
gCOSY AsCy6-(NEt2) (CD3OD-d4)
N
N
f2 (ppm)
f1 (ppm)
9
8
7
6
5
4
3
2
1

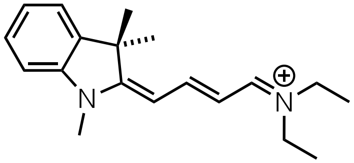

^{1}H-NMR **AsCy4**-(NEt_2) (CD_3OD-d_4)

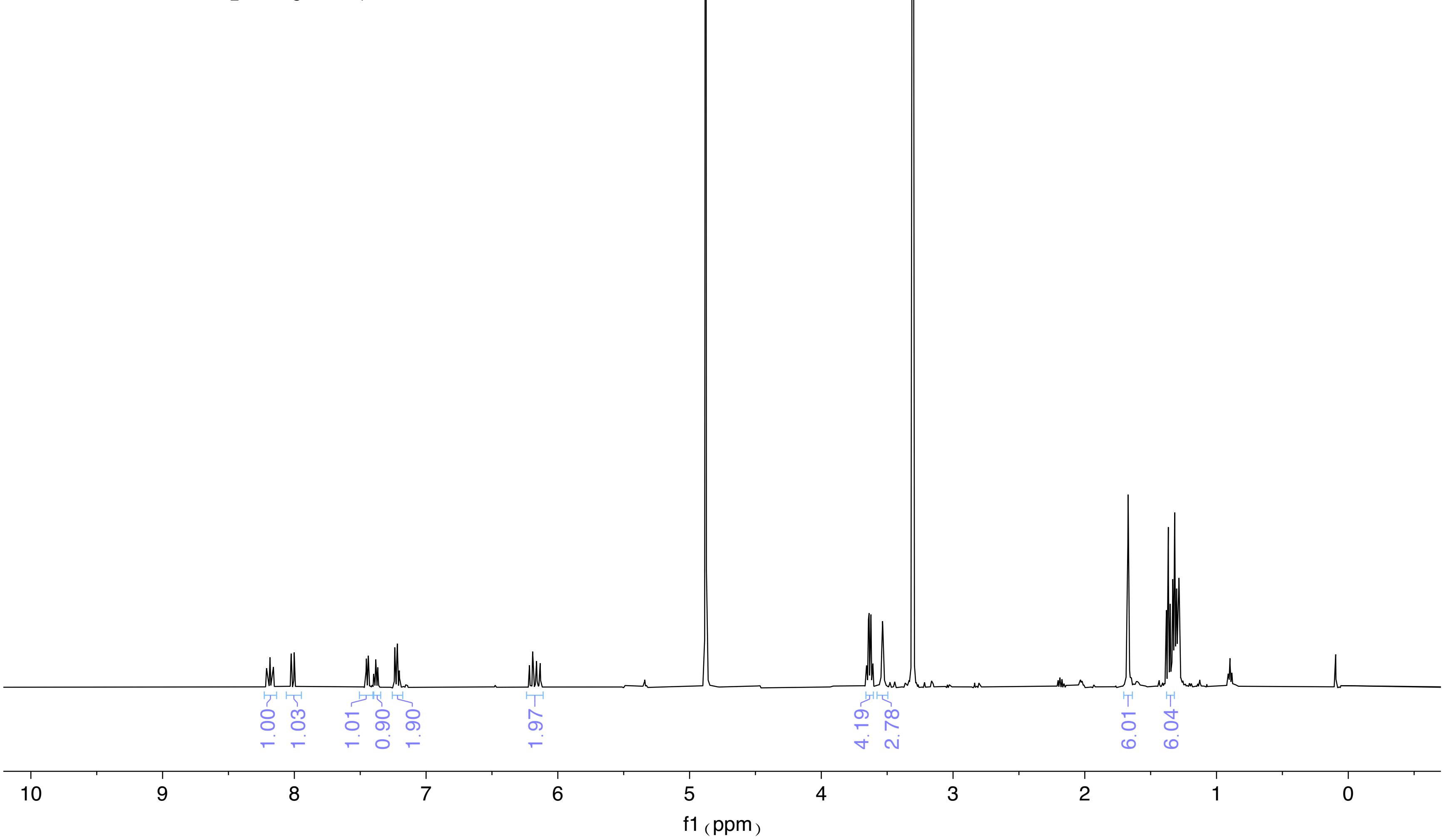

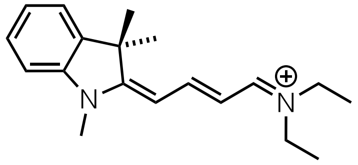

^{13}C-NMR **AsCy4**-(NEt_2) (CD_3OD-d_4)

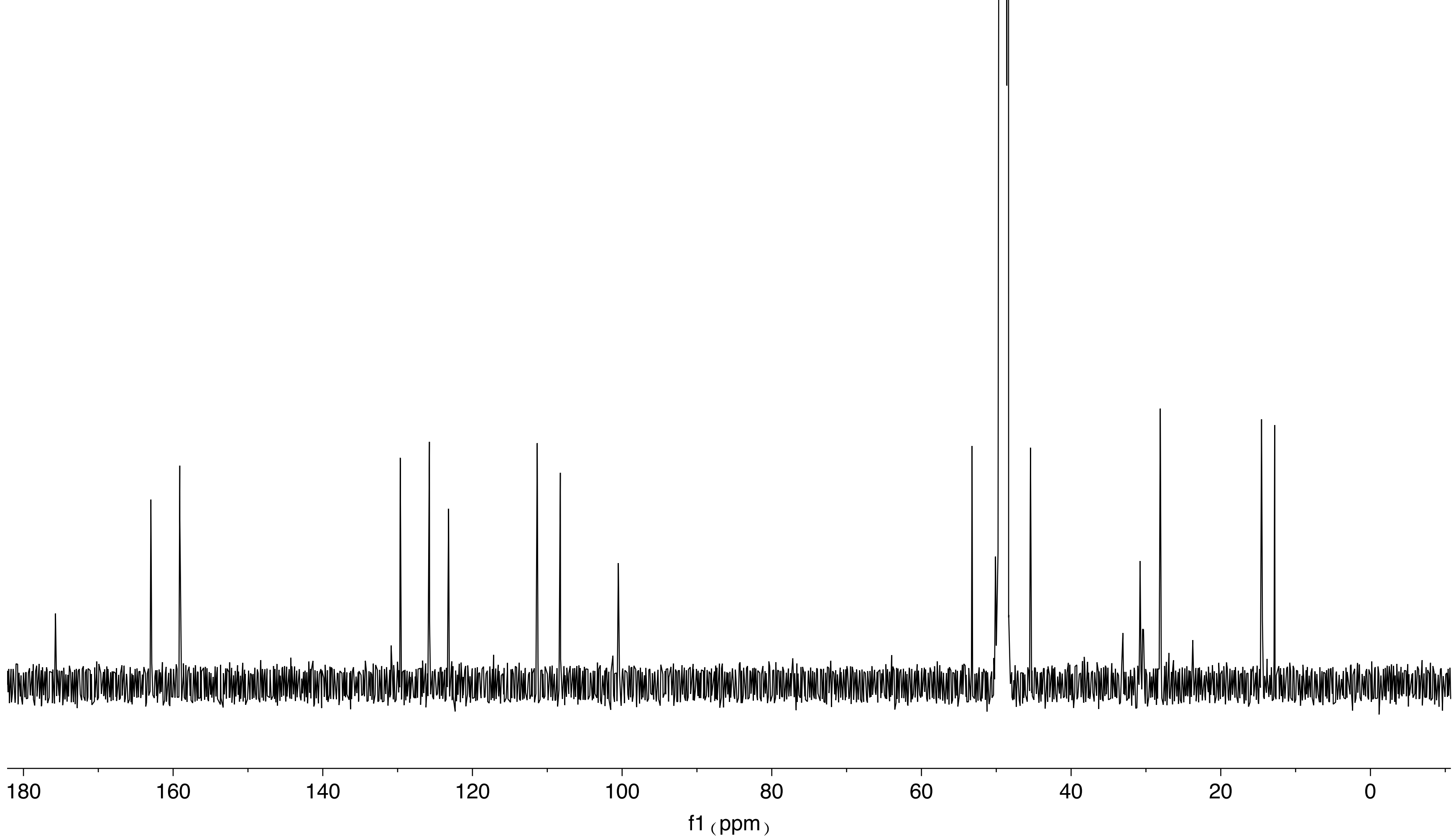

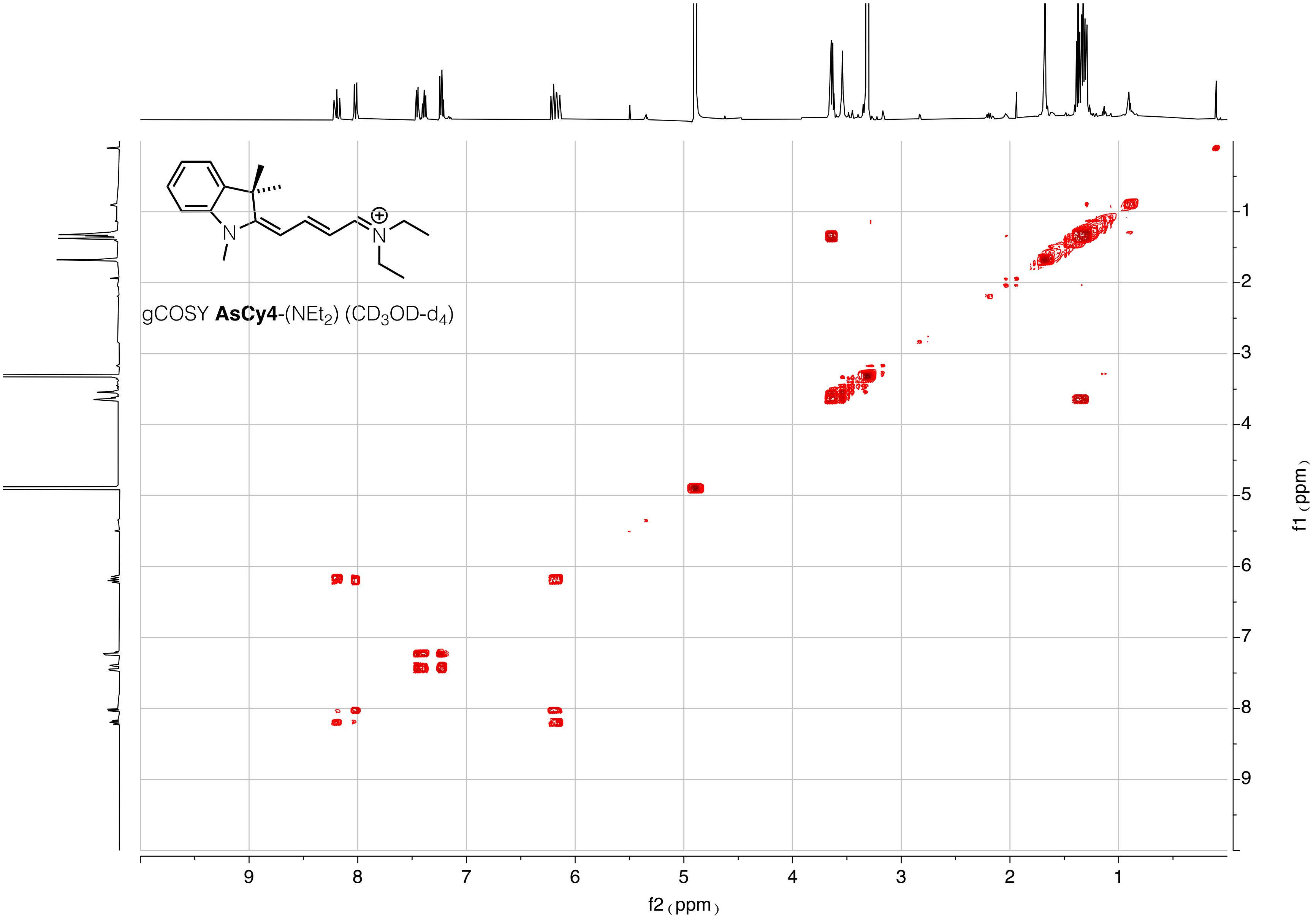

gCOSY **AsCy4**-(NEt2) (CD3OD-d4)
f2 (ppm)
f1 (ppm)

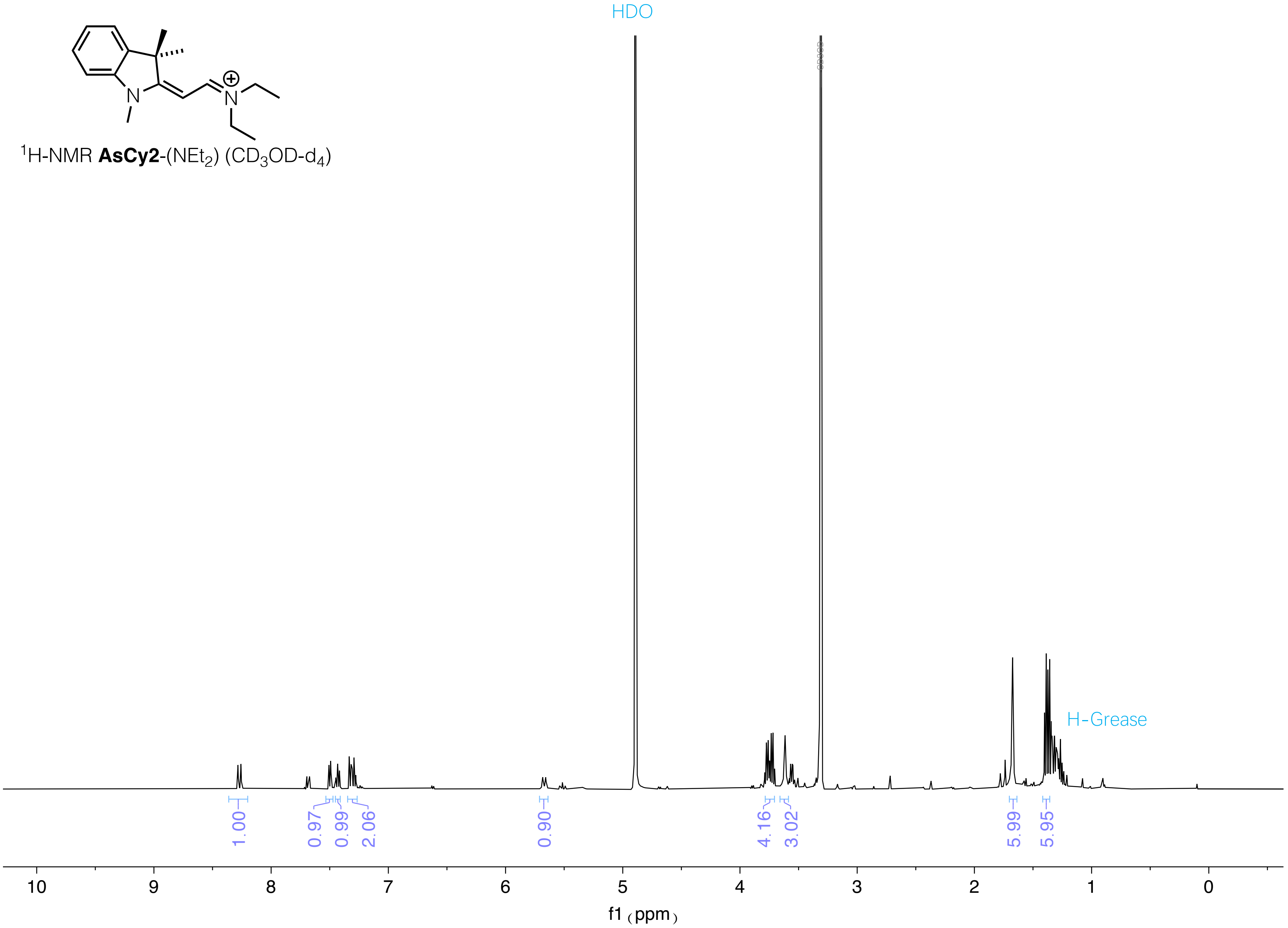

N
N
1H-NMR AsCy2-(NEt2) (CD3OD-d4)
HDO
H-Grease
1.00
0.97
0.99
2.06
0.90
4.16
3.02
5.99
5.95
10
9
8
7
6
5
4
3
2
1
0
f1 (ppm)

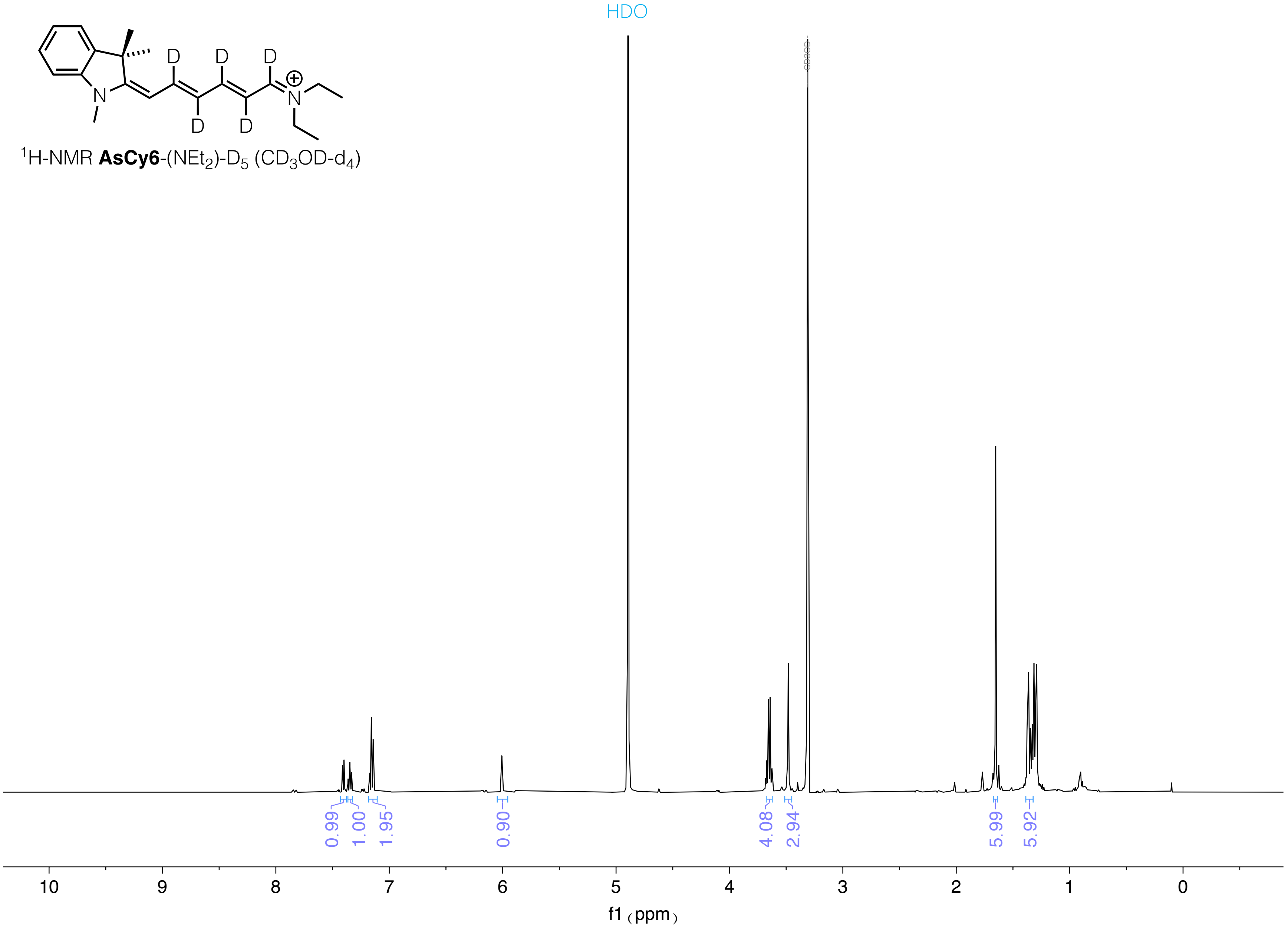
D
D
D
D
D
N
N
^{1}H-NMR **AsCy6**-(NEt$_2$)-D$_5$ (CD$_3$OD-d$_4$)
HDO
0.99
1.00
1.95
0.90
4.08
2.94
5.99
5.92
10
9
8
7
6
5
4
3
2
1
0
f1 (ppm)

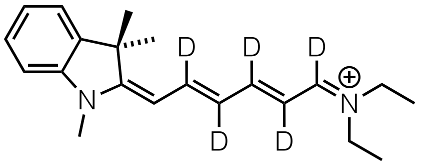

^{13}C-NMR **AsCy6**-(NEt_2)-D_5 (CD_3OD-d_4)

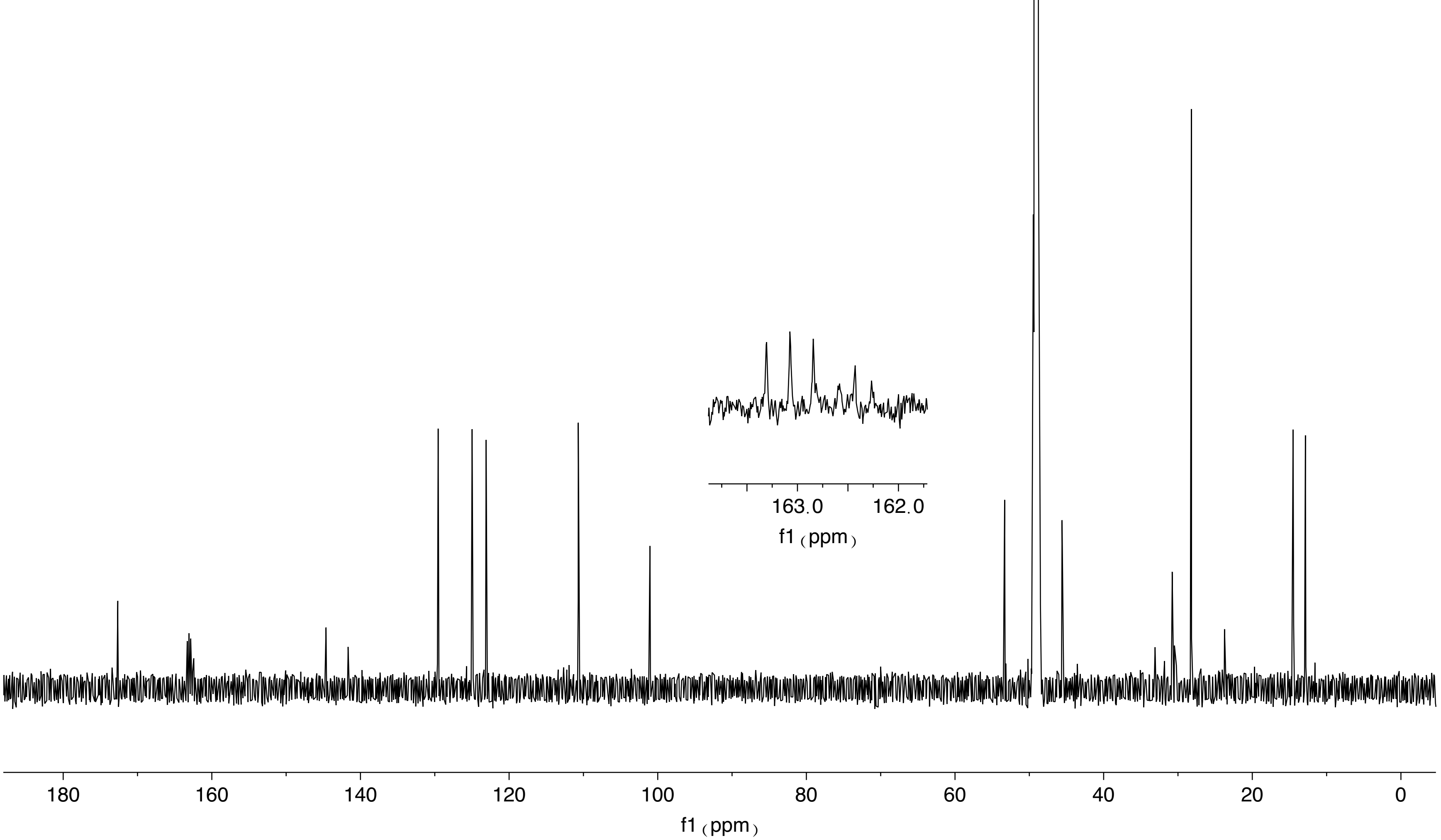

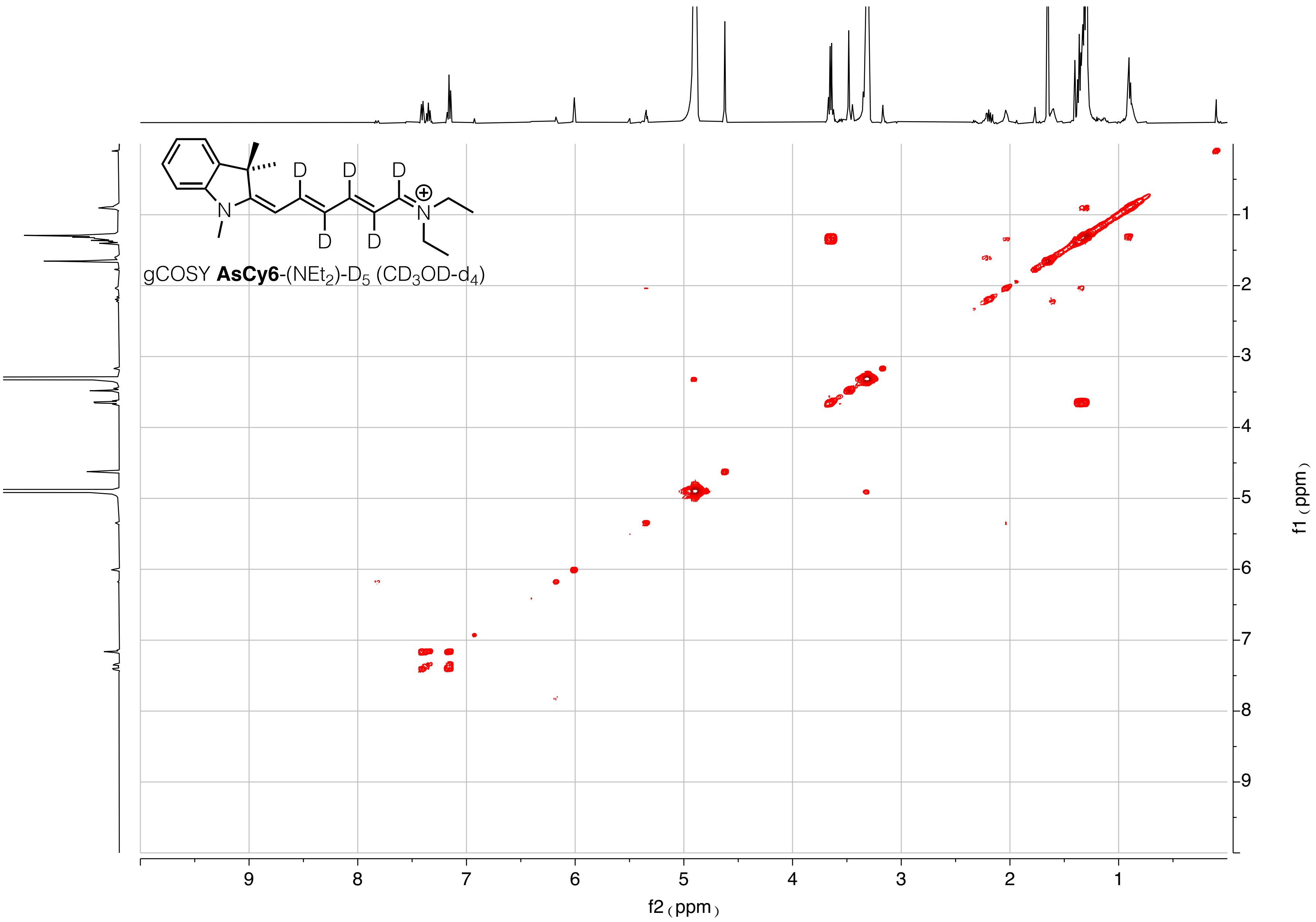

D
D
D
N
N
D
D
gCOSY **AsCy6**-(NEt2)-D5 (CD3OD-d4)
9
8
7
6
5
4
3
2
1
f2 (ppm)
f1 (ppm)

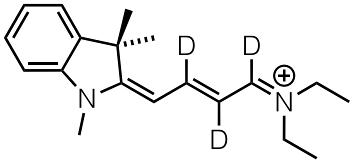

^{1}H-NMR **AsCy4**-(NEt$_2$)-D$_3$ (CD$_3$OD-d$_4$)

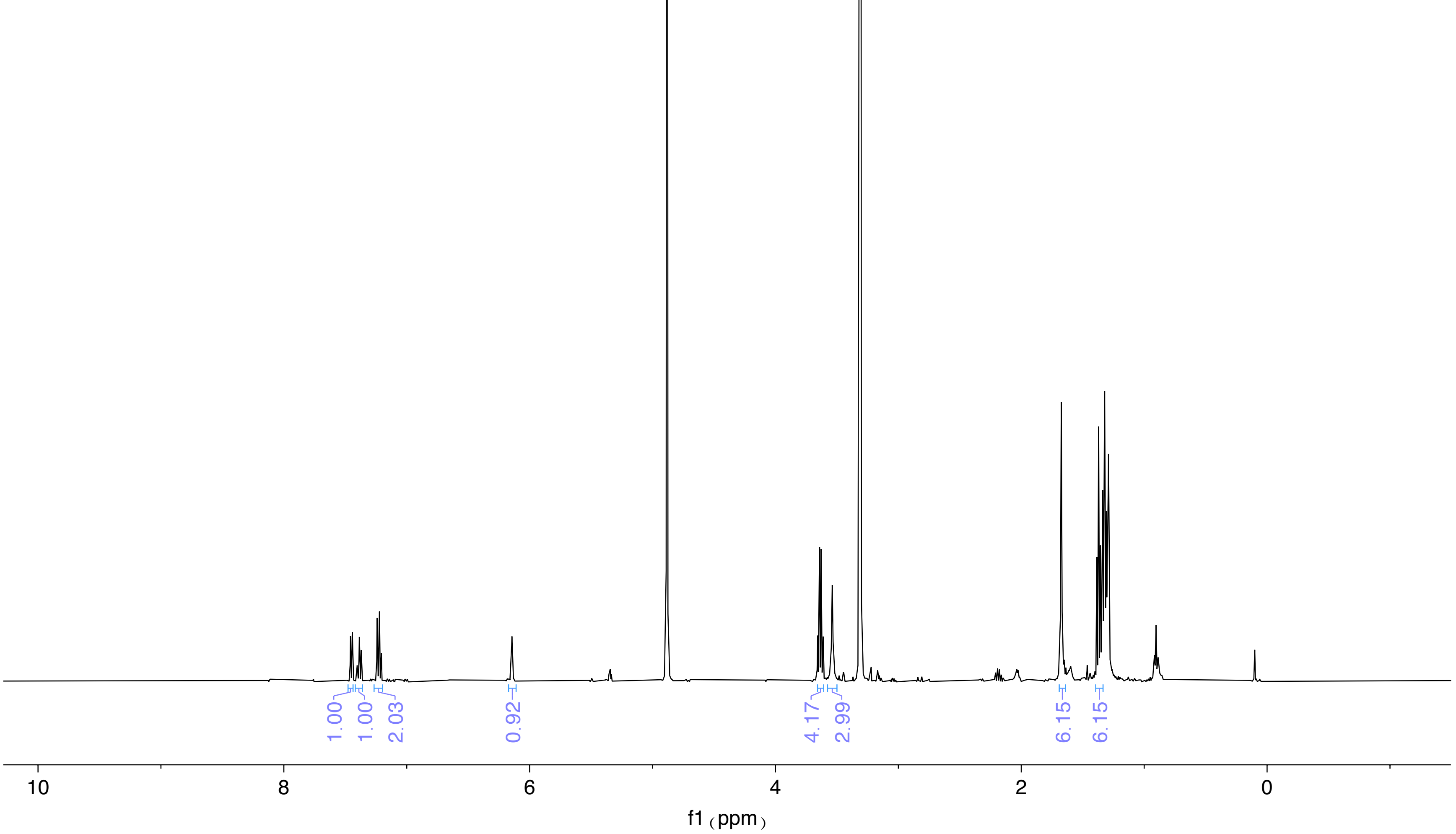

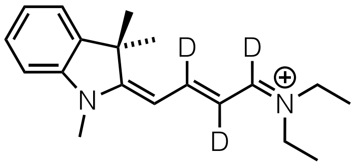

^{13}C-NMR **AsCy4**-(NEt$_2$)-D$_3$ (CD$_3$OD-d$_4$)

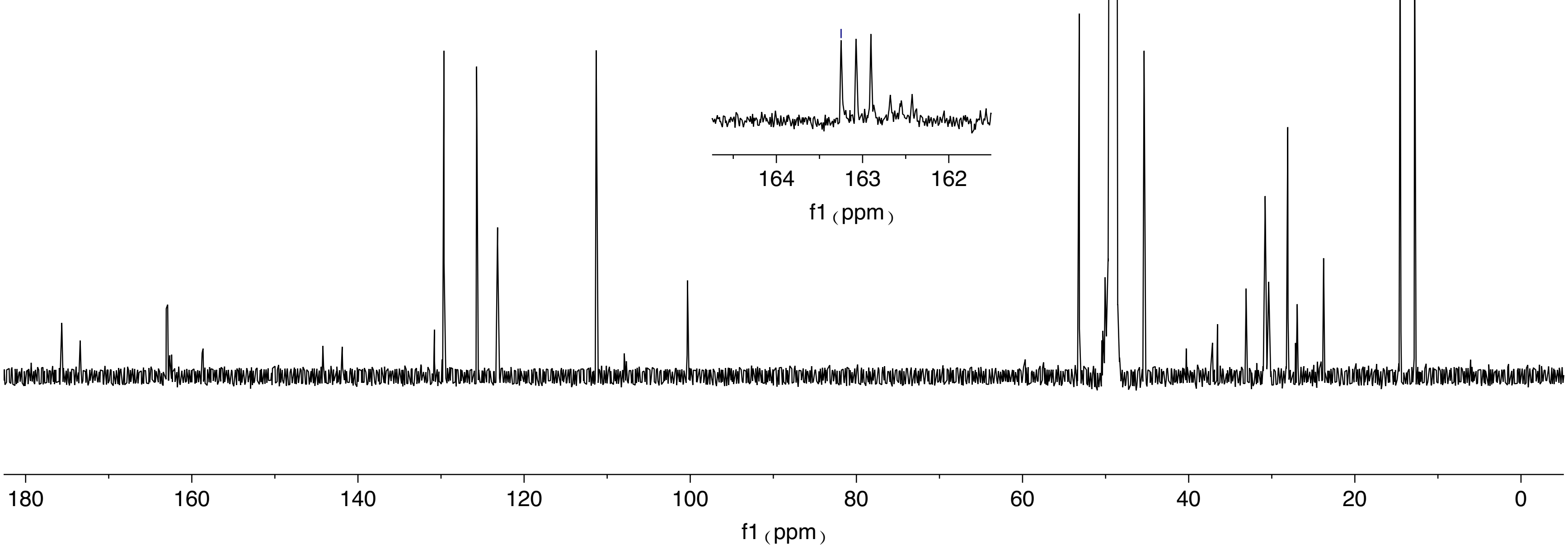

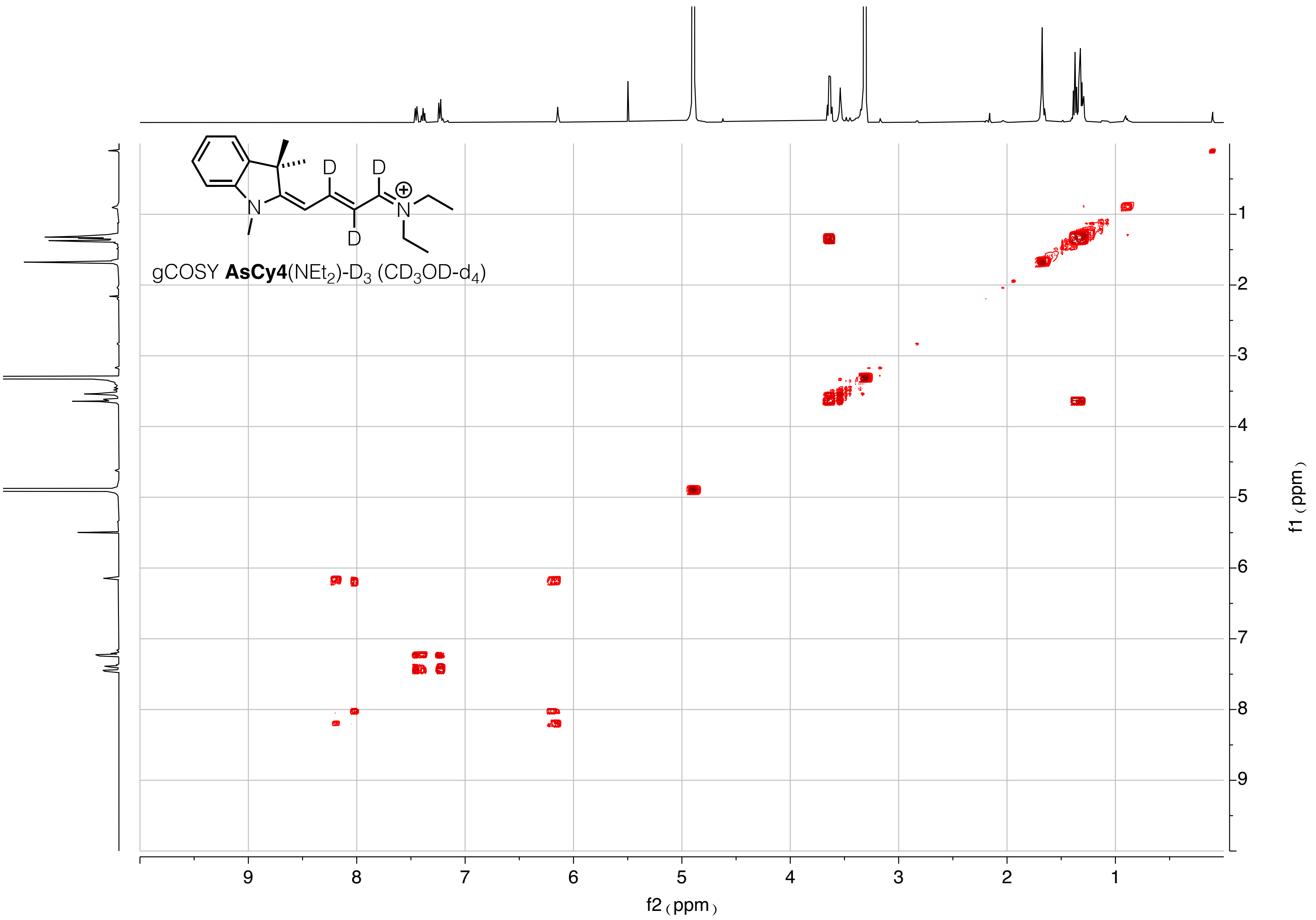

D
D
D
N
N
gCOSY **AsCy4**(NEt_2)-D_3 (CD_3OD-d_4)
1
2
3
4
5
6
7
8
9
f1 (ppm)
9
8
7
6
5
4
3
2
1
f2 (ppm)

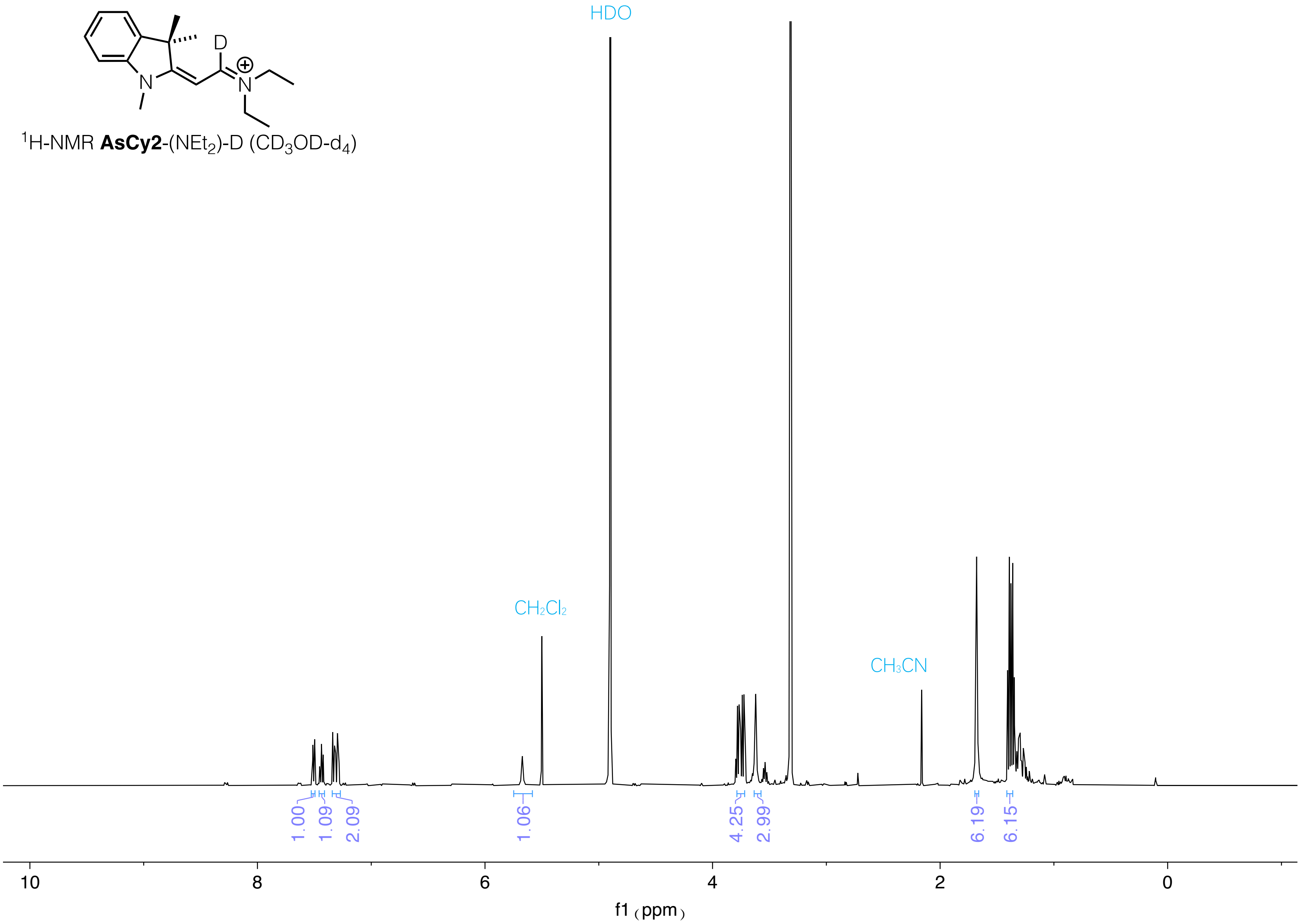
D
N
N
^{1}H-NMR **AsCy2**-(NEt$_2$)-D (CD$_3$OD-d$_4$)
HDO
CH2Cl2
CH3CN
1.00
1.09
2.09
1.06
4.25
2.99
6.19
6.15
10
8
6
4
2
0
f1 (ppm)

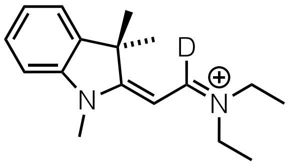

^{13}C-NMR **AsCy2**-(NEt$_2$)-D (CD$_3$OD-d$_4$)

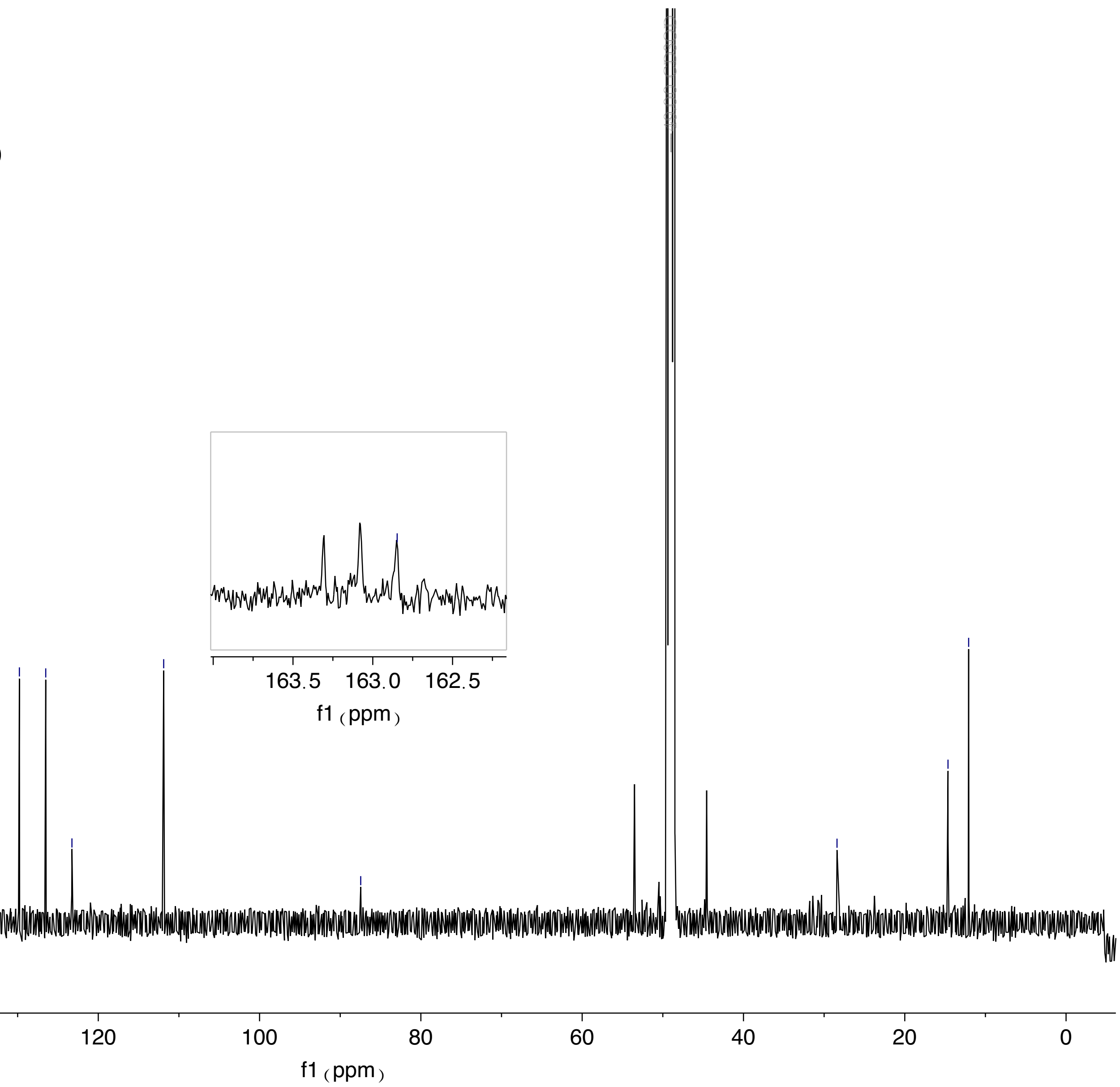

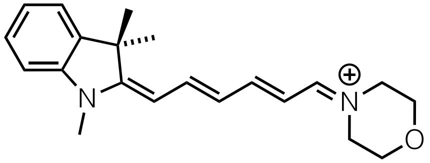

^{1}H-NMR **AsCy6**(Morph) (CD_3OD-d_4)

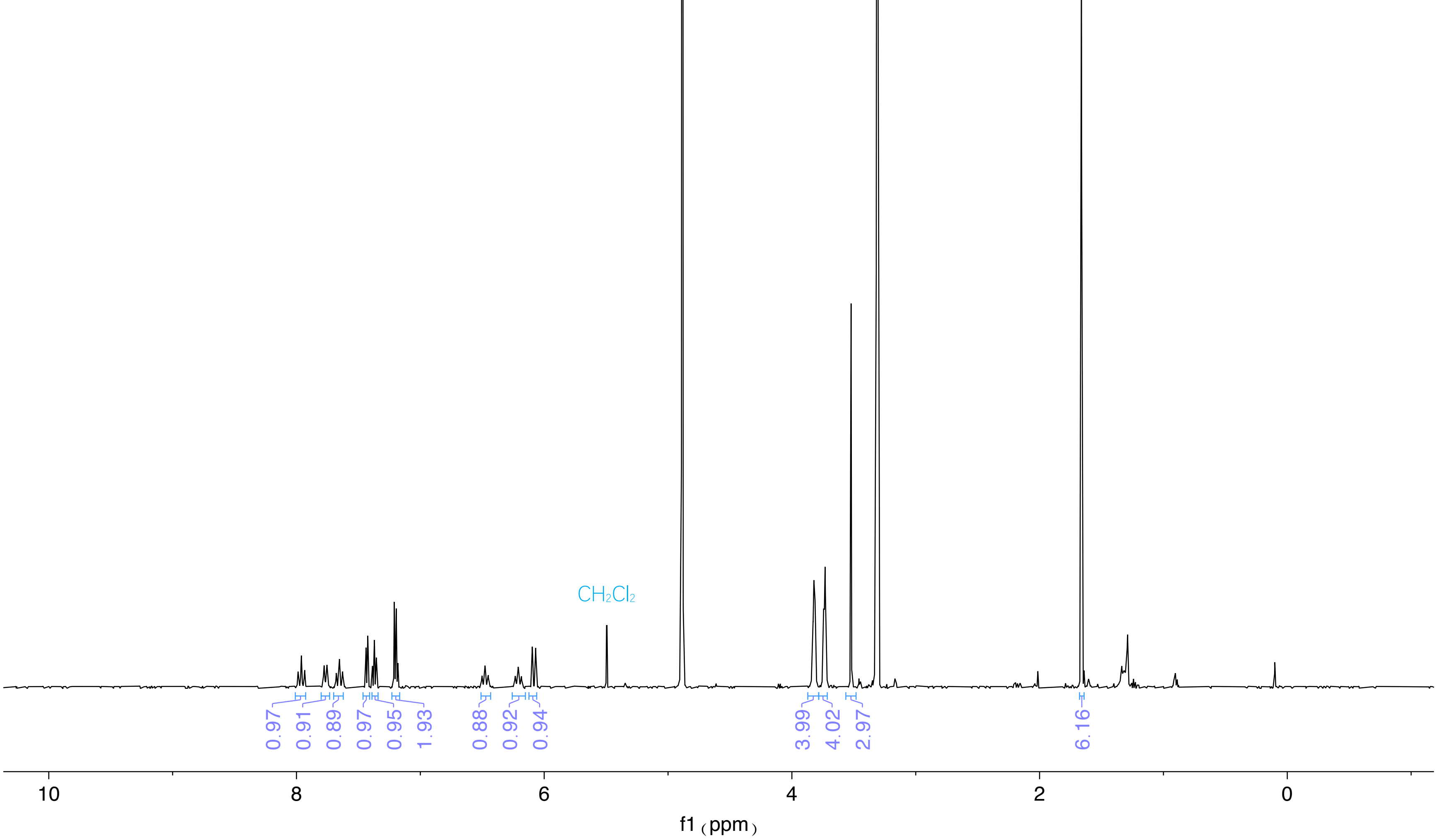

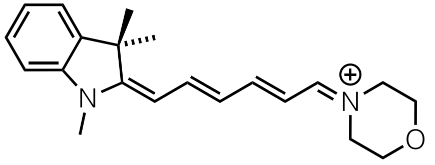

^{13}C-NMR **AsCy6**(Morph) (CD_3OD-d_4)

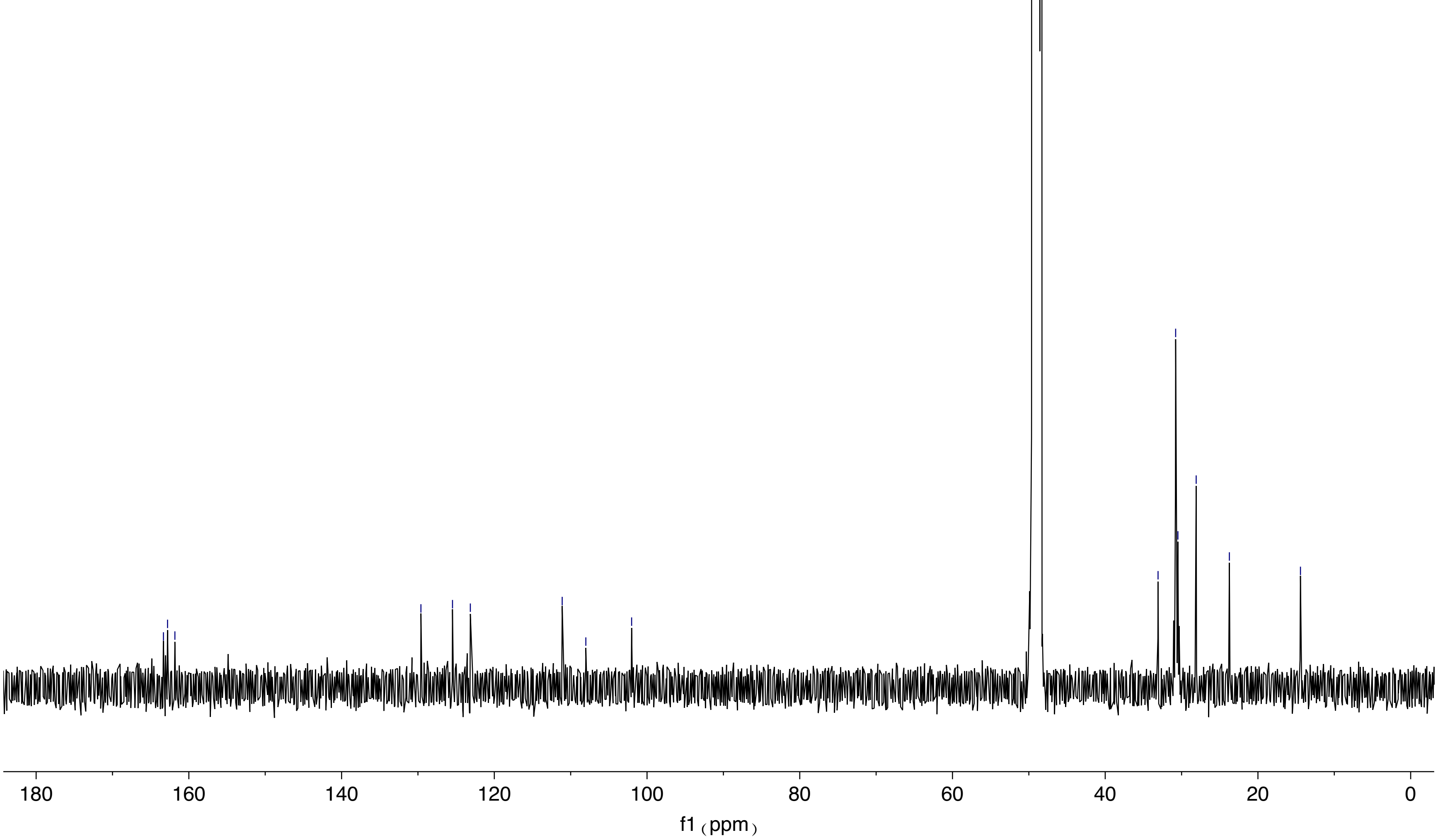

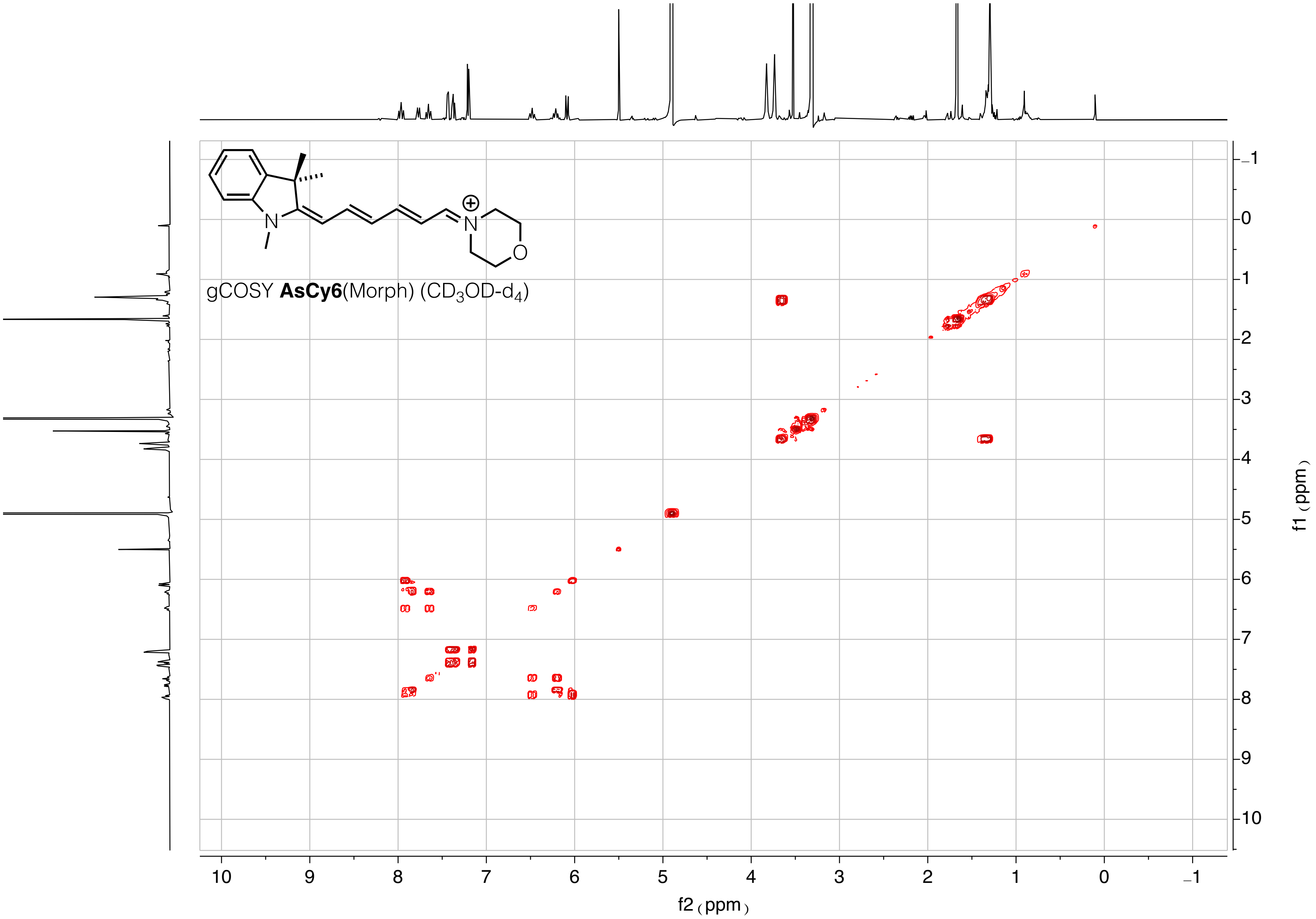
N
O
gCOSY AsCy6(Morph) (CD3OD-d4)
f2 (ppm)
f1 (ppm)

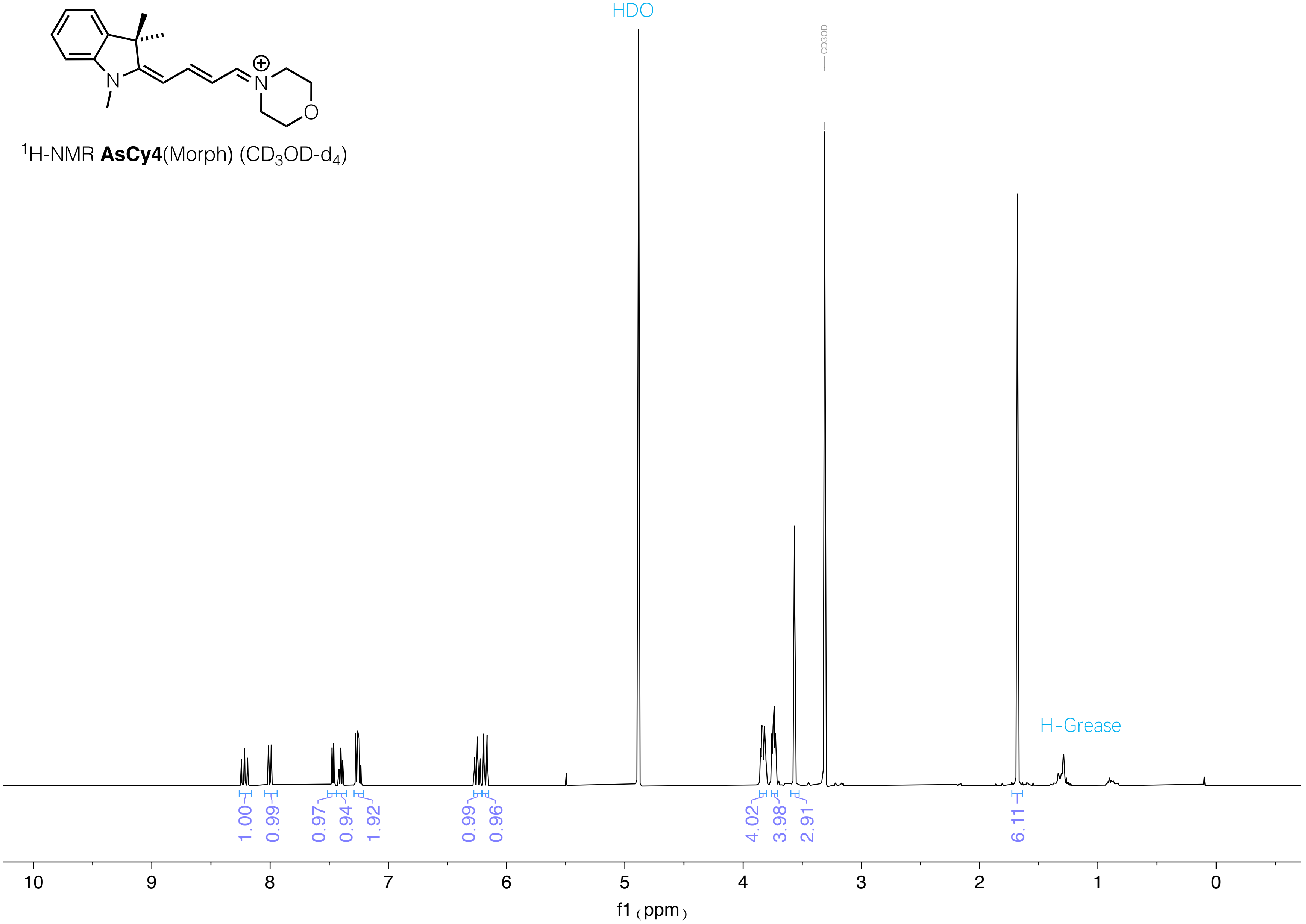

N
N
O
^{1}H-NMR **AsCy4**(Morph) (CD_3OD-d_4)
HDO
CD3OD
H-Grease
1.00
0.99
0.97
0.94
1.92
0.99
0.96
4.02
3.98
2.91
6.11
10
9
8
7
6
5
4
3
2
1
0
f1 (ppm)

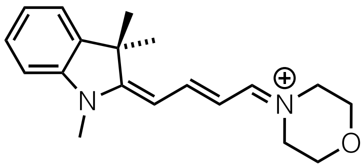

^{13}C-NMR **AsCy4**(Morph) (CD_3OD-d_4)

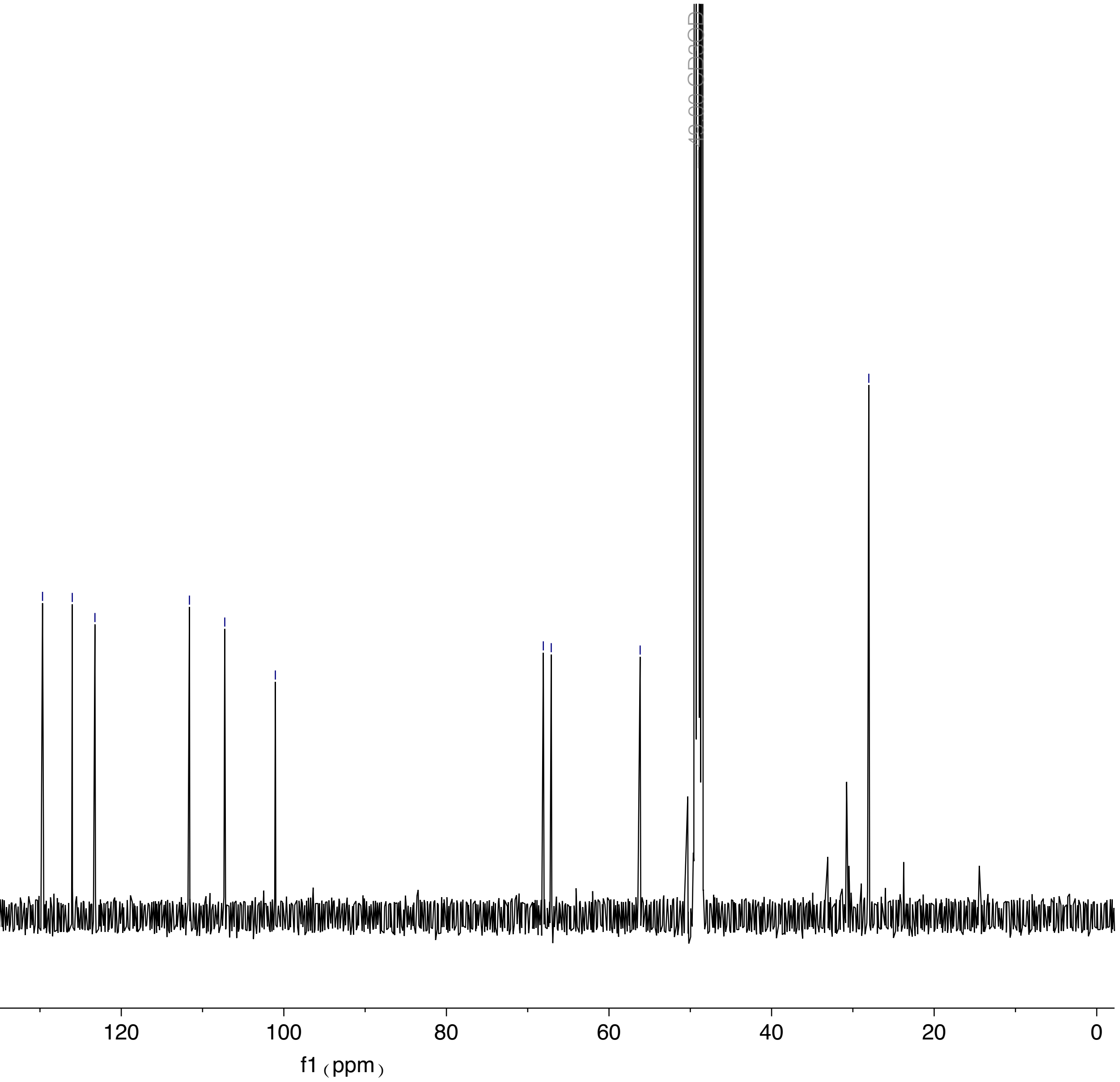

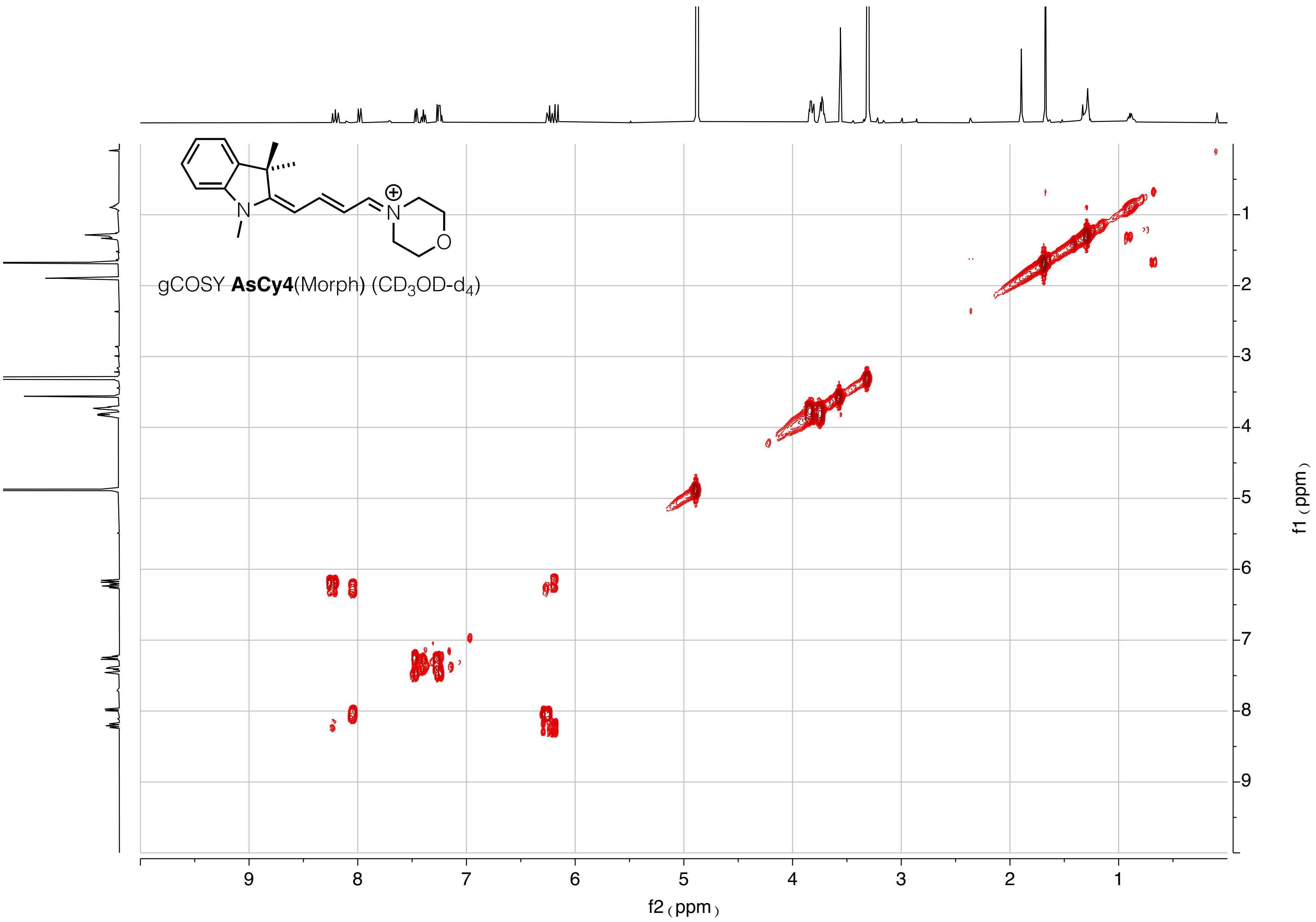
gCOSY AsCy4(Morph) (CD3OD-d4)
N
N
O
9
8
7
6
5
4
3
2
1
f2 (ppm)
f1 (ppm)

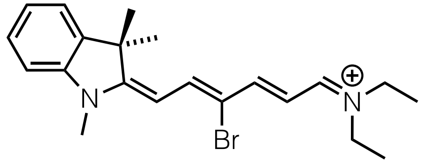

^{1}H-NMR 3'-Br-**AsCy6**(NEt_2) (CD_3OD-d_4)

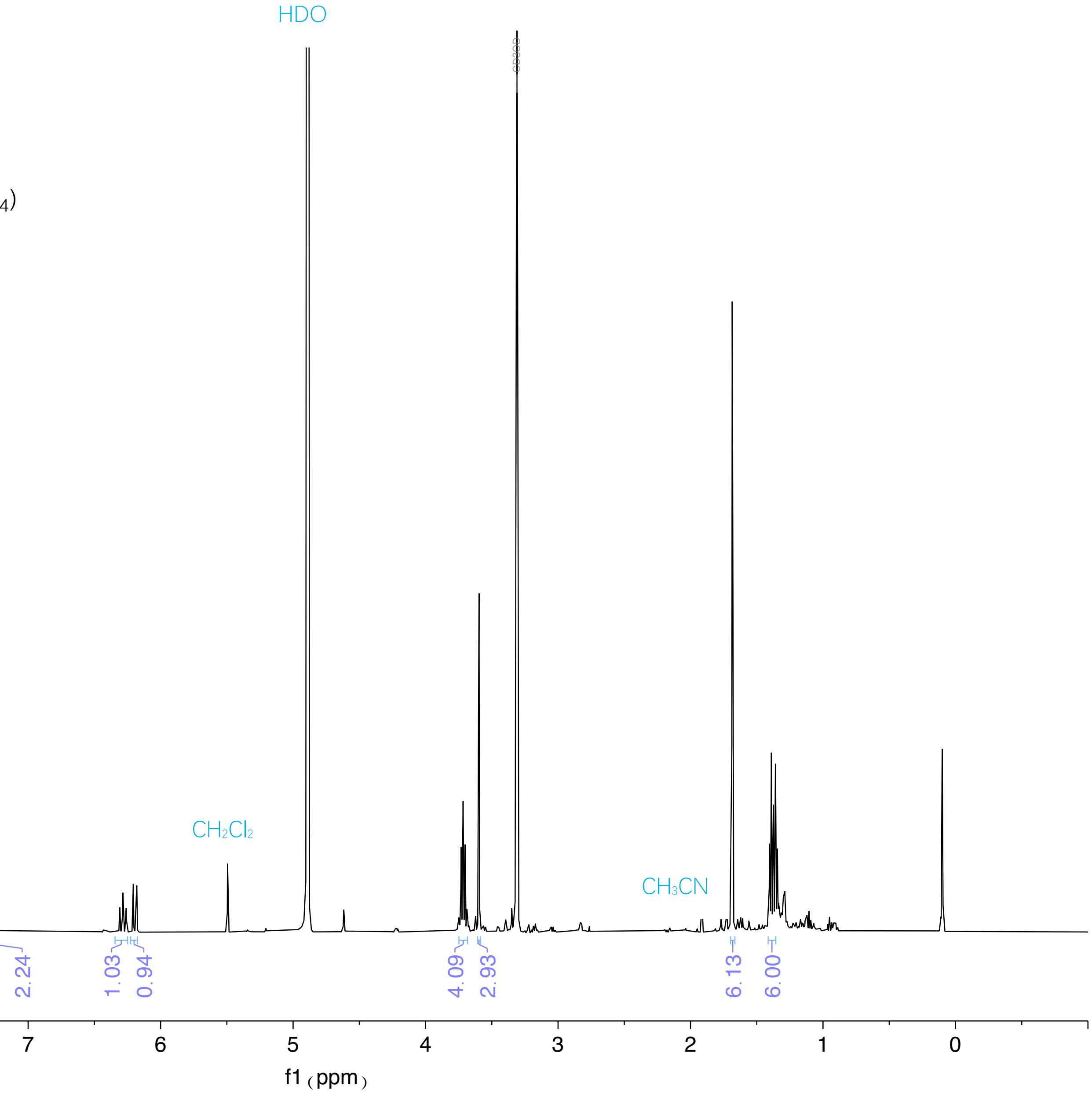

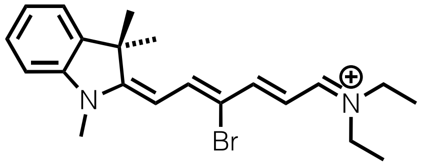

^{13}C-NMR 3'-Br-**AsCy6**(NEt$_2$) (CD$_3$OD-d$_4$)

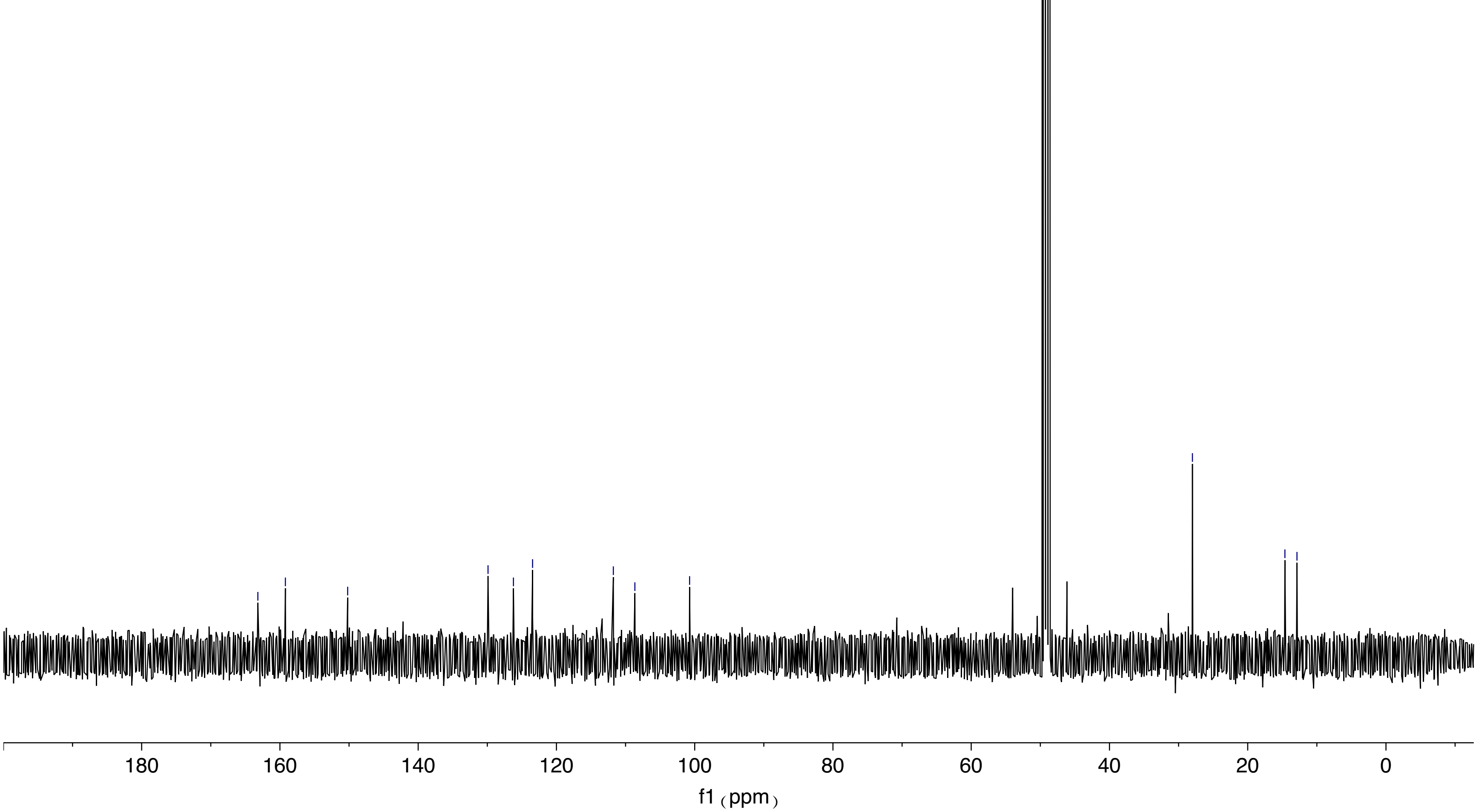

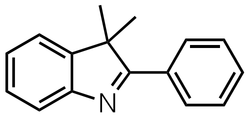

^{1}H-NMR **TMP**-[DeMe] ($CDCl_3$)

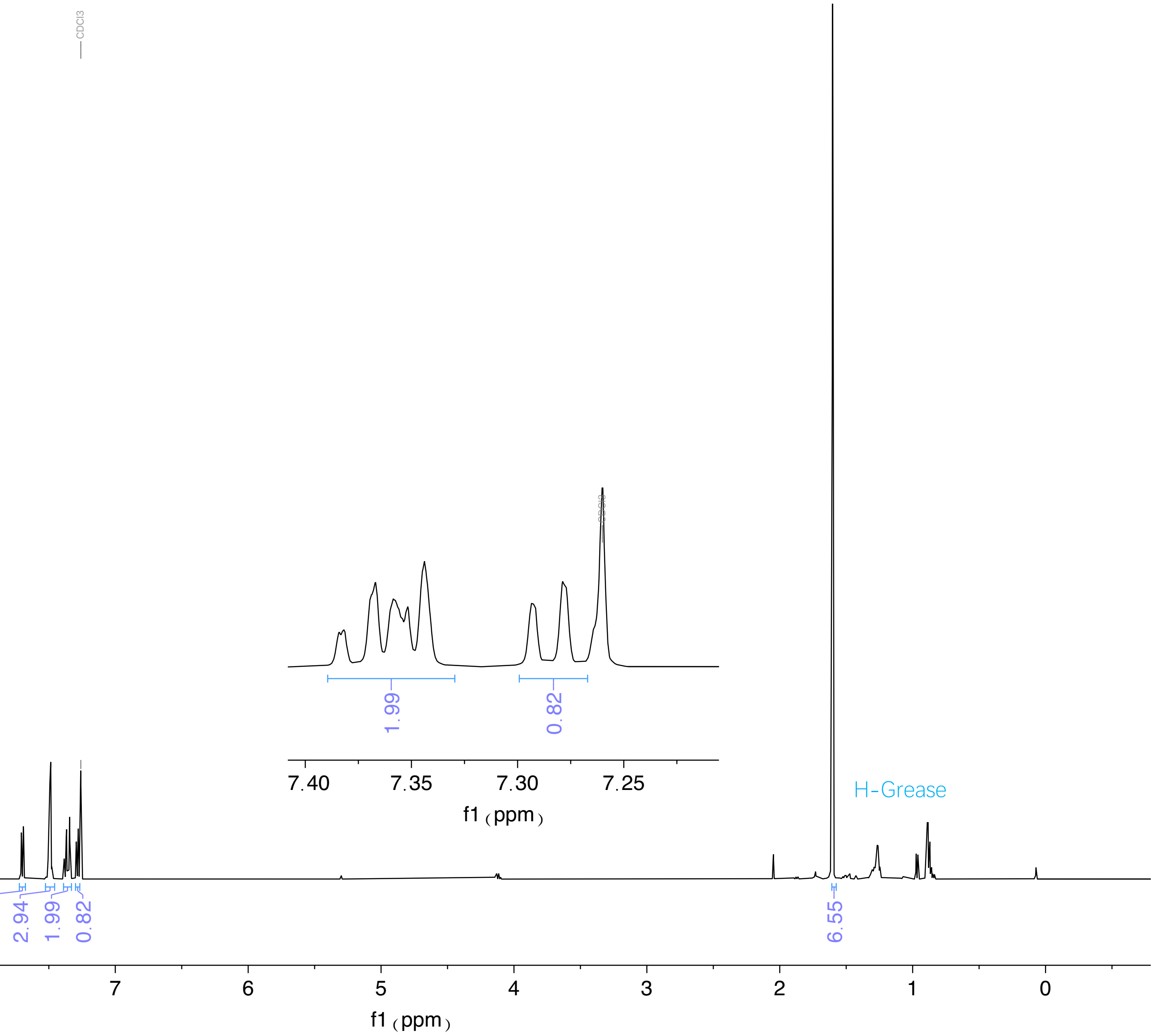

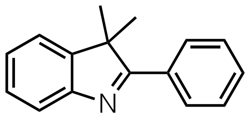

^{13}C-NMR **TMP**-[DeMe] ($CDCl_3$)

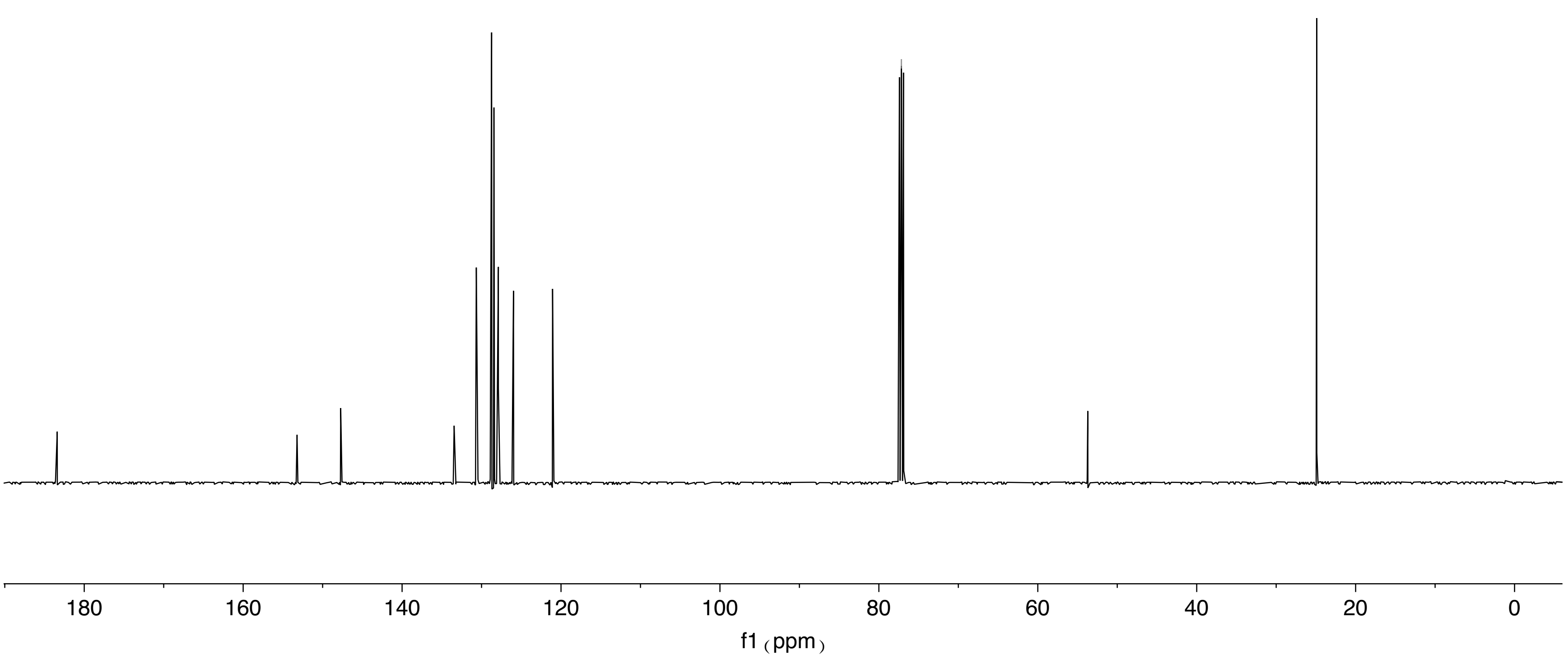

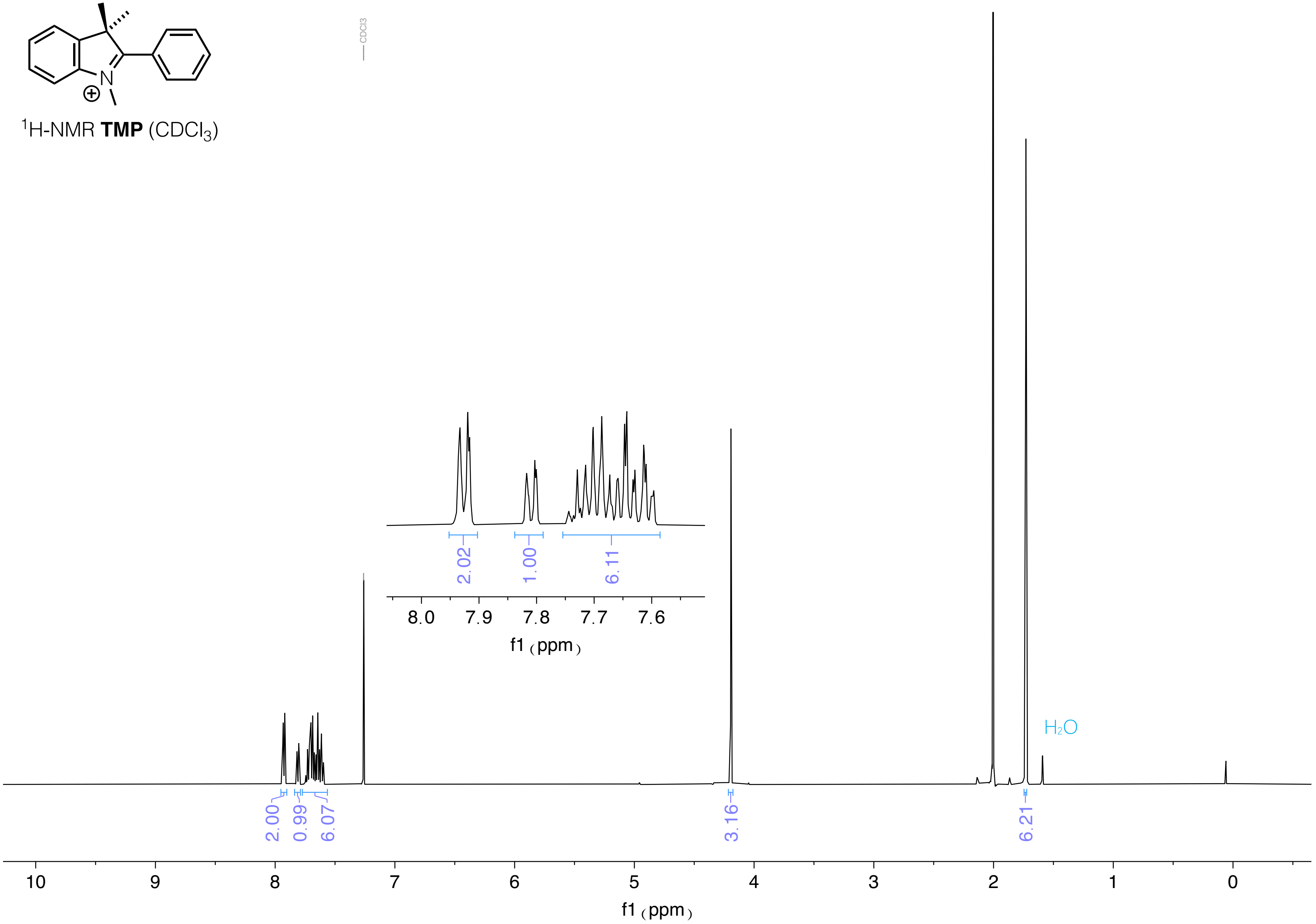

1H-NMR TMP (CDCl3)
CDCl3
H2O
2.02
1.00
6.11
8.0
7.9
7.8
7.7
7.6
f1 (ppm)
2.00
0.99
6.07
3.16
6.21
10
9
8
7
6
5
4
3
2
1
0
f1 (ppm)

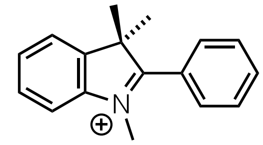

^{13}C-NMR **TMP** ($CDCl_3$)

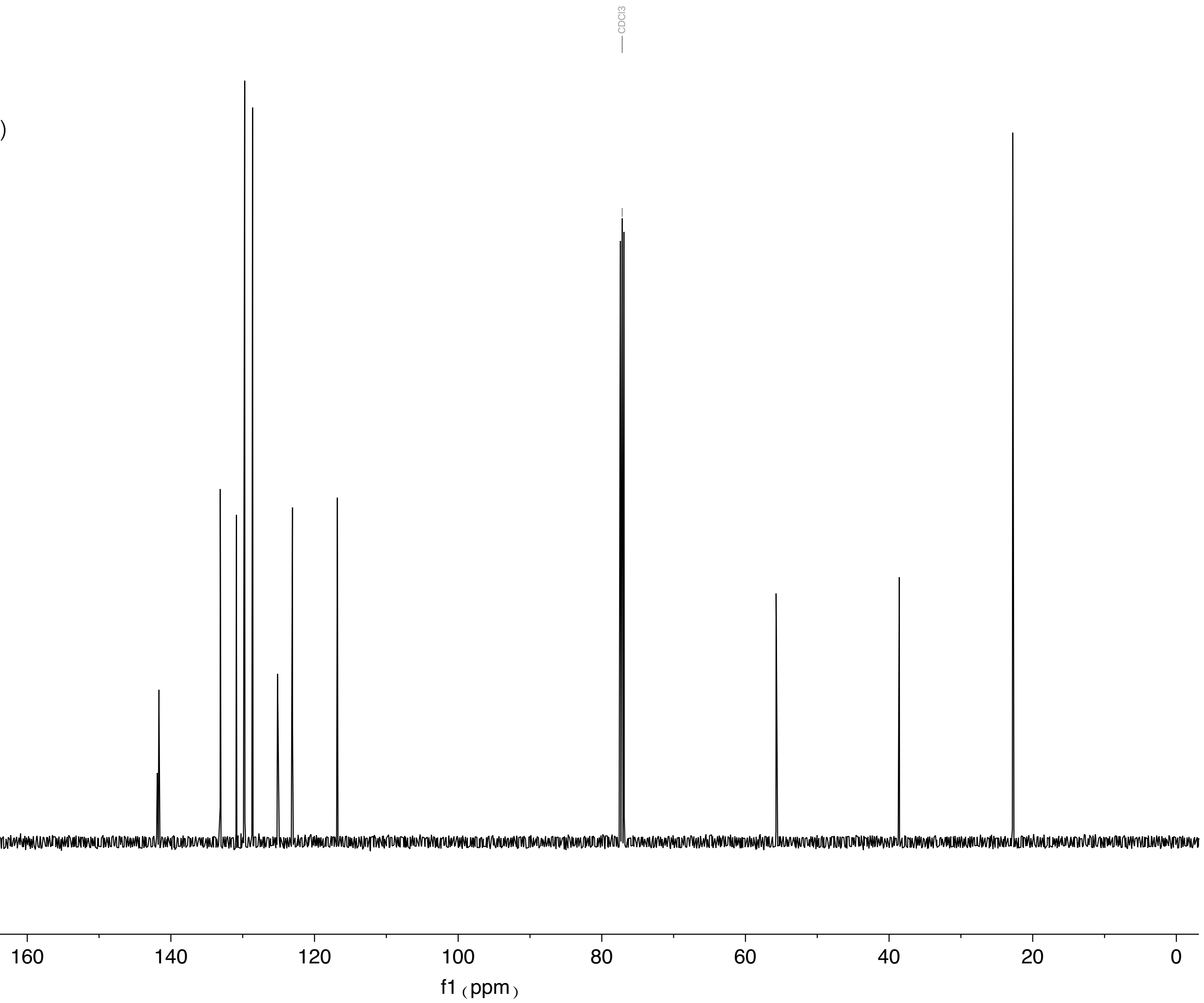

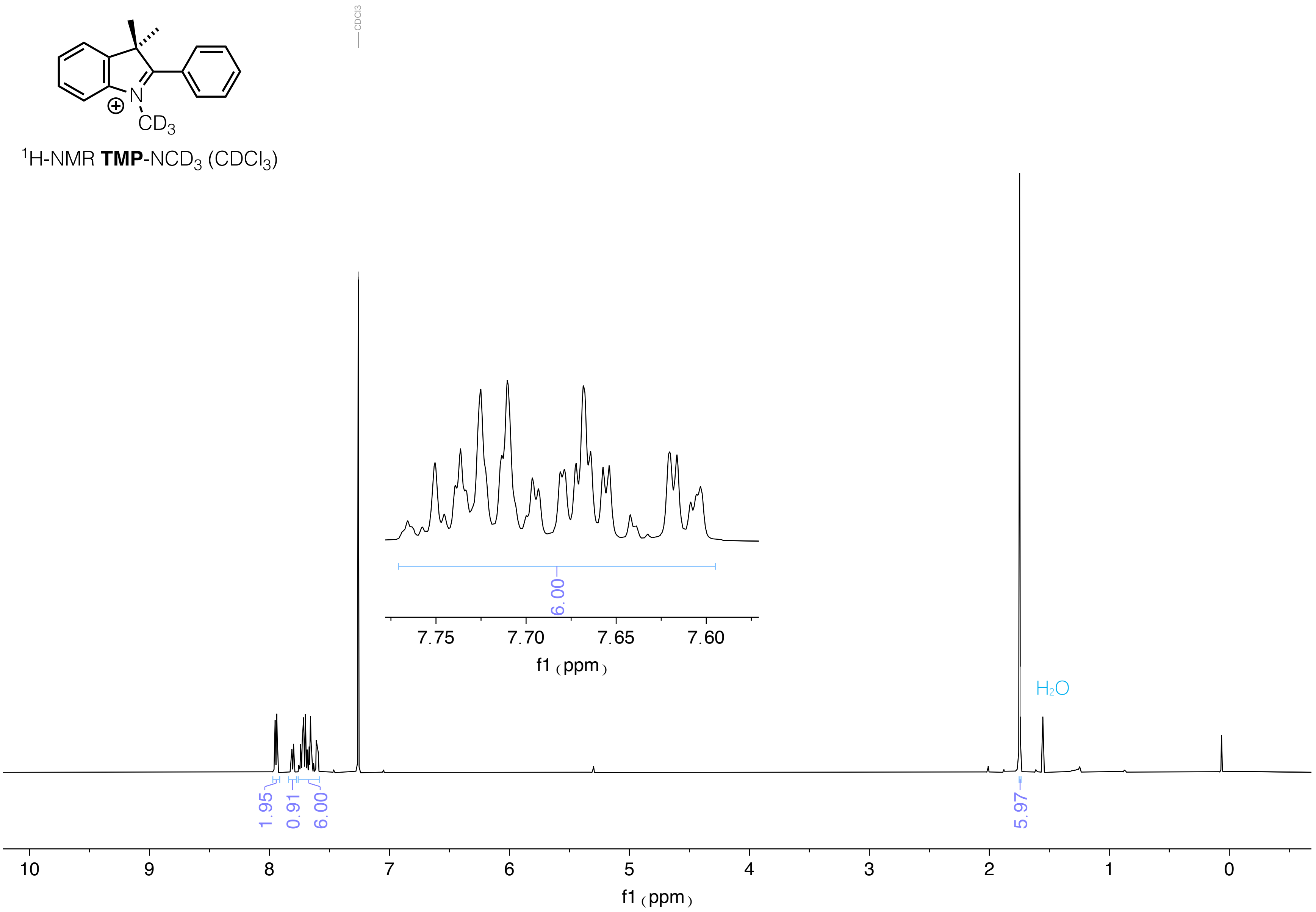

N
CD_3
^{1}H-NMR **TMP**-NCD_3 ($CDCl_3$)
CDCl3
H_2O
1.95
0.91
6.00
5.97
6.00
7.75
7.70
7.65
7.60
f1 (ppm)
10
9
8
7
6
5
4
3
2
1
0
f1 (ppm)

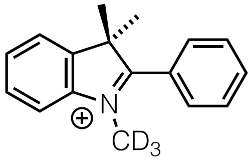

^{13}C-NMR **TMP**-NCD_3 ($CDCl_3$)

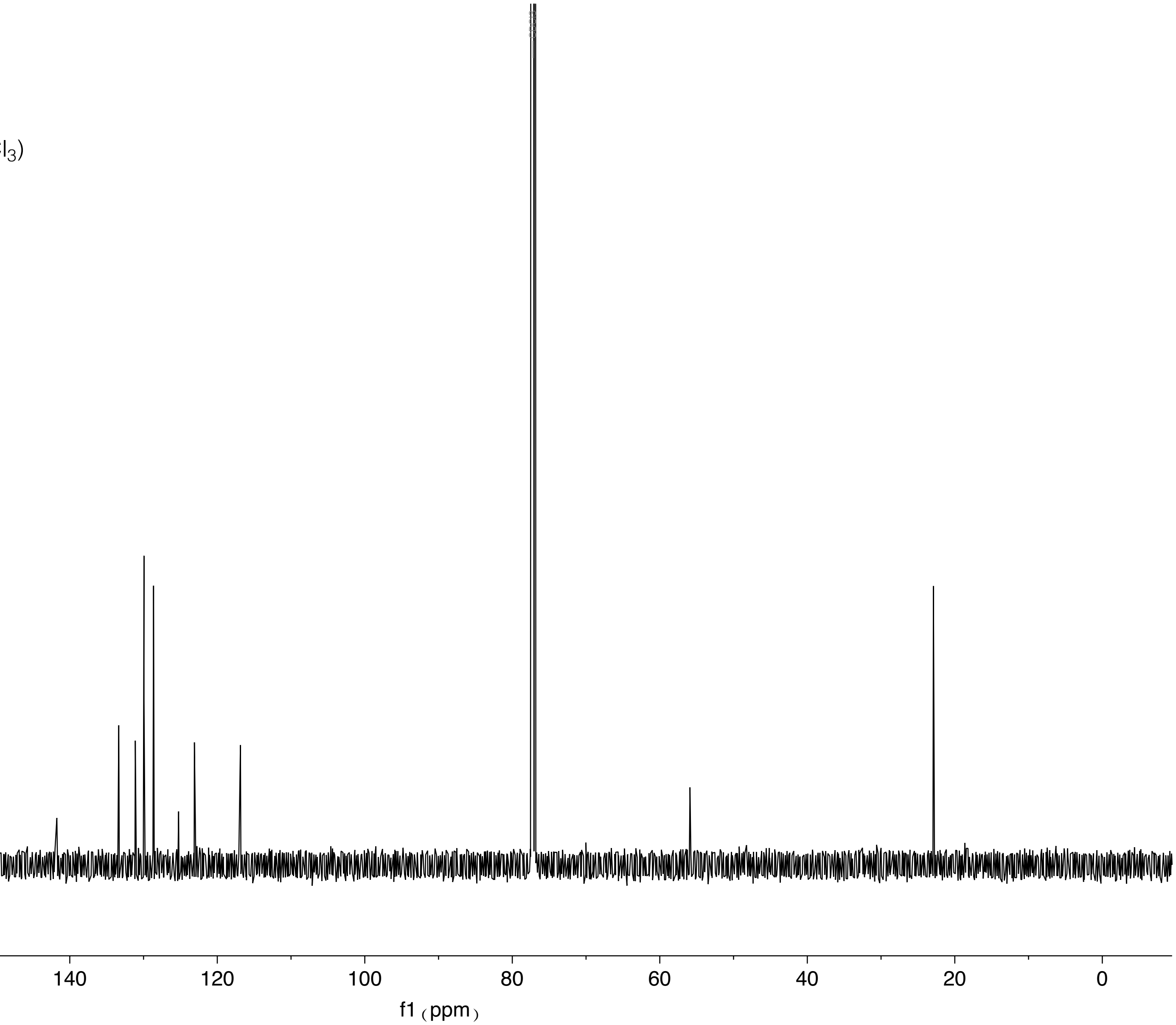

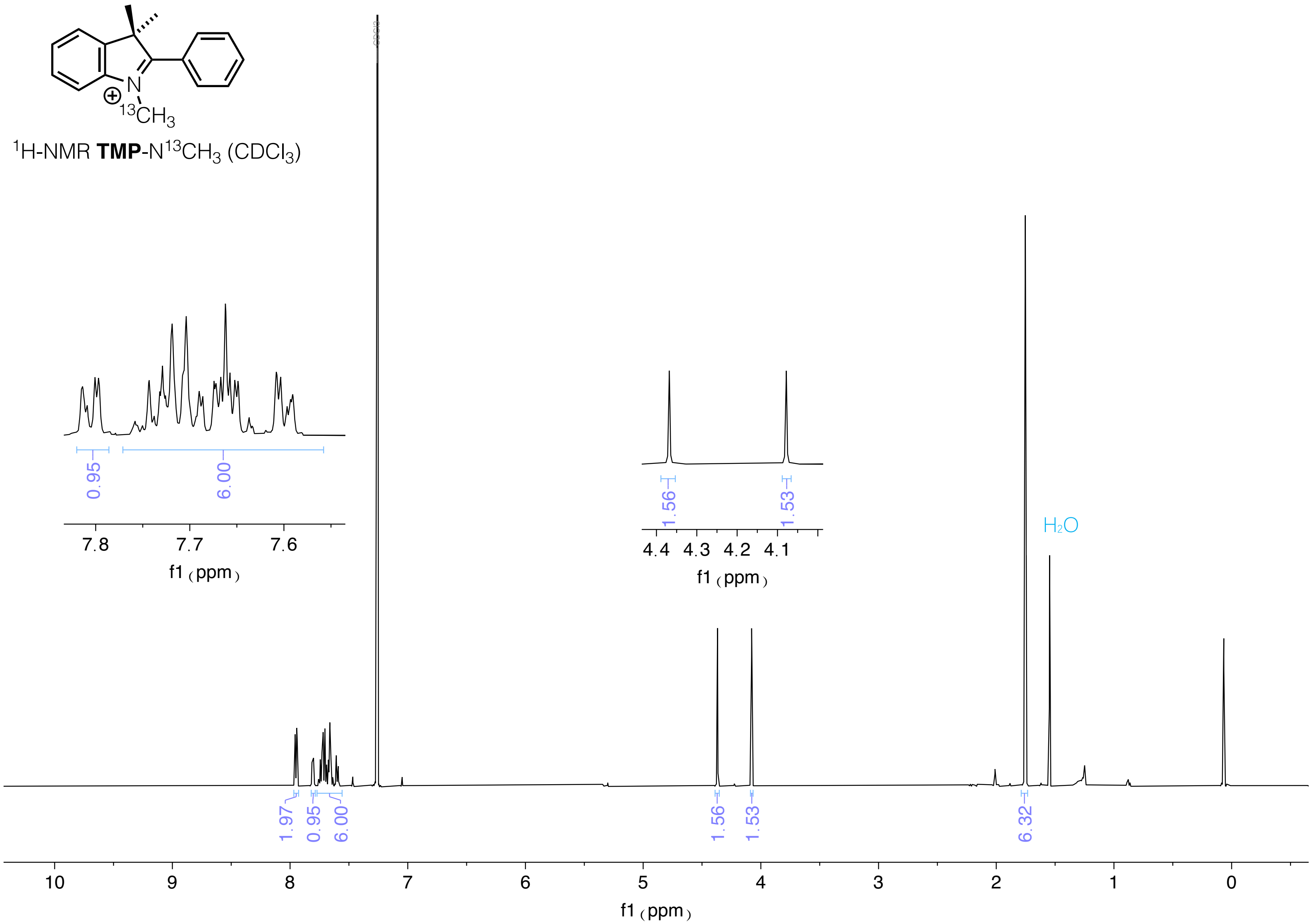

N
$^{13}CH_3$
^{1}H-NMR **TMP**-N^{13}CH$_3$ (CDCl$_3$)
0.95
6.00
7.8
7.7
7.6
f1 (ppm)
1.56
1.53
4.4
4.3
4.2
4.1
f1 (ppm)
H$_2$O
1.97
0.95
6.00
1.56
1.53
6.32
10
9
8
7
6
5
4
3
2
1
0
f1 (ppm)

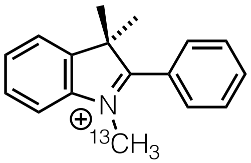

^{13}C-NMR **TMP**-N^{13}CH$_3$ (CDCl$_3$)

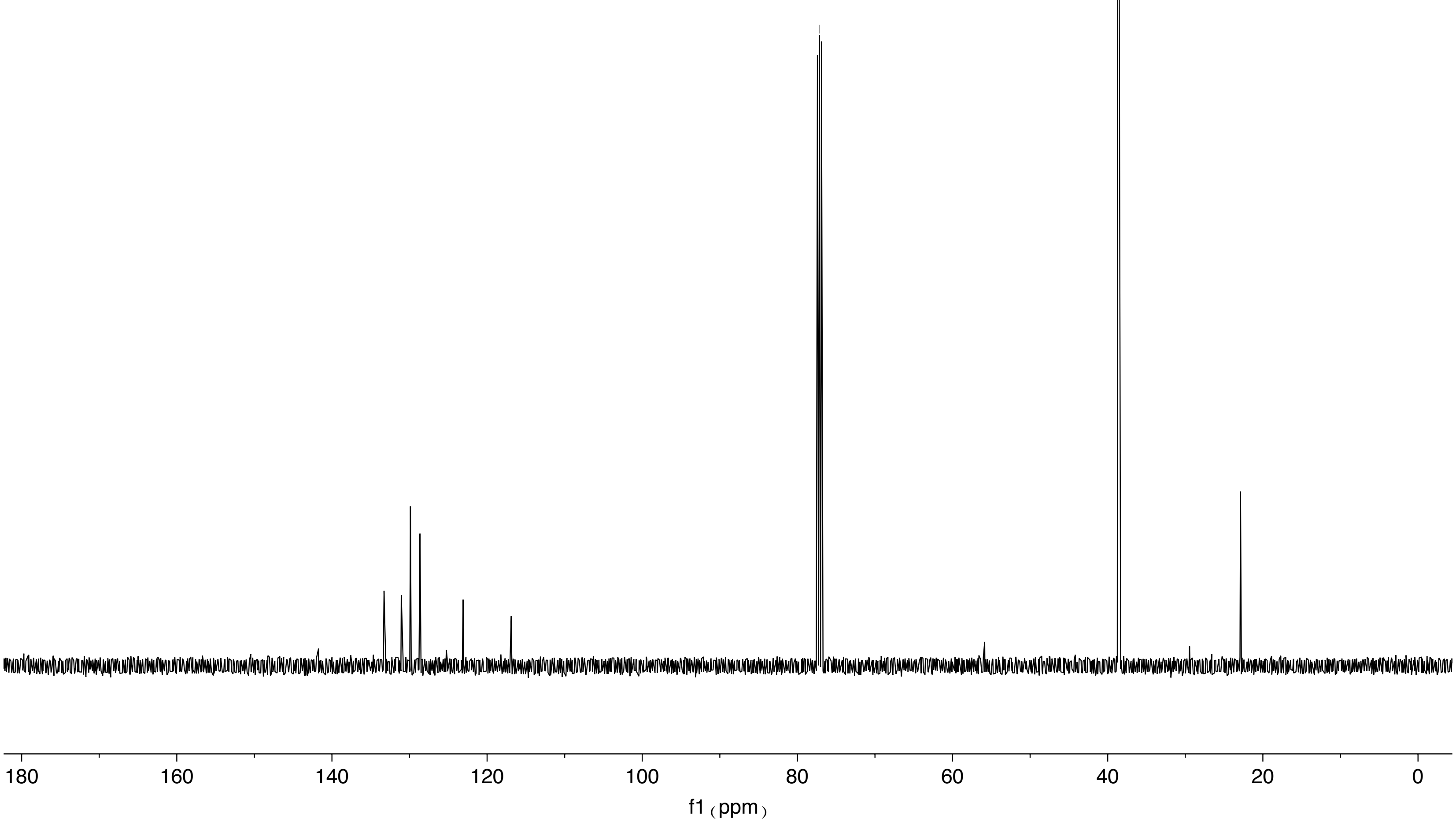

X. Quantum Chemical Calculation Results

Software used: Spartan 20

Method (both optimization and frequencies): ωB97X-D/6-31G*/CPCM:acetonitrile

Default settings were used for all studies.

Vibrational analysis was used to (a) confirm characterization of structures as minima (0 imaginary frequencies) or transition state structures (1 imaginary frequency), and (b) to compute enthalpy and entropy contributions to their energies.

Table of contents:

Section 1: Building blocks

Label (charge)	E_{elec}(au)	H°(au)	G°(au)	NImag
Cy7 (+)	-1233.92804	-1233.37501	-1233.45284	0
Cy5 (+)	-1156.55161	-1156.03191	-1156.10620	0
Cy3 (+)	-1079.17623	-1078.69001	-1078.76083	0
FB (0)	-520.89970	-520.65742	-520.70605	0
FB-H (+)	-521.37449	-521.11865	-521.16787	0
FB-CH=CH_2 (0)	-598.27485	-597.99812	-598.05066	0
FB=CH-CH_3 (+)	-598.74792	-598.45844	-598.51154	0
FB-CH=CH-CH=CH_2 (0)	-675.65440	-675.34387	-675.40027	0
FB=CH-CH=CH-CH_3 (+)	-676.12830	-675.80485	-675.86170	0
TMP (+)	-713.04238	-712.73291	-712.78752	0
TMP-H (0)	-713.80192	-713.48302	-713.53765	0

Section 2: Cy7 cyclization to form TMP and FB

Label (charge)	E_{elec}(au)	H°(au)	G°(au)	NImag

Cy7 coiled	-1233.92072	-1233.36589	-1233.44145	0
Cy7 cis cyclization TS (+)	-1233.88889	-1233.33511	-1233.41129	1
Cy7 trans cyclization TS (+)	-1233.87638	-1233.32302	-1233.39947	1
Cy7 cyclized cis (+)	-1233.92525	-1233.36852	-1233.44333	0
Cy7 cyclized trans (+)	-1233.92404	-1233.36804	-1233.44340	0
Cy7 cyclized deprot. (0)	-1233.45455	-1232.91277	-1232.98840	0
Cy7 cyclized H isom. (+)	-1233.92970	-1233.37264	-1233.44698	0
Cy7 cyclized H isom. FB cleavage TS (+)	-1233.90844	-1233.35388	-1233.42907	1
Cy7 cyclized deprot. oxidized (+)	-1232.75929	-1232.22529	-1232.29935	0

Section 3: FB+Cy7 reaction at C2’

Label (charge)	E_{elec}(au)	H°(au)	G°(au)	NImag
Cy7+FB C2' attack TS (+)	-1754.82522	-1754.02688	-1754.12449	1
Cy7+FB C2' adduct (+)	-1754.85725	-1754.05451	-1754.15003	0
Cy7+FB C2' adduct C1' deprot. (0)	-1754.36997	-1753.58399	-1753.68148	0
Cy3+FBCH=CH-CH=CH_2 C2’ adduct (+)	-1754.84877	-1754.04725	-1754.14328	0
Cy3+FBCH=CH-CH=CH_2 C2’ cleavage TS (+)	-1754.81653	-1754.01754	-1754.11482	1

Section 4: FB+Cy7 reaction at C4’

Label (charge)	E_{elec}(au)	H°(au)	G°(au)	NImag
Cy7+FB C4’ attack TS (+)	-1754.82742	-1754.02865	-1754.12608	1
Cy7+FB C4’ adduct (+)	-1754.86145	-1754.05837	-1754.15331	0
Cy7+FB C4’ adduct deprot. (0)	-1754.37278	-1753.58666	-1753.68431	0
Cy5+FB-CH=CH_2 C2’ adduct (+)	-1754.85097	-1754.04824	-1754.14388	0
Cy5+FBCH=CH_2 C2’ cleavage TS (+)	-1754.82036	-1754.02130	-1754.11876	1

Section 5: FB+Cy5 reaction at C2’

Label (charge)	E_{elec}(au)	H°(au)	G°(au)	NImag
Cy5+FB C2’ attack TS (+)	-1677.44694	-1676.68222	-1676.77648	1

Cy5+FB C2’ adduct (+)	-1677.47748	-1676.70891	-1676.80149	0
Cy5+FB C2’ adduct deprot. (0)	-1676.99287	-1676.24027	-1676.33384	0
Cy3+FB-CH=CH_2 C2’ adduct (+)	-1677.46662	-1676.69908	-1676.79197	0
Cy3+FB=CHCH=CH_2 C2’ cleavage TS (+)	-1677.44102	-1676.67557	-1676.76915	1

Section 6: FB+Cy3 reaction at C2’

Label (charge)	E_{elec}(au)	H°(au)	G°(au)	NImag
Cy3+FB C2’ attack TS (+)	-1600.06623	-1599.33430	-1599.42464	1
Cy3+FB C2' adduct (+)	-1600.08996	-1599.35592	-1599.44540	0
Cy3+FB C2' adduct deprot. (0)	-1599.61102	-1598.89260	-1598.98304	0
Cy3+FB C2' adduct deprot. oxidized (+)	-1598.88894	-1598.17747	-1598.26542	0

Section 1: Building blocks

Cy7 (+)

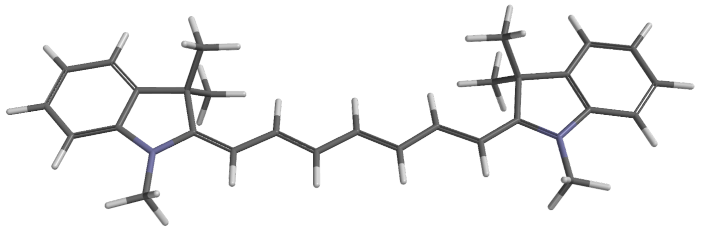

E_{elec}(au)	H°(au)	G°(au)	NImag
-1233.92804	-1233.37501	-1233.45284	0

C	9.0241	-0.0659	0.7885
C	9.1386	-0.1319	2.1767
C	8.0084	-0.1646	2.9966
C	6.7702	-0.1287	2.3710
C	6.6391	-0.0643	0.9874
C	7.7645	-0.0318	0.1828
N	5.4828	-0.1510	2.9464
C	4.5176	-0.1073	2.0037
C	5.1708	-0.0442	0.6197
C	4.8225	-1.2803	-0.2324
C	3.1686	-0.1200	2.3345
C	2.1118	-0.0767	1.4212
C	0.7692	-0.0817	1.7794
C	-0.2382	-0.0361	0.8174
C	-1.6070	-0.0278	1.0819
C	5.2235	-0.2121	4.3742
C	-2.5423	0.0173	0.0556
C	-3.9256	0.0346	0.2546
C	-4.8834	0.0733	-0.7504
N	-6.2046	0.0959	-0.4735
C	-6.9804	0.1164	-1.6508
C	-6.1252	0.1171	-2.7482
C	-4.6892	0.0927	-2.2697
C	-8.3610	0.1333	-1.7892
C	-8.8720	0.1539	-3.0891
C	-8.0282	0.1567	-4.1992
C	-6.6404	0.1380	-4.0326
C	-3.9854	-1.1824	-2.7725
C	-6.7529	0.0936	0.8713
C	4.8269	1.2654	-0.1161
C	-3.9552	1.3659	-2.7339
H	9.9186	-0.0418	0.1740
H	10.1224	-0.1591	2.6350
H	8.1136	-0.2171	4.0746
H	7.6715	0.0185	-0.8987
H	5.0525	-2.2025	0.3094
H	3.7666	-1.2955	-0.5132
H	5.4180	-1.2603	-1.1504

H	2.9036	-0.1650	3.3870
H	2.3273	-0.0359	0.3566
H	0.4977	-0.1180	2.8332
H	0.0699	-0.0034	-0.2292
H	-1.9493	-0.0563	2.1151
H	6.1687	-0.2249	4.9127
H	4.6499	0.6640	4.6894
H	4.6633	-1.1193	4.6184
H	-2.1475	0.0415	-0.9568
H	-4.2726	0.0138	1.2838
H	-9.0311	0.1300	-0.9366
H	-9.9483	0.1675	-3.2310
H	-8.4513	0.1725	-5.1986
H	-5.9793	0.1391	-4.8950
H	-4.5055	-2.0780	-2.4200
H	-3.9925	-1.1859	-3.8669
H	-2.9456	-1.2300	-2.4400
H	-6.4772	-0.8281	1.3917
H	-7.8376	0.1598	0.8203
H	-6.3759	0.9537	1.4311
H	5.4191	1.3236	-1.0346
H	5.0648	2.1340	0.5051
H	3.7699	1.3116	-0.3896
H	-2.9149	1.3797	-2.3995
H	-4.4552	2.2621	-2.3549
H	-3.9610	1.4024	-3.8277

Cy5 (+)

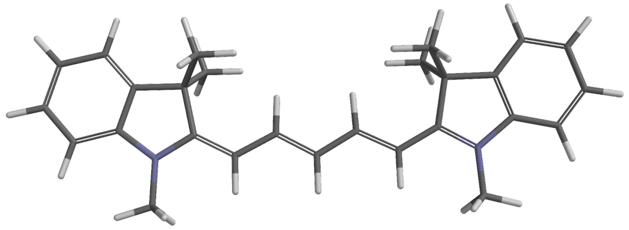

E_{elec}(au)	H°(au)	G°(au)	NImag
-1156.55161	-1156.03191	-1156.10620	0

C	-3.0058	4.0075	6.0977
C	-3.3535	5.1678	5.4067
C	-3.1187	5.2943	4.0359
C	-2.5257	4.2171	3.3933
C	-2.1720	3.0534	4.0682
C	-2.4086	2.9366	5.4267
N	-2.1800	4.0727	2.0326
C	-1.6143	2.8737	1.7904
C	-1.5535	2.0733	3.0943
C	-2.4086	0.7930	3.0206
C	-1.1789	2.5120	0.5197
C	-0.5953	1.2892	0.1892
C	-0.1529	0.9493	-1.0858
C	-2.4017	5.0875	1.0175
C	0.4195	-0.2940	-1.3367
C	0.8949	-0.7047	-2.5820
C	1.4664	-1.9437	-2.8527
N	1.9118	-2.2673	-4.0828
C	2.4453	-3.5734	-4.1144
C	2.3386	-4.1279	-2.8432
C	1.6989	-3.1255	-1.9068
C	3.0021	-4.2651	-5.1803
C	3.4589	-5.5609	-4.9296
C	3.3574	-6.1320	-3.6612
C	2.7930	-5.4131	-2.6039
C	0.3722	-3.6853	-1.3577
C	1.8469	-1.3799	-5.2318
C	-0.1013	1.7625	3.5059
C	2.6765	-2.7671	-0.7706
H	-3.1994	3.9359	7.1632
H	-3.8169	5.9926	5.9391
H	-3.3945	6.2039	3.5137
H	-2.1362	2.0309	5.9616
H	-3.4322	1.0268	2.7135
H	-1.9921	0.0660	2.3192
H	-2.4415	0.3296	4.0111
H	-1.2964	3.2347	-0.2823
H	-3.0320	4.6870	0.2188
H	-2.9041	5.9422	1.4658
H	-1.4470	5.4159	0.5973

H	0.8095	0.0053	-3.3993
H	3.0887	-3.8346	-6.1718
H	3.9009	-6.1293	-5.7420
H	3.7203	-7.1411	-3.4940
H	2.7132	-5.8539	-1.6140
H	-0.3176	-3.9208	-2.1734
H	0.5767	-4.6047	-0.8006
H	-0.1161	-2.9801	-0.6812
H	0.8070	-1.1243	-5.4522
H	2.2780	-1.8772	-6.0981
H	2.4121	-0.4657	-5.0304
H	-0.1016	1.3198	4.5067
H	0.4993	2.6766	3.5318
H	0.3720	1.0541	2.8219
H	2.2462	-2.0400	-0.0772
H	3.6073	-2.3559	-1.1742
H	2.9145	-3.6749	-0.2065
H	-0.2537	1.6676	-1.8975
H	-0.4666	0.5346	0.9608
H	0.4903	-0.9706	-0.4891

Cy3 (+)

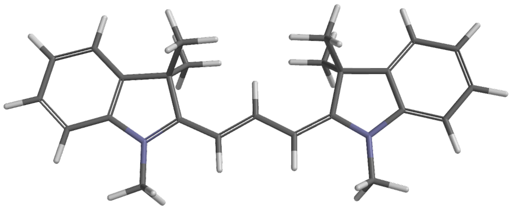

E_{elec}(au)	H°(au)	G°(au)	NImag
-1079.17623	-1078.69001	-1078.76083	0

C	-3.6376	-2.8012	4.9741
C	-4.7753	-2.9976	4.1918
C	-4.8054	-2.6200	2.8474
C	-3.6562	-2.0426	2.3275
C	-2.5134	-1.8415	3.0934
C	-2.4922	-2.2186	4.4250
N	-3.4099	-1.5703	1.0192
C	-2.1641	-1.0777	0.8968
C	-1.4399	-1.2011	2.2406
C	-0.2181	-2.1358	2.1438
C	-1.6978	-0.5625	-0.3109
C	-4.3761	-1.6264	-0.0655
C	-0.4296	-0.0274	-0.5215
C	-0.0170	0.4789	-1.7513
C	1.2350	1.0306	-2.0162
N	1.5583	1.4911	-3.2380
C	2.8672	2.0213	-3.2639
C	3.4256	1.8854	-1.9980
C	2.4231	1.2323	-1.0714
C	3.5549	2.6004	-4.3203
C	4.8536	3.0465	-4.0654
C	5.4300	2.9136	-2.8023
C	4.7140	2.3286	-1.7543
C	2.9765	-0.1094	-0.5539
C	0.6683	1.4556	-4.3868
C	-1.0585	0.1753	2.8182
C	2.0763	2.1877	0.0866
H	-3.6412	-3.1046	6.0162
H	-5.6578	-3.4531	4.6302
H	-5.6977	-2.7811	2.2527
H	-1.6033	-2.0657	5.0309
H	-0.4991	-3.0976	1.7049
H	0.5820	-1.6993	1.5413
H	0.1745	-2.3145	3.1493
H	-2.3739	-0.5750	-1.1607
H	-4.0058	-2.2744	-0.8647
H	-5.3159	-2.0269	0.3086
H	-4.5541	-0.6234	-0.4616
H	-0.7357	0.4422	-2.5646
H	3.1189	2.7138	-5.3067
H	5.4194	3.5064	-4.8696

H	6.4411	3.2693	-2.6315
H	5.1589	2.2256	-0.7684
H	3.2051	-0.7828	-1.3852
H	3.8984	0.0779	0.0050
H	2.2716	-0.6083	0.1150
H	1.1971	1.8229	-5.2637
H	-0.2063	2.0865	-4.2063
H	0.3453	0.4289	-4.5766
H	-0.6756	0.0405	3.8343
H	-1.9298	0.8355	2.8604
H	-0.2800	0.6632	2.2274
H	1.3623	1.7414	0.7825
H	1.6543	3.1228	-0.2933
H	2.9897	2.4197	0.6425
H	0.2662	-0.0050	0.3071

FB Fischer base: 1,3,3-trimethyl-2-methyleneindoline (0)

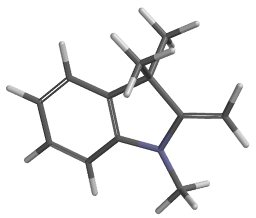

E_{elec}(au)	H°(au)	G°(au)	NImag
-520.899700	-520.657422	-520.706054	0

C	-2.0517	-0.0603	1.8989
C	-1.0155	-0.0396	1.0486
N	0.3270	-0.0362	1.4149
C	1.1523	0.0137	0.2951
C	0.3672	0.0267	-0.8654
C	-1.0995	-0.0154	-0.4823
C	2.5415	0.0477	0.2243
C	3.1302	0.0930	-1.0440
C	2.3594	0.1045	-2.2037
C	0.9614	0.0709	-2.1120
C	-1.8468	1.2336	-0.9742
C	0.7512	-0.0503	2.7921
C	-1.7801	-1.2868	-1.0143
H	-1.9226	-0.0732	2.9759
H	3.1618	0.0413	1.1149
H	4.2139	0.1202	-1.1181
H	2.8396	0.1404	-3.1767
H	0.3498	0.0801	-3.0112
H	-1.3765	2.1461	-0.5940
H	-1.8406	1.2708	-2.0692
H	-2.8883	1.2135	-0.6358
H	0.4113	0.8527	3.3145
H	1.8388	-0.0986	2.8476
H	0.3365	-0.9240	3.3089
H	-2.8194	-1.3347	-0.6710
H	-1.2583	-2.1844	-0.6669
H	-1.7775	-1.2864	-2.1101
H	-3.0654	-0.0656	1.5130

FB-H^+ Protonated FB 1,2,3,3-tetramethylindolium ion (+)

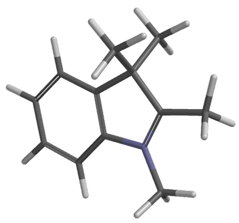

E_{elec}(au)	H°(au)	G°(au)	NImag
-521.374491	-521.118646	-521.167865	0

C	-0.9036	-0.0682	0.9382
N	0.3431	-0.0568	1.3124
C	1.2185	0.0210	0.1855
C	0.4404	0.0592	-0.9628
C	-1.0134	0.0035	-0.5661
C	2.6019	0.0557	0.1798
C	3.2108	0.1330	-1.0735
C	2.4503	0.1717	-2.2447
C	1.0547	0.1353	-2.2021
C	-1.7806	1.2759	-0.9803
C	0.8564	-0.1141	2.6769
C	-1.7135	-1.2604	-1.1063
H	3.1878	0.0258	1.0920
H	4.2936	0.1629	-1.1350
H	2.9536	0.2315	-3.2044
H	0.4681	0.1652	-3.1153
H	-1.3042	2.1742	-0.5777
H	-1.7832	1.3397	-2.0720
H	-2.8173	1.2335	-0.6345
H	1.4422	0.7878	2.8653
H	1.4935	-0.9955	2.7706
H	0.0311	-0.1752	3.3828
H	-2.7491	-1.3089	-0.7581
H	-1.1871	-2.1675	-0.7962
H	-1.7183	-1.2139	-2.1988
C	-2.0508	-0.1401	1.8724
H	-2.0371	0.7155	2.5562
H	-1.9960	-1.0546	2.4729
H	-2.9920	-0.1360	1.3226

FB-CH=CH_2 (E) (0)

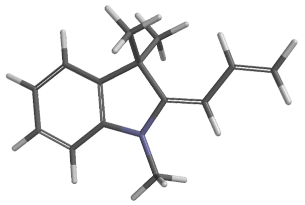

E_{elec}(au)	H°(au)	G°(au)	NImag
-598.274847	-597.998117	-598.050662	0

C	-0.4924	-0.0246	0.7460
N	0.8390	-0.0050	1.1468
C	1.6998	0.0247	0.0520
C	0.9493	0.0371	-1.1270
C	-0.5301	0.0133	-0.7901
C	3.0908	0.0415	0.0216
C	3.7154	0.0731	-1.2294
C	2.9769	0.0867	-2.4099
C	1.5774	0.0686	-2.3576
C	-1.2093	1.2911	-1.3161
C	1.2420	-0.0174	2.5319
C	-1.1852	-1.2468	-1.3832
H	3.6865	0.0296	0.9285
H	4.8008	0.0860	-1.2733
H	3.4845	0.1106	-3.3692
H	0.9922	0.0785	-3.2741
H	-0.7405	2.1826	-0.8877
H	-1.1094	1.3392	-2.4058
H	-2.2749	1.3107	-1.0718
H	0.8148	0.8413	3.0627
H	2.3277	0.0382	2.6029
H	0.9053	-0.9356	3.0282
H	-2.2510	-1.2980	-1.1450
H	-0.7023	-2.1511	-0.9997
H	-1.0813	-1.2368	-2.4736
C	-1.5190	-0.0698	1.6285
H	-1.2798	-0.0995	2.6896
C	-2.9381	-0.0834	1.3147
H	-3.2331	-0.0520	0.2687
C	-3.9112	-0.1309	2.2368
H	-3.6858	-0.1635	3.3012
H	-4.9592	-0.1383	1.9531

FB=CHCH$_3$ (E) (+)

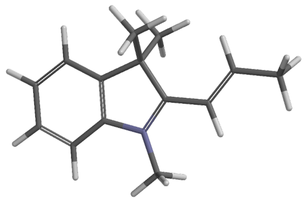

E_{elec}(au)	H°(au)	G°(au)	NImag
-598.747924	-598.458436	-598.511543	0

C	0.3378	0.0178	0.6415
N	-0.9164	0.0051	1.0458
C	-1.8189	-0.0292	-0.0558
C	-1.0694	-0.0425	-1.2236
C	0.4000	-0.0164	-0.8790
C	-3.2052	-0.0473	-0.0481
C	-3.8366	-0.0815	-1.2911
C	-3.1000	-0.0965	-2.4775
C	-1.7049	-0.0772	-2.4540
C	1.0886	-1.3007	-1.3881
C	-1.3504	0.0237	2.4429
C	1.0611	1.2550	-1.4505
H	-3.7895	-0.0356	0.8648
H	-4.9208	-0.0966	-1.3312
H	-3.6202	-0.1233	-3.4296
H	-1.1322	-0.0887	-3.3766
H	0.6243	-2.1908	-0.9542
H	0.9785	-1.3451	-2.4752
H	2.1549	-1.3090	-1.1527
H	-0.9338	-0.8382	2.9661
H	-2.4352	-0.0293	2.4795
H	-1.0193	0.9497	2.9159
H	2.1276	1.2953	-1.2195
H	0.5805	2.1555	-1.0576
H	0.9474	1.2458	-2.5381
C	1.4201	0.0591	1.5859
H	1.1551	0.0884	2.6384
C	2.7271	0.0705	1.2580
H	3.0395	0.0432	0.2187
C	3.8271	0.1245	2.2569
H	3.4536	0.1588	3.2830
H	4.4523	1.0054	2.0683
H	4.4775	-0.7501	2.1368

FB-CH=CH-CH=CH_2 (E,E) (0)

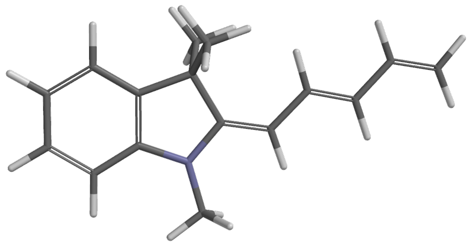

E_{elec}(au)	H°(au)	G°(au)	NImag
-675.654399	-675.343873	-675.400270	0

C	-0.2248	0.0107	0.4729
N	-1.5528	-0.0104	0.8735
C	-2.4146	-0.0619	-0.2213
C	-1.6638	-0.0736	-1.3995
C	-0.1853	-0.0272	-1.0624
C	-3.8051	-0.0997	-0.2502
C	-4.4292	-0.1508	-1.5006
C	-3.6904	-0.1630	-2.6810
C	-2.2913	-0.1237	-2.6298
C	0.5140	-1.2932	-1.5901
C	-1.9569	0.0146	2.2586
C	0.4499	1.2446	-1.6536
H	-4.4004	-0.0915	0.6570
H	-5.5142	-0.1814	-1.5442
H	-4.1977	-0.2032	-3.6399
H	-1.7062	-0.1331	-3.5463
H	0.0618	-2.1927	-1.1607
H	0.4109	-1.3420	-2.6794
H	1.5807	-1.2954	-1.3504
H	-1.5578	-0.8551	2.7934
H	-3.0439	-0.0046	2.3271
H	-1.5920	0.9237	2.7503
H	1.5147	1.3126	-1.4152
H	-0.0478	2.1404	-1.2693
H	0.3461	1.2335	-2.7440
C	0.8041	0.0562	1.3571
H	0.5645	0.0751	2.4182
C	2.2134	0.0826	1.0382
H	2.5095	0.0697	-0.0086
C	3.2001	0.1233	1.9596
H	2.9463	0.1376	3.0207
C	4.6125	0.1490	1.6144
H	6.6552	0.2045	2.1834
C	5.6169	0.1872	2.5008
H	5.4274	0.2023	3.5724
H	4.8462	0.1350	0.5487

FB=CH-CH=CH-CH_3 (E,E) (+)

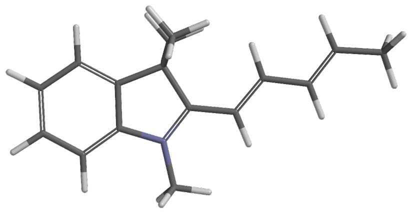

E_{elec}(au)	H°(au)	G°(au)	NImag
-676.128300	-675.804853	-675.861697	0

C	-0.1997	0.3408	-0.4040
C	0.4066	-1.0598	-0.3018
C	1.8541	-1.0142	0.2307
H	1.8958	-0.7122	1.2791
H	2.4634	-0.3230	-0.3586
H	2.2868	-2.0153	0.1478
C	-0.4859	-2.0055	0.5279
H	-0.5039	-1.7291	1.5840
H	-0.0859	-3.0204	0.4483
H	-1.5115	-2.0057	0.1476
C	0.4060	-1.4833	-1.7512
C	0.1743	-1.7214	-4.5022
C	-0.1199	-0.4607	-2.5242
C	0.8295	-2.6555	-2.3558
C	0.7080	-2.7652	-3.7428
C	-0.2541	-0.5375	-3.9015
H	1.2453	-3.4685	-1.7680
H	1.0329	-3.6744	-4.2383
H	-0.6683	0.2734	-4.4904
H	0.0898	-1.8291	-5.5787
N	-0.4719	0.6166	-1.6693
C	-1.0505	1.8292	-2.2363
H	-1.2742	2.5576	-1.4633
H	-1.9704	1.5633	-2.7612
H	-0.3359	2.2574	-2.9425
C	-0.4603	1.2567	0.6576
H	-0.9072	2.2123	0.4061
C	-0.1882	1.0195	1.9674
H	0.2598	0.0832	2.2849
C	-0.4638	1.9703	3.0139
H	-0.9120	2.9229	2.7362
C	-0.1790	1.6975	4.3007
H	0.2689	0.7329	4.5404
C	-0.4268	2.6175	5.4462
H	0.5136	2.8295	5.9691
H	-1.0899	2.1391	6.1773
H	-0.8757	3.5612	5.1249

TMP 1,3,3-trimethyl-2-phenylindolinium (+)

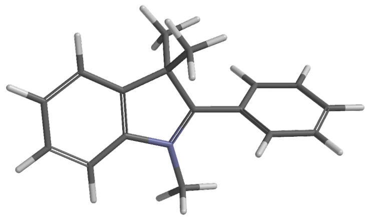

E_{elec}(au)	H°(au)	G°(au)	NImag
-713.042375	-712.732905	-712.787517	0

C	0.1630	-0.1166	0.2877
N	-1.0799	-0.1937	0.6738
C	-1.9671	-0.1808	-0.4446
C	-1.2043	-0.0927	-1.5999
C	0.2536	-0.0252	-1.2217
C	-3.3502	-0.2398	-0.4374
C	-3.9742	-0.2121	-1.6847
C	-3.2284	-0.1306	-2.8635
C	-1.8339	-0.0703	-2.8342
C	1.0753	-1.1686	-1.8446
C	-1.5809	-0.3019	2.0418
C	0.8735	1.3430	-1.5849
H	-3.9251	-0.3005	0.4802
H	-5.0572	-0.2547	-1.7362
H	-3.7436	-0.1117	-3.8185
H	-1.2597	-0.0047	-3.7534
H	0.7089	-2.1474	-1.5227
H	0.9846	-1.1013	-2.9323
H	2.1322	-1.0738	-1.5847
H	-2.2687	-1.1480	2.0835
H	-2.1080	0.6189	2.2985
H	-0.7493	-0.4641	2.7238
H	1.9094	1.3908	-1.2375
H	0.3074	2.1661	-1.1389
H	0.8591	1.4549	-2.6726
C	1.2938	0.0108	1.2157
C	3.4586	0.3636	2.9266
C	2.3280	-0.9312	1.2319
C	1.3447	1.1296	2.0572
C	2.4321	1.3056	2.9042
C	3.4019	-0.7547	2.0975
H	2.2818	-1.8091	0.5973
H	0.5459	1.8649	2.0311
H	2.4749	2.1778	3.5485
H	4.1969	-1.4929	2.1207
H	4.3049	0.5005	3.5926

TMP-H 1,3,3-trimethyl-2-phenylindoline (0)

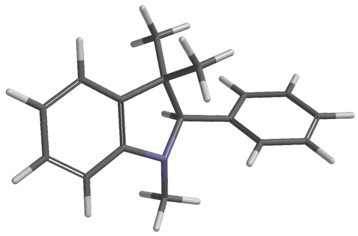

E_{elec}(au)	H°(au)	G°(au)	NImag
-713.801918	-713.483020	-713.537648	0

C	0.1397	-0.5326	0.3437
N	-1.1533	0.0292	0.7571
C	-1.9729	0.0177	-0.3761
C	-1.2050	-0.0910	-1.5427
C	0.2644	-0.1377	-1.1685
C	-3.3613	0.1032	-0.4413
C	-3.9643	0.1007	-1.7038
C	-3.2057	0.0099	-2.8682
C	-1.8102	-0.0835	-2.7862
C	1.0675	-1.1764	-1.9511
C	-1.6947	-0.4626	2.0091
C	0.8793	1.2572	-1.3684
H	-3.9685	0.1650	0.4565
H	-5.0471	0.1632	-1.7707
H	-3.6948	0.0041	-3.8376
H	-1.2106	-0.1609	-3.6902
H	0.6256	-2.1738	-1.8552
H	1.1008	-0.9158	-3.0147
H	2.1000	-1.2177	-1.5854
H	-1.9867	-1.5237	1.9478
H	-2.5692	0.1268	2.2972
H	-0.9420	-0.3508	2.7933
H	1.9335	1.2696	-1.0718
H	0.3480	2.0150	-0.7834
H	0.8157	1.5352	-2.4260
C	1.2959	-0.1104	1.2186
C	3.5185	0.6580	2.7488
C	2.3985	-0.9584	1.3497
C	1.3146	1.1232	1.8743
C	2.4163	1.5042	2.6363
C	3.5066	-0.5762	2.1028
H	2.3867	-1.9297	0.8609
H	0.4546	1.7810	1.7883
H	2.4138	2.4649	3.1434
H	4.3563	-1.2471	2.1918
H	4.3777	0.9558	3.3428
H	0.0724	-1.6355	0.3789

Section 2: Cy7 cyclization to form TMP and FB

Cy7 coiled (+)

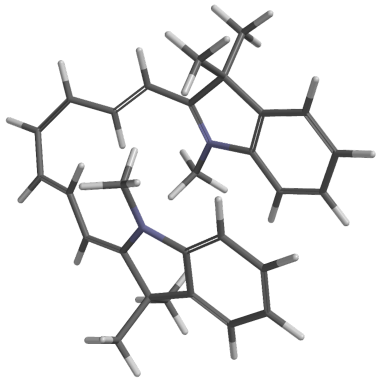

E_{elec}(au)	H°(au)	G°(au)	NImag
-1233.92072	-1233.36589	-1233.44145	0

C	1.7311	-1.4861	3.8986
C	0.9450	-2.3215	3.1068
C	0.8606	-2.1414	1.7244
C	1.5971	-1.1017	1.1787
C	2.3789	-0.2520	1.9536
C	2.4559	-0.4376	3.3227
N	1.6894	-0.7087	-0.1775
C	2.4271	0.4160	-0.3047
C	3.0337	0.7702	1.0555
C	4.5612	0.5610	0.9964
C	2.6256	1.2053	-1.4353
C	1.8556	1.1477	-2.5996
C	1.8609	2.0274	-3.6838
C	0.7783	2.0869	-4.5736
C	-0.5071	1.6094	-4.2817
C	1.1361	-1.5252	-1.2406
C	-0.9141	1.3943	-2.9630
C	-1.9603	0.5921	-2.5019
C	-2.1823	0.2573	-1.1674
N	-1.6597	0.8213	-0.0568
C	-1.9873	0.0675	1.0949
C	-2.8296	-0.9774	0.7313
C	-3.0832	-0.9103	-0.7558
C	-1.5660	0.2671	2.4001
C	-2.0400	-0.6269	3.3624
C	-2.8916	-1.6756	3.0189
C	-3.2934	-1.8593	1.6916
C	-2.7116	-2.2239	-1.4585
C	-0.9442	2.0786	0.0417
C	2.7066	2.2052	1.4932
C	-4.5516	-0.5401	-1.0524
H	1.7749	-1.6453	4.9716
H	0.3733	-3.1199	3.5692
H	0.2213	-2.7791	1.1234

H	3.0663	0.2176	3.9379
H	4.8073	-0.4568	0.6778
H	5.0182	1.2697	0.2989
H	4.9867	0.7278	1.9909
H	3.3153	2.0359	-1.3139
H	1.1260	0.3530	-2.6765
H	2.7199	2.6736	-3.8496
H	0.9467	2.5321	-5.5529
H	-1.1978	1.4108	-5.0982
H	0.1480	-1.1666	-1.5500
H	1.8138	-1.5242	-2.0962
H	1.0411	-2.5502	-0.8818
H	-0.3003	1.8890	-2.2225
H	-2.5428	0.0350	-3.2302
H	-0.8790	1.0607	2.6741
H	-1.7222	-0.5073	4.3934
H	-3.2404	-2.3599	3.7862
H	-3.9538	-2.6783	1.4202
H	-1.6805	-2.5133	-1.2377
H	-3.3739	-3.0202	-1.1046
H	-2.8306	-2.1375	-2.5425
H	0.1273	1.9450	-0.1434
H	-1.0798	2.4815	1.0455
H	-1.3567	2.7937	-0.6725
H	3.1424	2.3879	2.4805
H	1.6265	2.3614	1.5618
H	3.1257	2.9355	0.7946
H	-4.7109	-0.4379	-2.1304
H	-4.8262	0.3995	-0.5636
H	-5.2068	-1.3327	-0.6779

Cy7 *cis* cyclization TS (+)

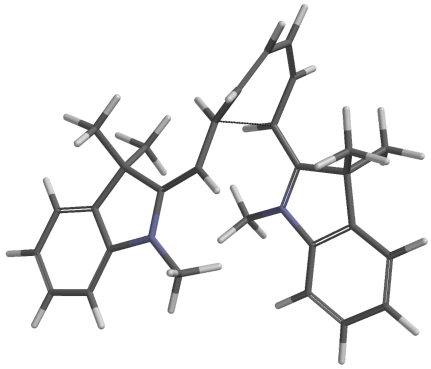

E_{elec}(au)	H°(au)	G°(au)	NImag
-1233.88889	-1233.33511	-1233.41129	1

H	0.5588	-1.4051	-3.4499
C	-0.1769	-0.6316	-3.2414
C	-2.4266	0.2995	-3.7059
C	-1.9317	0.5249	-1.2395
C	-2.7781	0.6497	-2.4556
C	0.1014	0.2445	-2.1028
C	-1.2524	-0.5004	-4.0356
H	-1.5555	1.4788	-0.8844
H	-3.7617	1.0971	-2.3126
H	-1.3291	-1.1266	-4.9213
H	-3.1386	0.4813	-4.5069
H	0.0264	1.3023	-2.3349
C	0.9783	-0.1563	-1.0830
H	1.0392	-1.2223	-0.8831
C	-2.0798	-0.4572	-0.2715
N	-1.4969	-0.3910	0.9543
C	-2.7880	-1.8106	-0.4105
C	-2.4658	-2.4591	0.9166
C	-1.6137	-3.1481	3.4630
C	-2.8080	-3.6997	1.4242
C	-1.7055	-1.5831	1.6802
C	-1.2639	-1.8925	2.9581
C	-2.3744	-4.0416	2.7098
H	-3.4053	-4.3940	0.8394
H	-0.6709	-1.1987	3.5449
H	-2.6336	-5.0106	3.1248
H	-1.2857	-3.4276	4.4595
C	-1.0082	0.7893	1.6461
C	-4.3067	-1.5889	-0.5566
H	-4.8145	-2.5583	-0.5332
H	-4.5393	-1.1038	-1.5087
H	-4.6962	-0.9729	0.2596
C	-2.2582	-2.6668	-1.5685
H	-1.1715	-2.7806	-1.5129
H	-2.5157	-2.2254	-2.5319
H	-2.7143	-3.6602	-1.5101

C	1.7510	0.6921	-0.3049
C	2.1216	2.1611	-0.5567
N	2.4253	0.2478	0.7778
C	3.1171	2.4059	0.5573
C	4.8421	2.3274	2.7221
C	3.2696	1.2450	1.3085
C	3.8245	3.5475	0.8917
C	4.6941	3.5021	1.9855
C	4.1300	1.1712	2.3940
H	3.7098	4.4606	0.3141
H	5.2597	4.3866	2.2609
H	4.2593	0.2623	2.9713
H	5.5231	2.3047	3.5673
C	2.3423	-1.1063	1.2941
H	1.2994	-1.4328	1.3013
H	2.7171	-1.1254	2.3165
H	2.9312	-1.7923	0.6773
C	0.9492	3.1507	-0.4366
C	2.8098	2.2969	-1.9299
H	3.1934	3.3158	-2.0415
H	2.1069	2.1012	-2.7445
H	3.6475	1.5986	-2.0174
H	1.3372	4.1665	-0.5607
H	0.1908	2.9932	-1.2077
H	0.4784	3.0889	0.5474
H	-1.0873	1.6631	1.0052
H	-1.6159	0.9535	2.5419
H	0.0353	0.6595	1.9461

Cy7 *trans* cyclization TS (+)

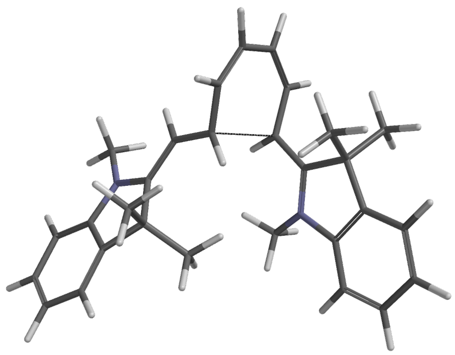

E_{elec}(au)	H°(au)	G°(au)	NImag
-1233.87638	-1233.32302	-1233.39947	1

H	-3.2778	0.1852	1.8518
C	-2.8407	0.8935	1.1462
C	-2.8457	3.2270	0.2650
C	-0.6030	2.2106	-0.3240
C	-1.6246	3.2878	-0.2809
C	-1.7311	0.2795	0.3875
C	-3.3710	2.1167	1.0531
H	-0.5054	1.7798	-1.3172
H	-1.3558	4.1999	-0.8146
H	-4.2451	2.3320	1.6626
H	-3.4911	4.0972	0.1745
C	-2.0097	-0.3648	-0.8137
H	-2.8227	0.0394	-1.4093
C	0.5337	2.1785	0.4572
N	1.6670	1.4987	0.1103
C	0.7466	2.8298	1.8290
C	2.1559	2.3882	2.1558
C	4.7315	1.3765	2.2407
C	2.9420	2.6485	3.2637
C	2.6550	1.6228	1.1056
C	3.9437	1.1071	1.1176
C	4.2425	2.1341	3.3034
H	2.5597	3.2483	4.0855
H	4.3399	0.5204	0.2958
H	4.8750	2.3306	4.1634
H	5.7444	0.9870	2.2787
C	1.9830	1.0273	-1.2266
H	2.7101	0.2179	-1.1606
H	2.4019	1.8414	-1.8292
H	1.0928	0.6322	-1.7131
C	0.6769	4.3655	1.7210
H	0.9695	4.8052	2.6801
H	-0.3390	4.6939	1.4872
H	1.3563	4.7384	0.9481
C	-0.2491	2.3222	2.8818
H	-0.2194	1.2309	2.9611

H	-1.2669	2.6285	2.6327
H	0.0127	2.7444	3.8575
C	-1.4013	-1.5415	-1.2610
C	-0.5104	-2.5172	-0.4843
N	-1.6945	-2.0690	-2.4609
C	-0.3976	-3.6573	-1.4728
C	-0.5113	-5.4254	-3.5999
C	-1.1167	-3.3483	-2.6220
C	0.2824	-4.8598	-1.3803
C	0.2198	-5.7486	-2.4566
C	-1.1959	-4.2130	-3.7042
H	0.8495	-5.1095	-0.4878
H	0.7422	-6.6984	-2.4020
H	-1.7609	-3.9772	-4.5992
H	-0.5524	-6.1260	-4.4281
C	-2.5451	-1.4246	-3.4485
H	-3.5734	-1.3632	-3.0800
H	-2.5333	-2.0042	-4.3695
H	-2.1707	-0.4195	-3.6571
C	-1.2176	-2.9926	0.8003
H	-0.6304	-3.7971	1.2534
H	-2.2180	-3.3750	0.5772
H	-1.3082	-2.1830	1.5293
C	0.8894	-1.9562	-0.1770
H	1.4715	-2.7275	0.3365
H	0.8523	-1.0737	0.4668
H	1.4080	-1.6965	-1.1025
H	-0.9193	-0.0980	1.0026

Cy7 cyclized *cis* (+)

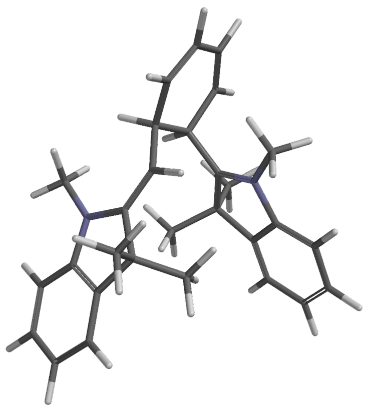

E_{elec}(au)	H°(au)	G°(au)	NImag
-1233.92525	-1233.36852	-1233.44333	0

C	3.1625	2.3270	-2.7490
C	3.1180	3.2387	-1.6917
C	2.0073	3.3135	-0.8506
C	0.9709	2.4387	-1.1264
C	0.9908	1.5249	-2.1684
C	2.0969	1.4600	-3.0006
N	-0.2584	2.2827	-0.4176
C	-0.9727	1.3160	-0.9186
C	-0.3000	0.7487	-2.1527
C	-0.0786	-0.7699	-2.0820
C	-2.3349	0.8767	-0.4465
C	-3.3467	1.9763	-0.7241
C	-4.0764	2.5407	0.2424
C	-3.9137	2.1711	1.6508
C	-3.1067	1.1674	2.0092
C	-0.5675	3.1468	0.7145
C	-2.3721	0.2893	1.0206
C	-0.9912	-0.0461	1.5390
C	-0.2324	-1.1483	1.3994
N	-0.5510	-2.3807	0.8392
C	0.5867	-3.1872	0.7437
C	1.6802	-2.5353	1.3210
C	1.2362	-1.1931	1.8603
C	0.7199	-4.4474	0.1707
C	1.9823	-5.0485	0.2038
C	3.0758	-4.4112	0.7842
C	2.9226	-3.1396	1.3499
C	1.3160	-1.1685	3.3973
C	-1.8882	-2.8370	0.5381
C	-1.1745	1.1048	-3.3806
C	2.0665	-0.0440	1.2707
H	4.0418	2.2910	-3.3843
H	3.9605	3.8998	-1.5168
H	1.9705	4.0182	-0.0270
H	2.1362	0.7523	-3.8232

H	0.5434	-1.0454	-1.2294
H	-1.0298	-1.3054	-2.0168
H	0.4260	-1.0863	-2.9991
H	-2.5868	0.0415	-1.1071
H	-3.4532	2.2855	-1.7597
H	-4.7961	3.3139	-0.0096
H	-4.4498	2.7436	2.4017
H	-2.9741	0.9074	3.0567
H	-0.6130	4.1772	0.3565
H	-1.5162	2.8656	1.1623
H	0.2327	3.0465	1.4506
H	-2.9774	-0.6170	0.9394
H	-0.4966	0.7742	2.0542
H	-0.1146	-4.9571	-0.2995
H	2.1060	-6.0321	-0.2403
H	4.0464	-4.8973	0.7946
H	3.7695	-2.6326	1.8061
H	0.7241	-1.9802	3.8322
H	2.3560	-1.2852	3.7209
H	0.9368	-0.2158	3.7834
H	-1.8692	-3.9102	0.3453
H	-2.3055	-2.3401	-0.3456
H	-2.5474	-2.6620	1.3934
H	-1.3557	2.1818	-3.4427
H	-2.1336	0.5800	-3.3407
H	-0.6443	0.7860	-4.2820
H	1.7010	0.9259	1.6243
H	2.0327	-0.0469	0.1770
H	3.1126	-0.1430	1.5793

Cy7 cyclized *trans* (+)

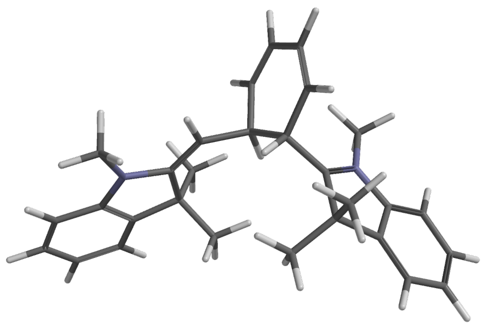

E_{elec}(au)	H°(au)	G°(au)	NImag
-1233.92404	-1233.36804	-1233.44340	0

C	5.8805	-1.5017	0.0606
C	5.8523	-0.9447	-1.2207
C	4.8250	-0.0856	-1.6113
C	3.8519	0.1739	-0.6620
C	3.8575	-0.3654	0.6162
C	4.8820	-1.2179	0.9945
N	2.7017	1.0124	-0.7899
C	2.0015	1.0176	0.3082
C	2.6460	0.1382	1.3570
C	3.0297	0.9888	2.5860
C	0.7552	1.8112	0.5539
C	1.1119	3.2837	0.6676
C	0.5607	4.2180	-0.1123
C	-0.3946	3.8798	-1.1683
C	-0.8115	2.6212	-1.3408
C	2.4547	1.7483	-2.0254
C	-0.4218	1.4711	-0.4370
C	-1.6390	1.0626	0.3545
C	-2.4008	-0.0327	0.1731
N	-3.5231	-0.2987	0.9555
C	-4.1656	-1.4616	0.5420
C	-3.4669	-2.0252	-0.5300
C	-2.2572	-1.1670	-0.8585
C	-5.3185	-2.0562	1.0442
C	-5.7562	-3.2406	0.4432
C	-5.0664	-3.8128	-0.6233
C	-3.9079	-3.1976	-1.1150
C	-0.9674	-1.9791	-0.6471
C	-3.9508	0.5626	2.0282
C	1.6998	-1.0107	1.7584
C	-2.3469	-0.6492	-2.3045
H	6.6937	-2.1662	0.3344
H	6.6410	-1.1820	-1.9267
H	4.8003	0.3496	-2.6043
H	4.9086	-1.6529	1.9889
H	3.6769	1.8240	2.3036
H	2.1377	1.3788	3.0848

H	3.5679	0.3523	3.2936
H	0.4205	1.4983	1.5486
H	1.8190	3.5477	1.4493
H	0.8273	5.2610	0.0302
H	-0.7497	4.6734	-1.8190
H	-1.5137	2.3712	-2.1315
H	3.2964	2.4221	-2.1955
H	2.3833	1.0302	-2.8427
H	1.5324	2.3196	-1.9543
H	-0.1053	0.6422	-1.0758
H	-1.9274	1.7814	1.1182
H	-5.8724	-1.6272	1.8730
H	-6.6551	-3.7191	0.8213
H	-5.4256	-4.7328	-1.0740
H	-3.3627	-3.6369	-1.9473
H	-0.8961	-2.3325	0.3856
H	-0.9676	-2.8499	-1.3106
H	-0.0749	-1.3885	-0.8727
H	-3.1501	0.6811	2.7675
H	-4.8174	0.1304	2.5264
H	-4.2222	1.5551	1.6485
H	1.4715	-1.6485	0.9013
H	2.1961	-1.6154	2.5224
H	0.7634	-0.6265	2.1746
H	-2.3954	-1.4965	-2.9970
H	-1.4728	-0.0487	-2.5735
H	-3.2436	-0.0376	-2.4443

Cy7 cyclized deprot. (0)

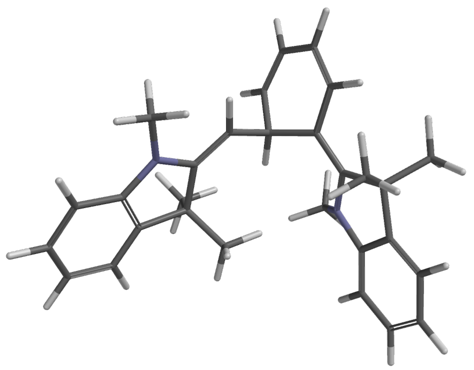

E_{elec}(au)	H°(au)	G°(au)	NImag
-1233.45455	-1232.91277	-1232.98840	0

C	5.0619	-2.1821	0.5175
C	4.6199	-2.3516	-0.7929
C	3.6398	-1.5173	-1.3396
C	3.1241	-0.5134	-0.5288
C	3.5576	-0.3313	0.7832
C	4.5304	-1.1592	1.3136
N	2.1473	0.4312	-0.8475
C	1.8340	1.2056	0.2772
C	2.8418	0.8611	1.3945
C	3.8640	1.9894	1.6380
C	0.7577	2.0335	0.3461
C	0.6479	3.1071	1.3259
C	-0.1491	4.1749	1.0874
C	-0.8875	4.2901	-0.1660
C	-0.9935	3.2364	-0.9915
C	1.9605	0.8639	-2.2208
C	-0.4702	1.8858	-0.5557
C	-1.5694	1.1969	0.2393
C	-2.1879	0.0331	-0.0202
N	-3.2065	-0.4757	0.8021
C	-3.7403	-1.6458	0.2813
C	-3.0547	-1.9922	-0.8889
C	-1.9623	-0.9740	-1.1606
C	-4.7846	-2.4338	0.7594
C	-5.1238	-3.5815	0.0354
C	-4.4449	-3.9352	-1.1280
C	-3.3969	-3.1299	-1.5934
C	-0.5853	-1.6497	-1.0268
C	-3.6707	0.2085	1.9812
C	2.1382	0.4493	2.6970
C	-2.1474	-0.3539	-2.5552
H	5.8200	-2.8446	0.9240
H	5.0358	-3.1483	-1.4026
H	3.2888	-1.6608	-2.3570
H	4.8815	-1.0175	2.3332
H	4.3458	2.2812	0.6994

H	3.3912	2.8742	2.0730
H	4.6396	1.6402	2.3287
H	1.2726	3.1059	2.2129
H	-0.1825	4.9980	1.7954
H	-1.3270	5.2500	-0.4258
H	-1.5254	3.3024	-1.9378
H	2.8867	0.6918	-2.7782
H	1.1505	0.3263	-2.7286
H	1.7393	1.9339	-2.2337
H	-0.2405	1.2866	-1.4313
H	-1.8819	1.7577	1.1170
H	-5.3296	-2.1774	1.6622
H	-5.9373	-4.2059	0.3947
H	-4.7263	-4.8309	-1.6732
H	-2.8586	-3.3975	-2.4998
H	-0.4918	-2.1391	-0.0515
H	-0.4624	-2.4101	-1.8066
H	0.2256	-0.9232	-1.1157
H	-2.8429	0.3680	2.6826
H	-4.4333	-0.3885	2.4812
H	-4.1001	1.1864	1.7278
H	1.4110	-0.3457	2.5047
H	2.8766	0.0762	3.4154
H	1.6139	1.2885	3.1616
H	-2.1185	-1.1415	-3.3162
H	-1.3587	0.3652	-2.7923
H	-3.1122	0.1581	-2.6267

Cy7 cyclized H isomerized (+)

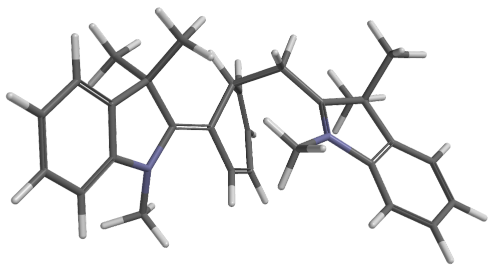

E_{elec}(au)	H°(au)	G°(au)	NImag
-1233.92970	-1233.37264	-1233.44698	0

C	3.8892	-2.9274	-4.1008
C	3.9496	-3.8643	-3.0723
C	3.5044	-3.5542	-1.7833
C	2.9995	-2.2771	-1.5675
C	2.9314	-1.3326	-2.5870
C	3.3792	-1.6453	-3.8573
N	2.5089	-1.7316	-0.3766
C	1.9781	-0.4604	-0.5992
C	2.3964	-0.0295	-2.0251
C	1.2828	0.5197	-2.9260
C	1.2487	0.2398	0.3160
C	0.8507	-0.3132	1.6132
C	0.6388	0.4936	2.6749
C	0.8956	1.9242	2.5557
C	0.9864	2.4860	1.3431
C	2.9161	-2.3020	0.8971
C	0.7669	1.6578	0.0973
C	-0.7376	1.7731	-0.3873
C	-1.8054	1.1385	0.4291
N	-2.1235	-0.1304	0.3911
C	-3.2123	-0.4187	1.2650
C	-3.6446	0.7702	1.8306
C	-2.8251	1.8980	1.2620
C	-3.7751	-1.6515	1.5480
C	-4.8313	-1.6545	2.4594
C	-5.2847	-0.4692	3.0428
C	-4.6973	0.7586	2.7315
C	-3.6879	2.7099	0.2553
C	-1.5438	-1.1923	-0.4219
C	3.5645	0.9790	-1.9324
C	-2.2534	2.8367	2.3278
H	4.2381	-3.1904	-5.0946
H	4.3446	-4.8571	-3.2697
H	3.5451	-4.2939	-0.9901
H	3.3438	-0.9089	-4.6558
H	0.4032	-0.1312	-2.9215
H	0.9815	1.5312	-2.6463
H	1.6511	0.5739	-3.9554
H	0.7055	-1.3834	1.7177

H	0.3251	0.0833	3.6297
H	0.9937	2.5219	3.4579
H	1.1565	3.5523	1.2199
H	3.8899	-2.7847	0.7662
H	3.0212	-1.5093	1.6378
H	2.2067	-3.0470	1.2707
H	1.3172	2.1310	-0.7152
H	-3.4193	-2.5708	1.0966
H	-5.3044	-2.5957	2.7189
H	-6.1070	-0.5042	3.7503
H	-5.0549	1.6779	3.1845
H	-4.0912	2.0696	-0.5338
H	-4.5209	3.1563	0.8038
H	-3.1010	3.5130	-0.1978
H	-2.3493	-1.6640	-0.9874
H	-1.0751	-1.9262	0.2356
H	-0.7980	-0.7853	-1.0959
H	3.9517	1.1818	-2.9366
H	4.3776	0.5709	-1.3243
H	3.2443	1.9268	-1.4900
H	-1.6017	3.5900	1.8790
H	-1.6885	2.2863	3.0819
H	-3.0891	3.3471	2.8155
H	-0.9638	2.8405	-0.4540
H	-0.7974	1.3671	-1.4010

Cy7 cyclized H isomerized FB release TS (+)

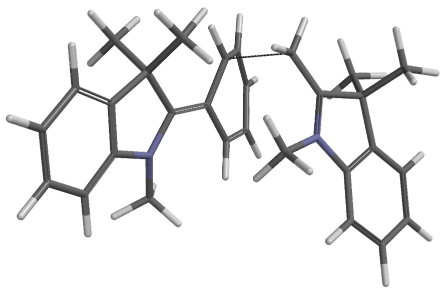

E_{elec}(au)	H°(au)	G°(au)	NImag
-1233.90844	-1233.35388	-1233.42907	1

C	3.7052	-2.9278	-3.9502
C	3.4927	-3.9168	-2.9895
C	2.9212	-3.6124	-1.7523
C	2.5777	-2.2869	-1.5254
C	2.7660	-1.2960	-2.4749
C	3.3406	-1.6031	-3.6969
N	1.9996	-1.7161	-0.3667
C	1.6921	-0.4110	-0.5566
C	2.2955	0.0258	-1.9141
C	1.3541	0.7051	-2.9205
C	1.0421	0.4149	0.3782
C	0.5039	-0.0391	1.6356
C	0.2432	0.8406	2.6469
C	0.5687	2.2234	2.4940
C	0.8520	2.7098	1.2581
C	1.9959	-2.4803	0.8757
C	0.8412	1.8235	0.1222
C	-1.0974	1.9744	-0.6638
C	-2.0354	1.2648	0.0797
N	-2.2503	-0.0723	0.0282
C	-3.1632	-0.4727	1.0199
C	-3.6604	0.6537	1.6711
C	-3.0852	1.8827	1.0080
C	-3.5405	-1.7620	1.3644
C	-4.4568	-1.8964	2.4115
C	-4.9645	-0.7800	3.0751
C	-4.5701	0.5105	2.7032
C	-4.1553	2.5103	0.0773
C	-1.6718	-1.0435	-0.8790
C	3.5412	0.9049	-1.6394
C	-2.5881	2.9385	1.9943
H	4.1523	-3.1898	-4.9038
H	3.7699	-4.9445	-3.2034
H	2.7479	-4.3922	-1.0183
H	3.5048	-0.8348	-4.4456
H	0.4432	0.1205	-3.0769
H	1.0855	1.7232	-2.6364
H	1.8729	0.7736	-3.8813

H	0.2698	-1.0828	1.7937
H	-0.1565	0.4789	3.5887
H	0.5415	2.8820	3.3572
H	1.0320	3.7667	1.0900
H	2.7802	-3.2352	0.8101
H	2.2287	-1.8208	1.7116
H	1.0364	-2.9762	1.0423
H	1.2612	2.2454	-0.7796
H	-3.1422	-2.6329	0.8543
H	-4.7720	-2.8907	2.7134
H	-5.6730	-0.9129	3.8871
H	-4.9710	1.3807	3.2159
H	-4.5188	1.7828	-0.6557
H	-5.0034	2.8513	0.6797
H	-3.7334	3.3683	-0.4561
H	-2.4764	-1.6196	-1.3455
H	-1.0095	-1.7297	-0.3422
H	-1.1061	-0.5374	-1.6576
H	4.0584	1.0861	-2.5867
H	4.2308	0.3963	-0.9594
H	3.2708	1.8694	-1.2034
H	-2.0389	3.7342	1.4818
H	-1.9412	2.5011	2.7553
H	-3.4520	3.3903	2.4928
H	-1.2161	3.0544	-0.6636
H	-0.7930	1.5595	-1.6174

Cy7 cyclized deprotonated oxidized (+)

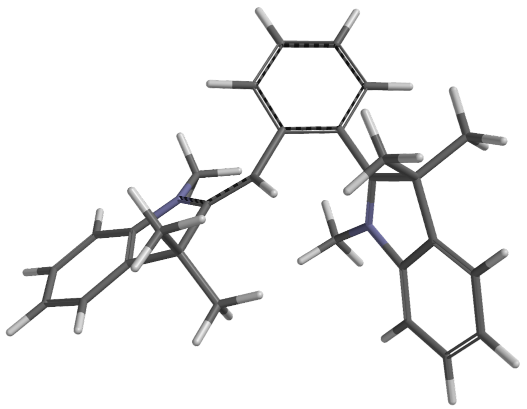

Eelec(au)	H°(au)	G°(au)	NImag
-1232.75929	-1232.22529	-1232.29935	0

C	-5.3126	-0.3375	1.5991
C	-5.1027	0.6258	0.6087
C	-3.9949	0.5607	-0.2367
C	-3.1311	-0.5019	-0.0334
C	-3.3122	-1.4648	0.9493
C	-4.4183	-1.3942	1.7804
N	-1.9339	-0.8140	-0.7443
C	-1.3550	-1.8745	-0.2524
C	-2.1654	-2.4423	0.8966
C	-2.6364	-3.8824	0.6123
C	-0.0529	-2.3784	-0.7133
C	0.0278	-3.6317	-1.3299
C	1.2537	-4.1117	-1.7702
C	2.4045	-3.3529	-1.5586
C	2.3287	-2.1190	-0.9256
C	-1.4698	-0.0036	-1.8637
C	1.1006	-1.5874	-0.5066
C	1.0003	-0.3142	0.2162
C	1.5998	0.8768	-0.0070
N	2.3717	1.2966	-1.0749
C	2.8725	2.5834	-0.8426
C	2.3769	3.0780	0.3647
C	1.4586	2.0462	0.9823
C	3.7320	3.3286	-1.6399
C	4.0845	4.6067	-1.1927
C	3.5952	5.1134	0.0090
C	2.7302	4.3429	0.7969
C	1.9099	1.6512	2.3950
C	2.4853	0.6577	-2.3686
C	-1.3223	-2.4069	2.1900
C	0.0047	2.5529	1.0033
H	-6.1850	-0.2607	2.2402
H	-5.8108	1.4391	0.4914
H	-3.8271	1.3081	-1.0042
H	-4.5836	-2.1378	2.5544

H	-3.1678	-3.9504	-0.3413
H	-1.7881	-4.5714	0.6062
H	-3.3160	-4.1838	1.4142
H	-0.8732	-4.2114	-1.4995
H	1.3113	-5.0748	-2.2663
H	3.3708	-3.7326	-1.8768
H	3.2352	-1.5551	-0.7296
H	-2.3250	0.2223	-2.5015
H	-1.0320	0.9174	-1.4754
H	-0.7279	-0.5645	-2.4276
H	0.3501	-0.3270	1.0858
H	4.1290	2.9393	-2.5719
H	4.7562	5.2095	-1.7971
H	3.8858	6.1067	0.3371
H	2.3422	4.7345	1.7340
H	2.9368	1.2733	2.3862
H	1.8662	2.5215	3.0587
H	1.2574	0.8741	2.8078
H	2.4687	1.4258	-3.1469
H	1.6366	-0.0069	-2.5264
H	3.4093	0.0760	-2.4650
H	-1.0483	-1.3835	2.4614
H	-1.9170	-2.8301	3.0044
H	-0.4145	-3.0052	2.0687
H	-0.0759	3.4298	1.6547
H	-0.6692	1.7766	1.3825
H	-0.3238	2.8376	-0.0018

Section 3: FB+Cy7 reaction at C2'

Cy7+FB C2' attack TS (+)

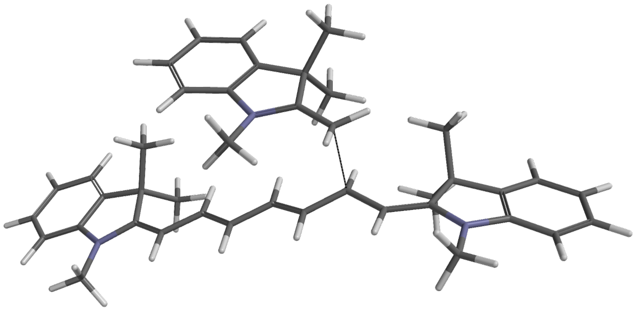

E_{elec}(au)	H°(au)	G°(au)	NImag
-1754.82522	-1754.02688	-1754.12449	1

C	7.3094	-5.4906	1.2117
C	7.8024	-5.5779	-0.0886
C	7.1497	-4.9570	-1.1580
C	5.9876	-4.2482	-0.8777
C	5.4833	-4.1542	0.4201
C	6.1368	-4.7716	1.4708
N	5.1485	-3.5411	-1.7480
C	4.0950	-2.9672	-1.0761
C	4.2130	-3.3267	0.4122
C	4.3874	-2.0739	1.2879
C	3.1446	-2.2144	-1.7062
C	2.0086	-1.6066	-1.0860
C	1.0704	-0.8746	-1.7488
C	-0.0569	-0.2955	-1.0835
C	-1.0009	0.4768	-1.6860
C	5.3472	-3.4160	-3.1755
C	-2.1246	1.0443	-0.9921
C	-3.2221	1.5524	-1.7579
C	-4.5252	1.6837	-1.3533
N	-5.4909	2.1979	-2.1767
C	-6.7627	2.1305	-1.5850
C	-6.6401	1.5838	-0.3093
C	-5.1836	1.2811	-0.0261
C	-7.9931	2.5238	-2.0957
C	-9.1137	2.3570	-1.2763
C	-9.0033	1.8148	0.0029
C	-7.7535	1.4236	0.4950
C	-4.6996	2.1703	1.1316
C	-5.2283	2.7075	-3.5066
C	3.0254	-4.1823	0.8913
C	-4.9920	-0.2115	0.2928
H	7.8347	-5.9812	2.0253
H	8.7127	-6.1377	-0.2829
H	7.5509	-5.0364	-2.1629
H	5.7456	-4.6993	2.4826
H	5.2206	-1.4612	0.9294

H	3.4878	-1.4540	1.2936
H	4.5986	-2.3719	2.3204
H	3.2388	-2.0631	-2.7789
H	1.8646	-1.7358	-0.0159
H	1.1704	-0.7100	-2.8215
H	-0.1548	-0.5070	-0.0182
H	-0.9005	0.7095	-2.7456
H	4.4807	-3.8101	-3.7176
H	5.4915	-2.3665	-3.4547
H	6.2294	-3.9811	-3.4739
H	-2.9779	1.8515	-2.7738
H	-8.1000	2.9452	-3.0896
H	-10.0878	2.6583	-1.6503
H	-9.8887	1.6959	0.6197
H	-7.6588	1.0022	1.4926
H	-4.8369	3.2304	0.8967
H	-5.2697	1.9366	2.0363
H	-3.6447	1.9987	1.3453
H	-6.1264	3.1836	-3.8980
H	-4.9315	1.8984	-4.1830
H	-4.4286	3.4537	-3.4704
H	3.2109	-4.5200	1.9162
H	2.9011	-5.0638	0.2548
H	2.0900	-3.6166	0.8854
H	-3.9519	-0.4478	0.5315
H	-5.3040	-0.8324	-0.5524
H	-5.6028	-0.4777	1.1616
H	-2.3219	0.6454	-0.0040
C	-1.2743	2.8828	-0.1627
H	-1.1221	3.3187	-1.1429
H	-2.1632	3.2151	0.3591
C	-0.1922	2.5041	0.6055
C	-0.2216	2.2247	2.1124
C	-0.6234	3.5017	2.8710
H	-1.6555	3.7786	2.6322
H	0.0327	4.3379	2.6105
H	-0.5541	3.3267	3.9494
C	-1.1555	1.0647	2.4969
H	-2.1992	1.3091	2.2880
H	-1.0671	0.8774	3.5718
H	-0.8960	0.1428	1.9691
N	1.0506	2.2460	0.1324
C	1.5647	2.5300	-1.1957
H	0.7470	2.7589	-1.8754
H	2.0925	1.6496	-1.5727
H	2.2560	3.3781	-1.1511
C	1.2154	1.8387	2.3747
C	1.9187	1.8583	1.1732
C	1.8569	1.4874	3.5484
H	1.3161	1.4651	4.4908
C	3.2186	1.1681	3.4986
H	3.7409	0.8954	4.4104
C	3.9100	1.2023	2.2886
H	4.9661	0.9514	2.2666

C	3.2676	1.5460	1.0947
H	3.8104	1.5666	0.1556

Cy7+FB C2' adduct (+)

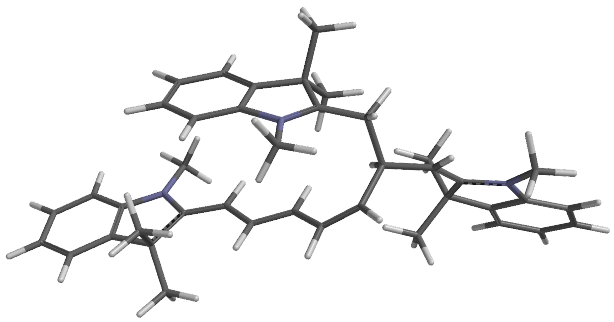

E_{elec}(au)	H°(au)	G°(au)	NImag
-1754.85725	-1754.05451	-1754.15003	0

C	2.5683	4.5333	6.6098
C	3.6991	3.9185	7.1416
C	4.1926	2.7186	6.6184
C	3.5156	2.1508	5.5429
C	2.3810	2.7623	5.0005
C	1.9014	3.9463	5.5267
N	3.7859	0.9772	4.8474
C	2.8816	0.7870	3.7991
C	1.8490	1.9272	3.8502
C	1.8278	2.7530	2.5529
C	2.9838	-0.2359	2.9319
C	2.1091	0.3935	-0.6232
C	0.6726	0.5434	-0.7762
C	4.9007	0.0952	5.0846
C	0.0598	0.6509	-1.9753
C	-1.3733	0.6542	-2.1378
C	-2.0520	0.6462	-3.3125
N	-3.4343	0.5728	-3.3723
C	-3.8888	0.6092	-4.6902
C	-2.7942	0.6992	-5.5542
C	-1.5098	0.7256	-4.7472
C	-5.1914	0.5648	-5.1754
C	-5.3708	0.6106	-6.5614
C	-4.2870	0.6978	-7.4320
C	-2.9835	0.7426	-6.9225
C	-0.6378	-0.4884	-5.1160
C	-4.2718	0.5220	-2.1981
C	0.4376	1.4166	4.1918
C	-0.7540	2.0442	-4.9921
H	2.2045	5.4651	7.0319
H	4.2162	4.3761	7.9803
H	5.0771	2.2593	7.0478
H	1.0172	4.4180	5.1039
H	2.8335	3.1006	2.2961
H	1.4400	2.1733	1.7106
H	1.1829	3.6284	2.6860
H	3.8201	-0.9249	3.0356

H	0.0513	0.4913	0.1159
H	5.4344	0.4037	5.9834
H	4.5492	-0.9333	5.2278
H	5.6001	0.1072	4.2388
H	-1.9466	0.5942	-1.2151
H	-6.0501	0.4977	-4.5155
H	-6.3805	0.5768	-6.9605
H	-4.4521	0.7320	-8.5044
H	-2.1313	0.8109	-7.5944
H	-1.1926	-1.4194	-4.9711
H	-0.3460	-0.4219	-6.1696
H	0.2729	-0.5346	-4.5128
H	-5.3160	0.4281	-2.4935
H	-4.1556	1.4321	-1.5979
H	-4.0067	-0.3416	-1.5781
H	0.0090	0.8423	3.3670
H	0.4554	0.7837	5.0847
H	-0.2247	2.2676	4.3841
H	-0.4867	2.1240	-6.0513
H	0.1666	2.0943	-4.4040
H	-1.3791	2.9030	-4.7282
C	1.7563	-2.0520	1.7458
H	0.6899	0.6948	-2.8608
C	0.6618	-2.3955	0.8043
N	0.8006	-2.6572	-0.4666
C	-0.7948	-2.5185	1.2069
C	-1.4559	-2.7032	-0.1331
C	-2.0751	-3.1363	-2.7997
C	-0.4683	-2.8252	-1.0989
C	-2.7885	-2.7975	-0.4988
C	-3.0880	-3.0105	-1.8467
C	-0.7303	-3.0462	-2.4394
H	-3.5793	-2.7051	0.2396
H	-4.1253	-3.0769	-2.1595
H	0.0570	-3.1332	-3.1800
H	-2.3353	-3.3026	-3.8401
C	-1.3406	-1.3440	2.0252
H	-1.2948	-0.4019	1.4734
H	-0.7913	-1.2412	2.9653
H	-2.3879	-1.5456	2.2676
C	-0.9134	-3.8287	2.0341
H	-0.5076	-4.6843	1.4867
H	-1.9727	-4.0105	2.2346
H	-0.3896	-3.7304	2.9891
C	2.0284	-2.8029	-1.2405
H	2.0335	-3.8044	-1.6758
H	2.0293	-2.0514	-2.0318
H	2.8960	-2.6624	-0.6029
C	2.0872	-0.5140	1.7532
H	2.7096	0.6272	-1.5027
C	2.7426	-0.0816	0.4623
H	3.8238	-0.2038	0.4261
H	1.4341	-2.3278	2.7535
H	2.6669	-2.6156	1.5224

H	1.1420	0.0159	1.8782

Cy7+FB C2' adduct C1' deprot. (0)

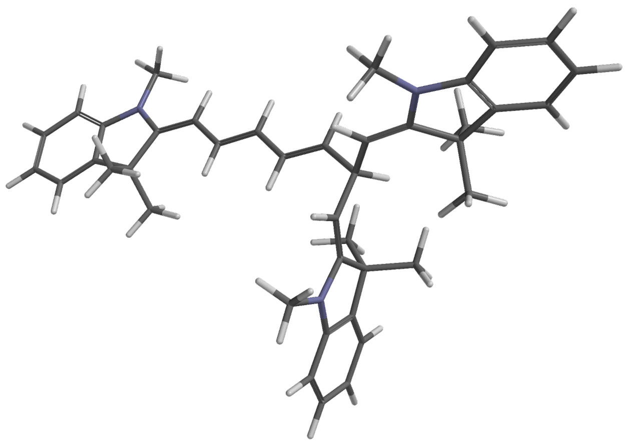

E_{elec}(au)	H°(au)	G°(au)	NImag
-1754.36997	-1753.58399	-1753.68148	0

C	6.5050	5.2339	5.1544
C	5.9191	5.1290	6.4137
C	4.7090	4.4540	6.6024
C	4.1033	3.8847	5.4866
C	4.6830	3.9835	4.2184
C	5.8791	4.6536	4.0440
N	2.9126	3.1679	5.3958
C	2.6599	2.7679	4.0891
C	3.8073	3.2750	3.2015
C	4.5876	2.1212	2.5457
C	1.5597	2.0549	3.7428
C	1.2168	1.5865	2.4174
C	0.1076	0.8735	2.1274
C	-0.2066	0.3956	0.7913
C	-1.2874	-0.3307	0.4758
C	2.0322	2.8615	6.4967
C	-1.6625	-0.8272	-0.9094
C	-0.6222	-0.5469	-1.9705
C	0.3967	-1.3360	-2.3547
N	1.2906	-0.9634	-3.3714
C	2.3303	-1.8753	-3.4902
C	2.1241	-2.9375	-2.6035
C	0.8394	-2.7147	-1.8259
C	3.4450	-1.8438	-4.3244
C	4.3478	-2.9098	-4.2518
C	4.1481	-3.9736	-3.3758
C	3.0211	-3.9863	-2.5434
C	-0.1673	-3.8286	-2.1657
C	1.2082	0.3017	-4.0546
C	3.3097	4.2774	2.1440
C	1.1198	-2.7039	-0.3153
H	7.4450	5.7634	5.0333
H	6.4069	5.5802	7.2732
H	4.2714	4.3849	7.5929

H	6.3280	4.7297	3.0565
H	4.9330	1.4091	3.3018
H	3.9765	1.5817	1.8174
H	5.4636	2.5212	2.0237
H	0.8520	1.7941	4.5272
H	1.8923	1.8141	1.5955
H	-0.5946	0.6263	2.9254
H	0.5104	0.6365	0.0055
H	-2.0117	-0.5758	1.2549
H	1.0412	3.3021	6.3351
H	1.9164	1.7771	6.6088
H	2.4442	3.2635	7.4218
H	-0.7251	0.4210	-2.4560
H	3.6233	-1.0239	-5.0127
H	5.2233	-2.8996	-4.8953
H	4.8632	-4.7895	-3.3361
H	2.8563	-4.8116	-1.8543
H	-0.4210	-3.8190	-3.2305
H	0.2707	-4.8035	-1.9249
H	-1.0891	-3.7264	-1.5862
H	1.9670	0.3514	-4.8355
H	1.3600	1.1394	-3.3607
H	0.2244	0.4179	-4.5238
H	4.1654	4.7054	1.6108
H	2.7554	5.0942	2.6168
H	2.6570	3.7972	1.4098
H	1.8180	-1.9038	-0.0508
H	1.5603	-3.6621	-0.0176
H	0.2010	-2.5551	0.2572
H	-1.8136	-1.9063	-0.8287
C	-2.9758	-0.1588	-1.2758
C	-4.1384	-0.7261	-1.6372
C	-4.4722	-2.2077	-1.8732
C	-3.6575	-2.7524	-3.0599
H	-2.5844	-2.6740	-2.8703
H	-3.8826	-2.1859	-3.9694
H	-3.9050	-3.8049	-3.2382
C	-4.2860	-3.0771	-0.6184
H	-3.2331	-3.1807	-0.3433
H	-4.6815	-4.0816	-0.8045
H	-4.8236	-2.6482	0.2331
N	-5.2914	0.0229	-1.9130
C	-5.3367	1.4573	-1.7923
H	-6.3400	1.8172	-2.0193
H	-4.6317	1.9300	-2.4874
H	-5.0754	1.7688	-0.7732
C	-5.9423	-2.1343	-2.2453
C	-6.3563	-0.7972	-2.2593
C	-6.8283	-3.1470	-2.5585
H	-6.5021	-4.1845	-2.5486
C	-8.1506	-2.8229	-2.8902
H	-8.8576	-3.6087	-3.1381
C	-8.5532	-1.4900	-2.9013
H	-9.5789	-1.2400	-3.1587

C	-7.6657	-0.4554	-2.5874
H	-8.0028	0.5760	-2.6036
H	-2.9352	0.9272	-1.2113

Cy3+FBCH=CH-CH=CH_2 C2' adduct (+)

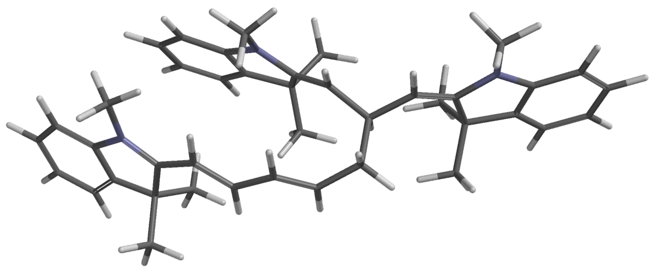

E_{elec}(au)	H°(au)	G°(au)	NImag
-1754.84877	-1754.04725	-1754.14328	0

C	-0.0899	-3.0123	-7.5896
C	1.3059	-3.0143	-7.5511
C	2.0051	-2.1991	-6.6607
C	1.2423	-1.4003	-5.8233
C	-0.1437	-1.3799	-5.8477
C	-0.8299	-2.1908	-6.7366
N	1.6878	-0.4957	-4.8250
C	0.6699	0.0840	-4.2074
C	-0.6460	-0.3966	-4.8179
C	-1.5698	-1.1063	-3.8110
C	0.8580	1.0122	-3.1445
C	-0.1351	1.5577	-2.3920
C	0.1528	2.2857	-1.1872
C	-0.7613	2.4209	-0.2074
C	-0.3904	2.7562	1.1962
C	3.1123	-0.2556	-4.6283
C	-0.0163	1.4484	1.9996
C	0.3248	1.8230	3.4198
C	-0.4581	1.7244	4.5076
N	-0.0274	2.1179	5.7827
C	-1.0507	2.0177	6.7150
C	-2.1914	1.4869	6.1030
C	-1.9029	1.2074	4.6383
C	-1.0528	2.3579	8.0654
C	-2.2285	2.1437	8.7920
C	-3.3666	1.6095	8.1934
C	-3.3457	1.2767	6.8323
C	-1.9998	-0.3052	4.3705
C	1.2834	2.6603	6.0318
C	-1.3652	0.7817	-5.5109
C	-2.8869	1.9721	3.7381
H	-0.6064	-3.6573	-8.2933
H	1.8614	-3.6589	-8.2246
H	3.0891	-2.1993	-6.6377
H	-1.9155	-2.1889	-6.7704
H	-1.0251	-1.8723	-3.2539
H	-2.0200	-0.4081	-3.1033
H	-2.3815	-1.5866	-4.3651
H	1.8777	1.2405	-2.8542

H	-1.1820	1.3776	-2.6162
H	1.1911	2.5479	-0.9949
H	-1.7909	2.1168	-0.3959
H	3.4719	0.4126	-5.4145
H	3.3018	0.1789	-3.6509
H	3.6349	-1.2102	-4.6852
H	-0.1796	2.7797	8.5523
H	-2.2472	2.4044	9.8467
H	-4.2679	1.4527	8.7777
H	-4.2293	0.8593	6.3549
H	-1.2703	-0.8578	4.9710
H	-3.0025	-0.6630	4.6292
H	-1.8257	-0.5337	3.3157
H	1.4240	2.8104	7.1022
H	1.4226	3.6214	5.5196
H	2.0545	1.9662	5.6777
H	-2.2370	0.3928	-6.0448
H	-0.7059	1.2746	-6.2311
H	-1.7078	1.5214	-4.7831
H	-2.7211	1.7443	2.6809
H	-2.7896	3.0529	3.8805
H	-3.9144	1.6827	3.9843
H	-0.8843	0.7917	1.9846
C	1.1619	0.7905	1.3291
C	1.2132	-0.3334	0.5971
C	0.1751	-1.4547	0.3914
C	0.1504	-2.3354	1.6577
H	-0.1908	-1.7534	2.5194
H	1.1498	-2.7237	1.8798
H	-0.5280	-3.1836	1.5143
C	-1.2462	-1.0087	0.0300
H	-1.7563	-0.5208	0.8638
H	-1.8400	-1.8874	-0.2436
H	-1.2411	-0.3257	-0.8210
N	2.3706	-0.7075	-0.1143
C	3.5236	0.1508	-0.2211
H	3.9234	0.3744	0.7731
H	3.2757	1.0994	-0.7179
H	4.3049	-0.3529	-0.7908
C	0.7984	-2.2267	-0.7592
C	2.0878	-1.7402	-0.9982
C	0.2927	-3.2665	-1.5161
H	-0.7067	-3.6511	-1.3275
C	1.0714	-3.8091	-2.5482
H	0.6756	-4.6109	-3.1635
C	2.3511	-3.3138	-2.7810
H	2.9532	-3.7306	-3.5838
C	2.8845	-2.2812	-2.0030
H	3.8866	-1.9143	-2.1981
H	2.0596	1.4076	1.3562
H	1.3185	2.2513	3.5402
H	0.4839	3.4179	1.2165
H	-1.2115	3.2583	1.7157

Cy3+FB-CH=CH-CH=CH_2 C2' adduct cleavage TS (+)

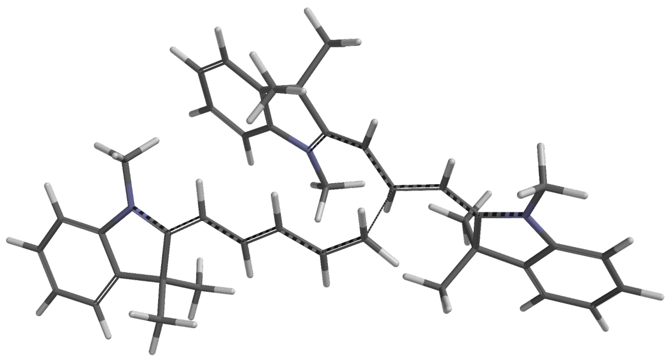

E_{elec}(au)	H°(au)	G°(au)	NImag
-1754.81653	-1754.01754	-1754.11482	1

C	3.5758	1.8968	-1.1669
N	4.3228	1.9090	-2.2977
C	4.9792	3.1438	-2.4692
C	4.6191	3.9912	-1.4249
C	3.6646	3.2722	-0.4962
C	5.8579	3.5398	-3.4685
C	6.3724	4.8370	-3.3911
C	6.0184	5.6972	-2.3525
C	5.1321	5.2743	-1.3573
C	4.2766	3.1687	0.9138
C	4.4663	0.7718	-3.1867
C	2.3061	4.0000	-0.4752
H	6.1495	2.8815	-4.2797
H	7.0643	5.1752	-4.1566
H	6.4359	6.6985	-2.3138
H	4.8560	5.9388	-0.5428
H	5.2365	2.6442	0.8807
H	4.4459	4.1748	1.3100
H	3.6156	2.6372	1.6029
H	3.4810	0.3705	-3.4380
H	5.0693	-0.0119	-2.7170
H	4.9499	1.0915	-4.1087
H	1.5877	3.5090	0.1853
H	1.8771	4.0492	-1.4805
H	2.4546	5.0215	-0.1112
C	2.9179	0.7633	-0.7246
H	3.0605	-0.1627	-1.2753
C	2.0801	0.7105	0.4016
H	1.8256	1.6342	0.9152
C	1.5294	-0.4486	0.9159
H	1.8050	-1.4086	0.4883
C	0.6027	-0.4217	1.9667
H	-0.6732	-1.4247	3.3434
C	-0.0881	-1.5292	2.4368
H	0.3079	-2.5227	2.2451
H	0.3206	0.5579	2.3562

C	1.6047	-0.5433	-5.2113
C	1.0937	0.6889	-4.8057
C	0.3488	0.8159	-3.6304
C	0.1277	-0.3344	-2.8825
C	0.6386	-1.5713	-3.2732
C	1.3773	-1.6868	-4.4367
N	-0.5975	-0.4804	-1.6897
C	-0.5178	-1.7667	-1.2190
C	0.2356	-2.6160	-2.2583
C	1.4657	-3.3088	-1.6535
C	-1.0379	-2.3245	-0.0830
C	-1.3118	0.6134	-1.0736
C	-1.6677	-1.7235	1.0615
C	-2.5543	-2.5358	1.8642
C	-3.5484	-2.0696	2.6718
N	-4.2852	-2.8971	3.4885
C	-5.2632	-2.1851	4.1922
C	-5.1875	-0.8364	3.8434
C	-4.0898	-0.6383	2.8161
C	-6.1965	-2.6469	5.1127
C	-7.0626	-1.7085	5.6821
C	-6.9981	-0.3595	5.3401
C	-6.0508	0.0834	4.4098
C	-4.7059	-0.1489	1.4918
C	-4.0330	-4.3148	3.6193
C	-0.7077	-3.6598	-2.8845
C	-3.0405	0.3565	3.3418
H	2.1851	-0.6149	-6.1257
H	1.2825	1.5736	-5.4066
H	-0.0249	1.7867	-3.3228
H	1.7746	-2.6506	-4.7445
H	2.1750	-2.5772	-1.2561
H	1.1759	-3.9913	-0.8480
H	1.9769	-3.8901	-2.4281
H	-0.8957	-3.4005	-0.0066
H	-1.4691	1.4010	-1.8114
H	-2.2954	0.2723	-0.7446
H	-0.7592	1.0278	-0.2227
H	-1.9020	-0.6715	1.0094
H	-2.3554	-3.6043	1.8511
H	-6.2655	-3.6932	5.3907
H	-7.7997	-2.0457	6.4051
H	-7.6838	0.3493	5.7939
H	-5.9956	1.1341	4.1363
H	-3.9479	-0.0322	0.7129
H	-5.4618	-0.8540	1.1325
H	-5.1849	0.8234	1.6474
H	-4.6702	-4.7303	4.3989
H	-2.9870	-4.4887	3.8942
H	-4.2421	-4.8361	2.6787
H	-0.1848	-4.1968	-3.6831
H	-1.5949	-3.1812	-3.3106
H	-1.0299	-4.3885	-2.1337
H	-2.2543	0.5513	2.6076

H	-2.5753	-0.0144	4.2603
H	-3.5290	1.3107	3.5642

Section 4: FB+Cy7 reaction at C4’

Cy7+FB C4' attack TS (+)

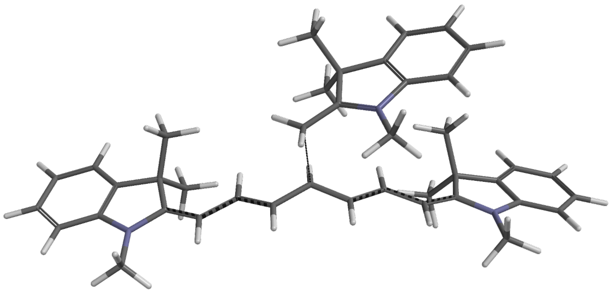

E_{elec}(au)	H°(au)	G°(au)	NImag
-1754.82742	-1754.02865	-1754.12608	1

C	7.8900	-2.6060	-1.7978
C	8.2545	-3.1603	-0.5722
C	7.3605	-3.2038	0.5014
C	6.0920	-2.6753	0.2993
C	5.7145	-2.1171	-0.9209
C	6.6073	-2.0766	-1.9764
N	5.0142	-2.5907	1.1930
C	3.9368	-1.9712	0.6220
C	4.2855	-1.6205	-0.8318
C	3.3910	-2.3959	-1.8181
C	2.7574	-1.7849	1.2967
C	1.5894	-1.1702	0.7673
C	0.3775	-1.1406	1.3982
C	-0.7586	-0.4945	0.8262
C	-2.0962	-0.8287	1.2425
C	5.0234	-3.0937	2.5501
C	-3.1777	-0.5344	0.4728
C	-4.5294	-0.8478	0.8091
C	-5.6261	-0.5521	0.0484
N	-6.8999	-0.9211	0.4059
C	-7.8483	-0.4306	-0.4996
C	-7.1934	0.2473	-1.5270
C	-5.6981	0.2215	-1.2777
C	-9.2325	-0.5471	-0.4667
C	-9.9531	0.0315	-1.5158
C	-9.3104	0.7036	-2.5535
C	-7.9152	0.8154	-2.5606
C	-4.9878	-0.5290	-2.4204
C	-7.2260	-1.6802	1.5934
C	4.2404	-0.1090	-1.1127
C	-5.1655	1.6595	-1.1352
H	8.6035	-2.5830	-2.6156
H	9.2529	-3.5671	-0.4410
H	7.6642	-3.6333	1.4500
H	6.3173	-1.6410	-2.9290
H	3.4189	-3.4696	-1.6083
H	3.7520	-2.2333	-2.8388

H	2.3520	-2.0588	-1.7655
H	2.6804	-2.1639	2.3126
H	1.6455	-0.7036	-0.2125
H	0.2663	-1.6021	2.3783
H	-0.6564	-0.1660	-0.2052
H	-2.2235	-1.3178	2.2072
H	4.9863	-2.2686	3.2708
H	4.1614	-3.7484	2.7134
H	5.9300	-3.6745	2.7189
H	-4.6798	-1.3624	1.7547
H	-9.7494	-1.0585	0.3384
H	-11.0365	-0.0454	-1.5141
H	-9.8927	1.1454	-3.3560
H	-7.4075	1.3425	-3.3645
H	-3.9064	-0.5730	-2.2672
H	-5.3674	-1.5519	-2.5061
H	-5.1758	-0.0108	-3.3665
H	-6.5066	-2.4936	1.7224
H	-7.2138	-1.0464	2.4881
H	-8.2171	-2.1216	1.4807
H	4.5948	0.0779	-2.1317
H	4.8829	0.4367	-0.4179
H	3.2300	0.2974	-1.0278
H	-5.3553	2.2104	-2.0623
H	-4.0892	1.6793	-0.9449
H	-5.6707	2.1797	-0.3155
H	-2.9798	-0.0290	-0.4695
C	-0.6719	1.5517	1.5943
H	-1.5106	1.9452	1.0306
H	-0.8908	1.2405	2.6087
H	4.4860	1.7517	2.5707
C	4.1711	2.2459	1.6577
C	3.3468	3.5353	-0.7234
C	2.8432	2.2770	1.2604
C	5.0967	2.8842	0.8266
C	4.6960	3.5143	-0.3505
C	2.4216	2.9166	0.0977
H	6.1462	2.8820	1.1047
H	5.4354	3.9986	-0.9806
H	3.0324	4.0374	-1.6343
N	1.7292	1.7211	1.9170
C	0.5840	2.0024	1.2532
C	0.9159	2.8153	0.0006
C	0.2641	4.2075	0.1123
H	0.5693	4.8241	-0.7392
H	-0.8270	4.1184	0.1032
H	0.5677	4.7118	1.0348
C	0.4573	2.1242	-1.2920
H	0.7322	2.7442	-2.1512
H	0.9291	1.1449	-1.4149
H	-0.6303	1.9955	-1.2974
C	1.8652	1.1101	3.2256
H	2.5811	0.2849	3.1672
H	2.2267	1.8532	3.9437

H	0.9070	0.7188	3.5592

Cy7+FB C4' adduct (+)

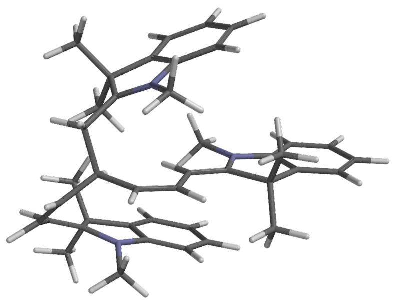

E_{elec}(au)	H°(au)	G°(au)	NImag
-1754.86145	-1754.05837	-1754.15331	0

C	-2.8745	-3.4072	-0.1944
C	-3.3731	-2.1222	-0.3989
C	-2.7708	-1.2466	-1.3069
C	-1.6516	-1.6966	-1.9963
C	-1.1463	-2.9853	-1.8053
C	-1.7539	-3.8475	-0.9106
N	-0.8647	-1.0018	-2.9185
C	0.2424	-1.7751	-3.2752
C	0.0281	-3.1995	-2.7397
C	-0.3952	-4.1169	-3.9060
C	1.3023	-1.3814	-4.0088
C	1.6608	-0.0350	-4.4837
C	1.7733	1.0918	-3.7654
C	1.5799	1.2195	-2.2767
C	0.5418	2.2532	-1.9295
C	-1.3912	0.0730	-3.7373
C	-0.3718	2.1623	-0.9445
C	-0.5366	1.0510	-0.0320
C	-1.4138	0.9688	0.9985
N	-1.5006	-0.1476	1.8147
C	-2.4466	0.0309	2.8212
C	-3.0451	1.2850	2.6770
C	-2.4445	1.9984	1.4815
C	-2.8156	-0.8317	3.8482
C	-3.8134	-0.4039	4.7298
C	-4.4196	0.8427	4.5921
C	-4.0303	1.6977	3.5535
C	-3.5338	2.2510	0.4234
C	-0.6469	-1.3017	1.6756
C	1.2509	-3.7866	-2.0321
C	-1.7827	3.3135	1.9284
H	-3.3523	-4.0670	0.5233
H	-4.2364	-1.7825	0.1666
H	-3.1488	-0.2391	-1.4423
H	-1.3673	-4.8532	-0.7649
H	-1.2582	-3.7004	-4.4357
H	0.4297	-4.2288	-4.6182
H	-0.6669	-5.1079	-3.5261

H	1.9867	-2.1730	-4.3113
H	1.9284	0.0303	-5.5394
H	2.0776	2.0056	-4.2764
H	0.5724	3.1646	-2.5251
H	-1.1343	-0.1136	-4.7844
H	-2.4800	0.0859	-3.6409
H	-0.9984	1.0506	-3.4476
H	0.0946	0.1870	-0.2046
H	-2.3527	-1.8047	3.9763
H	-4.1157	-1.0627	5.5389
H	-5.1914	1.1524	5.2899
H	-4.4960	2.6735	3.4389
H	-4.0259	1.3129	0.1483
H	-4.2917	2.9291	0.8302
H	-3.1215	2.7035	-0.4826
H	-0.6670	-1.6642	0.6434
H	-1.0051	-2.1045	2.3192
H	0.3863	-1.0578	1.9511
H	2.1123	-3.8280	-2.7068
H	1.0343	-4.8073	-1.6984
H	1.5231	-3.1938	-1.1560
H	-1.3526	3.8576	1.0832
H	-0.9874	3.1162	2.6540
H	-2.5292	3.9605	2.4012
C	2.9727	1.5973	-1.6380
H	3.7374	0.9356	-2.0525
H	3.2315	2.6250	-1.9064
C	2.9210	1.3940	-0.1735
C	3.3491	0.1125	0.5090
C	2.7925	-1.1597	-0.1378
H	3.1112	-1.2422	-1.1810
H	1.7015	-1.1890	-0.1000
H	3.1754	-2.0280	0.4059
C	4.9009	0.0834	0.4744
H	5.2608	-0.0293	-0.5521
H	5.2386	-0.7763	1.0594
H	5.3262	0.9930	0.9081
N	2.4733	2.2488	0.7053
C	2.1317	3.6559	0.5222
H	2.7830	4.2437	1.1727
H	1.0894	3.8036	0.8059
H	2.2710	3.9467	-0.5148
C	2.8664	0.3534	1.9148
C	2.3771	1.6487	1.9967
C	2.8568	-0.4553	3.0395
H	3.2371	-1.4714	3.0005
C	2.3262	0.0666	4.2218
H	2.2935	-0.5537	5.1117
C	1.8347	1.3728	4.2779
H	1.4269	1.7542	5.2081
C	1.8582	2.2007	3.1549
H	1.4810	3.2167	3.1958
H	1.2848	0.2425	-1.8902
H	-1.0330	3.0159	-0.8246

Cy7+FB C4' adduct deprot. (0)

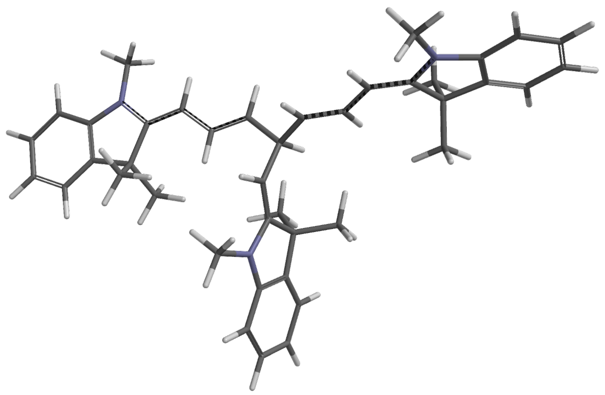

E_{elec}(au)	H°(au)	G°(au)	NImag
-1754.37278	-1753.58666	-1753.68431	0

C	-7.7015	1.1907	5.7831
C	-7.0159	1.7962	6.8334
C	-5.6268	1.9560	6.8029
C	-4.9439	1.4885	5.6840
C	-5.6229	0.8810	4.6231
C	-6.9958	0.7285	4.6648
N	-3.5812	1.5189	5.4044
C	-3.3047	0.9543	4.1615
C	-4.6309	0.4779	3.5469
C	-4.9516	1.2130	2.2321
C	-2.0529	0.8876	3.6528
C	-1.6684	0.3283	2.3675
C	-0.4219	0.3523	1.8730
C	-0.0427	-0.1879	0.5122
C	1.0313	-1.2568	0.6261
C	-2.5609	2.0769	6.2567
C	2.1046	-1.3244	-0.1748
C	0.3885	0.9548	-0.3761
C	-0.0856	1.2738	-1.5907
N	0.4163	2.3453	-2.3430
C	-0.2085	2.4286	-3.5790
C	-1.1839	1.4297	-3.6809
C	-1.2010	0.6009	-2.4079
C	0.0143	3.3241	-4.6217
C	-0.7675	3.1954	-5.7739
C	-1.7421	2.2070	-5.8841
C	-1.9517	1.3146	-4.8239
C	-2.5721	0.7243	-1.7179
C	1.5505	3.1244	-1.9184
C	-4.6609	-1.0484	3.3475
C	-0.8709	-0.8673	-2.7293
H	-8.7803	1.0780	5.8300
H	-7.5659	2.1547	7.6989
H	-5.1137	2.4321	7.6320
H	-7.5215	0.2552	3.8389

H	-4.9260	2.2974	2.3790
H	-4.2404	0.9558	1.4420
H	-5.9546	0.9353	1.8902
H	-1.2378	1.3038	4.2416
H	-2.4398	-0.1336	1.7547
H	0.3834	0.8125	2.4496
H	-1.8071	1.3199	6.5026
H	-2.0577	2.9169	5.7631
H	-3.0085	2.4353	7.1833
H	1.1990	1.5552	0.0336
H	0.7661	4.1040	-4.5568
H	-0.6063	3.8864	-6.5968
H	-2.3374	2.1266	-6.7885
H	-2.7086	0.5374	-4.9009
H	-2.7950	1.7709	-1.4866
H	-3.3566	0.3433	-2.3813
H	-2.6080	0.1520	-0.7861
H	1.7982	3.8653	-2.6789
H	2.4248	2.4798	-1.7567
H	1.3340	3.6487	-0.9800
H	-5.6605	-1.3570	3.0218
H	-4.4286	-1.5657	4.2837
H	-3.9431	-1.3705	2.5885
H	0.0965	-0.9417	-3.2361
H	-1.6384	-1.2840	-3.3909
H	-0.8302	-1.4808	-1.8249
C	3.1451	-2.3347	-0.1241
C	4.2012	-2.4024	-0.9679
C	4.5483	-1.4856	-2.1548
C	3.4573	-1.4919	-3.2413
H	2.5434	-0.9976	-2.9011
H	3.2074	-2.5171	-3.5320
H	3.8172	-0.9595	-4.1284
C	4.8636	-0.0478	-1.6993
H	3.9772	0.4542	-1.3010
H	5.2308	0.5366	-2.5498
H	5.6366	-0.0493	-0.9239
N	5.1855	-3.3827	-0.8755
C	5.1833	-4.4022	0.1438
H	4.3155	-5.0641	0.0357
H	5.1514	-3.9455	1.1395
H	6.0904	-5.0016	0.0703
C	5.8089	-2.1455	-2.6824
C	6.1290	-3.2527	-1.8905
C	6.6021	-1.7999	-3.7595
H	6.3513	-0.9375	-4.3718
C	7.7320	-2.5740	-4.0528
H	8.3647	-2.3148	-4.8964
C	8.0400	-3.6777	-3.2611
H	8.9147	-4.2789	-3.4925
C	7.2450	-4.0372	-2.1677
H	7.5017	-4.9049	-1.5680
H	3.0521	-3.0874	0.6565
H	-0.9407	-0.6646	0.1017

H	0.8774	-2.0061	1.4047
H	2.1921	-0.5492	-0.9294

Cy5+FB-CH=CH₂ C2' adduct (+)

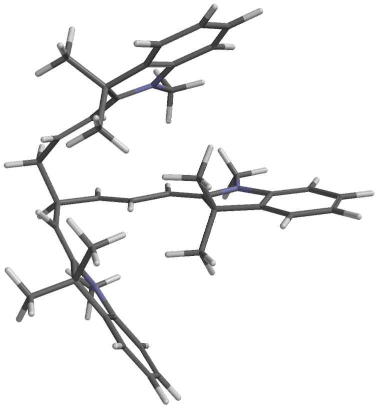

E_{elec}(au)	H°(au)	G°(au)	NImag
-1754.85097	-1754.04824	-1754.14388	0

C	-2.2568	-5.4436	-0.7193
C	-1.5645	-5.9409	-1.8209
C	-0.7185	-5.1265	-2.5806
C	-0.5870	-3.7953	-2.1989
C	-1.2789	-3.2856	-1.0964
C	-2.1118	-4.0998	-0.3526
N	0.1834	-2.7840	-2.7688
C	0.0272	-1.5834	-2.0883
C	-0.9318	-1.8211	-0.9103
C	-0.2300	-1.6259	0.4463
C	0.6505	-0.4387	-2.4609
C	0.5601	0.8305	-1.7636
C	1.1798	1.9600	-2.1339
C	1.1545	3.2556	-1.3551
C	2.5150	3.4191	-0.5831
C	1.0467	-2.9273	-3.9154
C	2.6220	2.4382	0.5359
C	3.0235	1.1647	0.3505
C	2.9698	0.1366	1.3500
N	3.1633	-1.1270	1.0291
C	3.0913	-1.9709	2.1724
C	2.8391	-1.1744	3.2803
C	2.7271	0.2674	2.8472
C	3.2267	-3.3485	2.2464
C	3.0980	-3.9215	3.5114
C	2.8448	-3.1376	4.6389
C	2.7112	-1.7523	4.5326
C	1.3188	0.8032	3.1811
C	3.4122	-1.6252	-0.3232
C	-2.1952	-0.9486	-1.0066
C	3.8246	1.1341	3.4972
H	-2.9081	-6.0958	-0.1455
H	-1.6802	-6.9837	-2.1028
H	-0.1903	-5.5347	-3.4361
H	-2.6464	-3.7017	0.5064

H	0.6291	-2.2984	0.5365
H	0.1181	-0.5969	0.5706
H	-0.9260	-1.8519	1.2613
H	1.2894	-0.4584	-3.3416
H	-0.0515	0.8762	-0.8688
H	1.8119	1.9618	-3.0232
H	0.7078	-2.2912	-4.7416
H	2.0747	-2.6448	-3.6583
H	1.0467	-3.9639	-4.2512
H	3.3514	0.8687	-0.6397
H	3.4243	-3.9662	1.3781
H	3.1965	-4.9970	3.6155
H	2.7485	-3.6124	5.6100
H	2.5101	-1.1438	5.4093
H	0.5426	0.2260	2.6724
H	1.1647	0.7139	4.2601
H	1.2087	1.8546	2.9125
H	3.3350	-2.7100	-0.3209
H	4.4132	-1.3304	-0.6446
H	2.6572	-1.2233	-1.0021
H	-1.9617	0.1113	-0.8854
H	-2.9029	-1.2315	-0.2196
H	-2.6868	-1.0792	-1.9760
H	3.7630	2.1694	3.1522
H	4.8201	0.7437	3.2665
H	3.6863	1.1207	4.5820
C	-0.0027	3.3943	-0.3861
C	-1.3123	3.1439	-0.5612
C	-2.3151	3.0933	0.6087
C	-1.8325	2.1559	1.7235
H	-0.9308	2.5544	2.1987
H	-1.6024	1.1609	1.3287
H	-2.6056	2.0537	2.4931
C	-2.5730	4.5009	1.1726
H	-1.6543	4.9109	1.6067
H	-3.3384	4.4596	1.9555
H	-2.9183	5.1800	0.3864
N	-2.0009	2.8191	-1.7329
C	-1.5955	3.0960	-3.0945
H	-2.4174	3.5953	-3.6201
H	-1.3311	2.1821	-3.6361
H	-0.7379	3.7660	-3.0968
C	-3.5529	2.5477	-0.0722
C	-3.3045	2.4263	-1.4418
C	-4.7848	2.1970	0.4460
H	-4.9825	2.2930	1.5111
C	-5.7765	1.7136	-0.4195
H	-6.7466	1.4268	-0.0253
C	-5.5174	1.5995	-1.7831
H	-6.2895	1.2227	-2.4482
C	-4.2749	1.9556	-2.3195
H	-4.0778	1.8502	-3.3817
H	1.1638	4.0816	-2.0800
H	2.2820	2.7708	1.5107

H	0.2641	3.6737	0.6302
H	3.3302	3.2741	-1.2991
H	2.5753	4.4388	-0.1910

Cy5+FBCH=CH$_2$ C2’ cleavage TS (+)

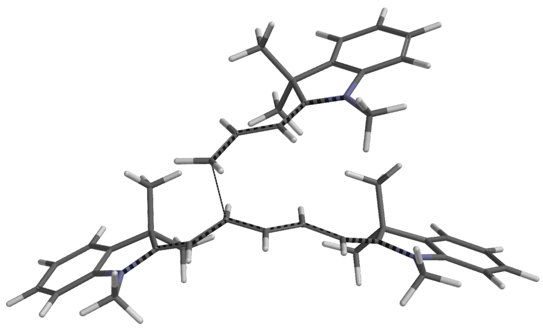

E_{elec}(au)	H°(au)	G°(au)	NImag
-1754.82036	-1754.02130	-1754.11876	1

C	-1.3745	1.8177	1.9889
N	-1.7406	1.1718	3.1209
C	-1.0784	1.6912	4.2498
C	-0.2277	2.7134	3.8387
C	-0.3396	2.8920	2.3393
C	-1.1966	1.3219	5.5824
C	-0.4195	2.0190	6.5109
C	0.4391	3.0431	6.1139
C	0.5393	3.3972	4.7651
C	-0.8602	4.3079	2.0216
C	-2.7118	0.0942	3.1561
C	1.0208	2.6251	1.6676
H	-1.8649	0.5327	5.9086
H	-0.4930	1.7560	7.5616
H	1.0300	3.5707	6.8559
H	1.2033	4.1982	4.4514
H	-1.8327	4.4780	2.4934
H	-0.1528	5.0460	2.4123
H	-0.9630	4.4719	0.9463
H	-2.4020	-0.7084	2.4807
H	-3.6978	0.4620	2.8555
H	-2.7752	-0.3036	4.1672
H	0.9662	2.7336	0.5816
H	1.3770	1.6169	1.8989
H	1.7537	3.3446	2.0461
C	-1.9241	1.5095	0.7558
H	-2.6895	0.7410	0.7171
C	-1.5886	2.1307	-0.4583
H	-0.8267	2.9044	-0.4645
C	-2.0794	1.7347	-1.6885
H	-2.9673	1.1106	-1.7417
H	-1.8816	2.3566	-2.5536
C	1.8332	-5.3212	4.9062
C	0.7290	-6.1630	4.7909
C	-0.2949	-5.8993	3.8765
C	-0.1735	-4.7639	3.0847
C	0.9276	-3.9133	3.1901

C	1.9349	-4.1833	4.0978
N	-1.0384	-4.2743	2.0995
C	-0.5380	-3.1358	1.5126
C	0.7884	-2.7774	2.1952
C	1.9669	-2.8030	1.2040
C	-1.1671	-2.5140	0.4734
C	-0.7079	-1.3528	-0.2301
C	-1.2385	-0.9516	-1.4176
C	-2.2738	-4.9033	1.6890
C	-0.7144	0.1434	-2.1826
C	-0.8569	0.1199	-3.6108
C	-0.0278	0.7233	-4.5124
N	-0.2408	0.6441	-5.8648
C	0.7640	1.3067	-6.5814
C	1.6750	1.8610	-5.6826
C	1.2589	1.5230	-4.2640
C	0.9160	1.4461	-7.9551
C	2.0212	2.1697	-8.4117
C	2.9379	2.7302	-7.5247
C	2.7653	2.5749	-6.1447
C	2.3456	0.6548	-3.6000
C	-1.3661	-0.0474	-6.4554
C	0.7023	-1.4276	2.9319
C	1.0004	2.8056	-3.4543
H	2.6169	-5.5500	5.6215
H	0.6573	-7.0463	5.4187
H	-1.1440	-6.5698	3.7978
H	2.7961	-3.5250	4.1785
H	2.0018	-3.7579	0.6703
H	1.8957	-1.9983	0.4676
H	2.9069	-2.6805	1.7518
H	-2.0745	-2.9680	0.0836
H	-3.1029	-4.1918	1.7608
H	-2.2035	-5.2607	0.6555
H	-2.4883	-5.7509	2.3384
H	-1.7075	-0.4410	-3.9876
H	0.2142	1.0154	-8.6611
H	2.1626	2.2922	-9.4813
H	3.7887	3.2867	-7.9047
H	3.4788	3.0060	-5.4473
H	2.5122	-0.2625	-4.1727
H	3.2850	1.2159	-3.5654
H	2.0777	0.3802	-2.5763
H	-1.3375	0.0640	-7.5380
H	-2.3072	0.3724	-6.0852
H	-1.3338	-1.1144	-6.2104
H	1.6393	-1.2423	3.4673
H	-0.1152	-1.4338	3.6595
H	0.5369	-0.6001	2.2366
H	0.7178	2.5820	-2.4220
H	0.2056	3.4027	-3.9118
H	1.9140	3.4084	-3.4286
H	-2.0700	-1.5086	-1.8478
H	0.1364	-0.7899	0.1589

H	0.1699	0.6139	-1.7671

Section 5: FB+Cy5 reaction at C2’

Cy5+FB C2' attack TS (+)

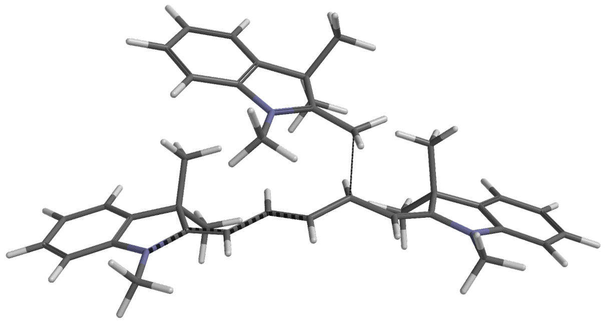

E_{elec}(au)	H°(au)	G°(au)	NImag
-1677.44694	-1676.68222	-1676.77648	1

C	-1.3821	5.9762	5.6064
C	-0.4790	6.9231	5.1271
C	0.3329	6.6606	4.0195
C	0.2080	5.4168	3.4136
C	-0.6944	4.4633	3.8812
C	-1.4925	4.7307	4.9785
N	0.8995	4.8916	2.3116
C	0.4708	3.6256	2.0043
C	-0.5868	3.2097	3.0380
C	-1.9412	2.8798	2.3875
C	0.9388	2.9433	0.9139
C	0.5675	1.6205	0.4971
C	0.6828	1.2704	-0.8889
C	1.9200	5.5985	1.5670
C	0.0027	0.2306	-1.4504
C	0.0943	-0.1307	-2.8270
C	-0.6510	-1.0901	-3.4585
N	-0.5448	-1.3831	-4.7959
C	-1.4200	-2.4110	-5.1663
C	-2.1657	-2.8074	-4.0589
C	-1.7458	-1.9895	-2.8549
C	-1.5803	-2.9997	-6.4140
C	-2.5341	-4.0167	-6.5247
C	-3.2897	-4.4223	-5.4260
C	-3.1057	-3.8146	-4.1780
C	-2.9357	-1.1579	-2.3402
C	0.3435	-0.7717	-5.7632
C	-0.0857	2.0521	3.9166
C	-1.1986	-2.9252	-1.7636
H	-2.0013	6.2040	6.4685
H	-0.3997	7.8876	5.6200
H	1.0280	7.4119	3.6603
H	-2.1928	3.9854	5.3475
H	-2.6926	2.7292	3.1694
H	-1.8958	1.9671	1.7879
H	-2.2720	3.7006	1.7440

H	1.6589	3.4452	0.2731
H	1.3551	1.8675	-1.5042
H	2.8246	4.9864	1.4946
H	1.5707	5.8389	0.5567
H	2.1709	6.5247	2.0826
H	-0.6573	-0.3374	-0.8014
H	0.8102	0.4402	-3.4120
H	-0.9920	-2.6928	-7.2727
H	-2.6835	-4.4975	-7.4871
H	-4.0232	-5.2150	-5.5372
H	-3.6913	-4.1308	-3.3185
H	-3.3145	-0.4956	-3.1248
H	-3.7441	-1.8310	-2.0368
H	-2.6597	-0.5473	-1.4764
H	1.1262	-1.4758	-6.0674
H	-0.2302	-0.4755	-6.6474
H	0.8104	0.1192	-5.3470
H	0.8772	2.2959	4.3759
H	0.0338	1.1403	3.3328
H	-0.8111	1.8525	4.7116
H	-1.9945	-3.6001	-1.4323
H	-0.8365	-2.3756	-0.8922
H	-0.3722	-3.5285	-2.1476
H	-0.2143	1.1093	1.0474
C	2.1446	0.4410	1.4182
H	2.9223	0.9861	0.8962
H	1.9674	0.7552	2.4393
H	2.6877	-3.6203	-1.8841
C	2.1487	-3.8386	-0.9681
C	0.7333	-4.4101	1.4152
C	1.9024	-2.8666	-0.0103
C	1.6617	-5.1236	-0.7091
C	0.9582	-5.4084	0.4606
C	1.2208	-3.1389	1.1719
H	1.8321	-5.9108	-1.4373
H	0.5864	-6.4132	0.6361
H	0.1920	-4.6311	2.3314
N	2.2738	-1.5071	-0.0306
C	1.9107	-0.8858	1.1171
C	1.1391	-1.8757	1.9968
C	3.1098	-0.9842	-1.0957
H	3.3180	0.0701	-0.9282
H	2.5878	-1.0903	-2.0518
H	4.0515	-1.5416	-1.1323
C	1.8430	-2.0301	3.3560
H	1.3384	-2.8011	3.9470
H	2.8901	-2.3202	3.2260
H	1.8069	-1.0892	3.9148
C	-0.3318	-1.4767	2.2037
H	-0.8498	-1.3266	1.2523
H	-0.4151	-0.5638	2.7959
H	-0.8453	-2.2744	2.7496

Cy5+FB C2' adduct (+)

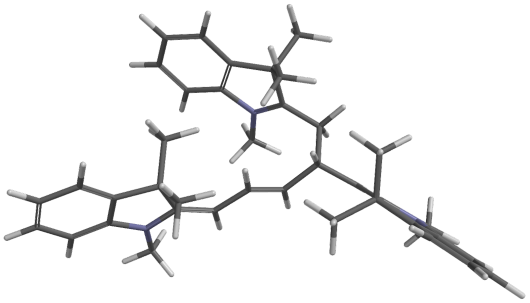

E_{elec}(au)	H°(au)	G°(au)	NImag
-1677.47748	-1676.70891	-1676.80149	0

C	-7.2011	1.1344	-0.8936
C	-7.1218	2.3395	-1.5874
C	-5.8890	2.9127	-1.9151
C	-4.7374	2.2383	-1.5236
C	-4.8050	1.0284	-0.8280
C	-6.0292	0.4711	-0.5094
N	-3.4014	2.5856	-1.7162
C	-2.5478	1.6391	-1.1698
C	-3.4022	0.5336	-0.5275
C	-3.1686	-0.8352	-1.1922
C	-1.1978	1.7453	-1.2358
C	-0.2494	0.8222	-0.6468
C	1.0833	0.9819	-0.6413
C	-2.9298	3.7756	-2.3812
C	2.0084	0.0551	0.1020
C	2.4157	0.6533	1.5021
C	1.2251	0.9842	2.3204
N	0.5557	2.1017	2.2493
C	-0.6179	2.0530	3.0601
C	-0.6553	0.8219	3.6969
C	0.5859	0.0506	3.3273
C	-1.5949	3.0224	3.2017
C	-2.6670	2.7012	4.0351
C	-2.7326	1.4646	4.6814
C	-1.7232	0.5121	4.5233
C	1.5369	-0.0528	4.5467
C	0.8627	3.2982	1.4734
C	-3.1967	0.4520	0.9959
C	0.2996	-1.3489	2.7654
H	-8.1699	0.7093	-0.6505
H	-8.0332	2.8512	-1.8833
H	-5.8501	3.8516	-2.4575
H	-6.0811	-0.4693	0.0341
H	-3.3111	-0.7690	-2.2753
H	-2.1604	-1.2127	-1.0000
H	-3.8842	-1.5627	-0.7941
H	-0.7674	2.6064	-1.7426
H	-0.6442	-0.0530	-0.1357

H	1.5380	1.8430	-1.1323
H	-3.7773	4.3803	-2.7021
H	-2.3104	4.3744	-1.7025
H	-2.3303	3.5170	-3.2621
H	-1.5456	3.9769	2.6895
H	-3.4639	3.4240	4.1756
H	-3.5831	1.2402	5.3171
H	-1.7776	-0.4460	5.0311
H	1.7704	0.9351	4.9536
H	1.0392	-0.6426	5.3214
H	2.4669	-0.5568	4.2691
H	0.8668	4.1510	2.1549
H	0.0878	3.4273	0.7143
H	1.8340	3.2013	0.9973
H	-3.9009	-0.2698	1.4236
H	-3.3723	1.4242	1.4653
H	-2.1847	0.1302	1.2532
H	1.2337	-1.8593	2.5142
H	-0.3392	-1.3110	1.8790
H	-0.2103	-1.9355	3.5348
H	1.4671	-0.8705	0.3056
C	3.2670	-0.2309	-0.6724
C	3.5762	-1.3701	-1.3164
C	2.7755	-2.6800	-1.4223
C	2.6566	-3.3901	-0.0617
H	2.0312	-2.8243	0.6341
H	3.6427	-3.5271	0.3930
H	2.1991	-4.3760	-0.1983
C	1.3887	-2.4665	-2.0534
H	0.7341	-1.8753	-1.4072
H	0.9095	-3.4367	-2.2241
H	1.4757	-1.9510	-3.0150
N	4.7572	-1.5258	-2.0463
C	5.7349	-0.4760	-2.1813
H	6.0885	-0.1545	-1.1946
H	5.3095	0.3949	-2.6963
H	6.5901	-0.8366	-2.7523
C	3.6554	-3.4956	-2.3533
C	4.7991	-2.7611	-2.6835
C	3.4675	-4.7666	-2.8612
H	2.5781	-5.3365	-2.6022
C	4.4337	-5.3115	-3.7170
H	4.2976	-6.3077	-4.1265
C	5.5671	-4.5707	-4.0425
H	6.3138	-4.9938	-4.7087
C	5.7700	-3.2845	-3.5320
H	6.6595	-2.7243	-3.8014
H	3.0127	-0.0946	2.0305
H	3.0433	1.5355	1.3467
H	3.9539	0.6104	-0.7433

Cy5+FB C2' adduct deprot. (0)

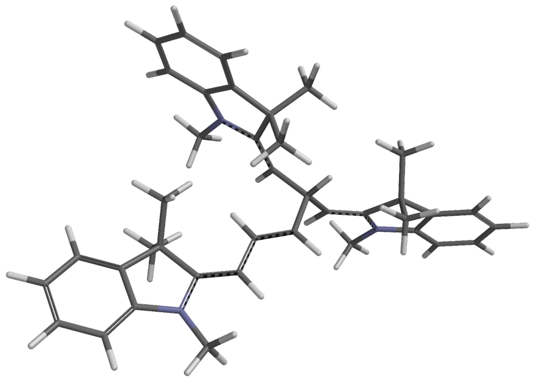

E_{elec}(au)	H°(au)	G°(au)	NImag
-1676.99287	-1676.24027	-1676.33384	0

C	-1.6769	-6.4915	4.0651
C	-0.5808	-6.4540	4.9233
C	0.4240	-5.4920	4.7810
C	0.2965	-4.5668	3.7491
C	-0.8005	-4.5950	2.8822
C	-1.7868	-5.5511	3.0327
N	1.1418	-3.5215	3.3920
C	0.6396	-2.8089	2.3067
C	-0.6698	-3.4844	1.8544
C	-1.8962	-2.5542	1.9221
C	1.2636	-1.7253	1.7876
C	0.7478	-0.9389	0.6844
C	1.2602	0.1982	0.1955
C	2.3737	-3.1721	4.0544
C	0.6167	1.0087	-0.9265
C	-0.6464	0.3506	-1.4323
C	-1.8930	0.4740	-0.9487
N	-2.9624	-0.3108	-1.4193
C	-4.0716	-0.1729	-0.5932
C	-3.8385	0.8384	0.3452
C	-2.4529	1.4216	0.1288
C	-5.2742	-0.8741	-0.6110
C	-6.2475	-0.5280	0.3315
C	-6.0296	0.4837	1.2629
C	-4.8109	1.1754	1.2677
C	-2.5721	2.8491	-0.4395
C	-2.7626	-1.4114	-2.3282
C	-0.5300	-4.0934	0.4455
C	-1.6613	1.4428	1.4437
H	-2.4454	-7.2472	4.1951
H	-0.4978	-7.1842	5.7234
H	1.2685	-5.4811	5.4621
H	-2.6391	-5.5728	2.3574
H	-1.9578	-2.0579	2.8957
H	-1.8723	-1.7865	1.1437
H	-2.8093	-3.1416	1.7774

H	2.1931	-1.3888	2.2423
H	-0.1738	-1.2804	0.2286
H	2.1880	0.6087	0.5975
H	3.2109	-3.1943	3.3465
H	2.3116	-2.1653	4.4865
H	2.5793	-3.8831	4.8545
H	-0.4777	-0.4030	-2.1988
H	-5.4640	-1.6696	-1.3242
H	-7.1906	-1.0676	0.3336
H	-6.7993	0.7339	1.9864
H	-4.6306	1.9638	1.9947
H	-3.1428	2.8523	-1.3737
H	-3.0825	3.4975	0.2813
H	-1.5818	3.2701	-0.6372
H	-3.7243	-1.8552	-2.5863
H	-2.1182	-2.1851	-1.8859
H	-2.2952	-1.0581	-3.2530
H	-1.4316	-4.6658	0.2001
H	0.3306	-4.7687	0.3988
H	-0.4019	-3.3173	-0.3153
H	-0.6738	1.8924	1.3155
H	-1.5268	0.4348	1.8444
H	-2.2087	2.0386	2.1825
H	0.3804	1.9986	-0.5240
C	1.6115	1.1563	-2.0597
C	2.3676	2.2225	-2.3663
C	2.4142	3.6120	-1.7086
C	1.1083	4.3873	-1.9599
H	0.2609	3.9042	-1.4666
H	0.8932	4.4464	-3.0316
H	1.1969	5.4065	-1.5677
C	2.7352	3.5510	-0.2064
H	1.9152	3.1067	0.3644
H	2.9003	4.5634	0.1785
H	3.6400	2.9623	-0.0256
N	3.2916	2.2183	-3.4234
C	3.5260	1.0604	-4.2465
H	3.9014	0.2190	-3.6494
H	4.2575	1.2954	-5.0191
H	2.5971	0.7450	-4.7354
C	3.5515	4.2698	-2.4698
C	4.0166	3.4007	-3.4638
C	4.1111	5.5233	-2.3149
H	3.7443	6.1971	-1.5439
C	5.1573	5.9166	-3.1602
H	5.6089	6.8974	-3.0472
C	5.6154	5.0444	-4.1440
H	6.4278	5.3497	-4.7980
C	5.0551	3.7739	-4.3132
H	5.4312	3.1102	-5.0852
H	1.7464	0.2470	-2.6435

Cy3+FB-CH=CH_2 C2' adduct (+)

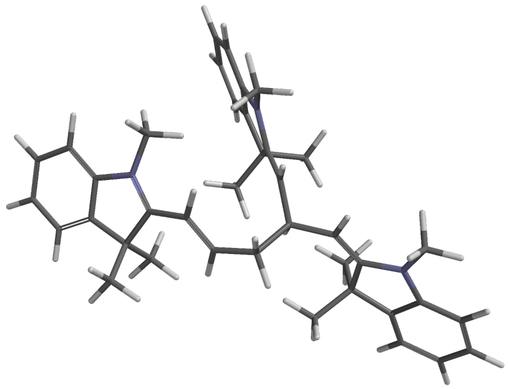

E_{elec}(au)	H°(au)	G°(au)	NImag
-1677.46662	-1676.69908	-1676.79197	0

C	-1.1993	1.3914	-7.2453
C	-1.6246	0.0620	-7.1896
C	-1.3020	-0.7531	-6.1044
C	-0.5431	-0.1720	-5.1019
C	-0.1053	1.1433	-5.1377
C	-0.4372	1.9477	-6.2157
N	-0.0707	-0.7667	-3.9009
C	0.6526	0.0793	-3.1967
C	0.6657	1.4448	-3.8737
C	-0.1093	2.5046	-3.0549
C	1.3467	-0.3353	-2.0089
C	1.7660	0.4930	-1.0328
C	2.3052	0.0006	0.2635
C	-0.4077	-2.1433	-3.5512
C	1.1213	-0.3725	1.2397
C	1.6500	-0.5329	2.6432
C	1.6655	0.3911	3.6202
N	2.1892	0.1250	4.8926
C	2.2078	1.2673	5.6815
C	1.6226	2.3280	4.9824
C	1.1778	1.8538	3.6096
C	2.6996	1.4417	6.9723
C	2.5794	2.7089	7.5520
C	1.9903	3.7690	6.8676
C	1.5059	3.5745	5.5674
C	-0.3537	1.9599	3.4964
C	2.7481	-1.1522	5.2546
C	2.1053	1.9028	-4.1632
C	1.8442	2.6927	2.5072
H	-1.4651	2.0009	-8.1031
H	-2.2151	-0.3478	-8.0024
H	-1.6279	-1.7865	-6.0621
H	-0.1113	2.9825	-6.2611
H	-1.1149	2.1541	-2.8073
H	0.4106	2.7731	-2.1339
H	-0.1981	3.4064	-3.6672

H	1.4898	-1.4022	-1.8747
H	1.6153	1.5651	-1.1147
H	0.2163	-2.8312	-4.1258
H	-0.2677	-2.2997	-2.4840
H	-1.4596	-2.3073	-3.7892
H	3.1673	0.6319	7.5228
H	2.9587	2.8623	8.5585
H	1.9079	4.7438	7.3383
H	1.0448	4.3960	5.0240
H	-0.8488	1.3138	4.2280
H	-0.6647	2.9937	3.6822
H	-0.7000	1.6852	2.4960
H	3.0101	-1.1556	6.3127
H	3.6482	-1.3768	4.6674
H	2.0149	-1.9483	5.0809
H	2.0731	2.8340	-4.7359
H	2.6463	1.1519	-4.7462
H	2.6488	2.0855	-3.2323
H	1.4883	2.4006	1.5147
H	2.9332	2.5864	2.5345
H	1.5973	3.7504	2.6497
H	0.3980	0.4427	1.2099
C	0.5033	-1.6596	0.7575
C	-0.7386	-1.9002	0.3051
C	-1.9833	-0.9967	0.2839
C	-2.4483	-0.7490	1.7314
H	-1.6759	-0.2263	2.3016
H	-2.6580	-1.6983	2.2348
H	-3.3612	-0.1432	1.7382
C	-1.8070	0.3270	-0.4724
H	-1.0662	0.9833	-0.0089
H	-2.7610	0.8646	-0.4911
H	-1.5064	0.1394	-1.5066
N	-1.1179	-3.1552	-0.1959
C	-0.2027	-4.2632	-0.3030
H	0.6401	-4.0182	-0.9638
H	-0.7205	-5.1321	-0.7081
H	0.1980	-4.5287	0.6821
C	-2.9858	-1.8757	-0.4419
C	-2.4179	-3.1303	-0.6864
C	-4.2786	-1.6006	-0.8448
H	-4.7190	-0.6250	-0.6539
C	-5.0143	-2.5923	-1.5069
H	-6.0301	-2.3894	-1.8315
C	-4.4382	-3.8367	-1.7489
H	-5.0093	-4.6028	-2.2659
C	-3.1313	-4.1278	-1.3445
H	-2.7010	-5.1029	-1.5480
H	1.2120	-2.4867	0.7324
H	2.0911	-1.5058	2.8495
H	2.9192	-0.8955	0.1218
H	2.9230	0.7698	0.7311

Cy3+FB=CHCH=CH_2 attack/cleave TS (+)

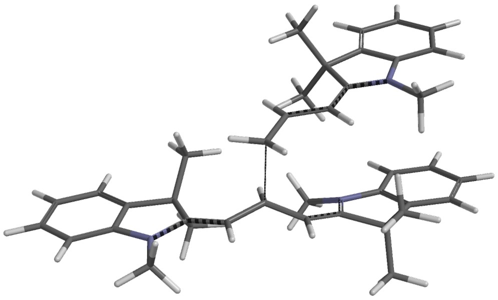

E_{elec}(au)	H°(au)	G°(au)	NImag
-1677.44102	-1676.67557	-1676.76915	1

C	2.6574	1.1731	-0.2634
N	3.5792	1.1523	-1.2541
C	3.8686	2.4486	-1.7202
C	3.1423	3.3671	-0.9685
C	2.3011	2.6309	0.0521
C	4.7131	2.8407	-2.7498
C	4.8199	4.2106	-3.0018
C	4.1058	5.1439	-2.2507
C	3.2561	4.7225	-1.2235
C	2.7297	3.0263	1.4792
C	4.1687	-0.0581	-1.7912
C	0.8116	2.9347	-0.1899
H	5.2709	2.1277	-3.3470
H	5.4728	4.5493	-3.8004
H	4.2075	6.2028	-2.4667
H	2.6937	5.4454	-0.6386
H	3.7876	2.8015	1.6446
H	2.5792	4.1023	1.6118
H	2.1408	2.5048	2.2378
H	4.5936	-0.6579	-0.9818
H	4.9646	0.2028	-2.4862
H	3.4109	-0.6423	-2.3207
H	0.1672	2.4118	0.5214
H	0.5221	2.6466	-1.2039
H	0.6407	4.0096	-0.0760
C	2.1824	0.0134	0.3314
H	2.5689	-0.9380	-0.0210
C	1.2479	-0.0261	1.3759
C	0.7104	-1.1852	1.9190
H	0.8566	0.9108	1.7611
C	2.3118	-0.0717	-5.2359
C	1.6473	1.0828	-4.8269
C	0.7428	1.0605	-3.7605
C	0.5428	-0.1531	-3.1163
C	1.2130	-1.3155	-3.5056
C	2.0860	-1.2876	-4.5782
N	-0.2857	-0.4334	-2.0230
C	-0.0430	-1.7073	-1.5586

C	0.7496	-2.4623	-2.6301
C	1.8936	-3.3114	-2.0723
C	-0.4601	-2.2600	-0.3854
C	-1.4002	0.4108	-1.6464
C	-1.0211	-1.5707	0.7448
C	-1.8201	-2.2933	1.6994
C	-2.7484	-1.7333	2.5288
N	-3.4641	-2.4723	3.4400
C	-4.2807	-1.6566	4.2325
C	-4.1720	-0.3370	3.7943
C	-3.2446	-0.2784	2.5957
C	-5.0894	-2.0045	5.3074
C	-5.8018	-0.9794	5.9376
C	-5.7060	0.3420	5.5064
C	-4.8845	0.6688	4.4211
C	-4.0757	0.0409	1.3344
C	-3.3310	-3.9042	3.5958
C	-0.2296	-3.3538	-3.4282
C	-2.1450	0.7751	2.8030
H	3.0083	-0.0289	-6.0674
H	1.8318	2.0216	-5.3408
H	0.2284	1.9651	-3.4526
H	2.5992	-2.1909	-4.8972
H	2.5779	-2.7156	-1.4624
H	1.5070	-4.1303	-1.4576
H	2.4611	-3.7531	-2.8980
H	-0.2562	-3.3196	-0.2522
H	-1.6578	1.0514	-2.4928
H	-2.2634	-0.2232	-1.4235
H	-1.1834	1.0460	-0.7824
H	-1.6230	-3.3587	1.7803
H	-5.1690	-3.0265	5.6623
H	-6.4376	-1.2248	6.7833
H	-6.2669	1.1208	6.0137
H	-4.8079	1.6977	4.0787
H	-3.4501	0.0415	0.4382
H	-4.8735	-0.6962	1.1993
H	-4.5313	1.0312	1.4370
H	-2.3739	-4.1663	4.0616
H	-3.3964	-4.3908	2.6178
H	-4.1450	-4.2790	4.2165
H	0.2959	-3.8219	-4.2672
H	-1.0609	-2.7623	-3.8251
H	-0.6355	-4.1410	-2.7842
H	-1.5393	0.9212	1.9052
H	-1.4853	0.5063	3.6332
H	-2.6124	1.7376	3.0335
H	1.1935	-2.1391	1.7246
H	0.1922	-1.1049	2.8668
H	-1.2819	-0.5343	0.5990

Section 6: FB+Cy3 reaction at C2'

Cy3+FB C2 attack TS (+)

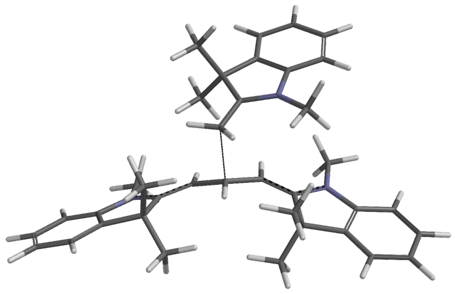

E_{elec}(au)	H°(au)	G°(au)	NImag
-1600.06623	-1599.33430	-1599.42464	1

C	0.6997	-3.9620	-6.3301
C	-0.5608	-3.5090	-6.7144
C	-1.2808	-2.6047	-5.9280
C	-0.6927	-2.1725	-4.7455
C	0.5675	-2.6181	-4.3504
C	1.2720	-3.5122	-5.1353
N	-1.1877	-1.2879	-3.7790
C	-0.3023	-1.1257	-2.7414
C	0.9489	-1.9703	-3.0343
C	2.1967	-1.0884	-3.2324
C	-0.5734	-0.2939	-1.6925
C	0.2707	-0.0463	-0.5568
C	0.0723	1.1598	0.2023
C	-2.4692	-0.6193	-3.8449
C	1.0438	1.9030	0.8050
N	0.7457	2.9631	1.6259
C	1.9035	3.6379	2.0348
C	3.0188	2.9998	1.4936
C	2.5697	1.8426	0.6237
C	2.0247	4.7554	2.8508
C	3.3157	5.2169	3.1243
C	4.4392	4.5839	2.5966
C	4.2918	3.4636	1.7709
C	2.9167	2.1415	-0.8484
C	-0.6033	3.3435	1.9819
C	1.2170	-3.0427	-1.9654
C	3.2295	0.5350	1.0899
H	1.2387	-4.6640	-6.9584
H	-0.9994	-3.8619	-7.6432
H	-2.2611	-2.2645	-6.2446
H	2.2557	-3.8596	-4.8296
H	3.0099	-1.6988	-3.6380
H	2.5424	-0.6661	-2.2854
H	1.9956	-0.2702	-3.9304
H	-1.5139	0.2464	-1.7045
H	-0.9596	1.4834	0.3105
H	-2.3345	0.4675	-3.8601

H	-2.9915	-0.9178	-4.7525
H	-3.0864	-0.8880	-2.9812
H	1.1611	5.2636	3.2663
H	3.4380	6.0884	3.7606
H	5.4311	4.9626	2.8226
H	5.1638	2.9692	1.3505
H	2.4908	3.1006	-1.1584
H	4.0040	2.1887	-0.9682
H	2.5272	1.3653	-1.5123
H	-0.5772	4.0570	2.8048
H	-1.1237	3.7964	1.1305
H	-1.1641	2.4623	2.3087
H	0.3198	-3.6367	-1.7661
H	1.5611	-2.5970	-1.0286
H	2.0034	-3.7161	-2.3213
H	4.3141	0.6239	0.9702
H	2.9053	-0.3305	0.5058
H	3.0234	0.3449	2.1461
H	1.2885	-0.4058	-0.6332
C	-0.1587	-1.6235	0.8362
H	-0.4954	-2.3864	0.1445
H	0.8734	-1.7149	1.1600
H	-1.3705	1.2884	5.0447
C	-2.2196	0.8502	4.5306
C	-4.4320	-0.2860	3.1781
C	-2.0644	0.0241	3.4283
C	-3.5284	1.1059	4.9498
C	-4.6205	0.5485	4.2856
C	-3.1404	-0.5425	2.7539
H	-3.6923	1.7514	5.8072
H	-5.6268	0.7639	4.6309
H	-5.2828	-0.7239	2.6635
N	-0.8628	-0.3738	2.8033
C	-1.1074	-1.1591	1.7348
C	-2.6141	-1.3970	1.6242
C	0.4214	-0.0936	3.4175
H	1.2099	-0.6092	2.8753
H	0.4080	-0.4494	4.4522
H	0.6247	0.9809	3.4116
C	-3.1930	-0.9820	0.2678
H	-4.2721	-1.1652	0.2667
H	-3.0244	0.0808	0.0720
H	-2.7440	-1.5650	-0.5413
C	-2.8983	-2.8900	1.8890
H	-2.4815	-3.2071	2.8499
H	-2.4638	-3.5066	1.0958
H	-3.9803	-3.0551	1.9063

Cy3+FB C2' adduct (0)

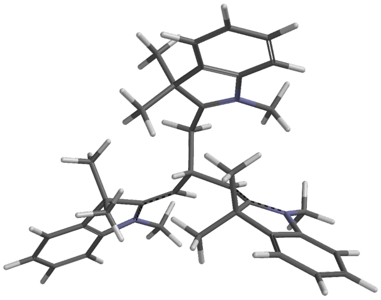

E_{elec}(au)	H°(au)	G°(au)	NImag
-1600.08996	-1599.35592	-1599.44540	0

C	-0.4260	4.7753	4.3277
C	0.1953	4.0574	5.3464
C	0.6561	2.7528	5.1446
C	0.4727	2.1865	3.8865
C	-0.1581	2.8958	2.8612
C	-0.6031	4.1870	3.0691
N	0.8489	0.9306	3.4193
C	0.3959	0.7217	2.1092
C	-0.2004	2.0454	1.6061
C	0.7017	2.6782	0.5348
C	0.5247	-0.4711	1.5016
C	1.3910	-0.1257	4.2373
C	0.1203	-0.9402	0.1255
C	-0.7464	-2.1671	0.3037
C	-1.8243	-2.5947	-0.3783
N	-2.5044	-3.7647	-0.0067
C	-3.7123	-3.8760	-0.6865
C	-3.8017	-2.8666	-1.6498
C	-2.5292	-2.0367	-1.6274
C	-4.7340	-4.8056	-0.5192
C	-5.8520	-4.7038	-1.3532
C	-5.9473	-3.7080	-2.3220
C	-4.9081	-2.7803	-2.4734
C	-1.7000	-2.3753	-2.8832
C	-2.1365	-4.5554	1.1416
C	-1.6377	1.8963	1.0910
C	-2.8400	-0.5372	-1.5753
H	-0.7720	5.7881	4.5081
H	0.3289	4.5147	6.3228
H	1.1376	2.2144	5.9542
H	-1.0872	4.7381	2.2663
H	1.7264	2.7951	0.9026
H	0.7238	2.0587	-0.3637
H	0.3196	3.6656	0.2546
H	0.9863	-1.2675	2.0833
H	1.6804	0.2729	5.2097
H	0.6544	-0.9257	4.3881
H	2.2820	-0.5582	3.7693

H	-0.4201	-2.7865	1.1368
H	-4.6823	-5.5863	0.2333
H	-6.6610	-5.4193	-1.2351
H	-6.8249	-3.6486	-2.9583
H	-4.9743	-2.0015	-3.2293
H	-1.5058	-3.4509	-2.9429
H	-2.2451	-2.0701	-3.7833
H	-0.7390	-1.8540	-2.8707
H	-2.7591	-5.4489	1.1860
H	-2.2574	-3.9903	2.0758
H	-1.0921	-4.8752	1.0613
H	-2.0713	2.8863	0.9140
H	-2.2632	1.3691	1.8182
H	-1.6685	1.3490	0.1469
H	-1.9385	0.0669	-1.7063
H	-3.3148	-0.2642	-0.6283
H	-3.5215	-0.2749	-2.3914
H	-0.4022	-0.1615	-0.4292
C	1.3987	-1.3449	-0.7086
H	1.9847	-2.0593	-0.1227
H	1.0727	-1.8551	-1.6167
C	2.2197	-0.1765	-1.1037
C	2.1511	0.4955	-2.4625
C	2.7681	-0.4975	-3.4843
H	2.1271	-1.3761	-3.6007
H	3.7675	-0.8185	-3.1764
H	2.8437	0.0094	-4.4501
C	0.7393	0.8873	-2.9193
H	0.1079	0.0031	-3.0281
H	0.8149	1.3692	-3.8981
H	0.2550	1.5839	-2.2316
N	3.1130	0.4114	-0.3539
C	3.6124	0.0098	0.9579
H	3.2095	-0.9603	1.2307
H	3.3093	0.7549	1.6960
H	4.7015	-0.0401	0.8997
C	3.0729	1.6694	-2.2576
C	3.6488	1.5655	-1.0003
C	3.4154	2.7296	-3.0814
H	2.9804	2.8320	-4.0707
C	4.3327	3.6671	-2.6018
H	4.6101	4.5103	-3.2263
C	4.8975	3.5409	-1.3304
H	5.6060	4.2845	-0.9806
C	4.5625	2.4742	-0.4960
H	4.9932	2.3761	0.4944

Cy3+FB C2' adduct deprot. (0)

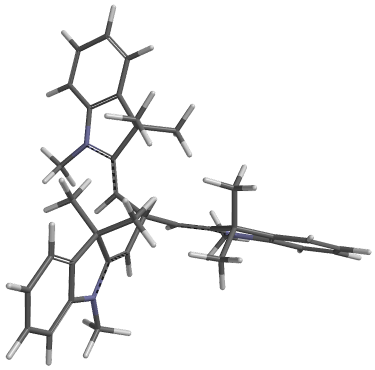

E_{elec}(au)	H°(au)	G°(au)	NImag
-1599.61102	-1598.89260	-1598.98304	0

C	-2.3914	-0.3018	6.5167
C	-1.4617	-1.2512	6.9329
C	-0.5250	-1.7936	6.0467
C	-0.5460	-1.3540	4.7252
C	-1.4786	-0.4021	4.2975
C	-2.3979	0.1269	5.1822
N	0.2630	-1.7221	3.6604
C	-0.1081	-1.0581	2.4791
C	-1.2714	-0.1105	2.8225
C	-0.9036	1.3714	2.6363
C	0.4977	-1.2997	1.3037
C	1.3433	-2.6721	3.7152
C	0.2391	-0.6750	-0.0601
C	-0.8008	-1.5015	-0.8052
C	-1.6971	-1.1154	-1.7298
N	-2.5943	-2.0207	-2.3216
C	-3.3825	-1.4017	-3.2802
C	-3.0562	-0.0424	-3.3474
C	-1.9527	0.2687	-2.3526
C	-4.3684	-1.9437	-4.1013
C	-5.0256	-1.0859	-4.9895
C	-4.7107	0.2688	-5.0598
C	-3.7134	0.7949	-4.2273
C	-0.7069	0.7919	-3.0834
C	-2.6343	-3.4171	-1.9744
C	-2.5351	-0.4619	2.0224
C	-2.4565	1.2988	-1.3283
H	-3.1096	0.1042	7.2223
H	-1.4579	-1.5842	7.9675
H	0.1876	-2.5348	6.3941
H	-3.1198	0.8675	4.8456
H	-0.0132	1.6292	3.2185
H	-0.7092	1.6120	1.5880
H	-1.7322	2.0016	2.9773
H	1.2799	-2.0557	1.2902
H	1.1330	-3.5476	3.0879

H	2.2755	-2.2127	3.3626
H	1.4912	-3.0074	4.7415
H	-0.7781	-2.5573	-0.5414
H	-4.6302	-2.9962	-4.0666
H	-5.7972	-1.4935	-5.6370
H	-5.2343	0.9152	-5.7572
H	-3.4577	1.8508	-4.2746
H	-0.3730	0.0741	-3.8398
H	-0.9369	1.7390	-3.5831
H	0.1168	0.9554	-2.3856
H	-1.6834	-3.9100	-2.2146
H	-3.4317	-3.9158	-2.5257
H	-2.8223	-3.5411	-0.9004
H	-3.3687	0.1734	2.3423
H	-2.8155	-1.5078	2.1833
H	-2.3755	-0.3150	0.9525
H	-1.6970	1.5281	-0.5762
H	-3.3517	0.9344	-0.8149
H	-2.7112	2.2322	-1.8421
H	-0.1269	0.3439	0.0682
C	1.5323	-0.6564	-0.8636
C	2.4708	0.3022	-0.9503
C	2.5830	1.6545	-0.2235
C	1.4656	2.6360	-0.6152
H	0.4839	2.2825	-0.2907
H	1.4370	2.7839	-1.6992
H	1.6455	3.6066	-0.1398
C	2.6315	1.4664	1.3004
H	1.7041	1.0248	1.6701
H	2.7791	2.4342	1.7927
H	3.4577	0.8058	1.5818
N	3.5967	0.1704	-1.7804
C	3.8089	-0.9683	-2.6359
H	4.6995	-0.8148	-3.2447
H	2.9516	-1.1067	-3.3064
H	3.9395	-1.8867	-2.0498
C	3.9155	2.1692	-0.7379
C	4.4548	1.2514	-1.6467
C	4.5868	3.3392	-0.4401
H	4.1640	4.0508	0.2656
C	5.8195	3.5984	-1.0553
H	6.3591	4.5128	-0.8284
C	6.3491	2.6786	-1.9565
H	7.3051	2.8799	-2.4322
C	5.6787	1.4907	-2.2668
H	6.1133	0.7869	-2.9695
H	1.6920	-1.5653	-1.4401

Cy3+FB C2' adduct deprot. and oxidized (+)

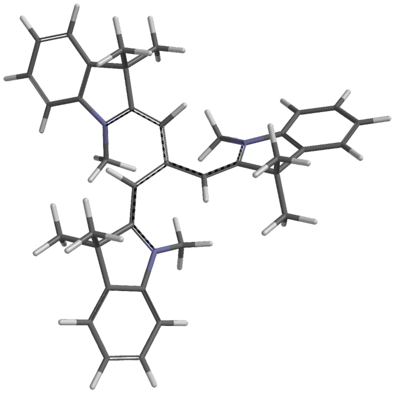

E_{elec}(au)	H°(au)	G°(au)	NImag
-1598.88894	-1598.17747	-1598.26542	0

C	0.9009	-7.0205	0.2711
C	-0.1912	-6.6974	1.0752
C	-0.5875	-5.3696	1.2598
C	0.1517	-4.3918	0.6106
C	1.2397	-4.6977	-0.2023
C	1.6275	-6.0143	-0.3753
N	-0.0317	-2.9935	0.6468
C	0.8542	-2.3729	-0.1773
C	1.8199	-3.4152	-0.7509
C	1.8067	-3.3911	-2.2867
C	0.9644	-1.0354	-0.4780
C	-0.8635	-2.3664	1.6555
C	-0.0097	0.0023	-0.3624
C	-1.3956	-0.3223	-0.4643
C	-2.4955	0.4492	-0.1671
N	-2.5839	1.5326	0.6492
C	-3.8824	2.0833	0.6058
C	-4.6947	1.2937	-0.2030
C	-3.8819	0.1401	-0.7413
C	-4.3522	3.2226	1.2426
C	-5.6970	3.5524	1.0500
C	-6.5263	2.7676	0.2500
C	-6.0257	1.6249	-0.3835
C	-4.4032	-1.2044	-0.1995
C	-1.6233	1.9401	1.6563
C	3.2453	-3.1810	-0.2154
C	-3.8504	0.1297	-2.2771
H	1.1871	-8.0597	0.1424
H	-0.7494	-7.4880	1.5675
H	-1.4441	-5.1256	1.8795
H	2.4803	-6.2628	-1.0015
H	0.7936	-3.5415	-2.6725
H	2.1840	-2.4337	-2.6596
H	2.4487	-4.1891	-2.6734
H	1.9031	-0.7543	-0.9469

H	-0.4482	-1.3904	1.9072
H	-1.8936	-2.2336	1.3124
H	-0.8555	-2.9956	2.5485
H	-1.6251	-1.2794	-0.9240
H	-3.7100	3.8435	1.8584
H	-6.0968	4.4397	1.5315
H	-7.5665	3.0469	0.1148
H	-6.6696	1.0105	-1.0071
H	-4.4080	-1.2107	0.8950
H	-5.4259	-1.3684	-0.5537
H	-3.7795	-2.0321	-0.5531
H	-0.9927	1.0892	1.9149
H	-2.1715	2.2577	2.5462
H	-0.9862	2.7584	1.3083
H	3.9033	-3.9830	-0.5649
H	3.2563	-3.1734	0.8791
H	3.6436	-2.2274	-0.5777
H	-3.2163	-0.6837	-2.6434
H	-3.4646	1.0767	-2.6672
H	-4.8629	-0.0202	-2.6657
C	0.4034	1.3645	-0.4639
C	1.6241	1.9278	-0.1706
C	2.0549	3.2803	-0.7469
C	2.0338	3.2508	-2.2828
H	1.0126	3.1104	-2.6506
H	2.6586	2.4387	-2.6674
H	2.4151	4.1992	-2.6751
C	1.1527	4.4086	-0.2124
H	0.1258	4.2866	-0.5721
H	1.5279	5.3743	-0.5663
H	1.1436	4.4184	0.8821
N	2.6057	1.4590	0.6447
C	2.4743	0.4238	1.6517
H	3.0288	0.7346	2.5400
H	2.8563	-0.5397	1.3019
H	1.4219	0.3103	1.9130
C	3.4598	3.4044	-0.2065
C	3.7346	2.3045	0.6011
C	4.4154	4.3885	-0.3866
H	4.2078	5.2547	-1.0090
C	5.6552	4.2459	0.2459
H	6.4195	5.0049	0.1108
C	5.9177	3.1334	1.0441
H	6.8862	3.0313	1.5242
C	4.9564	2.1369	1.2362
H	5.1709	1.2682	1.8498
H	-0.3111	2.0446	-0.9194

XI. X-Ray Crystal Structure of TMP

Experimental. Single yellow needle-shaped crystals of TMP recrystallised from a mixture of DCM and ether by Vapor Diffusion. A suitable crystal with dimensions $0.27 \times 0.14 \times 0.04$ mm^3 was selected and mounted on a nylon loop with paratone oil on a XtaLAB Synergy, Dualflex, HyPix diffractometer. The crystal was kept at a steady T = 100.00(10) K during data collection. The structure was solved with the ShelXT (Sheldrick, 2015) solution program using dual methods and by using O. V. Dolomanov, L. J. Bourhis, R. J. Gildea, J. A. K. Howard and H. Puschmann,Olex2: a complete structure solution, refinement and analysis program.J. Appl. Cryst. (2009). 42, 339-341. as the graphical interface. The model was refined with ShelXL 2018/3 (Sheldrick, 2015) using full matrix least squares minimisation on F^2.

Crystal Data. $C_{17}H_{18}IN$, M_r = 363.22, monoclinic, $P2_1/n$ (No. 14), a = 9.09021(9) Å, b = 12.65201(12) Å, c = 13.54509(12) Å, β = 95.7490(8)°, α = γ = 90°, V = 1549.98(3) Å^3, T = 100.00(10) K, Z = 4, Z' = 1, μ(Cu K$_\alpha$) = 16.107, 22060 reflections measured, 3275 unique (R_{int} = 0.0487) which were used in all calculations. The final wR_2 was 0.0654 (all data) and R_1 was 0.0240 ($I \geq 2\sigma(I)$).

**Compound**	**BB124A**
Formula	$C_{17}H_{18}IN$
CCDC	2324987
$D_{calc.}$/ g cm^{-3}	1.557
μ/mm^{-1}	16.107
Formula Weight	363.22
Colour	yellow
Shape	needle-shaped
Size/mm^3	0.27×0.14×0.04
T/K	100.00(10)
Crystal System	monoclinic
Space Group	$P2_1/n$
a/Å	9.09021(9)
b/Å	12.65201(12)
c/Å	13.54509(12)
α/°	90
β/°	95.7490(8)
γ/°	90
V/Å^3	1549.98(3)
Z	4
Z'	1
Wavelength/Å	1.54184
Radiation type	Cu K$_\alpha$
Θ_{min}/°	4.794
Θ_{max}/°	77.649
Measured Refl's.	22060
Indep't Refl's	3275
Refl's I≥2 σ(I)	3127
R_{int}	0.0487
Parameters	175
Restraints	0
Largest Peak	1.015
Deepest Hole	-0.897
GooF	1.035
wR_2 (all data)	0.0654
wR_2	0.0648
R_1 (all data)	0.0248
R_1	0.0240

Structure Quality Indicators

Reflections:	d min (CuKα) 2Θ=155.3° 0.79	I/σ(I) 38.3	Rint m=6.92 4.87%	Full 135.4° 99% to 155.3° 100
Refinement:	Shift -0.003	Max Peak 1.0	Min Peak -0.9	GooF 1.035

A yellow needle-shaped crystal with dimensions 0.27×0.14×0.04 mm^3 was mounted on a nylon loop with paratone oil. Data were collected using a XtaLAB Synergy, Dualflex, HyPix diffractometer equipped with an Oxford Cryosystems 800 low-temperature device, operating at T = 100.00(10) K.

MSU Data were measured using w scans using Cu K$_a$ radiation (micro-focus sealed X-ray tube, 50 kV, 1 mA). The total number of runs and images was based on the strategy calculation from the program CrysAlisPro 1.171.43.104a (Rigaku OD, 2023). The achieved resolution was Θ = 77.649.

Cell parameters were retrieved using the CrysAlisPro 1.171.43.104a (Rigaku OD, 2023) software and

refined using CrysAlisPro 1.171.43.104a (Rigaku OD, 2023) on 13230 reflections, 60 % of the observed reflections. Data reduction was performed using the CrysAlisPro 1.171.43.104a (Rigaku OD, 2023) software which corrects for Lorentz polarization. The final completeness is 100.00 out to 77.649 in Θ CrysAlisPro 1.171.43.104a (Rigaku Oxford Diffraction, 2023) Numerical absorption correction based on gaussian integration over a multifaceted crystal model Empirical absorption correction using spherical harmonics, implemented in SCALE3 ABSPACK scaling algorithm.

The structure was solved in the space group $P2_1/n$ (# 14) by using dual methods using the ShelXT (Sheldrick, 2015) structure solution program. The structure was refined by Least Squares ShelXL incorporated in Olex2 software program. All non-hydrogen atoms were refined anisotropically. Hydrogen atom positions were calculated geometrically and refined using the riding model, except for the hydrogen atom on the non-carbon atom(s) which were found by difference Fourier methods and refined isotropically when data permits.

CCDC 2324987 contains the supplementary crystallographic data for this paper. The data can be obtained free of charge from The Cambridge Crystallographic Data Centre via www.ccdc.cam.ac.uk/structures.

There is a single formula unit in the asymmetric unit, which is represented by the reported sum formula. In other words: Z is 4 and Z' is 1. The moiety formula is C17 H18 N, I.

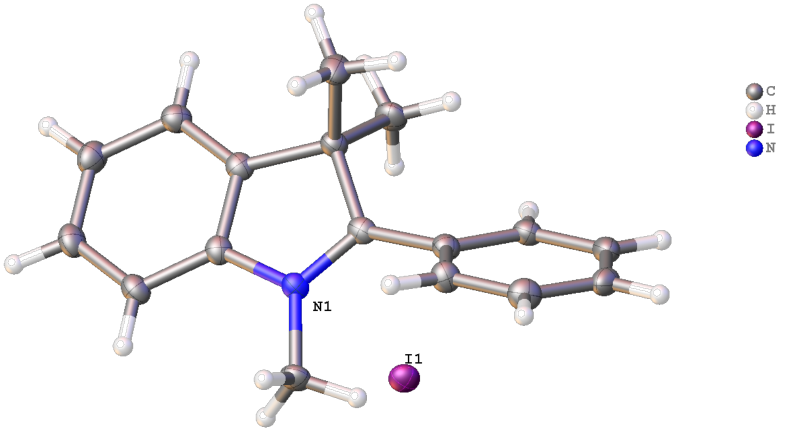

Figure S7. Drawing of compound at 50% ellipsoids showing labeling of hetero atoms.

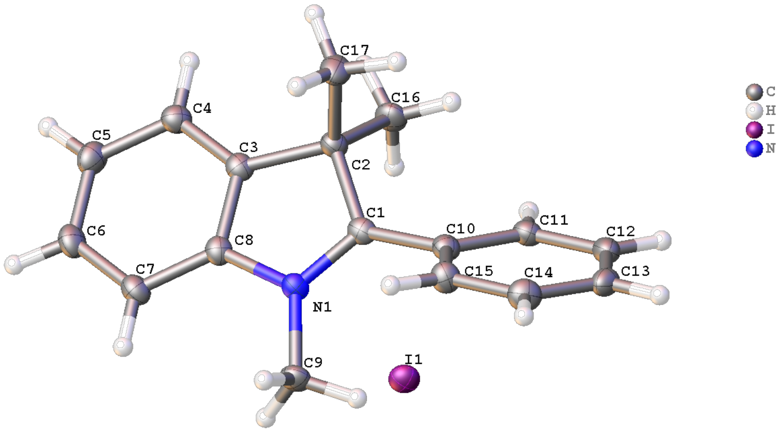

Figure S8. Drawing of compound at 50% ellipsoids showing labeling of non-hydrogen atoms.

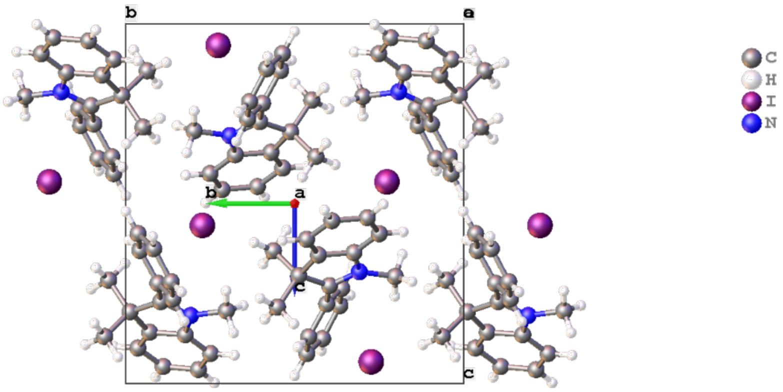

Figure S9. Packing diagram of **TMP** viewed along the a axis.

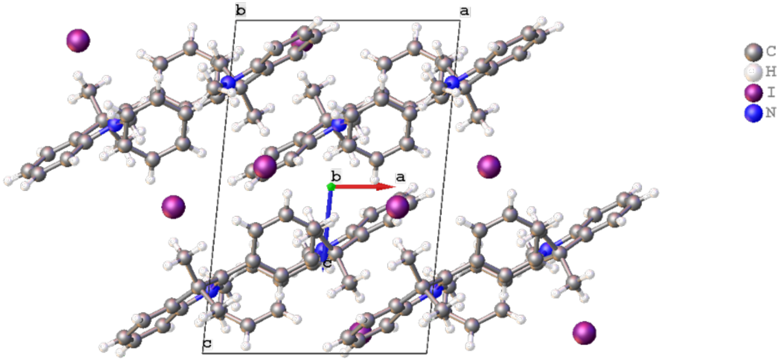

Figure S10. Packing diagram of **TMP** viewed along the b axis.

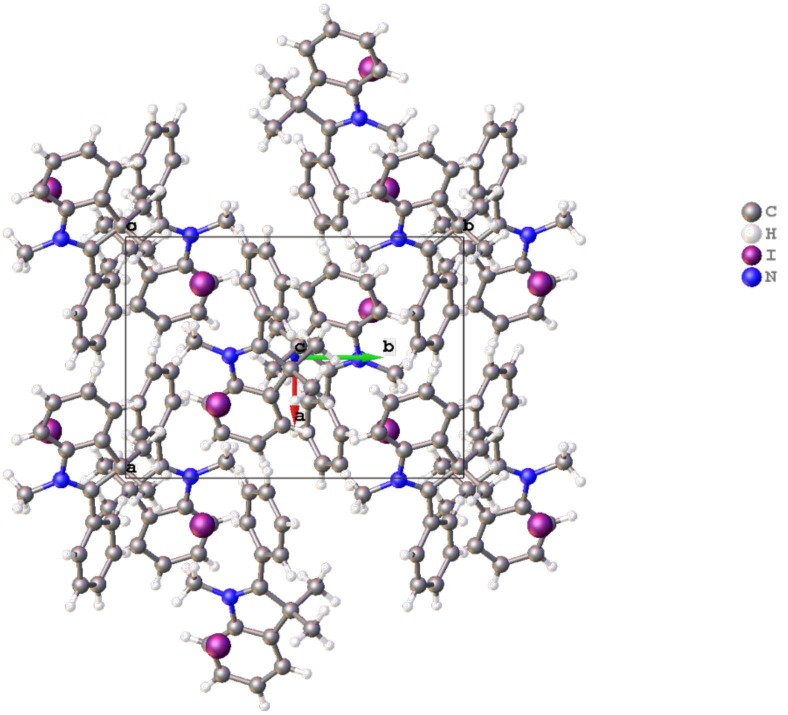

Figure S11. Packing diagram of TMP viewed along the c axis.

Reflection Statistics

Total reflections (after filtering)	22669	Unique reflections	3275
Completeness	0.992	Mean I/σ	27.88
hkl_{max} collected	(11, 16, 14)	hkl_{min} collected	(-11, -15, -17)
hkl_{max} used	(11, 16, 17)	hkl_{min} used	(-11, 0, 0)
Lim d_{max} collected	100.0	Lim d_{min} collected	0.77
d_{max} used	13.48	d_{min} used	0.79
Friedel pairs	2470	Friedel pairs merged	1
Inconsistent equivalents	1	R_{int}	0.0487
R_{sigma}	0.0261	Intensity transformed	0
Omitted reflections	0	Omitted by user (OMIT hkl)	0
Multiplicity	(3295, 2293, 1302, 751, 455, 275, 189, 93, 63, 48, 40, 19, 11, 2)	Maximum multiplicity	27
Removed systematic absences	609	Filtered off (Shel/OMIT)	0

Table S1: Fractional Atomic Coordinates (×10^4) and Equivalent Isotropic Displacement Parameters (Å^2×10^3) for TMP. U_{eq} is defined as 1/3 of the trace of the orthogonalised U_{ij}.

Atom	x	y	z	U_{eq}
N1	4926.1(18)	3084.0(14)	6892.1(12)	17.0(3)
C1	4509(2)	3968.3(16)	7277.4(14)	17.1(4)
C2	5480(2)	4879.4(16)	6997.4(14)	17.9(4)
C3	6575(2)	4297.2(16)	6427.7(14)	18.0(4)
C4	7799(2)	4644.4(17)	5989.3(15)	21.0(4)
C5	8577(2)	3896.2(19)	5475.9(16)	24.5(4)
C6	8124(3)	2850.0(19)	5390.6(19)	23.4(5)
C7	6898(3)	2494.0(19)	5835.5(16)	21.1(4)
C8	6177(2)	3243.3(16)	6357.4(14)	18.1(4)
C9	4325(3)	2020.8(18)	7021.8(17)	21.5(4)
C10	3168(2)	4052.0(16)	7788.1(14)	18.5(4)
C11	3181(2)	4582.7(16)	8696.1(15)	19.5(4)
C12	1899(2)	4640.0(17)	9167.9(16)	22.3(4)
C13	596(2)	4173.2(18)	8747.5(16)	22.9(4)
C14	568(2)	3662.1(18)	7835.0(16)	24.8(4)
C15	1840(2)	3606.8(17)	7353.5(15)	21.7(4)
C16	6242(2)	5508.3(17)	7874.3(16)	23.2(4)
C17	4508(2)	5628.5(19)	6309.0(16)	25.3(5)
I1	6954.1(2)	2733.2(2)	9394.9(2)	20.86(7)

Table S2. Anisotropic Displacement Parameters (×10^4) for TMP. The anisotropic displacement factor exponent takes the form: $-2p^2[h^2a*^2 \times U_{11}+ \ldots +2hka* \times b* \times U_{12}]$

Atom	U_{11}	U_{22}	U_{33}	U_{23}	U_{13}	U_{12}
N1	14.4(8)	17.0(8)	19.2(8)	-1.7(7)	-0.2(6)	-0.7(7)
C1	16.0(9)	18.0(9)	16.3(9)	-0.8(7)	-3.7(7)	0.1(7)
C2	16.6(9)	16.0(9)	20.9(9)	-0.4(7)	1.9(7)	0.5(7)
C3	15.3(9)	19.5(10)	18.6(9)	0.3(7)	-1.3(7)	2.2(7)
C4	17.9(9)	20.3(10)	24.5(10)	1.4(8)	1.1(8)	1.0(8)
C5	17.6(10)	30.3(12)	26.0(10)	3.5(9)	4.7(8)	3.0(9)
C6	20.0(11)	26.5(11)	23.7(10)	-1.5(8)	2.4(8)	7.6(8)
C7	20.1(10)	19.9(9)	22.9(10)	-2.3(8)	-0.3(8)	3.6(9)
C8	14.6(9)	21.4(10)	17.7(9)	-0.3(7)	-0.6(7)	2.1(7)
C9	22.8(10)	15.2(9)	26.4(11)	-2.4(9)	2.1(8)	-4.0(9)
C10	17.6(9)	16.8(9)	21.2(9)	0.7(7)	1.9(7)	1.5(7)
C11	20.1(10)	15.5(9)	22.5(9)	-0.2(7)	1.2(8)	0.1(7)
C12	29.5(11)	16.9(10)	21.1(9)	0.4(8)	6.2(8)	1.4(8)
C13	21.1(10)	22.0(10)	27.0(10)	4.8(8)	8.9(8)	3.6(8)
C14	18.7(10)	26.2(11)	28.9(11)	0.7(9)	-0.5(8)	-0.2(8)
C15	17.9(10)	23.7(10)	23.2(9)	-2.4(8)	0.8(8)	-0.5(8)
C16	25.3(10)	18.1(10)	26.2(10)	-4.2(8)	1.9(8)	-5.2(8)
C17	24.4(11)	25.7(11)	26.1(10)	5.3(8)	3.8(8)	9.3(9)
I1	18.9(1)	19.18(11)	24.39(10)	-0.78(4)	1.56(6)	-4.32(4)

Table S3: Bond Lengths in Å for TMP.

Atom	Atom	Length/Å
N1	C1	1.307(3)
N1	C8	1.423(3)
N1	C9	1.469(3)
C1	C2	1.523(3)
C1	C10	1.464(3)
C2	C3	1.511(3)
C2	C16	1.536(3)
C2	C17	1.544(3)
C3	C4	1.384(3)
C3	C8	1.382(3)

Atom	Atom	Length/Å
C4	C5	1.406(3)
C5	C6	1.387(3)
C6	C7	1.394(3)
C7	C8	1.386(3)
C10	C11	1.400(3)
C10	C15	1.407(3)
C11	C12	1.386(3)
C12	C13	1.393(3)
C13	C14	1.393(3)
C14	C15	1.385(3)

Table S4. Bond Angles in ° for TMP.

Atom	Atom	Atom	Angle/°
C1	N1	C8	111.16(17)
C1	N1	C9	127.57(18)
C8	N1	C9	121.10(19)
N1	C1	C2	110.47(17)
N1	C1	C10	122.63(18)
C10	C1	C2	126.43(18)
C1	C2	C16	115.30(16)
C1	C2	C17	107.68(17)
C3	C2	C1	100.74(16)
C3	C2	C16	112.25(17)
C3	C2	C17	110.94(16)
C16	C2	C17	109.60(17)
C4	C3	C2	131.56(19)
C8	C3	C2	108.92(17)
C8	C3	C4	119.51(19)

Atom	Atom	Atom	Angle/°
C3	C4	C5	117.6(2)
C6	C5	C4	121.6(2)
C5	C6	C7	121.0(2)
C8	C7	C6	116.0(2)
C3	C8	N1	108.58(17)
C3	C8	C7	124.1(2)
C7	C8	N1	127.3(2)
C11	C10	C1	121.01(18)
C11	C10	C15	119.41(18)
C15	C10	C1	119.58(18)
C12	C11	C10	119.75(19)
C11	C12	C13	120.6(2)
C12	C13	C14	119.9(2)
C15	C14	C13	120.0(2)
C14	C15	C10	120.31(19)

Table S5.Torsion Angles in ° for TMP.

Atom	Atom	Atom	Atom	Angle/°
N1	C1	C2	C3	-3.2(2)
N1	C1	C2	C16	-124.24(19)
N1	C1	C2	C17	113.06(18)
N1	C1	C10	C11	134.5(2)
N1	C1	C10	C15	-46.4(3)
C1	N1	C8	C3	0.9(2)
C1	N1	C8	C7	-178.5(2)
C1	C2	C3	C4	-177.6(2)
C1	C2	C3	C8	3.6(2)
C1	C10	C11	C12	-179.33(19)
C1	C10	C15	C14	178.8(2)
C2	C1	C10	C11	-54.1(3)
C2	C1	C10	C15	124.9(2)
C2	C3	C4	C5	-177.9(2)
C2	C3	C8	N1	-3.0(2)
C2	C3	C8	C7	176.38(19)
C3	C4	C5	C6	1.3(3)
C4	C3	C8	N1	178.07(17)
C4	C3	C8	C7	-2.6(3)

Atom	Atom	Atom	Atom	Angle/°
C4	C5	C6	C7	-1.7(4)
C5	C6	C7	C8	0.0(3)
C6	C7	C8	N1	-178.7(2)
C6	C7	C8	C3	2.1(3)
C8	N1	C1	C2	1.6(2)
C8	N1	C1	C10	174.22(17)
C8	C3	C4	C5	0.8(3)
C9	N1	C1	C2	176.94(19)
C9	N1	C1	C10	-10.5(3)
C9	N1	C8	C3	-174.80(18)
C9	N1	C8	C7	5.9(3)
C10	C1	C2	C3	-175.43(18)
C10	C1	C2	C16	63.5(3)
C10	C1	C2	C17	-59.2(2)
C10	C11	C12	C13	0.1(3)
C11	C10	C15	C14	-2.1(3)
C11	C12	C13	C14	-1.3(3)
C12	C13	C14	C15	0.8(3)
C13	C14	C15	C10	0.9(3)
C15	C10	C11	C12	1.7(3)
C16	C2	C3	C4	-54.4(3)
C16	C2	C3	C8	126.83(18)
C17	C2	C3	C4	68.6(3)
C17	C2	C3	C8	-110.2(2)

Table S6. Hydrogen Fractional Atomic Coordinates (×10^4) and Equivalent Isotropic Displacement Parameters (Å^2×10^3) for TMP. U_{eq} is defined as 1/3 of the trace of the orthogonalised U_{ij}.

Atom	x	y	z	U_{eq}
H4	8103.66	5362.57	6033.75	25
H5	9432.22	4112.27	5180.32	29
H6	8658.3	2368.8	5023.2	28
H7	6575.47	1780.08	5784.02	25
H9A	3704.42	2026.98	7573.2	32
H9B	5140.2	1519.93	7167.28	32
H9C	3729.24	1806.38	6411.61	32
H11	4065.89	4902.3	8987.82	23
H12	1908.55	5000.83	9783.61	27
H13	-272.1	4203.46	9082.74	28
H14	-322.97	3351.63	7543.13	30
H15	1815.01	3266.72	6726.8	26
H16A	5494.84	5883.35	8213.45	35
H16B	6928.24	6020.4	7629.05	35
H16C	6789.9	5022.5	8339.86	35
H17A	4057.83	5231.89	5734.84	38
H17B	5120.42	6199.33	6081.77	38
H17C	3729.17	5929.7	6673.43	38

XII. References

(1) Štacková, L.; Štacko, P.; Klán, P. Approach to a Substituted Heptamethine Cyanine Chain by the Ring Opening of Zincke Salts. *J. Am. Chem. Soc.* **2019**, *141*, 7155.

(2) Owens, E. A.; Bruschi, N.; Tawney, J. G.; Henary, M. A microwave-assisted and environmentally benign approach to the synthesis of near-infrared fluorescent pentamethine cyanine dyes. *Dyes Pigm.* **2015**, *113*, 27.

(3) Liu, C. G.; Wang, M. Y.; Xu, Y. H.; Li, Y. B.; Liu, Q. Manganese-Catalyzed Asymmetric Hydrogenation of -Indoles. *Angew. Chem. Int. Ed.* **2022**, *61*.
